# Morphotypes, preservation, and taphonomy of dinosaur footprints, tail traces, and swim tracks in the largest tracksite in the world: Carreras Pampa (Upper Cretaceous), Torotoro National Park, Bolivia

**DOI:** 10.1371/journal.pone.0335973

**Published:** 2025-12-03

**Authors:** Raúl Esperante, Jeremy A. McLarty, Kevin E. Nick, Lance R. Pompe, Roberto E. Biaggi, Helen D. Baltazar Medina, Nelson A. Llempen, Ángela B. Limachi Silvestri, Lourdes Lidia Mamani Quispe, Antonio Joaquín Garre Cano, Wilson Quiroga Saavedra, Germán Rocha Rodríguez

**Affiliations:** 1 Geoscience Research Institute, Loma Linda, California, United States of America,; 2 Department of Biological Sciences, Southwestern Adventist University, Keene, Texas, United States of America; 3 Earth and Biological Sciences Department, Loma Linda University, Loma Linda, California, United States of America; 4 Zona Bajo San Isidro, Condominios Bicentenario Bloque lX piso 4-A, La Paz, Bolivia; 5 Departamento de La Paz, Viacha, Ingavi, Bolivia,; 6 Universidad Mayor de San Andrés, La Paz, Bolivia; 7 Sarco, Sarcobamba, Cochabamba, Bolivia; 8 Universidad Adventista de Bolivia, Cochabamba, Bolivia; 9 Museo de Historia Natural Noel Kempff Mercado, Santa Cruz de la Sierra, Bolivia; Universiteit Maastricht, NETHERLANDS, KINGDOM OF THE

## Abstract

The Carreras Pampa tracksite in the Torotoro National Park, Bolivia, records a wealth of dinosaur tracks, tail traces, and swim tracks. In this study, we report 1321 trackways and 289 solitary tracks, totaling 16,600 theropod tracks, 280 swim trackways, totaling 1,378 swim tracks, and several trackways with tail traces. Numerous avian tracks occur locally and are associated with the theropod tracks. These tracks and trackways are located within nine study sites of the same exposed tracking surface with a total area of approximately 7485 m^2^. We describe eight preservation styles and 11 morphotypes for walking tracks, and three morphotypes for swim tracks. Tracks range in size from miniature to large. The range of track sizes and the diversity of morphotypes suggest that the Carreras Pampa tracksite represents a diverse group of trackmakers. Track depths vary from very shallow to very deep both within and among trackways, suggesting that the rheological conditions of the sites changed in time and space. We present estimates of the speeds, gaits, and sizes of trackmakers and propose diverse behaviors indicated by the trackways. Notably, trackways at the Carreras Pampa tracksite indicate that a significantly higher proportion of trackmakers had relative stride lengths above 2.0 compared to other sites. The trackways show a strong, bimodal orientation, probably moving along the paleocoastline. Other forms of bioturbation and fossils were found in association with the tracks. We compare our findings at the Carreras Pampa track site to those from other sites in various locations. The quality of preservation, the exceptionally high number of tracks, and the diversity of behaviors recorded make the Carreras Pampa tracksite one of the premier dinosaur track sites in the world.

## Introduction

Dinosaur trackways offer data from which the morphologies and behaviors of trackmakers in their environments can be deduced. At the Carreras Pampa tracksite in Torotoro National Park, Bolivia, data from 1275 trackways have been collected and analyzed, creating a detailed picture of dinosaur size distributions and behaviors that included turns, tail dragging, and swimming. The large number of trackways allows for the characterization of populations that left their marks on a single bedding surface during the Maastrichtian.

### Dinosaur tracks in Bolivia

Bolivia has one of the most extensive and diverse records of dinosaur tracksites in the world, spanning the Triassic, Jurassic, and Cretaceous. However, despite the abundance of tracksites, few scientific studies have been published. Triassic chirotheriid and dinosaurian tracks have been reported at the Ruditayoj tracksite by Apesteguía et al. [1] Apesteguía and Gallina [2] reported probable stegosaurian tracks at the Tunasniyoj tracksite, which is near the Jurassic-Cretaceous boundary of the Incapampa-Icla syncline. A detailed study of a tracksite in the Castellón Formation of the Jurassic-Cretaceous strata featuring abundant sauropod, theropod, and a few ornithopod tracks has been reported by Méndez Torrez et al. [3]. In the Upper Campanian (Upper Cretaceous) Torotoro Formation located in the Torotoro National Park (TTNP), Apesteguía et al. [4] reported probable dromaeosaurid tracks. Many tracksites have been discovered in the Upper Cretaceous (Maastrichtian) strata of the El Molino Formation in the Torotoro National Park (TTNP) and its vicinity, including Sucre, Maragua Syncline, Miraflores, and Camargo.

Félix Celso Reyes [5] reported the discovery of dinosaur tridactyl ichnites in the El Molino Formation by the Austrian geologist Andreas Unterladstätter in 1947, later corroborated and expanded by Leonardo Branisa [6], who visited the area between 1961 and 1968 and registered a theropod trackway NW of Torotoro towards the Umajalanta Cavern, and a quadrupedal trackway on the east side of Torotoro by the river, which was later described by Leonardi [7]. Unpublished work in the area included that of K.E. Campbell of the Natural History Museum of Los Angeles County, California, in 1983 and Giancarlo Ligabue of the Center of Studies and Research Ligabue in Venice, Italy (1983 − 1984). Leonardi [8,9] briefly noted the occurrence of dinosaur trackways near Torotoro and published a short description of the Huayllas tracksite on the outskirts of Torotoro, with purported ankylosaur and many theropod trackways [7].

Titanosaurid and theropod trackways have also been reported from several tracksites in the El Molino Formation [10–12]. Reported ankylosaurian tracks from the Niñu Mayu tracksite west of the Maragua Syncline were studied by Riguetti et al. [13] Of great significance is the study of the Cal Orcko tracksite, located on the outskirts of Sucre, preserved on a nearly vertical kilometer-long, limestone wall of rock within the quarry operated by the FANCESA cement company. The discovery of these tracks by workers in 1994 prompted engineer Hugo Hymann to halt operations and notify Dr. Mario Suarez Riglos, who studied, described, and published the first scientific report on these trackways. The studies by Lockley et al. [10], and in more detail by Meyer et al. [11] and Meyer et al. [14], highlighted the abundance of ankylosaur, sauropod, and theropod tracks in this extensive tracksite of the El Molino Formation. In their study, Meyer et al. [14] reported 12,093 individual footprints but estimated that the total number of tracks was likely closer to 14,000), comprising nine different morphotypes.

The occurrence of eight parallel sauropod (probable titanosaur) trackways at a tracksite in Torotoro, eleven parallel titanosaur trackways at the Humaca tracksite, and two parallel titanosaur trackways at the Cal Orcko tracksite, all near Sucre, has been interpreted as indicating some type of social behavior [10]. Herd behavior has been inferred from parallel sauropod trackways in the Castellón Formation at the Entre Ríos tracksite near Tarija [3].

### Overview of our work in the TTNP area

The research team led by Raúl Esperante in the TTNP has documented significant findings, including a new theropod tracksite on the vertical slope of the SE limb of the Torotoro Syncline [15], unusual tracks formed during the kick-off phase of dinosaur movement [16,17], the occurrence of many theropod trackways with well-preserved tail traces [18], the abundance of theropod swim traces [19,20], the occurrence of trackways with stops and turns [21], the abundance of tridactyl undertracks in the Sucusuma tracksite [22], and a new sauropod-theropod ichnosite that became exposed on the NW-facing slope of the Torotoro Syncline during the construction of the new road from Cochabamba to Torotoro [23,24]. In addition to dinosaur tracks, several sites have recently been found in the TTNP with well-preserved avian footprints associated with theropod and ornithopod tracks [24–26].

Here, we document 1321 theropod trackways, 16600 exposed dinosaur tracks (Tables 1 and S1), along with numerous solitary tracks, abundant tail traces and swim tracks, and various bird tracks in the Carreras Pampa study area. We estimate the total number of tracks to be much higher because this account does not include the solitary tracks found at sites CP1, CP5, and CP7 or tracks classified as M1 (described later). Additionally, 1378 theropod swim tracks are recorded, and many tail traces are associated with the theropod tracks.

**Table 1:** Trackways, number of tracks, area, and density of tracks at the Carreras Pampa tracksite. Site CP7 has a low density of tracks because much of its surface has been eroded or altered. The total density figure of 2.61 is calculated using the values for sites CP1-CP6, and CP8 and CP9.

Site	Trackways (2 + tracks)	Solitary tracks	Total number of tracks	Site area (m^2^)	Density (tracks/m^2^)
1	300	NA	5169	1903	2.71
2	447	164	6171	2607	2.36
3	117	16	1036	474	2.18
4	101	35	643	269	2.39
5	72	NA	734	412	1.78
6	89	46	1010	268	3.76
7	83	NA	745	1227	0.60
8	14	3	91	41	2.22
9	98	25	1001	284	3.52
Totals	1321	289	16600	7485	2.22 (2.61)

### Significance

The Carreras Pampa tracksite is significant for multiple reasons, including holding various world records: 1) it has the largest number of dinosaur tracks and theropod tracks globally; 2) it records the highest number of dinosaur trackways in the world; 3) it preserves the highest number of continuous swim trackways; and 4) it contains the most continuous swim trackways worldwide; 5) it preserves a diverse association of theropod and avian tracks; 6) it yields a variety of styles or modes of track preservation and morphotypes related to track formation, the morphology of the autopodium and the rheological conditions of the substrate at the time of impression; 7) the large quantity of tracks and trackways validates inferences of typical and unusual behaviors; and 8) the abundance of tail-drag and swim traces are significant because they indicate specific behaviors during locomotion.

The present study aims to introduce the world’s largest assemblage of dinosaur trackways and swim trackways at the Carreras Pampa ichnosite within the TTNP, Bolivia, detailing of the abundance of tracks and trackways, taphonomic aspects of preservation, and their paleoenvironmental significance. Additionally, we report the occurrence of bird tracks associated with the theropod tracks. Except for the swim traces, this study does not aim to discuss ichnotaxonomic affinities. Published records [14] and our observations confirm Bolivia’s high abundance of tracksites, especially in the Upper Cretaceous layers. In contrast, the record of skeletal remains is scant, consisting mainly of unpublished, occasional, and limited findings. Besides the figures in the text, supporting information is provided in the Supplementary Information, which is illustrated in abundance and is coded hereafter as S# Fig or S# Table.

### Geological setting

#### Regional geology.

**Fig 1:**
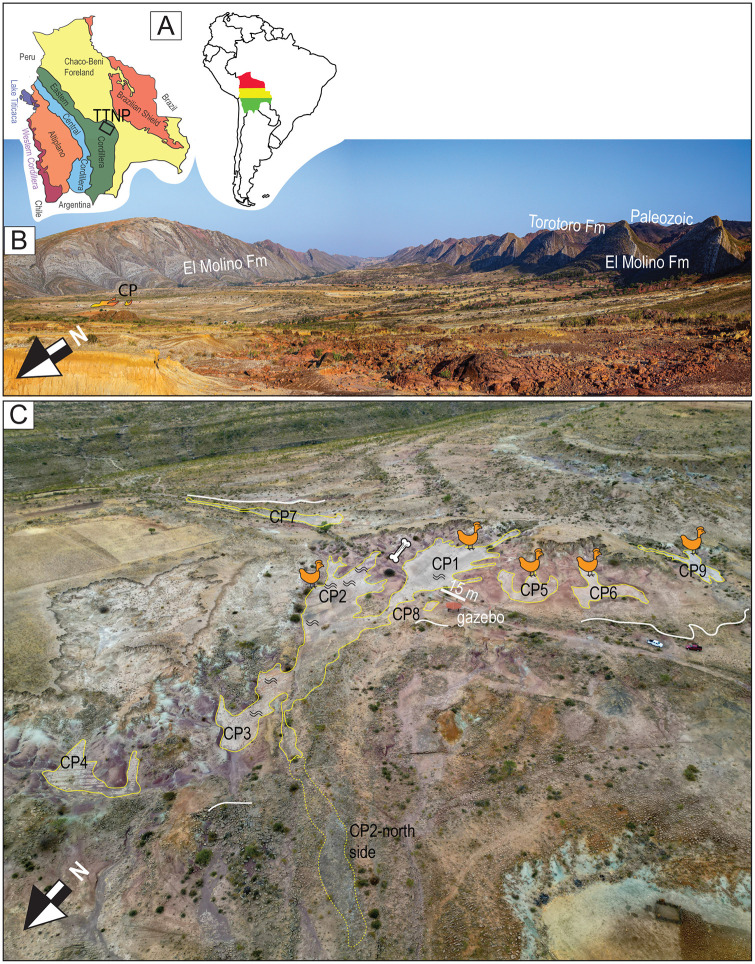
Geographic location and sites at the Carreras Pampa tracksite. A) Map of Bolivia displaying the main geological regions. The black square highlights the location of Torotoro National Park (TTNP) within the Eastern Cordillera. B) Aerial drone view of the Torotoro Syncline from the northwest. The primary geological formations are labeled. The Carreras Pampa tracksite is marked as CP. C) Aerial drone view of the Carreras Pampa tracksite with sites labeled CP1–CP9. A white bar measures 15 m long, corresponding to the length of the walkway sidewall. Two pickup trucks are located on the right side near site CP6. The orange bird icons denote the approximate area of avian tracks. The wave symbol indicates regions with the most noticeable ripples, though they are found throughout the exposed trackbed. The white line indicates where the track-bearing layer is revealed in a vertical cross-section.

The modern Andean Cordillera in Bolivia is divided into four physiographic regions [27] (Fig 1A): 1) the Western Cordillera (Cordillera Occidental), characterized by volcanic activity; 2) the Altiplano Plateau, a high-elevation, crustal-scale piggyback basin; 3) the Eastern Cordillera (Cordillera Oriental); and 4) the Subandean zone. In the Eastern Cordillera and Altiplano, the stratigraphic succession contains basin deposits of the Puca Group that include: 1) a lower succession dominated by Upper Triassic(?) – Lower Cretaceous sandstones of the La Puerta Formation and equivalent units (Kosmina, Macha, Ravelo, and Sucre Formations) unconformably overlying Paleozoic basement rocks; 2) a middle succession of mudstones, carbonates, and evaporites in localized, restricted basins unconformably overlying the lower succession, which include the Lower to Upper Cretaceous Condo, Tarapaya, Miraflores, Aroifilla, Torotoro, and Chaunaca Formations; and 3) a regionally extensive upper succession of carbonates, mudstones, and sandstones conformably overlying the middle succession or locally the Paleozoic basement that includes the Upper Cretaceous to Paleocene El Molino, Santa Lucía, and Impora Formations [28–30].

#### El Molino and Santa Lucía Formations.

The Maastrichtian-Danian El Molino Formation extensively crops out in the Eastern Cordillera and the eastern part of the Altiplano provinces, typically measuring 100–500 m in thickness and locally reaching up to 900 m. Regionally it is divided into three informal lithostratigraphic sequences or members: 1) a lower member consisting of calcareous sandstones, carbonates, and mudstones; many of the carbonate layers contain a variety of microbialites; 2) a middle member dominated by thick uncemented turquoise and burgundy marls, which alternate with thick layers of planar and cross-bedded oolitic and ostracod-rich fine grainstones and calcarenites, along with minor evaporites; the thickness of the combined red siltstones and green marls increases gradually upward; and 3) an upper member consisting of alternating red and green mudstones, siltstones, and calcarenites, along with a well-developed and laterally continuous sequence of several microbialite beds. The Santa Lucía Formation comprises brick-red mudstones and siltstones and transitionally overlies the El Molino. The K-Pg boundary occurs somewhere in the upper member of the El Molino Formation within the predominantly red/green mudstone [31–33].

In the TTNP, the Paleozoic is overlain by approximately 270 m of predominantly cross-bedded red sandstones from the Torotoro Formation, along with an additional 25 m of transitional facies that grade into the El Molino Formation. Paleoenvironmental interpretations for the El Molino vary both stratigraphically and geographically, but are primarily characterized by fluvio-lacustrine conditions with marine influences predominantly near the base [28,32,34,35]. The study area lies within the Torotoro Syncline in the Eastern Cordillera (Fig 1C), within the TTNP boundaries, where the El Molino Formation may reach a thickness of 500 m.

### The Carreras Pampa tracksite

#### Location.

The Carreras Pampa tracksite is located 1.5 km northwest of the outskirts of Torotoro town, accessible via an unpaved road that exits the village toward the southwest and then bends northwest toward the Umajalanta Cave. Approximately 4 km from the bridge at the northern entrance to the town, this unpaved path, marked with a boulder and the TTNP sign, leads to the Carreras Pampa tracksite. A shaded gazebo and sign indicate the Park’s location. The study area is situated on the northeast side of an interfluve, an erosional remnant formed by two tributaries (Quebradas Thipamayu and Chiflón) of the Torotoro River. Outcrops exposed on the interfluve and the tracked bed show slight structural deformation with a general dip of 2° − 8° to the north and northwest.

#### Study sites.

**Fig 2:**
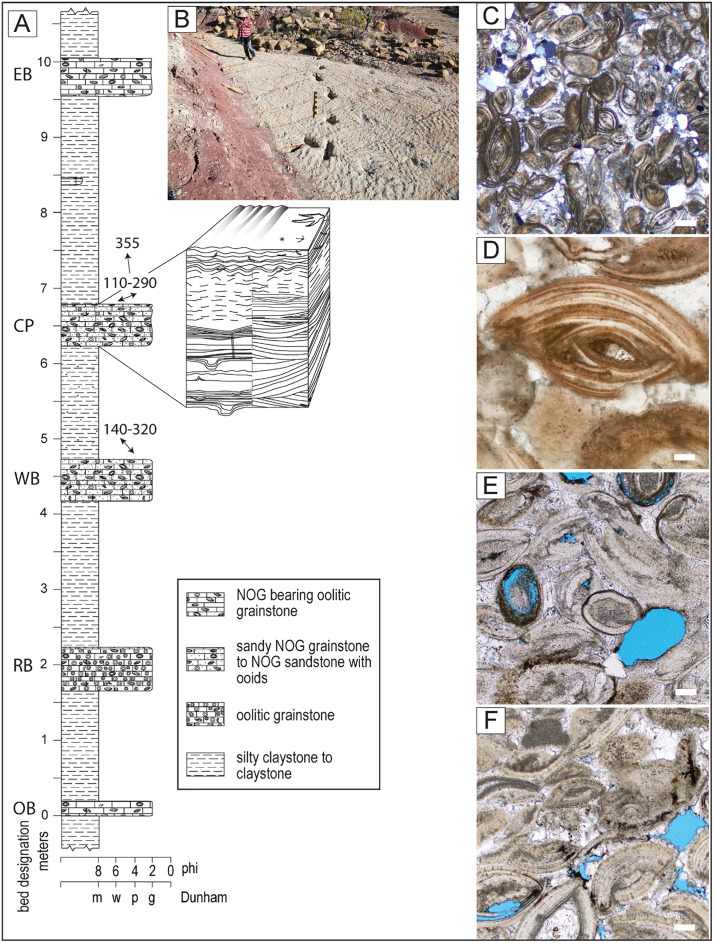
Contextual section for the tracked bed. A) Silty claystones separate five coarser-grained and resistant beds. For bed CP (trackbearing), a more detailed diagram of the range of sedimentary structures and trace fossils shows areas with greater amounts of horizontal and parting laminae, contrasting with localities dominated by trough cross-bedding. The upper portions of CP at most sites are similar and dominated by wave-ripple laminae. Trace fossils on the upper surface of the bed are the topic of this paper. Double-ended arrows show the mean directions of wave ripple crests in WB and CP, and the single-ended arrow shows the mean transport direction derived from sedimentary structures in CP. B) A view of part of the site CP3 with wave ripples and tracks. C–F) Thin section photomicrographs from resistant beds. C) General appearance of nested ostracod grains (NOGs) and quartz grains in the CP bed. Note darker micritic patches and acicular calcite cements. D) A detail of a NOG from CP shows seven or more ostracod valves stacked in a dish-in-dish structure. E) Ellipsoidal NOGs from the OB bed showing moderate packing and moldic porosity. F) NOGs from EB are moderately packed and cemented by euhedral spar, suggesting phreatic conditions. The scale bar for C represents 250 µm, and polarizers are partially crossed. D–F scale bars represent 50 µm with plane-polarized lighting.

The tracksite comprises nine study locations that can be enclosed within an area of approximately 7485 m^2^ (Table 1), designated CP1 through CP9 (Figs 1 and S1 Fig). Sites CP1 and CP2 are the largest. Sites CP1-CP3 are contiguous and nearly continuous, with a combined area of approximately 4984 m^2^. Sites CP4-CP9 are separated from each other and from CP1 and CP2 by remnants of outcrops from overlying units (Figs 1, 2 and S1 Fig), with visual continuity maintained between the sites. Some of the trackways can be traced from one site to another across the banks of sediment. The tracks are located on the upper depositional surface of the same carbonate cemented bed, which averages 45 cm in thickness.

The nine sites expose the same layer containing similar footprint content, lithology, and local stratigraphic setting. The tracked surface and associated sedimentary rocks show no evidence of weathering or pedogenesis during the Cretaceous, i.e., rhizoliths, soil horizons, or soil structures [36,37]. A zone of internal dissolution and alteration of undetermined but pre-modern age affects the carbonate components of the tracked bed in a zone just to the northeast of the gazebo.

The tracked surface at all sites is well exposed and has not been significantly thinned by modern weathering or erosion, except in a few small areas (S1 Fig). Areas of modern weathering are summarized with reference to locations on the site map (S1 Fig). Site CP1 has three small areas where the tracked bed’s surface is weathered, with the topmost layer broken up or removed; consequently, tracks are missing. Many footprints along the northern and western edges of CP2 have been eroded or lost due to weathering. An area of approximately 18 m² at site CP3 is heavily eroded, causing some footprints and swim tracks to be missing or incomplete. Site CP7 is narrow, oriented E-W, and the number of tracks could be much higher if not for the intense surface weathering and abundant growth of shrubs along the southern exposure area. Site CP8 is very small, with only a few trackways exposed and several patches where the tracked surface has eroded. The area near the northern edge of sites CP5, CP6, and CP9 is weathered. In a limited area of site CP2 close to site CP3 (Fig 1C), part of the upper surface of the trackbearing layer has been eroded, and the tracks are preserved as concave epirelief undertracks. Compared to the other sites, site CP8 is very small (S1 Fig), has few trackways and tracks, lacks tail traces, swim tracks, and avian tracks, and its trackways can be visually connected to the trackways in site CP1.

In several areas of the Carreras Pampa tracksite, plant roots growing at the interface of the carbonate-cemented tracked bed and the overlying claystone have recently altered the surface of the tracked layer, creating a network of short to long, sinuous grooves. These grooves become exposed when the overlying sediment is removed. The plants include the trees ‘molle’ (*Shinus molle*), ‘espinillo’ (*Acacia caven*), and several species of shrubs. A root of the *Acacia caven* shrub is shown in Fig 46A. These grooves are easily observed at sites CP1, CP5, CP6, and CP9. The tracked layer, along with the underlying and overlying layers, exhibits no structures indicative of pedogenic development, such as rhizoliths, soil horizons, or soil structures, prior to lithification [36,37].

**Fig 3:**
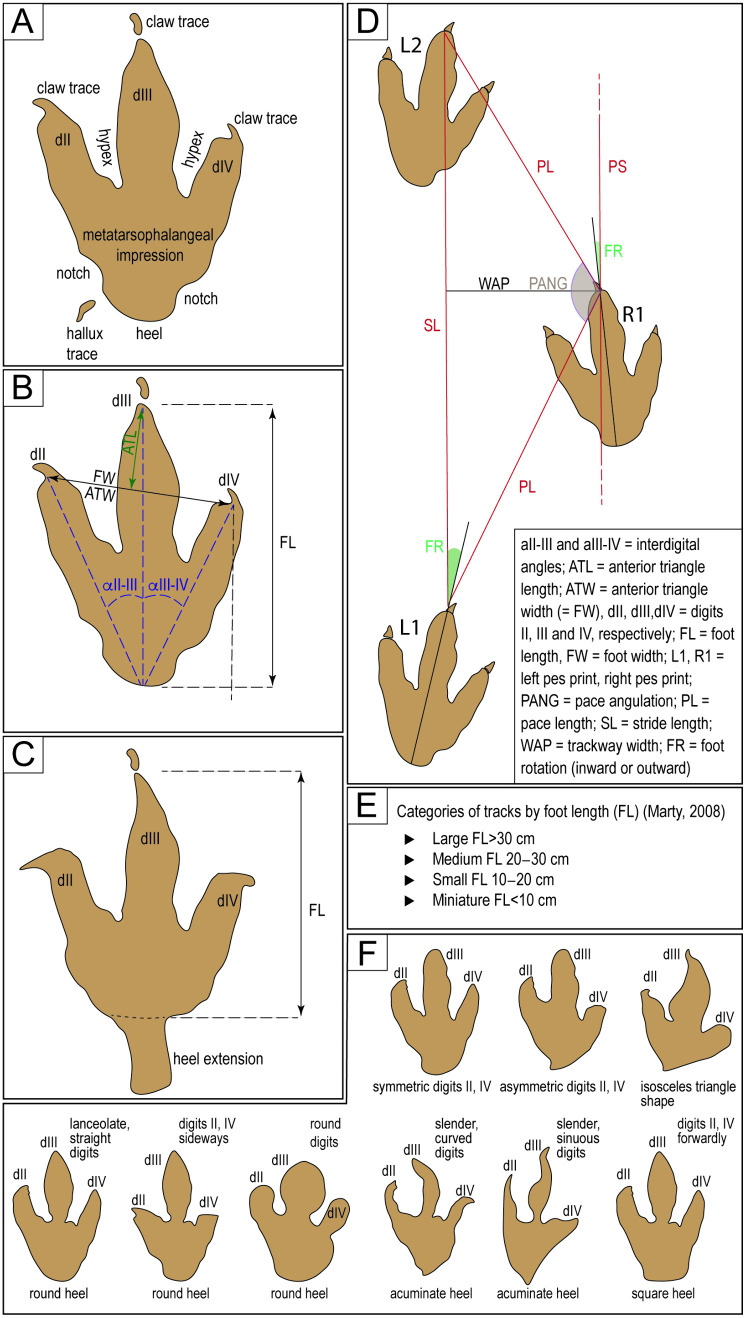
Nomenclature and measurement protocols for dimensions of trackways and tracks. The morphologies in 3F are provided for illustrative purposes, showing the morphologies of the toe and heel prints, and do not correspond to specific morphotypes.

**Fig 4:**
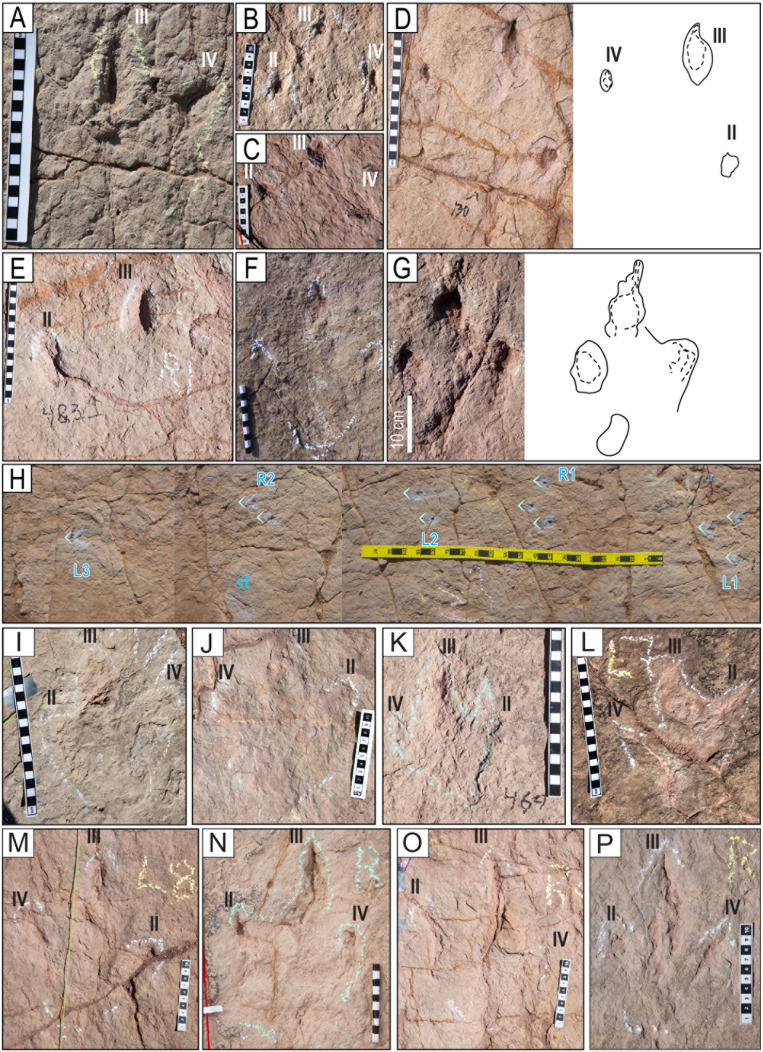
Tracks of the styles of preservation M1 and M2. A–H) Tracks of the style of preservation M1. A) Track of the trackway T22-2-1. B) Track of the trackway T22-2-1. C) Track of the trackway T22-2-36. D) Track of the trackway TO22-2-130. E) Track R1 of the trackway T22-2-483. F) Track of the trackway CPT114. G) Track of the trackway T22-2-31. H) Trackway CP6-62 (2023) from site CP6, each consisting of one, two, or three sets of indentations, st = swim trace. Blue angle bracket symbols (<) are added for clarity. I–P) Tracks of the style of preservation M2. I) Track R11 of the trackway T22-60. J) Track L1 of the trackway T22-2-51. K) Track of the trackway T22-2-469. L) Track L7 of the trackway T22-60. M) Track L8 of the trackway T22-2-8. N) Track R2 of the trackway T22-197. O) Track R8 of the trackway T22-2-8. P) Track R2 of the trackway T22-2-11. Scales are in cm; the scale in H is 1 m.

**Fig 5:**
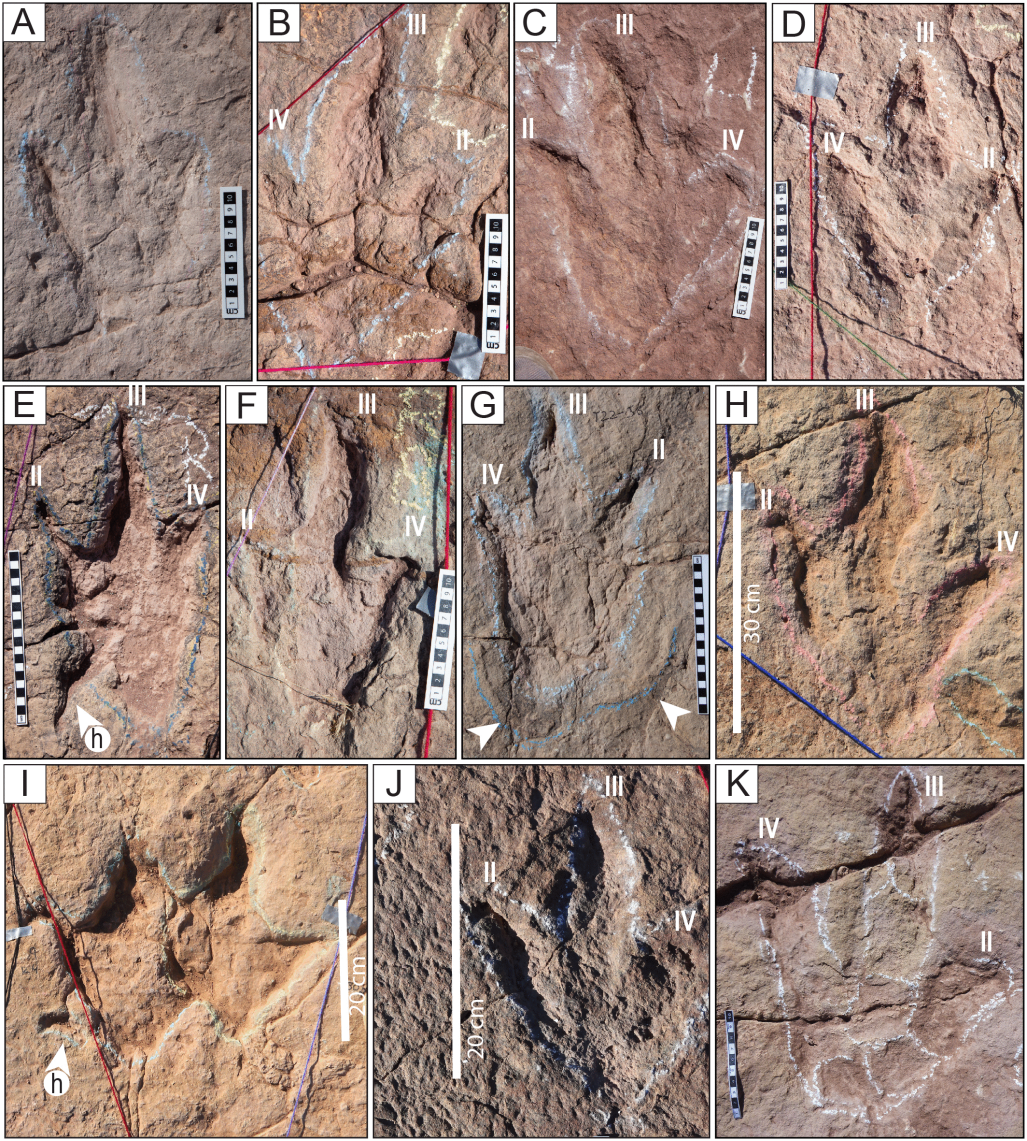
Styles of preservation M3 and M4. A–D) Tracks of the style of preservation M3. A) Track of the trackway T22-2. B) Track L11 of the trackway T22-2-50. C) Track L1 of the trackway T22-2-54. D) Track L3 of the trackway T22-2-3. E–K) Tracks of the style of preservation M4. E) Track R7 of the trackway T22-121. Notice the trace of the hallux (white arrowhead on the lower left corner). F) Track R8 of the trackway T22-2-49. G) Track L4 of trackway T22-56, showing a displacement rim (white arrowheads) matching the shape of the heel impression. Notice the deep traces of claws II and III, as well as the burrows in the impressions of digits III and IV. H) Track R3 of the trackway CP6-14. I) Two overlapping tracks. On the right is R4 of trackway CP6-30, which overlaps the right track R4 of trackway CP6-9. On the left side, note the prominent hallux impression on the lateroposterior side of the track and the backward orientation of the claw impression of its digit II. J) Right track of trackway on site CP2. Notice the numerous indentations on the surface of the substrate left by the eroded shells of small gastropods. K) Padding on a left track on site CP1. h = hallux. The scales are in cm.

**Fig 6:**
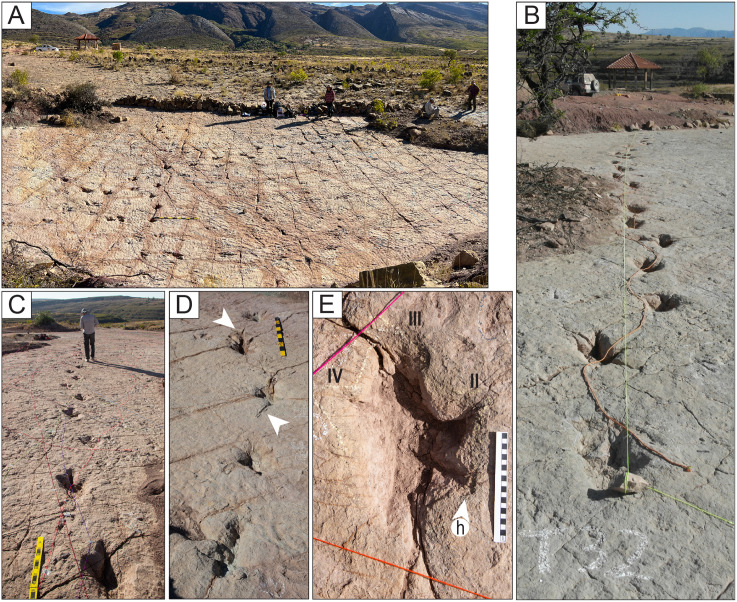
Style of preservation M5. A) Trackways with tracks of style of preservation M5 on site CP3. Notice the ripples on the surface of the layer. B) Trackway T32 has very deep tracks and tail traces. The sinuous cord marks the tail traces. C) Deep tracks of the trackway T22-2-25 D) Set of five very deep tracks of the trackway TS102. White arrowheads indicate tail traces. E) Track L10 of the trackway T22-126. The digits are marked with the numbers II, III, and IV. h = hallux. The scales in C and D are in 10 cm sections, and the scale in E is 20 cm.

**Fig 7:**
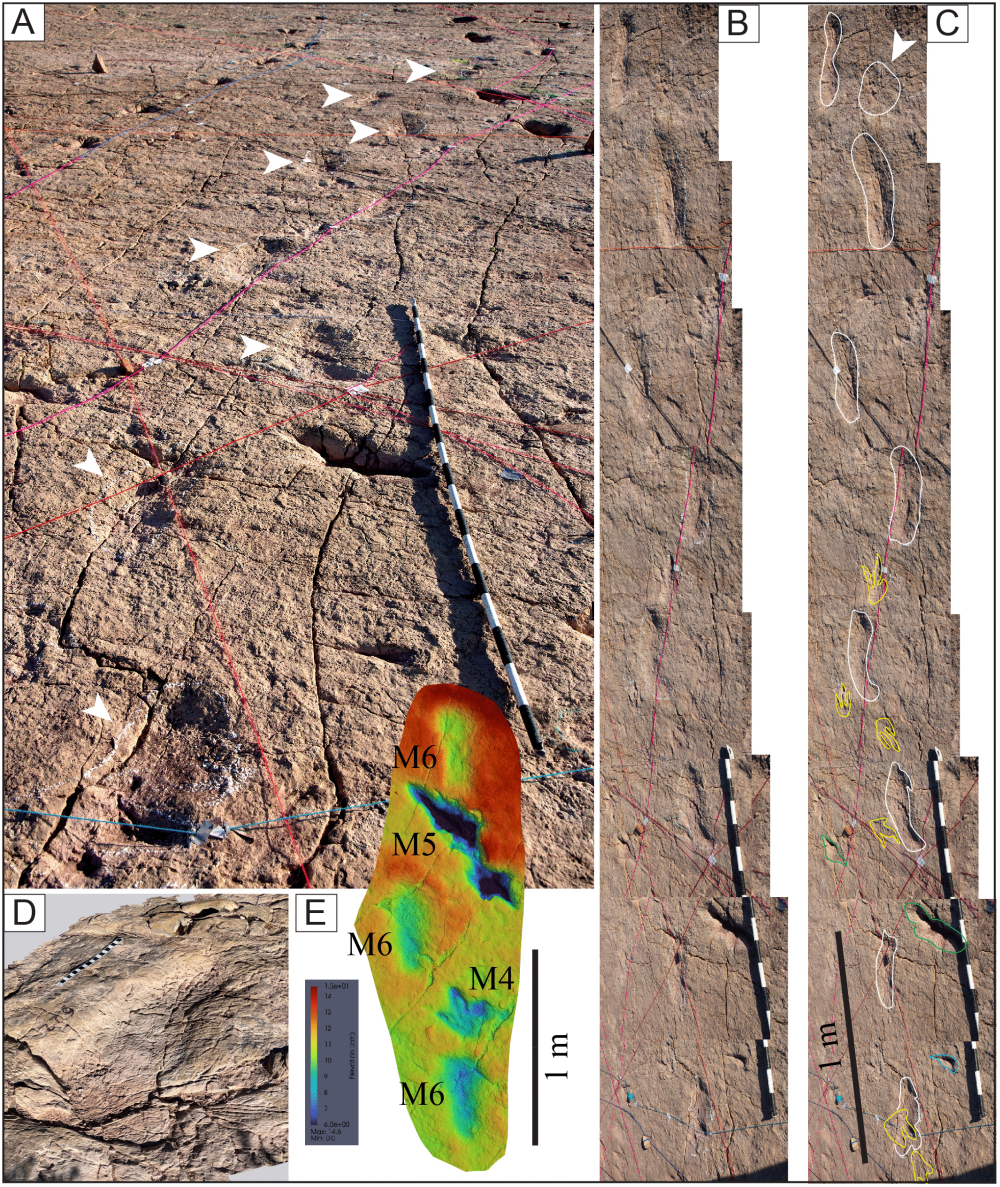
Style of preservation M6. A) View of a trackway on site CP2. The white arrows indicate the shallow tracks of the style of preservation M6. Notice the very deep track of the style of preservation M5 adjacent to the left side of the ruler and on the top right corner of the image. Also, note a trackway with tracks of the style of preservation M4 in the diagonal of the upper left corner of the picture. The scale is 2 m. B–C) The same trackway as in A, seen from above. A white arrow near the top indicates one subcircular track in another trackway. Some theropod tracks are marked in yellow lines. Deep tracks of the style of preservation M5 are marked in green. One swim trace is marked in blue. D) Deep track of the trackway T22-2-602. E) Depth map of a set of three tracks in site CP1 of the style of preservation M6, two very deep tracks of the style of preservation M5 (dark blue) and one of the style of preservation M4. The scale in D is 20 cm.

**Fig 8:**
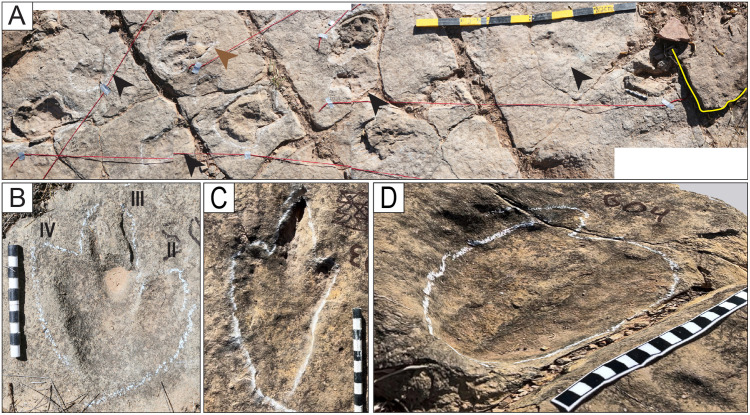
Style of preservation M7. A) Panorama of trackways TO22-2-569, TO22-2-570, and TO22-2-574, and associated tracks. These tracks are preserved in a layer 3–4 cm below the surface of the trackbearing layer at the north end of site CP2. The original trackbearing surface before erosion occurred is seen on the right side of the photo (yellow line). Some undertracks are preserved as concave epirelief (black arrowheads), and others with the sediment filling of the eroded layer that remains in the concavity of the track (white arrowheads). The track indicated with a brown arrowhead presents both types of preservation. B) Detail of a right track of the trackway TO-22-2-570. C) Detail of a track of the trackway TO22-2-574. D) Undertrack T22-2-604. Scale in A is 1 m, in B–C is 10 cm, and in D is 20 cm.

**Fig 9:**
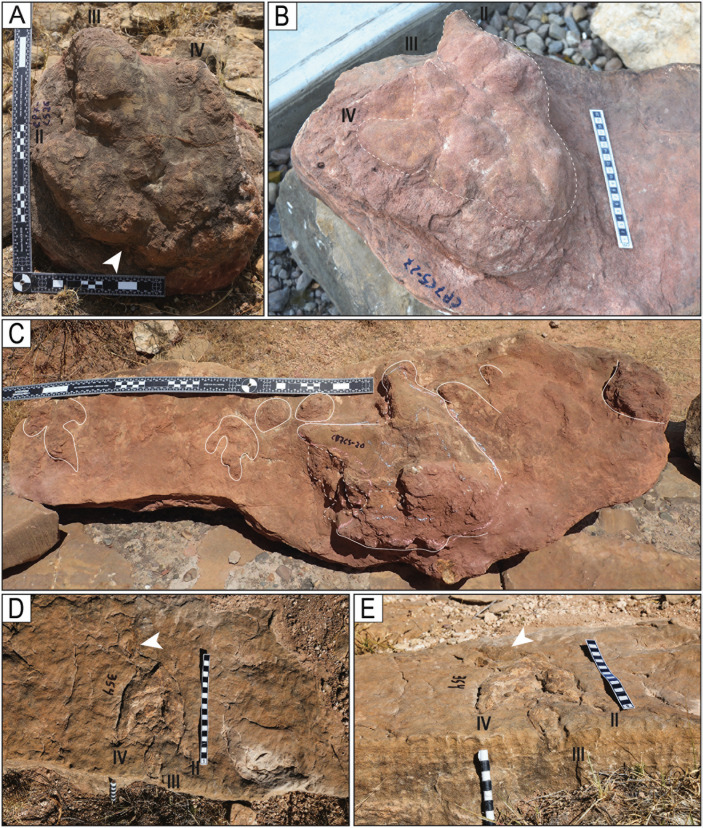
Style of preservation M8. A) Track CP7-CS26. Notice the impressions of the digital and metatarsal pads and the medial notch (white arrowhead). B) Track CP7CS-27. The impression of digit III is cut off. Notice the impressions of the digital and metatarsal pads. C) Track CP7CS-20 and other complete and incomplete tracks in convex epirelief. D–E) Track T22-2-354 from the edge of site CP8, in plain view and cross-section. Erosion of the upper layers of the unit left a natural cast of the print and the deformation of an undertrack to the right of the ruler. Notice the enigmatic curved posterior extension (white arrowhead). The scales are in cm.

**Fig 10:**
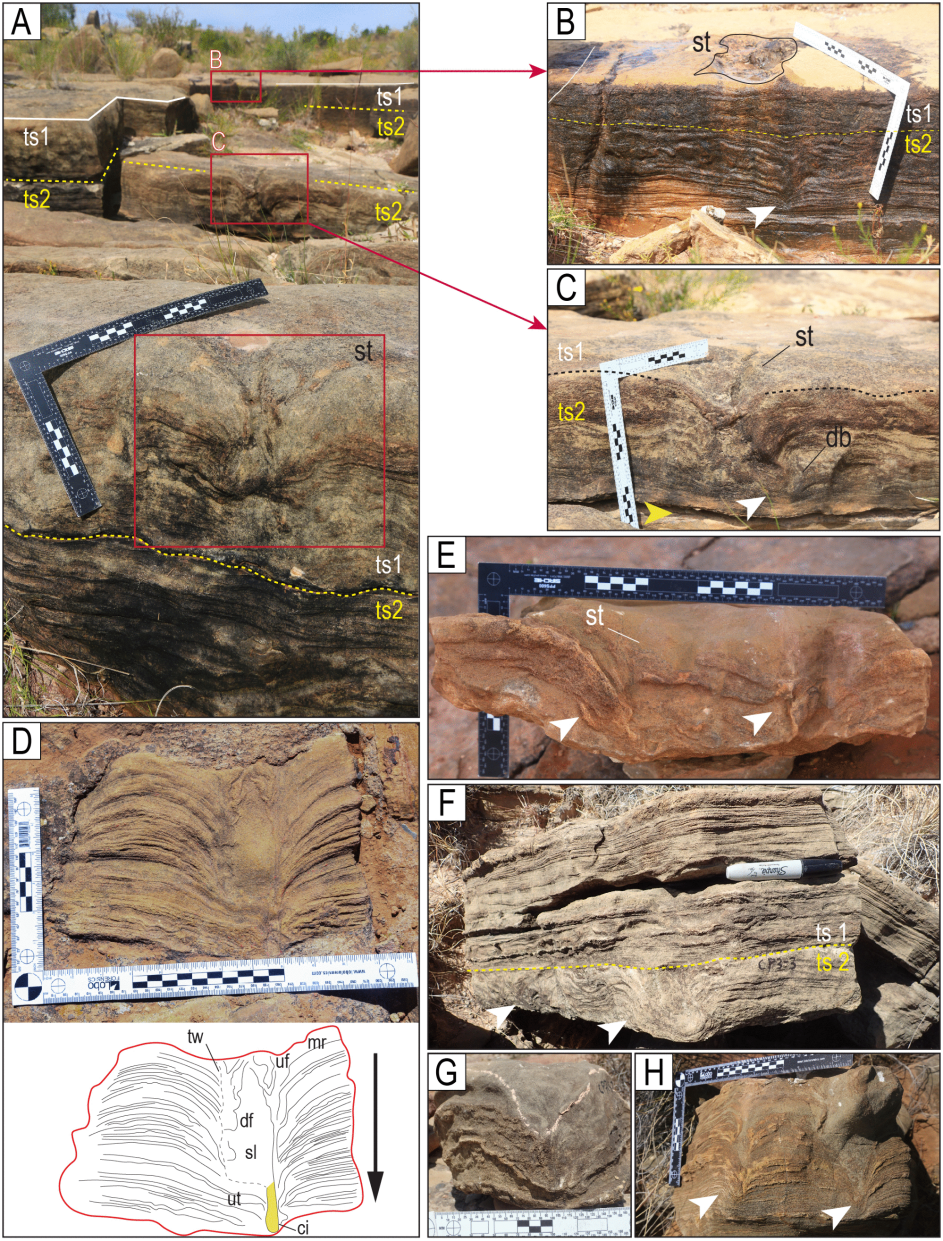
Tracks in cross-section. A–C) Theropod tracks in cross-section along the northern edge of site CP2. Here, the vertical exposure shows two levels (or surfaces) of tracks (ts1 and ts2). Track level ts1 is the level of the tracks on the tracking surface of Carreras Pampa. The evidence for tracks on ts2 is in the cross-section. The shallow, elongated depressions without morphological details on the surface of Carreras Pampa described as mode of preservation M6 (Fig 9) would correspond to the tracks preserved in ts2, which were covered by later sediment. The displacement bulb (db) was likely produced during the lateral movement of the tow or claw inside the soft substrate. D) A block used in a sidewalk shows a cross-section of a print. The black arrow indicates the downward movement of the toe into the soft substrate. The folds (df, uf) represent the liquefaction of the sediment in the lower and upper halves of the trace, likely produced by the downward and upward movement, respectively, of the toe when the autopodium was withdrawn from the substrate. E) Block of rock showing the sediment deformation produced by two of the toes. F) Deformation of possibly two different tracks in the ts2 sublayer. A yellow dotted line marks the boundary between sublayers ts2 and ts1. Notice the planar and low-angle lamination in the ts1 sublayer and the undeformed area of the ts2 sublayer. G) Track CP3−2. H) Track CP9CS-1. ci = undertrack formed by the claw impression (yellow), mr = marginal ridge, sl = structureless sediment, st = track on the surface, tw = track wall, ut = undertrack. The scales are in cm.

**Fig 11:**
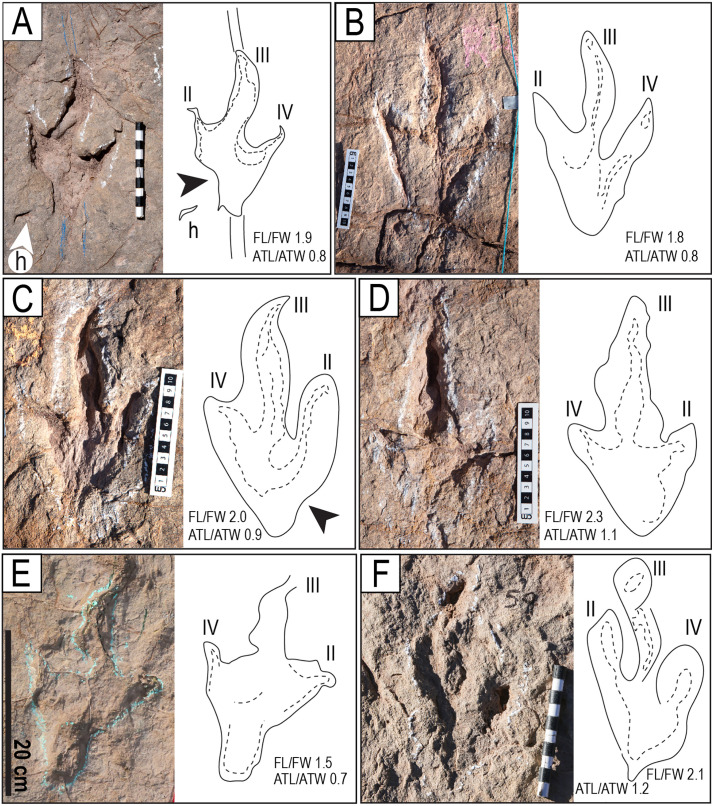
Morphotypes T1 and T2 A–D) Morphotype T1. A) Track R3 of the trackway T22-3. Notice the hallux impression (h) and the linear anterior and posterior grooves (parallel black lines). B) Track L3 of the trackway T22-2-8. C) Track R4 of the trackway T22-2-46. D) Left track of the trackway TO22-2-47. E–F) Morphotype T2. E) Track L2 of the trackway CP9-59. F) Right track of the trackway T22-2-59. The scales are in cm.

**Fig 12:**
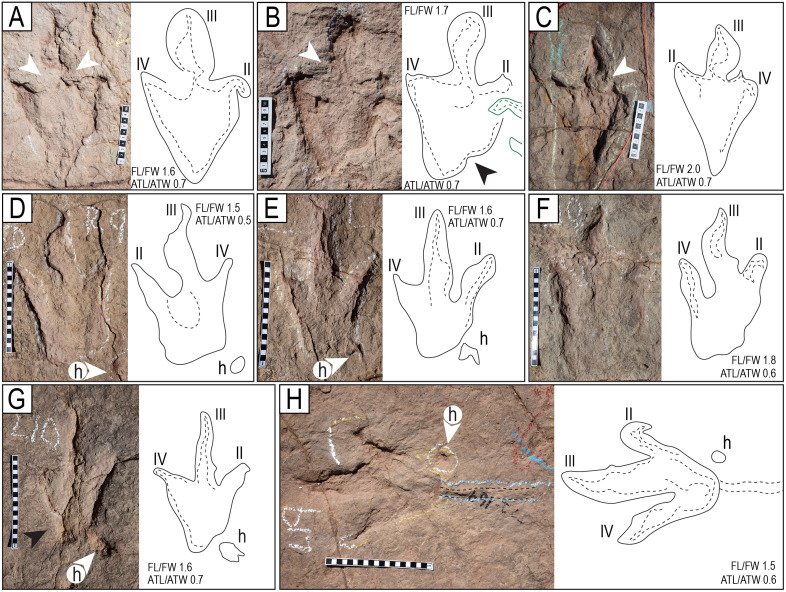
Morphotypes T3 and T4. A–C) Morphotype T3. A) Track L3 of the trackway T22-2-8. B) Left track of the trackway T22-2-21. C) Right track of the trackway T22-2-49. D–H) Morphotype T4. D) Track R9 of the trackway T22-56. E) Track L6 of the trackway T22-79. F) Track L10 of the trackway T22-56. G) Track L10 of the trackway T22-79; notch indicated with black arrowhead. H) Track L19 of the trackway T22-72. Notice the straight ridge of sediment between the two black dotted lines, extending from the edge of the heel impression. The scales are in cm.

**Fig 13:**
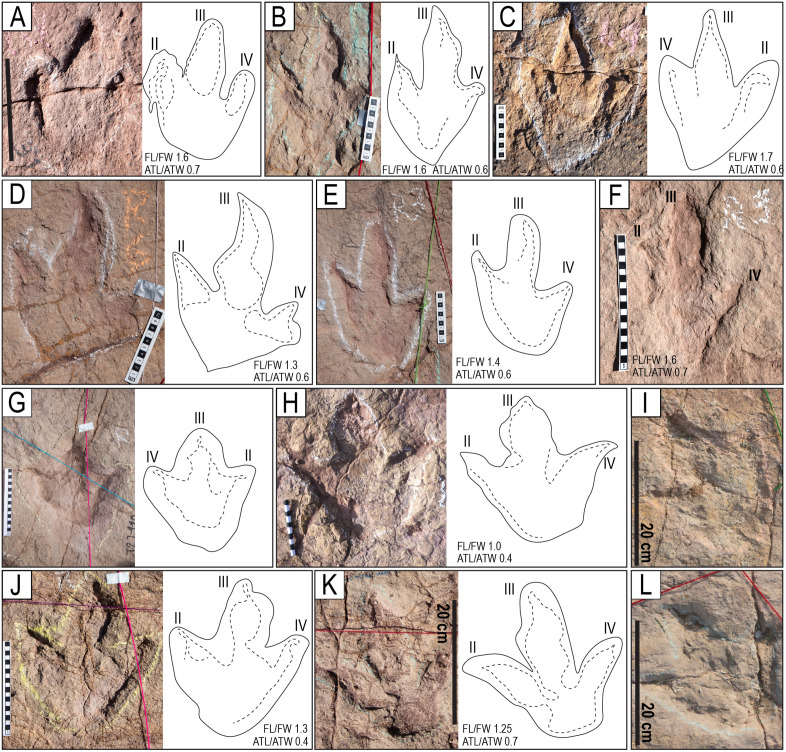
Morphotypes T5 and T6. A–F) Morphotype T5. A) Track R1 of the trackway T22-2-31. Scale bar is 20 cm. B) Track R4 of the trackway T22-2-49. C) Track of the trackway T22-2-46. D) Track R6 of the trackway T22-2-51. E) Track R3 of the trackway T22-2-54. F) Track R5 of the trackway T22-72. G–I) Morphotype T6. G) Track R17 of the trackway T22-126. H) Right track of the trackway CPT-117. I) Track L1 of the trackway CP9-53. J) Track R3 of the trackway T22-126. K) Right track of the trackway T22-121. L) Track R2 of the trackway CP9-53. The scales are in cm.

**Fig 14:**
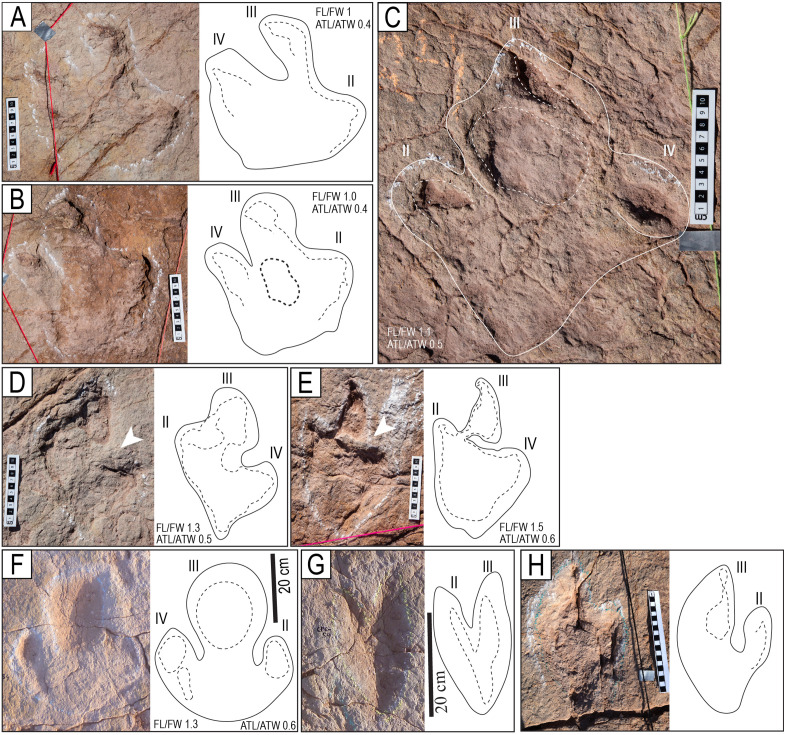
Morphotypes T7–T10. A–C) Morphotype T7. A) L1 track of the trackway T22-2-40. B) Track L2 of the trackway T22-2-40. C) Track R4 of the trackway T22-2-36. Scales in cm. D–E) Morphotype T8. D) Track of trackway T22-2-21. E) Track of trackway T22-2-45. F) Morphotype T9. Left track of trackway CP6-54. G–H) Morphotype T10. G) Right track of trackway CP6-54. H) Track of trackway T22-141. The scales are in cm.

**Fig 15:**
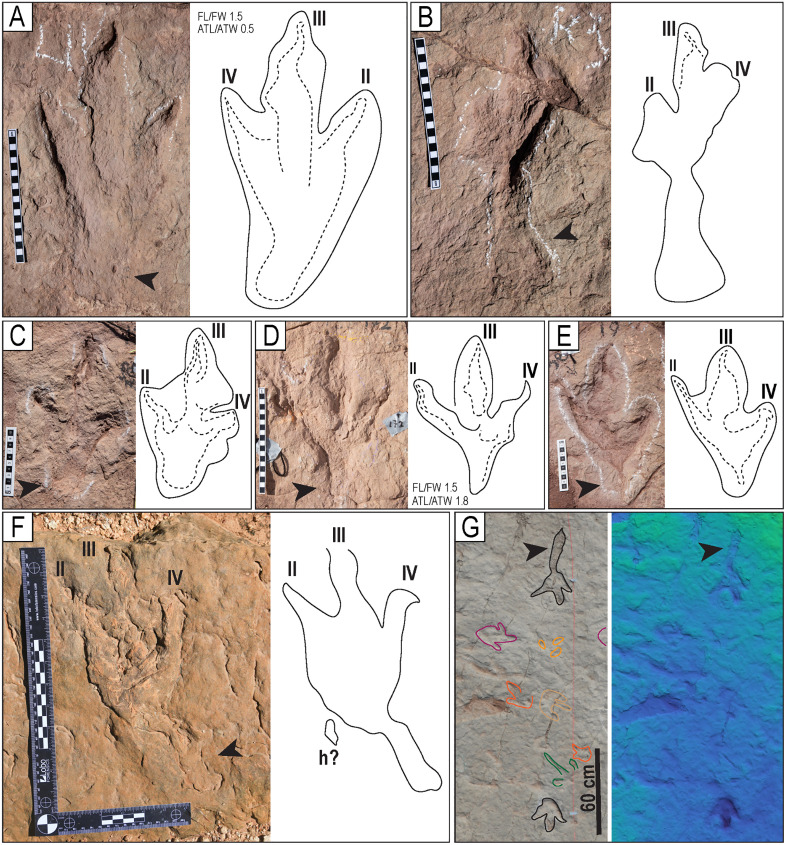
Morphotype T11. A) Track L11 of the trackway T22-56. B) Track R3 of the trackway T22-60. C) Right track of the trackway T22-2-19. D) Right track of the trackway T22-172. E) Track R1 of the trackway T22-2-58. F) Track T22-2-354, showing an enigmatic sinuous posterior extension. G) Partial view of a trackway in site CP7. The black arrowheads indicate the posterior extensions. The scales are in cm.

**Fig 16:**
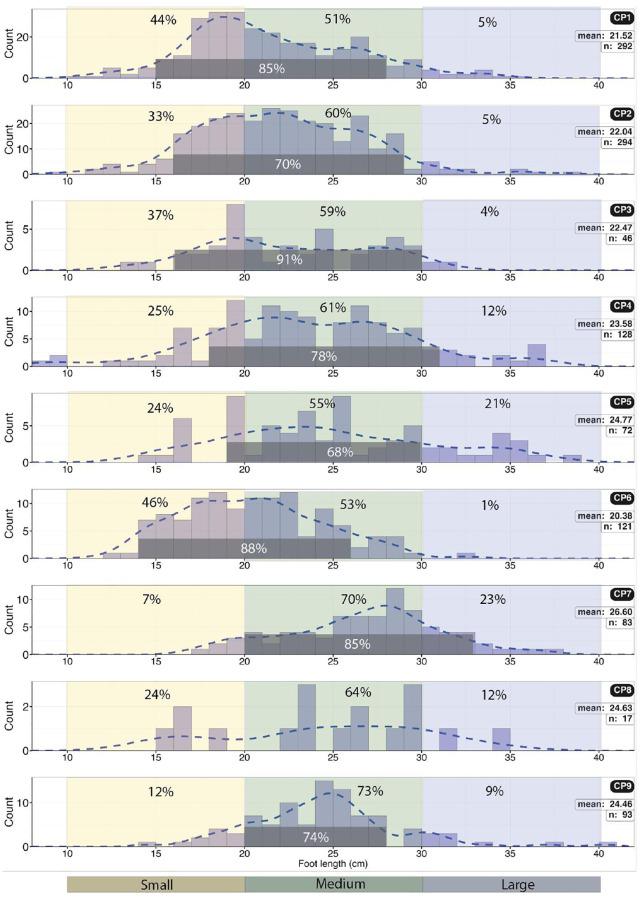
Histograms of foot length by size category (small, medium, or large) with percentage per category at each site. The dominant size is medium at all sites. The dominant range is also highlighted. CP8 has very little data. Combined data are not normally distributed and may be multi-modal.

**Fig 17:**
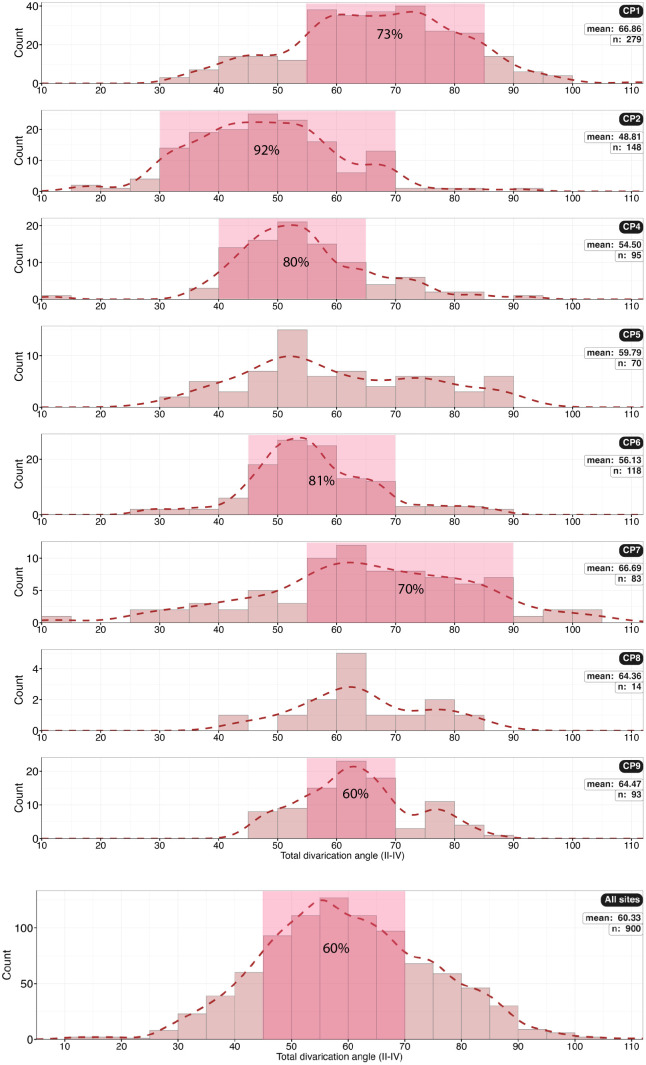
Histogram of total divarication angle (αII–IV) for all sites. The distribution of angles for all sites combined (n = 900) is normally distributed.

**Fig 18:**
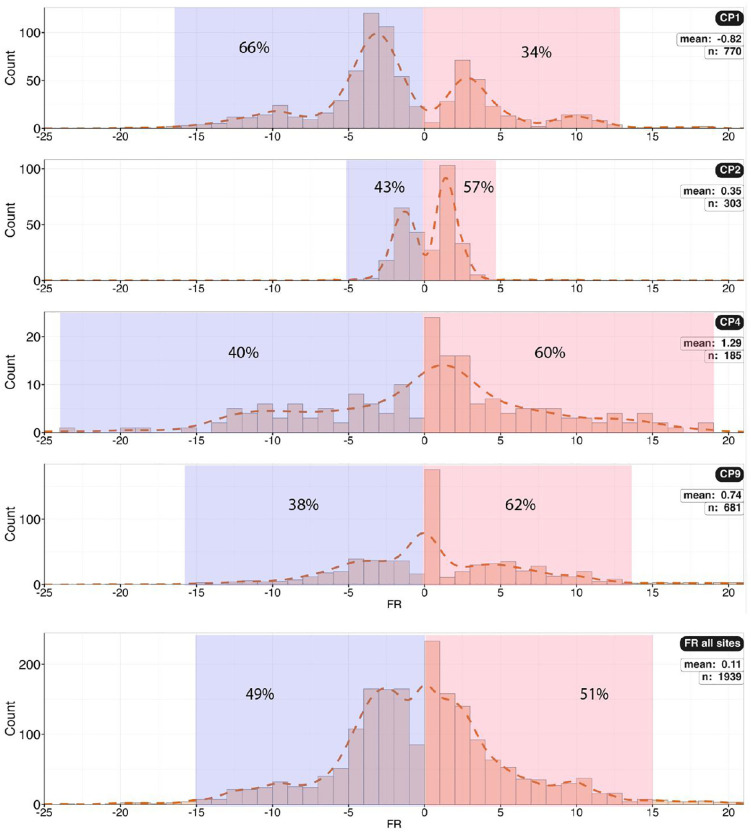
Histogram of foot rotation for sites CP1, CP2, CP4, and CP9, and the aggregated for those sites, with positive (outward) and negative (inward) values highlighted. A bimodal distribution is notable.

**Fig 19:**
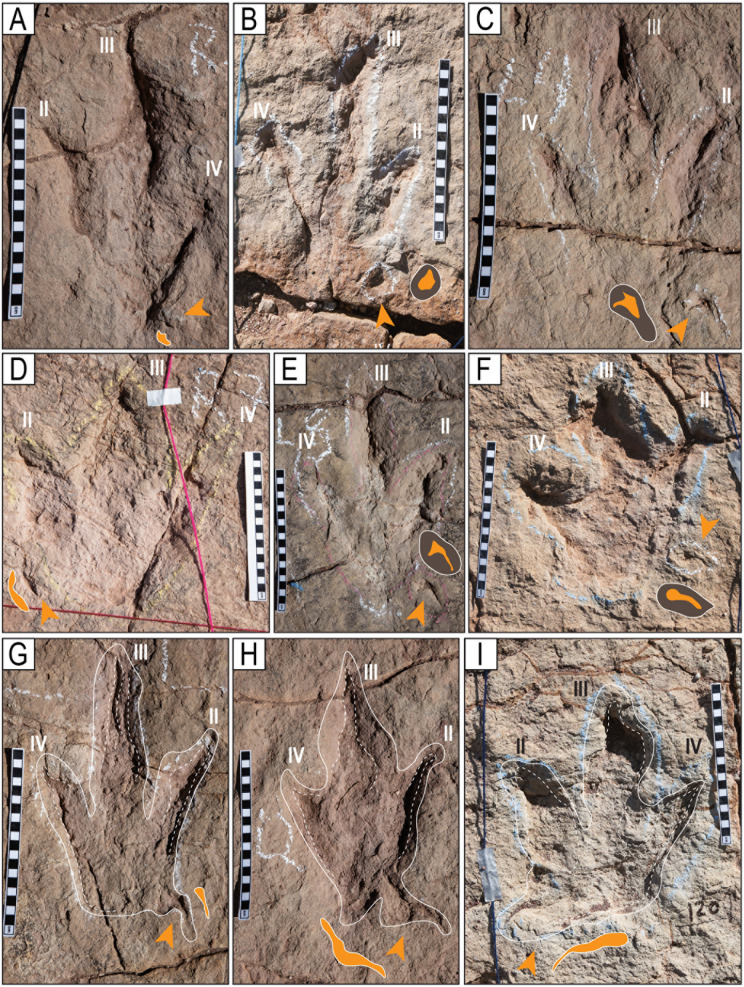
Preservation of the hallux with three different morphotypes. The orange arrowhead indicates hallux. The adjacent drawings indicate the morphology of the halluces (in orange color) and their expulsion rims (in brown color). A–C). Hallux morphotype H1. A) Track R2 of the trackway T22-72. B) Left track of the trackway T22-2-96. C) Track L4 of the trackway T22-2-54. D–F) Hallux morphotype H2. D) Track R7 of the trackway T22-126. E) Track L8 of the trackway T22-79. F) A left track of the trackway T22-2-120. G–I) Hallux morphotype H3. G) Track L6 of the trackway T22-79. H) Track L2 of the trackway T22-79. I) Right track of the trackway T22-2-120. The scales are in cm.

**Fig 20:**
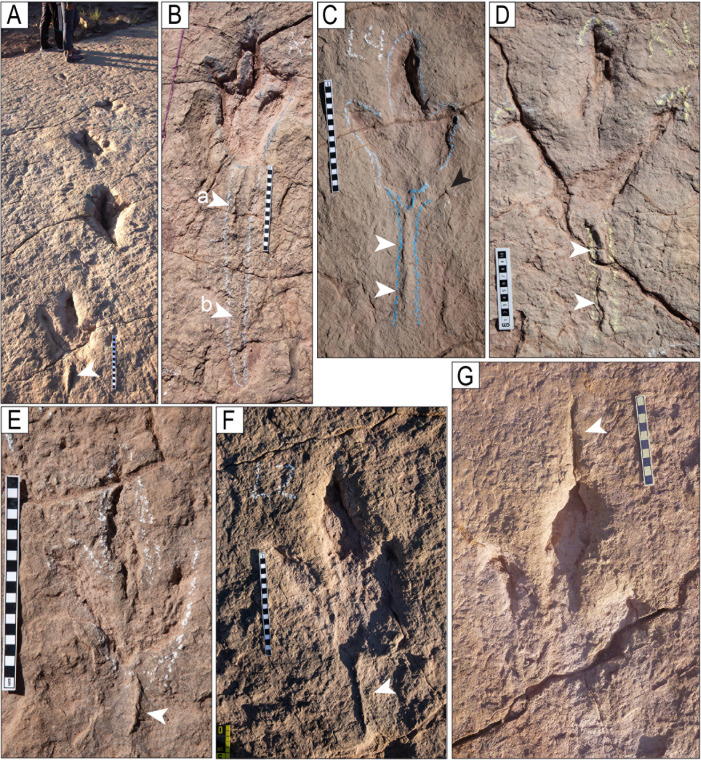
Tracks with posterior ridges and anterior grooves. White arrowheads indicate the ridges. A) Partial view of trackway T22-102, which consists of some very deep tracks and some deep and shallow tracks toward the people standing. The first track in the foreground has a short ridge of sediment. B) Track R8 of the trackway T22-121. C) Track L4 of the trackway T22-72. Notice the mark of the hallux (black arrow). D) Track R1 of the trackway T2-2-17. E) Track of the trackway T22-93. F) Track L2 of the trackway T22-78. G) Track of the trackway CP7-U7 showing a linear groove in the front of digit III. The scales are in cm.

**Fig 21:**
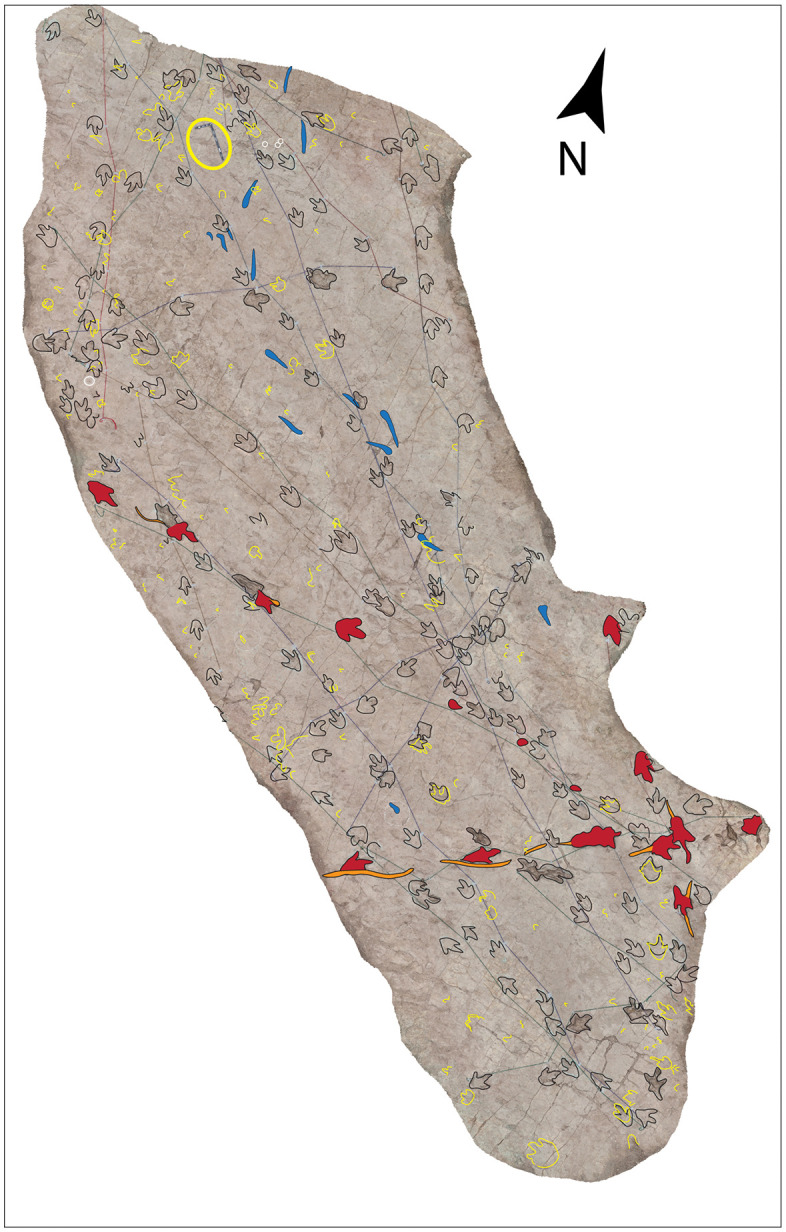
Image of sector 1 of site CP9 with a high density of tracks and the presence of tail traces, swim tracks, and avian tracks. A) Notice multiple trackways in parallel or semi-parallel orientation. The swim tracks are in dark blue. Three trackways with tail traces are marked by red color. A few bird tracks are outlined in white circles. The yellow ellipse indicates a 35-cm-long ruler. The tracks and partial tracks in yellow were noticed in the depth map but not in the field. B) Depth map of the same area.

**Fig 22:**
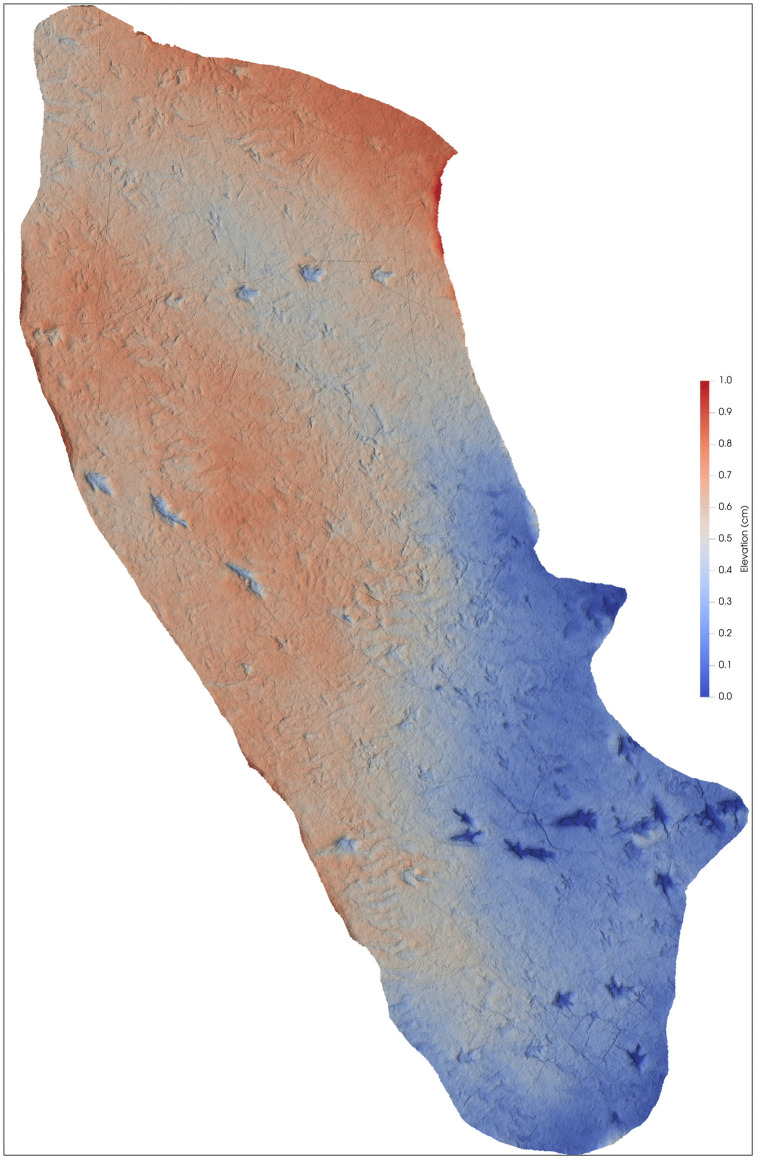
Color depth map of the CP9 area illustrated in Fig 21.

**Fig 23:**
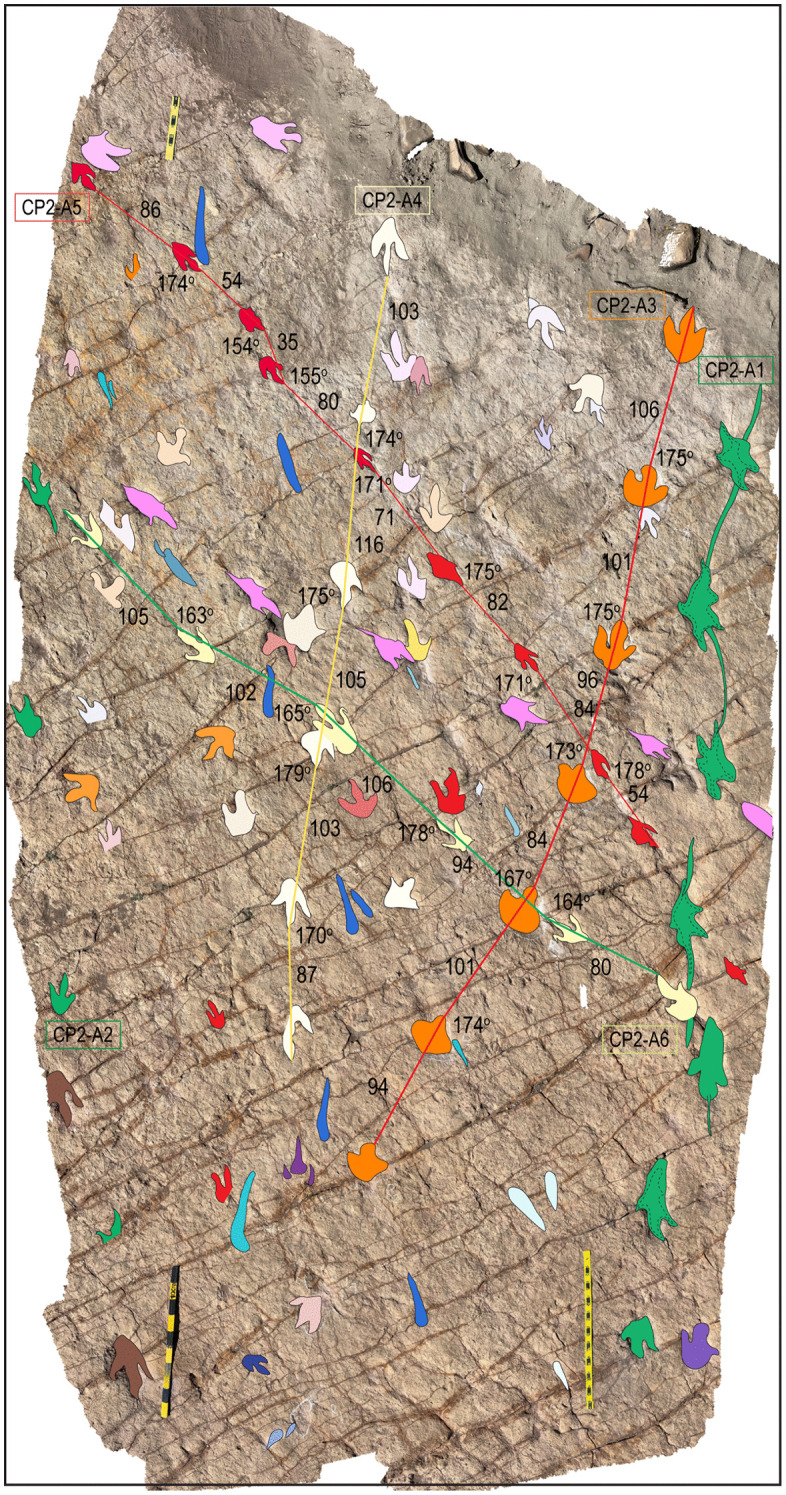
An area of site CP2 with trackways, tail traces, and swim tracks. Notice multiple parallel trackways. Trackway CP2-A1 in green color shows tail traces. Swim tracks are indicated in different shades of blue, with dark blue indicating a swim trackway consisting of six continuous, alternative right and left swim tracks. The numbers indicate the pace length and pace angulation (PANG) of two trackways in opposite directions NWN/NEN, and two trackways in opposite directions E–W. The two scales at the bottom of the image are 1 m each.

**Fig 24:**
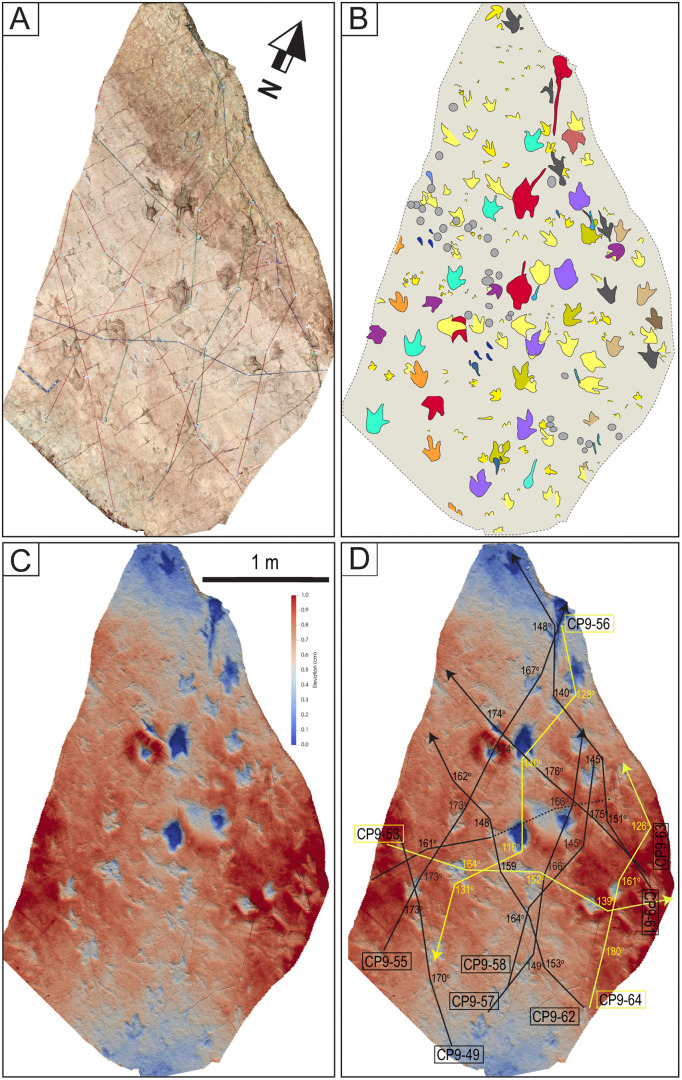
An area in site CP9 with multiple trackways, isolated tracks, swim tracks, tail traces, and bird tracks. A) 3D model of the area. B) Outlines of tracks. Tracks with associated tail traces are red. Swim traces are represented in different shades of blue. C–D) Depth map of the area. D) PANG values of selected trackways. Notice the narrow trackways CP9-49, CP9-57, CP9-61, CP9-63, and CP9-64. The latter shows a sudden change in direction toward the NW with a PANG of 126^o^. The black ruler on the left of A is 35 cm long.

**Fig 25:**
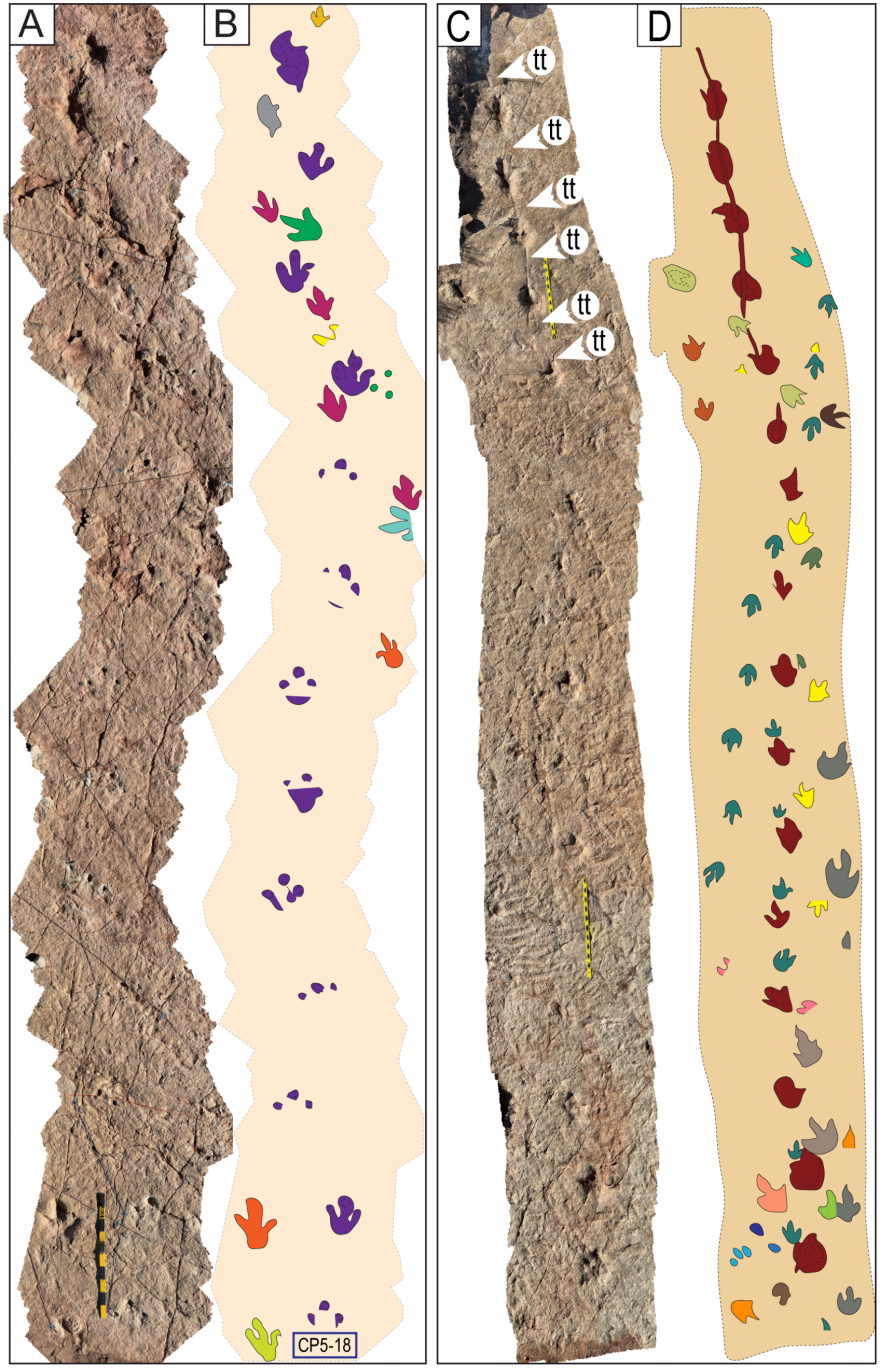
Narrow-gauge trackways. A–B) The purple trackway CP5-18 has tracks of the style of preservation M1. Purple and red trackways are narrow-gauge. C–D) Several trackways of narrow-gauge gait. tt = tail traces in the red trackway. Notice the continuous tail trace that extends over five tracks at the top of the trackway. Scales are 1 m.

**Fig 26:**
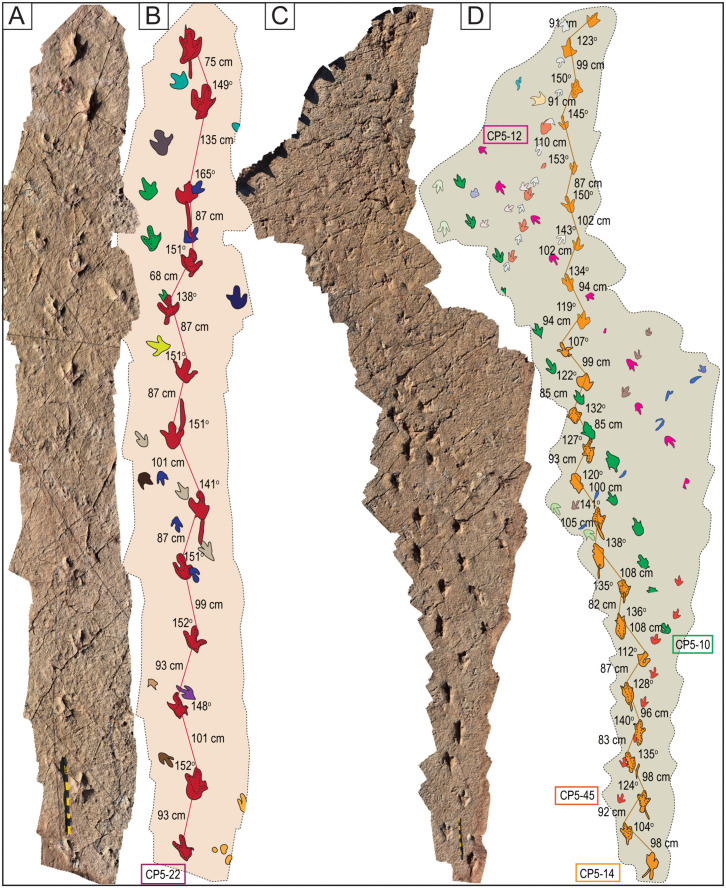
Wide-gauge trackways in site CP5. A–B) Notice trackway CP5-22 with trail traces and highly variable pace length. C–D) Notice trackway CP5-14 with highly variable pace length and pace angulation. Swim tracks are depicted in shades of blue. Yellow and black scales are 1 m.

**Fig 27:**
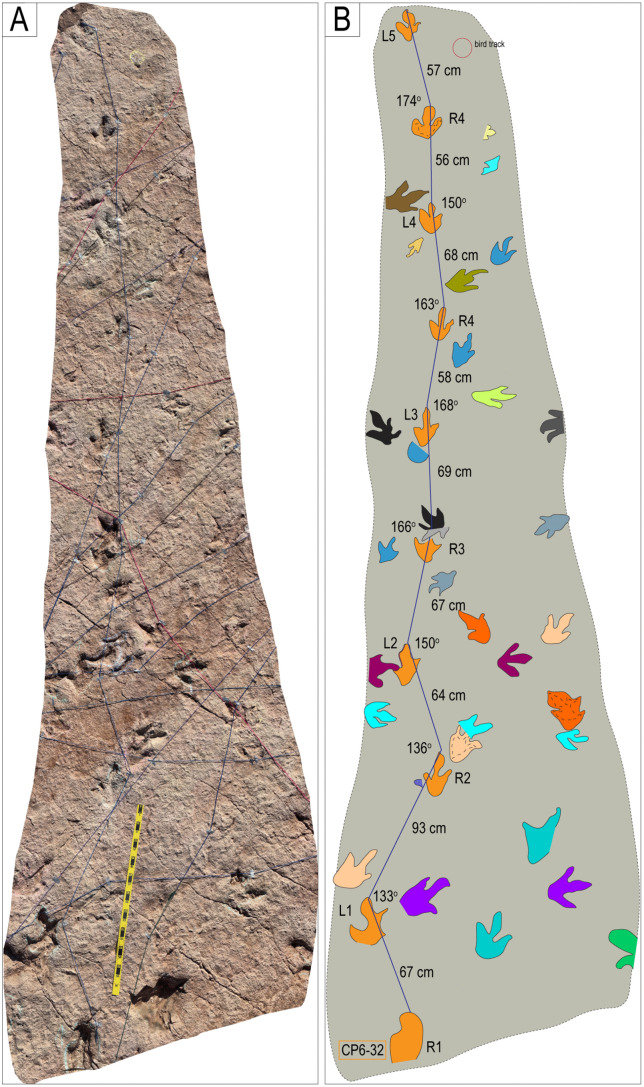
Trackway CP6-32 and associated tracks. Notice the progression from wide gauge in tracks R1–L2 to narrow gauge in the rest of the trackway (L2–L5). The scale is 1 m.

**Fig 28:**
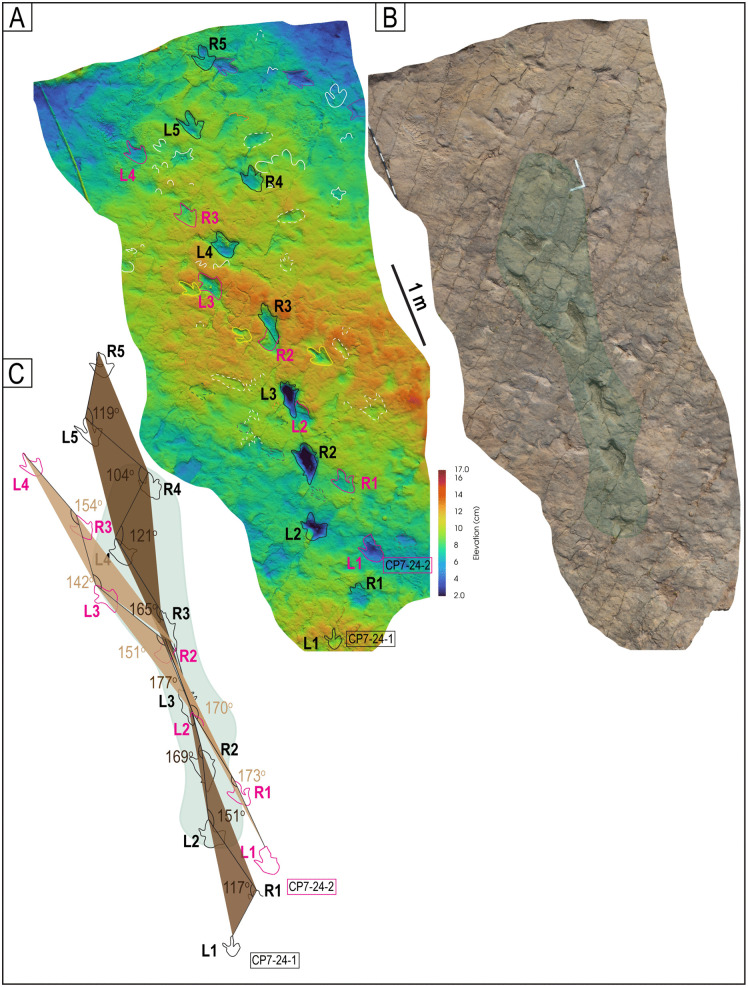
Trackways CP7-24-1 and CP7-24-2 and associated tracks. A) Depth map of the area with the two trackways marked by black and red labels. B) Image from a 3D model of the same area. C) PANG values of the two trackways.

**Fig 29:**
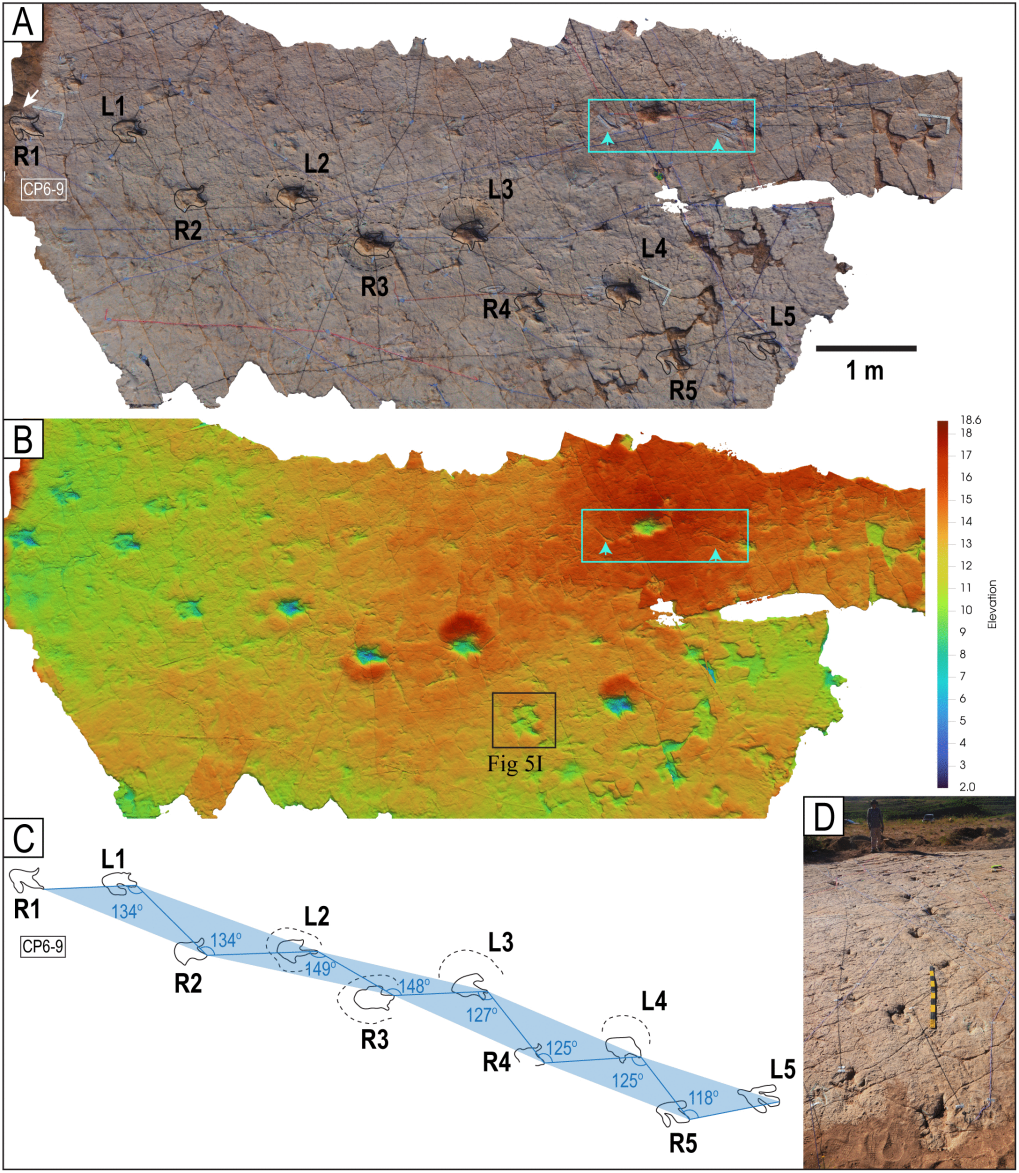
Trackway CP6-9 in site CP6. A) Orthogonal image from a 3D composite model of the area, including the trackway and associated tracks. B) Depth map of the same area. Not all tracks are outlined. The blue arrows indicate swim traces within the blue box. Other swim traces occur in the area. Notice the prominent displacement rims of tracks L2, R3, L3, and L4. C) Outline of the exposed trackway showing the low angles of the broad gauge. D) View of the trackway from the south.

**Fig 30:**
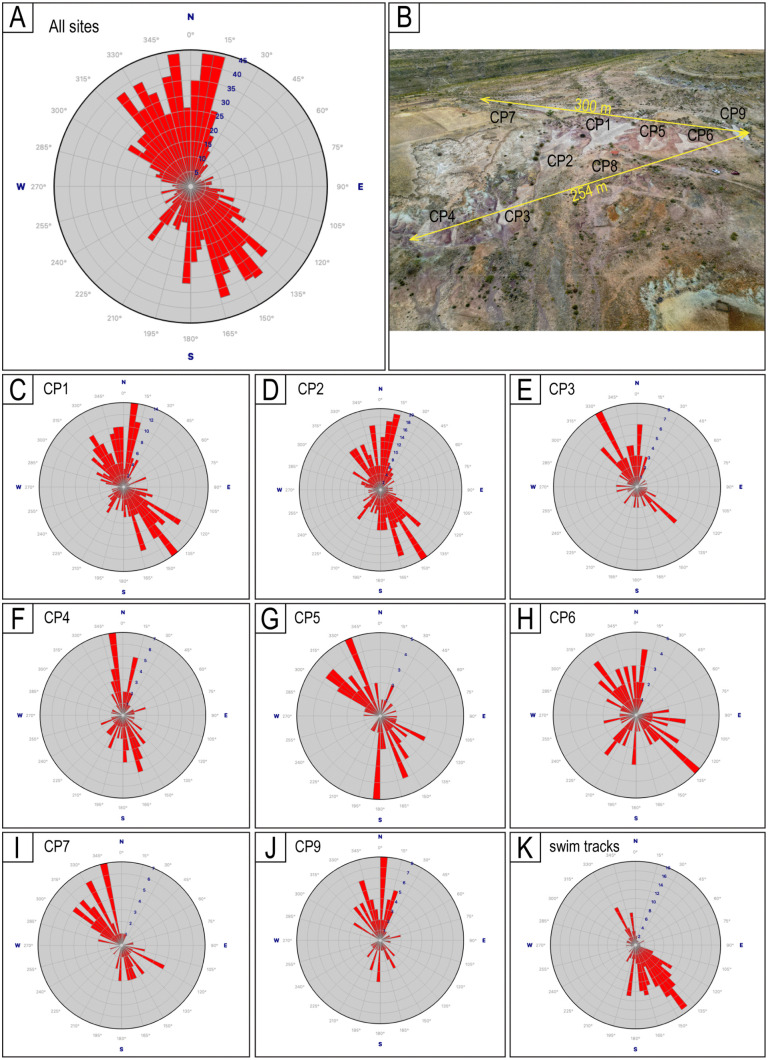
Rose diagrams with the orientation of the trackways (2 + tracks) and swim trackways. A) Orientation of all the trackways combined from all the sites. B) Distribution of the studied sites. The two lines indicate the distance between sites CP7 and CP9 and CP4 and CP9. C − J) Orientation of the trackways for each site, except for CP8. K) Orientation of the swim trackways. The quantity of orientation measurements for each is in Table 2.

**Fig 31:**
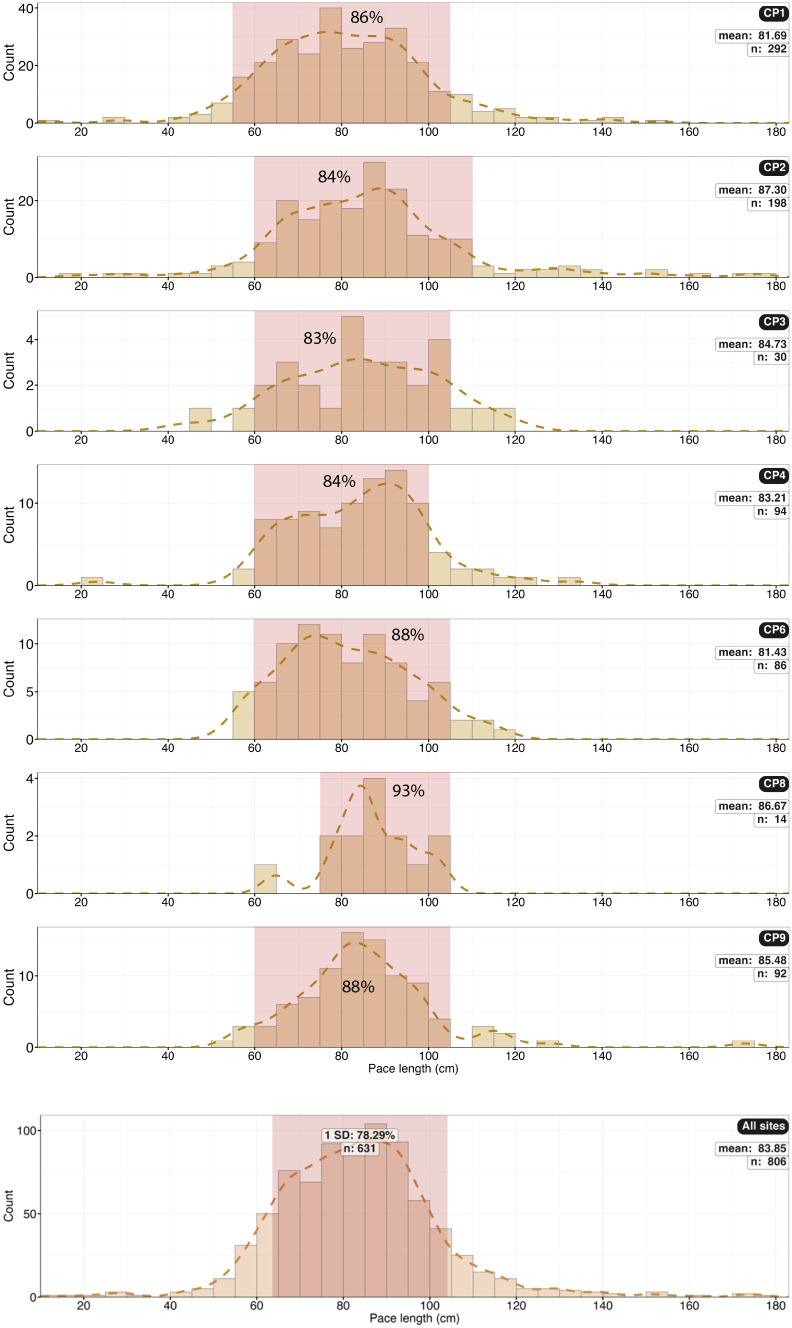
Histograms of pace length (PL) in cm for each site. CP8 has very little data. The histogram for all sites suggests multi-modal pace length distributions.

**Fig 32:**
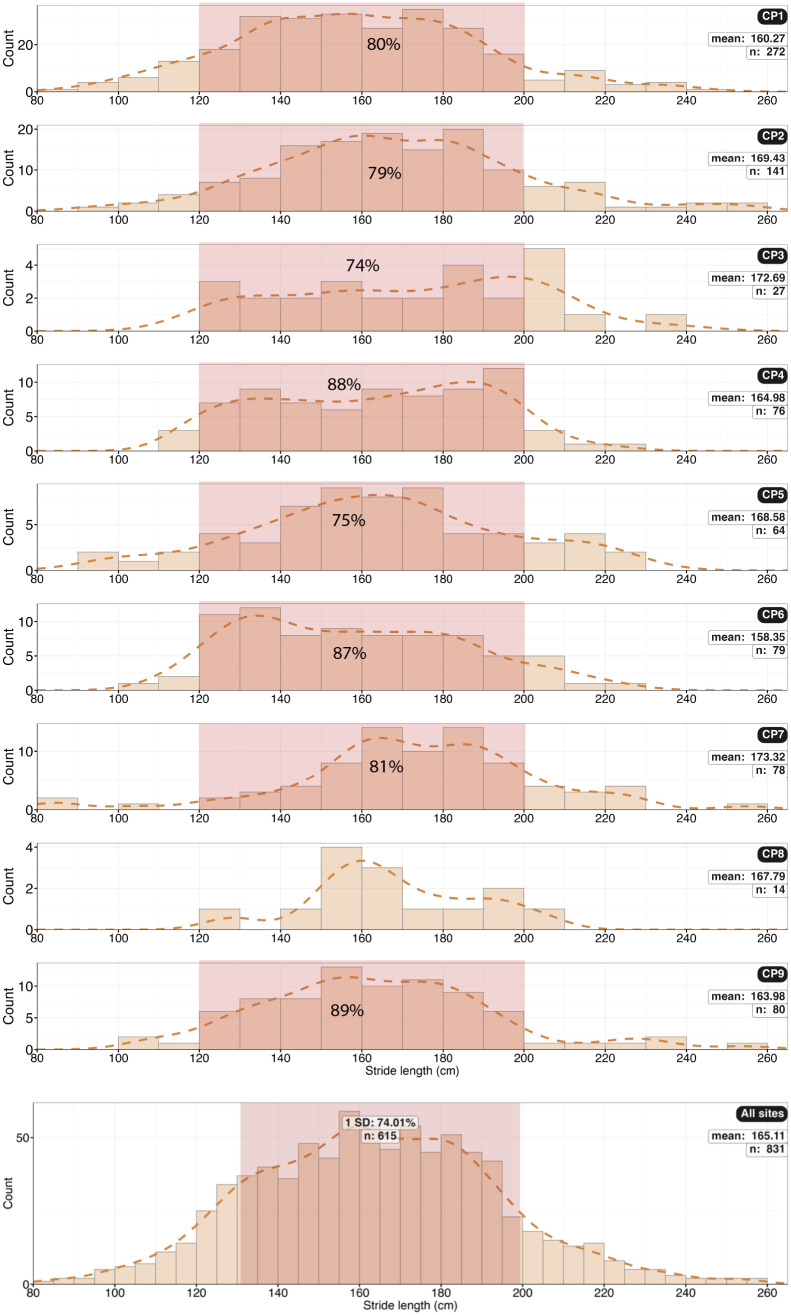
Histograms of stride length (SL). Combined data (n = 831) may show multiple modes of stride length.

**Fig 33:**
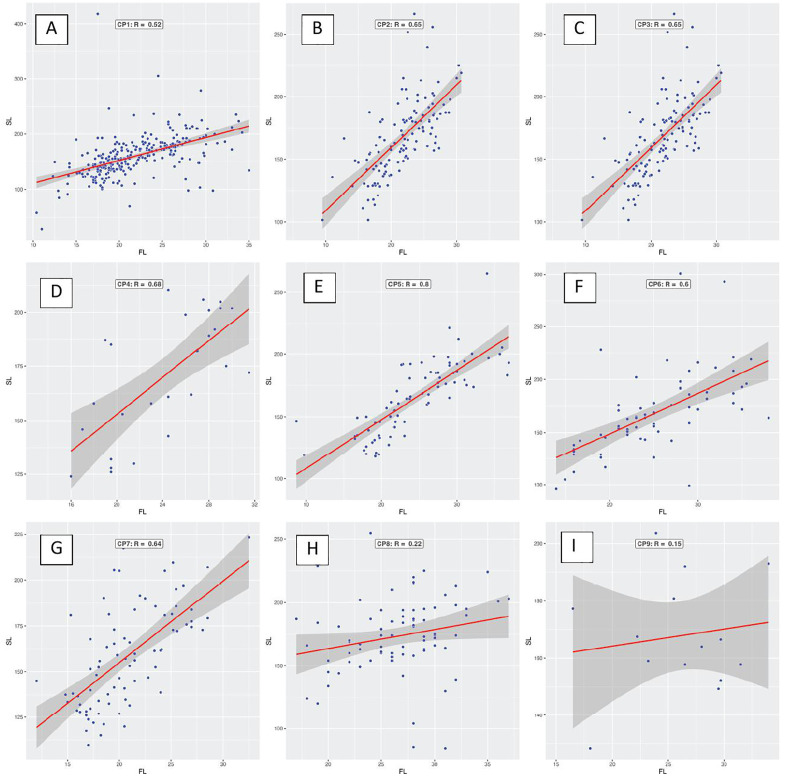
Scatterplots show a positive correlation between foot length (FL) and stride length (SL) for each site. Sites CP1, CP2, CP3, CP4, CP5, CP6, and CP9 (A, B, C, D, E, F, and I) correlate relatively strongly with R between 0.51 and 0.8, while CP7 (G) and CP8 (H) show relatively weak correlations. CP8 has a small number of samples, making its results statistically insignificant.

**Fig 34:**
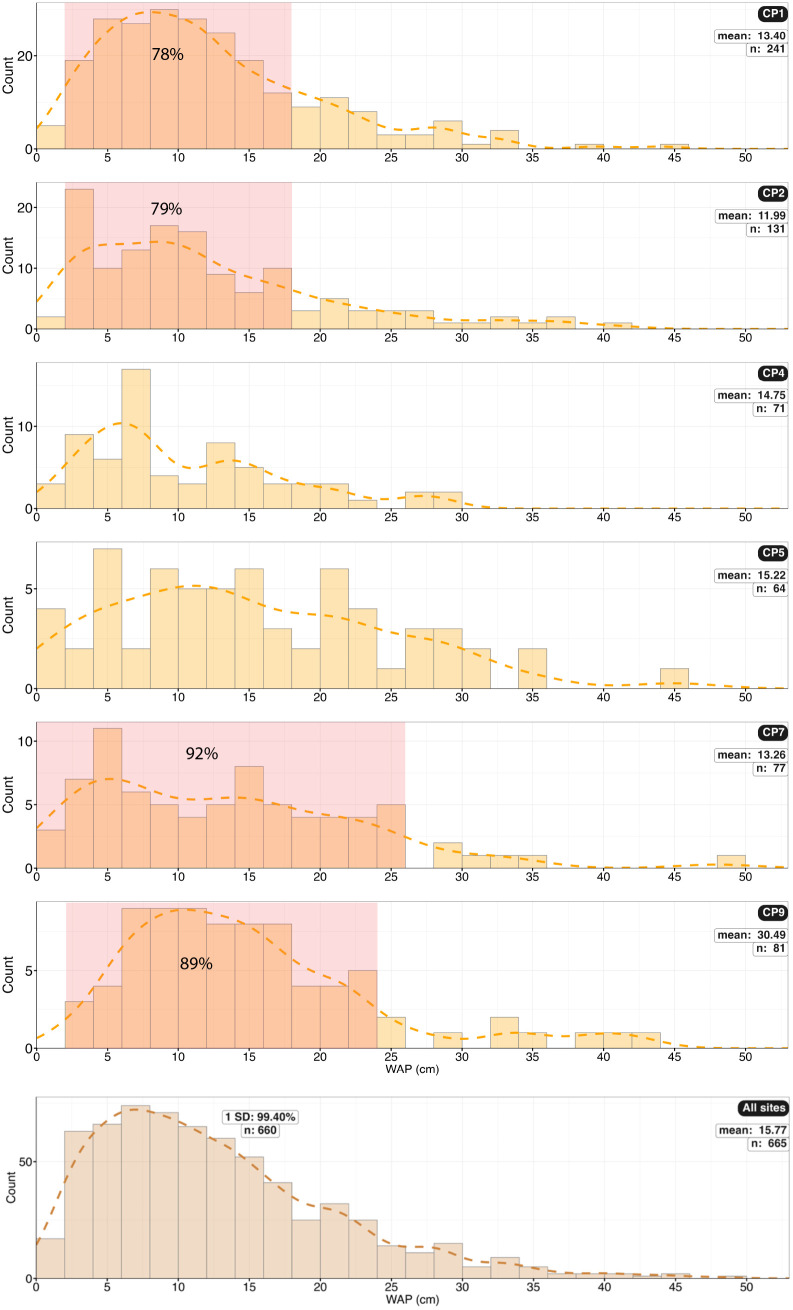
Histograms of the number of trackways in each trackway width (WAP) category. The first histogram shows the combined values for all sites. Most of the trackways have a narrow-gauge gait in the range of 5 to 10 cm, with a second group from 18 to 26 cm, and a minor number ranging from 26 to 50 cm. WAP distributions are also presented for each CP site.

**Fig 35:**
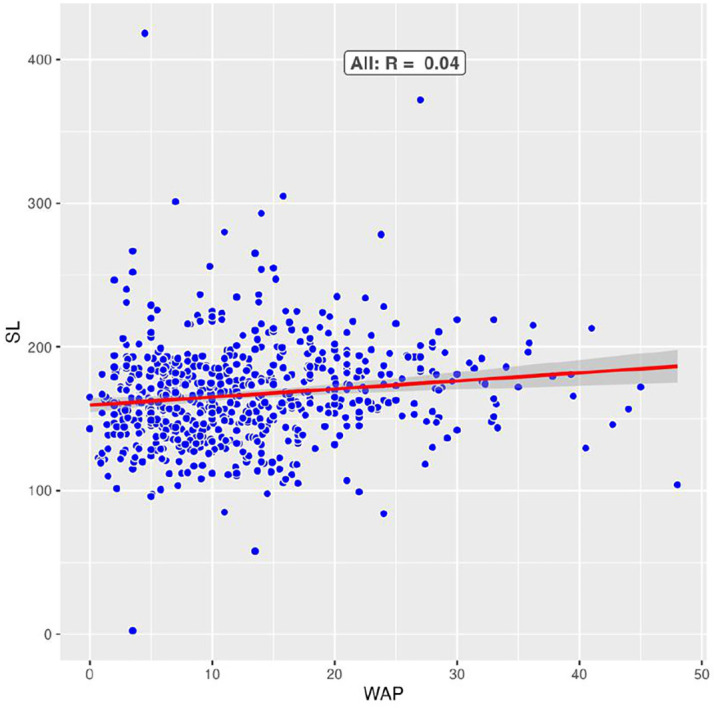
The scatterplot shows a low correlation between WAP and SL. Data is from all CP sites.

**Fig 36:**
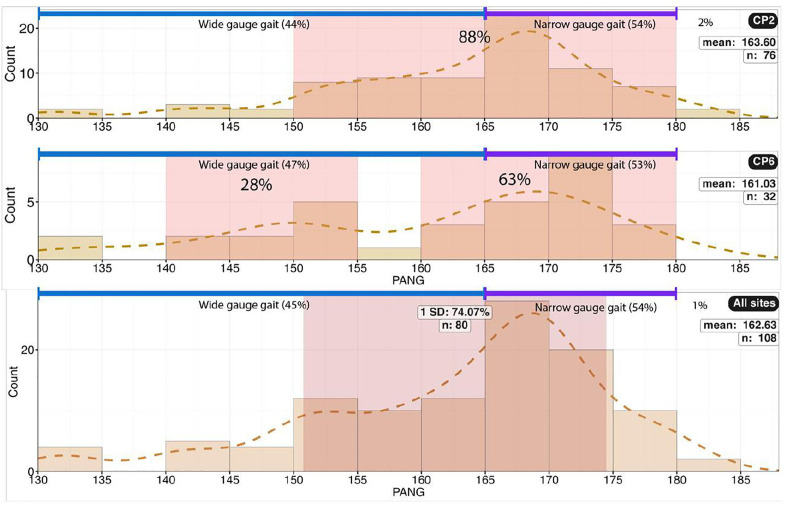
A) Histogram for trackway pace angulation (PANG) for site CP2. B) Histogram for trackway pace angulation (PANG) for CP6. For CP2, most trackways are between 150^°^ and 180°, while for CP6, two groupings are found, 140^°^ − 155^°^ and 160° − 180°. C) Histogram of PANG for both sites, showing a slight predominance of narrow-gauge gait (PANG 165°–180°).

**Fig 37:**
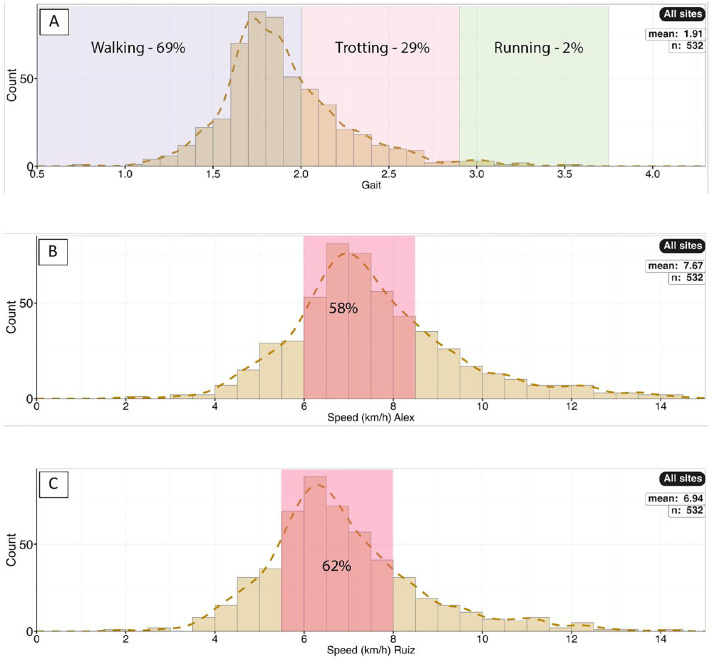
Gait and speed. A) Histogram of relative stride lengths (SL*/h*) for all sites showing proportion corresponding to SL/*h* ratios of <2.0, 2.0 − 2.9, and >2.9. We calculated the relative stride length for all trackways with two or more measured stride lengths, which totaled 532 trackways. B–C) Histograms of speed. Histogram B shows the estimated speeds as calculated using the formula of Alexander [51]. Histogram C shows the estimated speeds as calculated from the formula of Ruiz and Torices [54].

**Fig 38:**
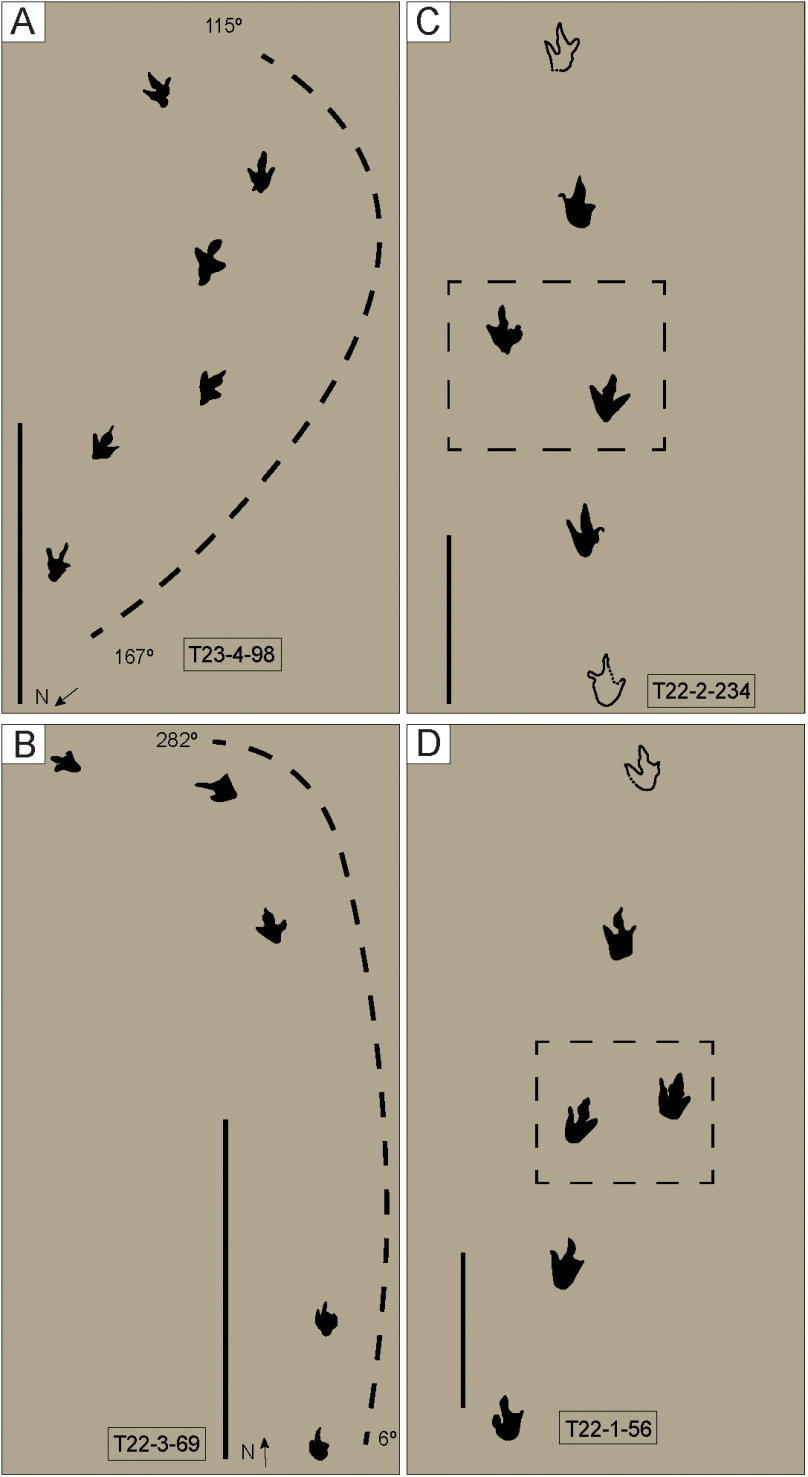
Examples of trackways with turns and stopping or pausing points. A) Section of trackway making a turn to the left of 52º. This trackway makes multiple turns along its exposure. The section shown here represents the turn with the most significant change in orientation. The average track length was 27.2 cm. The scale bar is 2 m. B) Section of trackway showing a turn to the left of 84°. The average track length was 7.7 cm. The scale bar is 1 m. C) Section of the trackway showing a stopping or pausing point (dashed box). The average track length was 30.7 cm. The scale bar is 1 m. D) Section of the trackway showing a stopping or pausing point (dashed box). The average track length was 26.2 cm. The scale bar is 1 m. Tracks not filled in represent tracks that did not have their complete outline preserved in the field.

**Fig 39:**
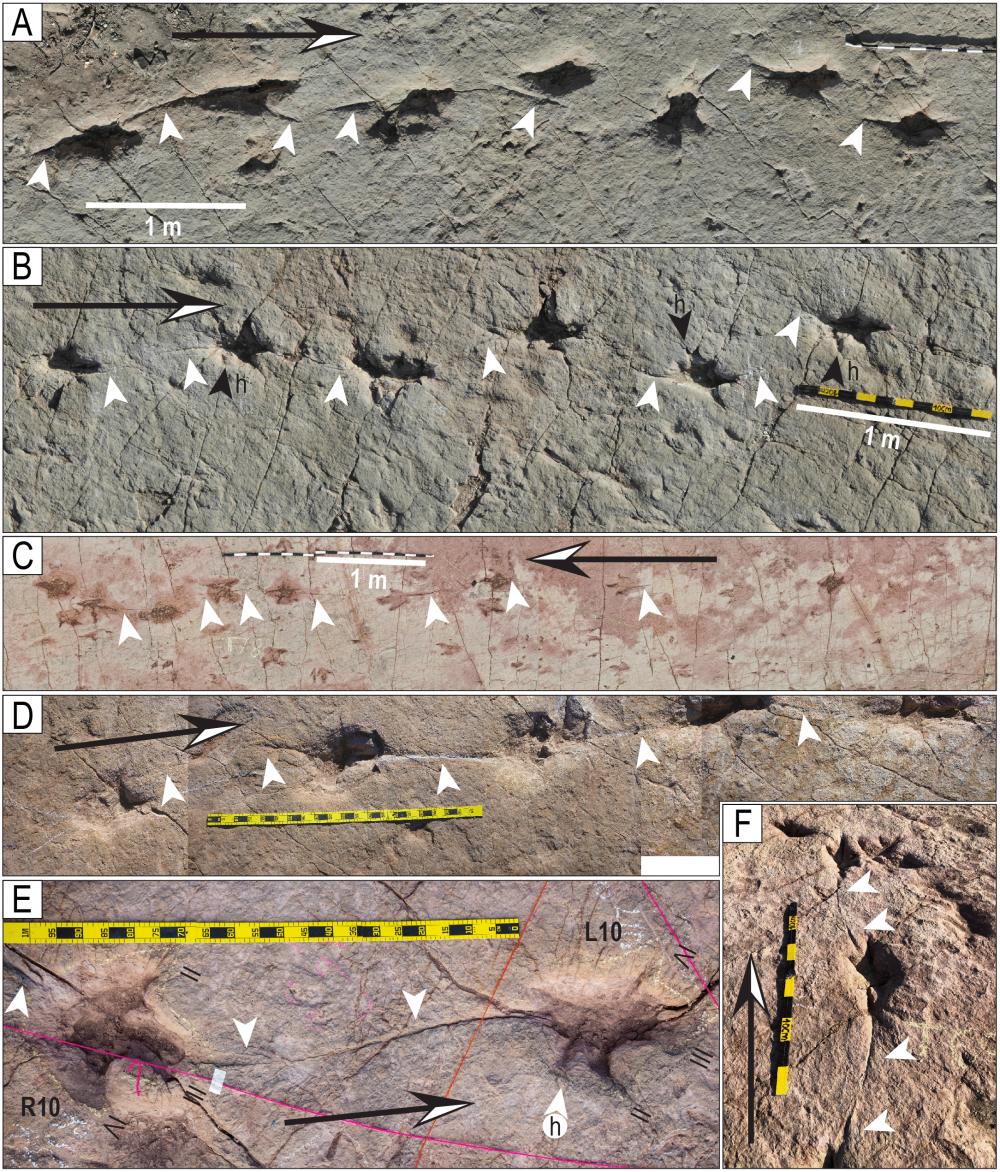
Partial view of trackways with tail traces. A) Trackway T32. B) Trackway T73. C) Trackway T78. D) Trackway CP7-6. E) Trackway T22-126. F) Trackway T22-2-148. Scales in D–F are 1 m. White arrowheads indicate the tail traces, and black arrowheads and the letter “h” indicate hallux impressions. Long arrows indicate the direction of movement.

**Fig 40:**
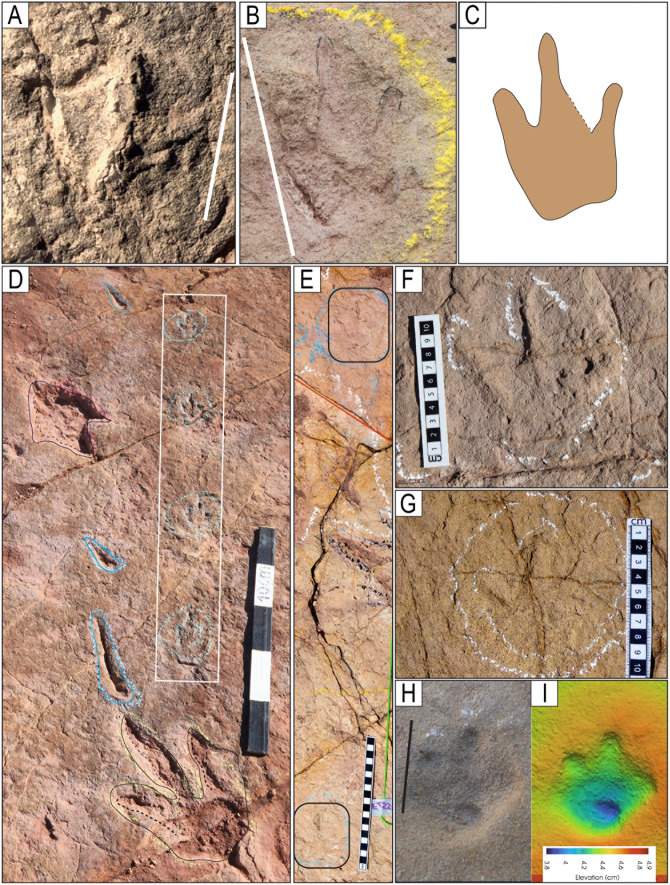
Miniature tracks. A) Track CP22−320, image obtained with a scanner. B-C) Track I22-54. D) Partial view of trackway T133 with diminutive tracks in a white rectangle box. This trackway consists of 99 preserved tracks. Notice other tracks of larger size in black outline and swim traces in blue outline. E) Trackway ET22−1 with two diminutive tracks, poorly preserved. F) Track I22-76. G) Track S23-9. H) Track CP9-S3 I) False-color depth map of CP9-S3. Scale size in A) 5 cm, B) 10 cm, H) 5 cm.

**Fig 41:**
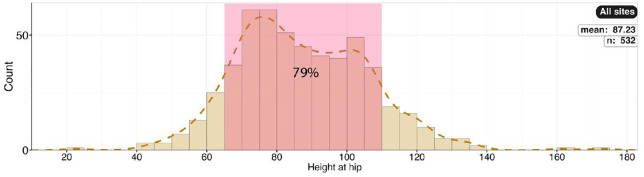
Histogram showing the calculated hip height for all sites (n = 532 trackways).

**Fig 42:**
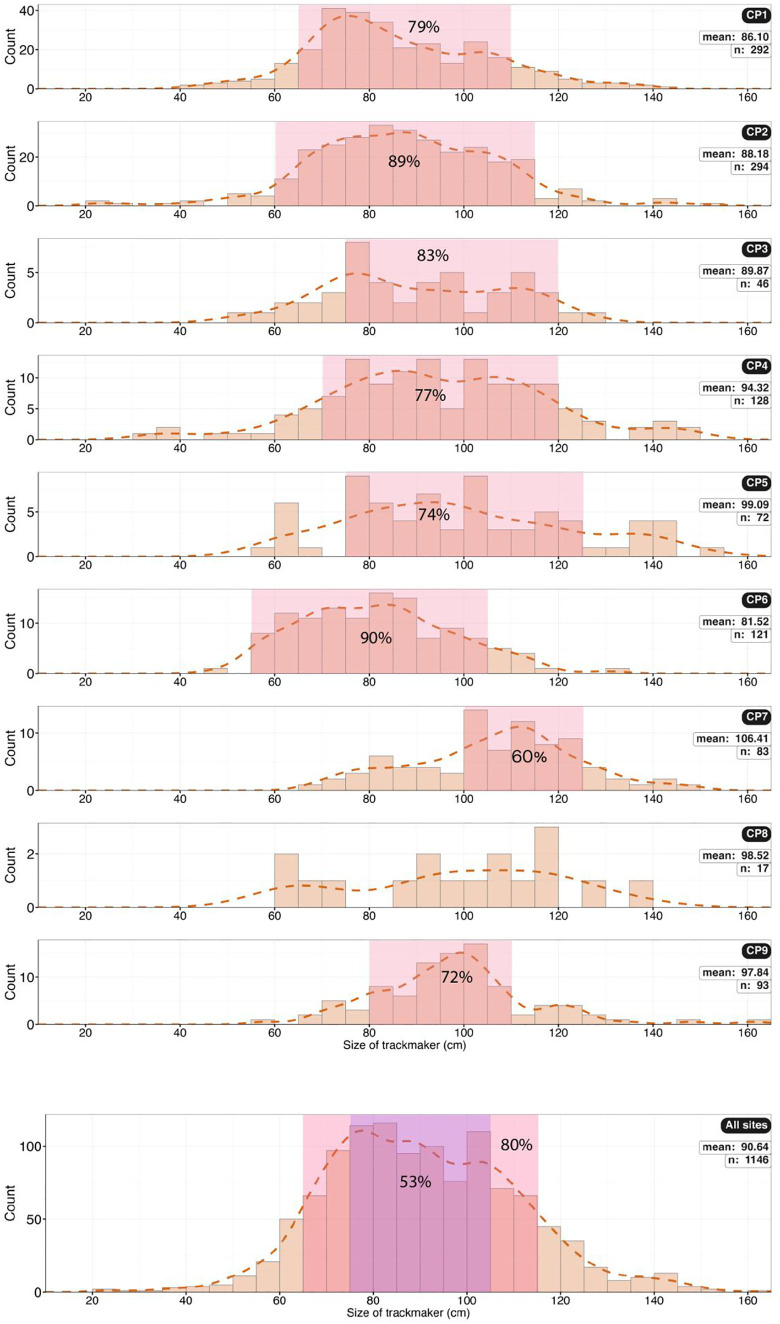
Histograms showing the size of trackmakers (height at hip) based on foot length (FL) for all the sites. The last histogram shows data for all the sites combined.

**Fig 43:**
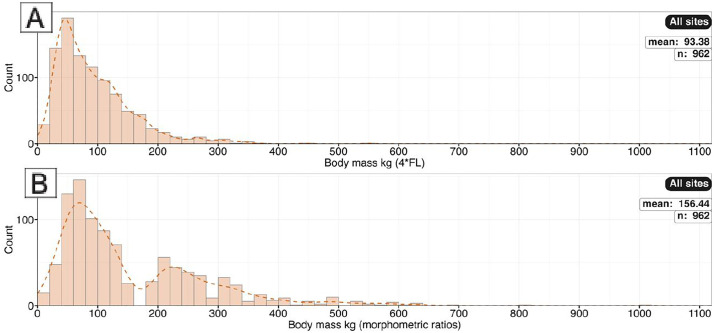
Body mass of trackmakers. A) Body mass as calculated by *h* = 4FL. B) Body mass calculated from morphometric ratios.

### Local stratigraphic context

The precise stratigraphic position of the CP sites within the El Molino is not known. Based on general comparisons, the CP bed is thought to be near the top of the first lacustrine sequence described by Camoin et al. [31] or the top of the lower El Molino Formation in the correlation scheme of Sempere et al. [28] The tracksites are located within a local exposure of approximately 95 m in thickness, measured from the base of the bounding valleys to the top of the hill situated between the valleys and the road. Exposed lithologies include interbedded limestones, sandstones, mixed carbonate and clastic sediments, mudstones, and claystones. The frequency and thickness of claystone and silty claystone beds increase upward in this area. The section below the CP sites (Maastrichtian, lower El Molino Formation of the Torotoro area) contains several carbonate beds with laterally extensive stromatolites (including the classic *Pucalithus*), which are part of an ongoing research project by some of the authors; their characteristics and significance will be described in a forthcoming paper.

Focusing on the stratigraphic context immediately above and below the tracked bed reveals five resistant beds, 30–50 cm thick, separated by silty claystone beds ranging from 1.2 to 2.6 m thick, as illustrated in a 10 m section (Fig 2A). While the five resistant beds associated with the tracked bed appear to look similar on the surface, each has unique characteristics that establish a diagnostic sequence to guide correlation. The contacts between the claystones and resistant beds are all sharp (not gradational); however, the bases of the resistant beds are locally undulatory, deformed, or they overlie mudcracked surfaces. Each resistant bed has been given a two-letter label for easy identification within the local area. The relatively flat area east of the gazebo and site CP3 forms an erosional plateau with an unconformity in the silty claystone overlying the tracked bed. This unconformity has been covered by up to 2 m of probable Pliocene or Pleistocene sediments.

### Description of the depositional units

#### Tracked bed – CP.

The fourth of the five resistant beds (Fig 2A and 2B) is the focus of this study, as it contains all the described tracks. It is named the Carreras Pampa (CP) bed after the designation of the National Park location. Weathered surfaces display variable colors (S1 Fig) but are generally pale yellowish brown (10 YR 6/2), while freshly fractured surfaces are a very light gray (N8). The composition of the bed consists of about 55% nested ostracod grains (Fig 2C and 2D), 10% ooids, and 35% siliciclastics. Nested ostracod grains (NOGs) are unusual, generally ellipsoidal, measure up to 400 µm across, and are well-sorted. In hand samples, broken grains reveal concentric layers. In thin sections, these grains comprise irregularly or regularly nested ostracod valves, typically cemented with fine, acicular epitaxial cement and occasionally by calcite mud (Fig 2C). Most NOGs contain 2–7 pairs of valves arranged with their internal surfaces facing each other, as when articulated. Fewer NOGs consist solely of one side of nested valves resembling an open disarticulated remnant. Even fewer NOGs contain fragments of randomly oriented valves within. Very few individual (not nested) ostracod valves are found in the rock. The identification of NOG components as ostracod valves is indicated by valve shape, size, and uniform thin shells with a radial crystalline structure, confirmed by the hinges and infolding (hooks) of the marginal zone (Fig 2D) visible on some valves.

Other grains in the CP are siliciclastics and ooids. Siliciclastic grains are dominated by very fine-grained, moderately to poorly sorted angular quartz, along with a smaller fraction of medium sand-sized and rounded grains. The very fine-grained fraction includes minor feldspars. Clastic grains are distributed throughout most of the unit and are often concentrated in subparallel laminae, only a few grains thick. In some locations, the base of the CP bed is dominated (80%) by the siliciclastic grain fraction. Ooids have a median diameter of 550 µm and display mixed radial and concentric layers, with many exhibiting cerebroid textures. Nuclei consist of ostracods, quartz grains, or calcitic mud peloids. Grain packing is moderate, with roughly equal amounts of point and long contacts. Cements are mainly acicular calcite, with patches of microspar and small quantities of degraded hydrocarbons. No porosity was observed.

The tracked CP bed generally measures about 47 cm in thickness, with local variation between 28 and 58 cm over a 1.5 km outcrop. A sharp contact separates the tracked sandstone from a mudstone, characterized by a sharp basal contact, small loading structures, and rare mudstone rip-up clasts near the base. The CP is overlain by a silty claystone that transitions from a pale olive-colored (10 Y 6/2) band a few centimeters thick to a predominant grayish-red (10 R 4/2) color in most of the claystone unit. Small-scale trough cross-bedding is the dominant sedimentary structure in the lower two-thirds of the CP bed, with trough widths ranging from centimeters to about a meter and trough depths between 2 and 30 cm. Irregular planar to low-angle bedding appears locally in the middle of the bed, along with thin beds and lenses. A clay-rich lamina creates a parting surface 10–15 cm from either the base or the top of the CP in many parts of the exposed bed, though it is not continuous across all sites. Below this surface, an older set of dinosaur tracks is visible in cross-section and, in a few instances, deforms the basal contact of the CP bed. The older trackbearing layer can be seen in cross-section along the exposed northern edge of the CP outcrops (Fig 1C, white line, S1A Fig white line) and in several other exposures outside the study area.

The upper portion of the bed is dominated by ripple cross and wave ripple cross lamination (S2A and S2B Fig). Wave ripple bedforms occur on the upper surface of the tracked bed in all the sites, with the best exposures in sites CP1-CP3, CP6, and CP9 (Fig 1C, wave symbol, Figs 2B and S2E).

Invertebrate trace fossils are abundant throughout the unit, appearing as sub-horizontal to sub-vertical burrows. Vertical burrows surrounded by rosettes of sediment on the tracked surface are found in many locations on the upper tracked surface. Theropod tracks, which are the focus of this paper, along with avian tracks, disrupt the upper surface of CP.

#### Orange bed – OB.

The stratigraphically lowest resistant bed in the local section (Fig 2A) is designated as the orange bed (OB) due to its color (dark yellowish orange, 10 YR 7/4). This bed is generally not exposed in outcrops where the slopes are low. The lithology consists of limestone, with allochems comprising about 90% NOGs and 10% ooids (Fig 2C). Early cements are acicular or bladed epitaxial calcite, overlain by microspar and spar that contain remnants of degraded hydrocarbons and about 5% moldic porosity. Due to its lesser resistance compared to other beds, sedimentary structures are typically not visible. Small amplitude trough cross-bedding was observed, and in one location, linguoid bedforms were visible on the upper surface.

#### Reddish bed – RB.

The second stratigraphically higher resistant bed is known as the reddish bed (RB) because it is the reddest of the five (grayish red, 10 R 4/2). This bed is composed of limestone with approximately 50% ooids, 30% NOGs, and 20% micritic intraclasts. The cements present are sparry, and no porosity was observed. Weathered surfaces appear grayish orange (10 YB 7/4) to pale yellowish brown (10 YB 4/2), while freshly broken surfaces range from pinkish gray (5 YR 8/1) to very light gray (N8). At the base of the RB unit, weathered surfaces typically exhibit distinctive raised granular laminae of ripple cross-lamination or small trough cross-lamination (Figs 2A and S2B–S2D). The remainder of the bed also displays faint cross-laminae of similar structures.

#### Wave rippled bed – WB.

The third resistant bed is called wave-rippled (WB) and forms moderately resistant pale brown (5 YR 5/2) outcrops. It comprises about 87% NOGs, 3% ooids, and 10% fine clastic grains dominated by angular quartz. Several exposures of the upper surface of the bed are present in the area, typically showcasing sharp-crested wave ripple bedforms with amplitudes of 3 cm and periods of 8 cm (Figs 2B, 6A and S2E). Their ripple crest orientation is consistent with an average of 140º–320°.

The fourth bed features the tracks of this study. It is referred to as the Carerras Pampa (CP) bed, which has already been described above.

#### Evaporite crystal bed – EB.

The fifth bed, the evaporite crystal-bearing bed (EB), is generally the most resistant of the five and produces pale yellowish-brown (10 YR 5/2) rectangular blocks that form prominent outcrops and contribute to colluvium on outcrop surfaces. Grains consist of about 90% NOGs, 3% ooids, 4% micritic clasts, and 3% angular quartz and feldspar. The rock contains equant calcite spar cement that appears phreatic in origin, degraded hydrocarbons, and retains about 10% porosity (Fig 2F). Molds of rhomboidal crystals 2–5 cm in size are present in the upper third of the bed and appear to be pseudomorphs of evaporite minerals that grew in situ, which were the focus of naming the bed (S2F Fig).

#### Mudstones.

Silty mudstones that separate the resistant units are all similar (S2G Fig). Illite dominates the oriented X-ray diffraction patterns, while minor amounts of quartz appear in the bulk XRD analysis. Freshwater fern and green algae palynomorphs, along with organic debris, are present in the reddish and olive mudstones. The silty mudstones below the local section contain ostracod valves and charophytes, but neither was observed in the mudstones associated with the CP-tracked bed. The mudstones are generally grayish red (5 R 4/2), with a pale olive (10 Y 6/2) color near the contacts with resistant beds.

### Methods

#### Site access.

The Carreras Pampa tracksite is an open field area in the TTNP, frequently visited by tourists who must access the site accompanied by tourist guides and obtain authorization from TTNP rangers. The tracksite was known to many tourists and scientists but remained unstudied until this study. The Servicio Nacional de Áreas Protegidas (SERNAP) (National Agency of Protected Areas) granted official authorization to this team for fieldwork conducted in May 2019, May–June 2021, April–May and August–September 2022, April–May 2023, and April–May, August–September 2024. Initial work involved cleaning debris from the surface of the exposure using brooms. Scattered, heavy cobbles and boulders were lifted and relocated to the edges of the tracksite. Small areas at the edges of the study sites were cleared of overlying sediment to reveal expected tracks of trackways and to observe undisturbed contacts between tracks and the overlying sediment. This cleaning was performed by local workers supervised by the scientific team using large and small brooms, shovels, and small hand picks to loosen the silty sediment washed into the tracks.

#### Sites and numbering systems.

The different study sites were successively named CP1 to CP9 as they were examined (Figs 1C and S2 Fig). Trackways were designated with acronyms CP, TO, or T, followed by the year and trackway number (e.g., CP22−1, TO22−1, T22-1) or T-trackway#. Individual tracks were outlined in chalk and numbered as right (R) or left (L) based on their position in each trackway, designated R1, L1, and so on. Due to the large number of trackways and high density of tracks, we used yarn of various colors to mark the trackways, which was particularly helpful for tracing trackways with turns. The yarn is visible in many of the photographs.

#### Terminology.

In this study, the terms “track,” “footprint,” “print,” and “impression” are used interchangeably to refer to the trace left by the contact between the autopodium and the substrate during locomotion. In ichnology, a trackway refers to a sequence of consecutive footprints an organism leaves as it moves across a surface. While there is no universally fixed minimum number of tracks required to define a trackway, it is generally accepted that at least three consecutive tracks are necessary to analyze and interpret the trackmaker’s gait, speed, and behavior effectively [38]. This standard allows researchers to observe patterns such as stride length and track spacing, which are crucial for accurate behavioral interpretations. In this study, however, we chose to define a trackway as the succession of two or more prints of either autopodium, because most trackways with only two tracks visible occurred near the edges of the study sites, where additional tracks could be exposed by removing the sediment cover. The use of two consecutive tracks to define a trackway was also used by Kozu et al. [39].

One of the main challenges in studying vertebrate tracks in the field is identifying the outer edge of the print [40]. Defining the boundary of the direct feature (where the expulsion rim begins) is difficult in most footprints, and maintaining a consistent method across thousands of footprints with different modes of preservation and morphotypes at several sites proved to be challenging. Due to the difficulty in determining the actual border of the print shaft that corresponds to the periphery of the autopodium, we drew the edge of the track at the outer edge of the slope that extends into the shaft. We acknowledge that this method does not ensure accuracy in the comparison between the morphology and dimensions of the tracks and those of the autopodium. Deep tracks exhibit a significant difference between their outer edge and inner impression. In some figures, we used a dotted line to indicate the inner impression in deep tracks or in deeper areas of relatively shallow tracks. We also recognize that other methods can introduce varying degrees of inaccuracy.

Solitary footprints were abundant and counted, except in sites CP1, CP5, and CP7. Most trackways are straight, and the average trends were easily determined by stretching yarn lines connecting along one side of the trackway. The identification of right and left tracks (known as siding a track) was established by the position of the tracks relative to the midline of the trackway and the inward orientation of the impressions of the digits. The animal responsible for fossil tracks is termed the “trackmaker” or “track producer,” and the substrate it walked upon is called “tracked surface” or just “substrate.” Tracks occurred at various degrees of depth. We used four categories of depth: very shallow tracks: < 0.5 cm, shallow tracks: 0.5–1 cm, deep tracks: 1–3 cm, and very deep tracks >3 cm.

The terminology used to describe the tracks and their dimensions is shown in Fig 3. Track dimensions include foot length (FL), foot width (FW), proximal depth (PD), distal depth (DD), number of digits (ND), and divarication angles of digits II − III, digits III − IV, and digits II − IV (total divarication). Foot length is measured straight from the tip of digit III and along its middle to the bottom of the heel impression. FL and FW are measured to the anterior end of the digits, not to the end of the claw traces (Fig 3B). The anterior triangle width (ATW) is the same distance as FW. The anterior triangle length (ATL) is the distance between the tip of the impression of digit III (excluding the detached impression of the claw) and the line between the tips of digits II and IV when measuring FW or ATW (Fig 3B). Divarication angles are measured between the midline of each digit (Fig 3B). In tracks with an extended impression of the heel, the length is measured from the tip of digit III to the closing of the line of the heel impression without including the extension (Fig 3C). We measure trackway pace length (PL), stride length (SL), and pace angulation (PANG) (Fig 3D). The width of the angulation pattern or trackway width (WAP) is measured perpendicular to the stride length. In contrast, the trackway pace angulation (PANG) is the angle between the right and left footprints in a bipedal or quadrupedal trackway, measured where the footprints form a sequence of three successive tracks (Fig 3D). Many well-preserved tracks were recorded in detail via photographs. The categories of track size (Fig 3E) follow the groups suggested by Marty [41].

The morphological description of the morphotypes used the terminology illustrated in Fig 3F. The terminology used in describing the tracks in cross-section follows Allen [42], Jackson et al. [43], and Milàn et al. [44]

Foot rotation (FR), the foot’s orientation to the trackway midline (Fig 3D), was measured in the trackways of sites CP6 and CP9. In the literature on dinosaur ichnites, two opposing classifications of foot rotation have been used. Some authors use the term “negative” for inward rotation [e.g., 45–47], whereas others use the term “negative” for outward rotation or “positive” for inward rotation [e.g., 38, 48–50]. The sign of the measurement in Table 1 is negative for inward rotation and positive for outward rotation of the track, consistent with the terms “inward” and “outward” rotation in the text.

#### Statistical methods.

Statistical analysis was carried out using the software R version 4.4.2 (R: A language and environment for statistical computing (http://www.r-project.org). Standard plotting methods were used. No outliers were removed. Missing data were ignored in analyses; no substitution was done.

#### Calculations for height and track density.

The height at the hip (*h*) (and thus the size) of trackmakers was calculated as *h = *4FL [51,52]. Calculating *h* in this way provides the trackmakers with a more conservative speed estimate [53]. The gaits of trackmakers were estimated using the principle that for bipedal dinosaurs, the transition from walking to running occurs when the ratio of SL/*h* exceeds 2.0 [51,53,54]. The density of tracks was assessed from the total tracks/total area and from portions of various sites. One area of site CP1, measuring 2.7x2 m, another measuring 4x4 m, and one area in site CP2, measuring 10x10 m, were delineated, and all the tracks were marked with chalk and counted.

#### Field measurement.

On-site measurements were taken using a measuring tape, precision rulers, and a Procise^TM^ digital caliper. Due to the high number of tracks, only the first three, and in some cases, the first six best-preserved continuous tracks in a trackway were measured. However, at sites CP6, CP8, and CP9, all the tracks were measured, including their dimensions, pace, and stride distances.

#### Photos and lighting.

Photographs and scans were taken using multiple cameras, including a Canon 6D with a 24–105 mm lens, an Olympus TG4, a Nikon S3200, and a Canon 7D Mark II with lenses EF-S 10–22 mm and EF 24–70 mm. We manually excluded photographs of low quality or containing foreign objects (e.g., the foot of the photographer) or with spots of excessive light from the input data set. We used a DJI Mini3 Pro drone for aerial photos. 3-D images were obtained using an iPad Pro and an iPhone Pro 13, with the software applications 3dScanner App (v. 2.1.3) and Scaniverse (v. 3.0.2). Images in illustrations were processed using Adobe Lightroom Classic 13.1 and Adobe Illustrator CS2024. No tracks were collected, nor were any track molds made.

Most tracks display their morphological details best in low-angle sunlight, particularly during the morning and afternoon. However, the photographs for the photogrammetry models were taken under cloudy conditions or using a tarp to create even shading within the area being photographed. Photogrammetric models were developed and cleaned using MetaShape software (v. 1.7.5). The 3D models were then imported into MeshLab (v. 2023.12) to rotate them to the XY plane, creating accurate depth maps. In the final step, scale bars and false-color depth maps were made using the software package ParaView (v. 5.10.0). We created rose diagrams showing trackway orientation using the software GeoRose (v. 0.5.1.1). We used Excel (v. 16.90.2) to organize the data on dimensions collected in the field and the R-core-Team software for statistical analysis. [55]

## Walking tracks and trackways

### Descriptive ichnology of tracks – General aspects

The tracks at Carreras Pampa are preserved as concave epirelief (concave epichnia), except for a few tracks of the style of preservation M8, which are preserved as convex epirelief. They are true walking tracks defined as direct structures created by the underside of the autopodium on the original substrate’s surface [56]. In the terminology of Gatesy [57], the studied prints consist of direct features, which are the portions of the tracks formed by grains that were in immediate contact with the autopodium, and indirect features, which are the other sedimentary distortions formed during track formation (e.g., displacement rims). True tracks are typically defined as those preserved on the exposed surface “upon which the animal walked” [58, p. 304].

Multiple lines of evidence indicate that the tracks are true and not undertracks, and therefore, were made on the original surface: 1) the presence of well-preserved swim traces; 2) the tracks and swim traces are superimposed onto ripple marks, which they locally disrupt; 3) the presence of displacement rims (expulsion rims, bourrelets) in both tracks and swim traces; 4) the presence of digital pad impressions in some tracks; 5) the occurrence of bird tracks and diminutive tridactyl tracks; 6) the absence of deformation structures in the overlying fine-grained sediments; 7) the lack of deformation in the overlying sediment above the tracks observed in cross-sections, except for deformation caused by the filling of the track concavity; and 8) the presence of rosette-like burrows within the tracks.

Particularly significant is the occurrence of swim traces and the co-existence of small and miniature tracks alongside larger tracks. Small and miniature tracks on a substrate surface several centimeters above would not have produced undertracks on the rock surface we observe today [59].

The burrows form rosette-like structures on the bed’s surface after the dinosaurs walked on the substrate, and their formation within the impressions of tracks indicates that these impressions are authentic tracks on the original surface where the animals walked. Furthermore, we examined over 500 m of exposed green and red mudstones above the trackbearing layer. We did not observe any deformation resulting from the pressure of an autopodium or any track preserved as convex epirelief or natural cast. Thus, the impressions are genuine tridactyl tracks rather than undertracks, and therefore, the dimensions of the footprints accurately reflect the original anatomy of the trackmaker’s foot.

The tracks are digitigrade and tridactyl, with very few exceptions of didactyl tracks found alongside tridactyl tracks. They contain limited information about the anatomy of the trackmaker and are attributed to theropod dinosaur trackmakers based on their tridactyl morphology (some tracks show the impression of the hallux). They are longer in length than width, mesaxonic (where digit III is longer than digits II and IV), with individualized digits II, III, and IV oriented anteriorly, often terminating in an acuminate point, and featuring claw impressions. Due to the high abundance of tracks at the tracksite, overlapping is relatively common but not predominant.

Most tracks in all morphotypes have a well-marked digit III impression, often deeper than the impressions of digits II and IV. This reflects the greater weight placed on the medial digit, which tended to sink deeply into the substrate. Reflux or sediment collapse inside the footprints is observed only in the impressions of digits II and IV in morphotype T3, not in the metatarsophalangeal area or digit III. On the north side of site CP2, a few concave epireliefs are preserved as undertracks, featuring less-impressed digits and smooth edges (Fig 8). Phalangeal and metatarsophalangeal pads are indistinct (lacking clear diagnostic pad impressions), except in some tracks of preservation styles M3 and M4, which show details of the autopodium pads (Fig 5K). Tracks do not exhibit differences in preservation across the various sites studied (aside from the undertracks mentioned above) or between tracks that were exposed before our study and those we revealed by removing the overlying sediment at the site edges. Exceptions include tracks affected by plant root growth before exposure or recent cracking.

Claw prints are commonly continuous with the digits, and very few exhibit a separation by a transverse crease. In many footprints, the claws are deflected sideways or backward, a trait not present in all tracks of every trackway and more conspicuous in tracks of style of preservation M4 (Figs 5H, 5I, 12A, 12C, 12F, 12H, 13C and 13J).

Many tracks show an indentation (or notch) behind the proximal margin of digit II, a characteristic of theropod tracks (Figs 11C and 12B). This notch is characteristic of theropod tracks, found, for instance, in Triassic theropod footprints in Southern Brazil [60], in Late Jurassic tridactyl dinosaur tracks from the Swiss Jura Mountains [61], in tracks from the Lower Jurassic Navajo Sandstone, Utah, the Lower Cretaceous Jiaguan Formation, Sichuan Province, China, the Upper Cretaceous Xiaoyan Formation, Anhui Province, China [62], the Lower Cretaceous of the Urbion Group, Cameros Basin in northern Spain [63], the Upper Cretaceous Calcare di Bari Formation in southern Italy [64], the Upper Cretaceous of Morocco [65], and the Upper Cretaceous Loncoche Formation in Argentina [66]. The medial notch is also very common in the theropod tracks found in other tracksites of the El Molino Formation in Bolivia, for example, the Km99 tracksite in the TTNP [24]. In some tracks, the notch appears at the base of digit IV, a rare feature also observed in theropod footprints of the Upper Cretaceous of Italy [67] and the Lower Cretaceous Batá Formation of Colombia [68].

Very few tracks display rims of displaced sediment or bourrelets, which are generally relatively shallow. Exceptionally, some deep tracks may exhibit prominent expulsion rims, such as tracks L2, R3, and L3 in trackway CP6–9 (Fig 28A and 28B), which show a thick bourrelet on the exterior side of the tracks. The tracks lack a crescent-shaped sandstone ridge (backfoot displacement ridge). Ejecta (sediment ejected during the kick-off phase) and mounds of sediment inside the tracks are absent. The sediment outside the tracks does not exhibit pressure pads or tension, nor does it display radial or concentric fractures resulting from the autopodium’s pressure on the substrate. Very few tracks display compressed sediment between two toes, a characteristic that may occur in footprints made by running theropods [69]. The tracks lack crescent displacement rims immediately in front of footprints in trackways made by running dinosaurs [69].

### Track preservation

The preservation of tracks was evaluated by comparing exposed tracks along a trackway with freshly uncovered tracks on the continuation of the same trackway that were buried beneath overlying Cretaceous sediment. In these cases, the newly exposed tracks exhibited the same quality of preservation as much of the naturally exposed tracked surface, indicating that, to this date, erosion has not significantly reduced preservation quality.

Regardless, tracks at Carreras Pampa exhibit a wide variety of preservation styles and morphological clarity, reflecting the differences in the gaits of the trackmakers as well as the rheological conditions of the tracked surface. Differences include examples with the entire contour of the autopodium imprinted, tracks preserved only as shallow and indistinct depressions, and tracks preserved as small indentations corresponding to claw marks. We distinguished between the style (or mode) of preservation and morphotype, a distinction also made in other studies [70–72].

### Style of preservation versus morphology

The term “mode of preservation” is commonly used in the literature [73–77, e.g., 78–81] but has not been adequately defined. It is akin to the term “style of preservation,” which, to our knowledge, was first used by Lockley et al. [82] in their study of dinosaur tracks in the Lower Cretaceous Cedar Mountain Formation of Utah, but they did not define it. The term “style of preservation” is also widely used in vertebrate ichnology [e.g., 83]. Some authors use both terms interchangeably [40,84,85].

Morphotype refers to the categories of tracks based on the morphological traits of the prints, including the shape of the digits, claws, and metatarsophalangeal area, as well as their dimensions, such as length, width, interdigital angles, depth, and the orientation of digits and claw marks. Morphotype refers to the categories of tracks based on the morphological traits of the prints (shape of the digits, claws, and metatarsophalangeal area) and their dimensions (length, width, interdigital angles, depth, orientation of digits and claw marks, etc.).

An example will illustrate the difference between our use of morphology and the style of preservation. Two tracks may show the same style of preservation, capturing the entire contour of the print, including the metatarsophalangeal area, the three digits, the hallux, and the claw marks. However, the two tracks may exhibit different morphotypes; for example, one has strong mesaxony, including slim digital impressions, a round heel impression, and the impressions of the three digits oriented forward, whereas the other track displays weak mesaxony with thick digit impressions, an acuminate heel impression, and the impressions of digits II and IV oriented laterally–that is, two distinct morphotypes in the same style of preservation.

The morphology of the footprints and the style of preservation can vary significantly within the same trackway, determined by the morphology of the animal’s autopodia, the dynamics of the animal’s movement (gait), the weight of the trackmaker, and the rheological conditions of the substrate, such as firmness, surface morphology, grain size and composition, moisture level, the presence of microbial mats, and later erosional processes [86–91].

It is unsurprising that, given the large number of tracks (16600) in the tracksite, we found several styles of preservation and morphotypes. At the Carreras Pampa tracksite, various preservation styles are commonly observed in each trackway. Therefore, each style category will be described.

### Style of preservation definition

Here, we define the style of preservation as the pattern of print impression that includes the presence of the digit impressions (the degree to which digits are impressed and distinguishable), the occurrence and depth of claw impressions, the relative depth of the metatarsophalangeal area, and the presence of the hallux impression. Style also encompasses whether the tracks are exposed as concave epirelief, convex epirelief, natural cast, undertrack, or as a vertical cross-section. Based on these criteria, we distinguish eight different styles of preservation, M1–M8 (Figs 4–10 and S3–S9 Figs).

#### Style of preservation M1 – Ghost preservation, lack of rims, only claw marks.

Tracks of this style consist of sets of two or, more commonly, three indentations or puncture-like marks arranged in a linear pattern of regularly spaced right and left traces, without the impression of the heel. (Figs 4 and S3). Some tracks may show only one indentation corresponding to the claw of digit III, but this indentation can be assigned to the track succession in the trackway. The markings are typically round, conical, narrow, and deep, but can also be elongated, fusiform, or tear-drop-shaped. The three indentations in each set are arranged roughly in an isosceles triangle, with similar distances between digits II and III and between III and IV, and a longer distance between digits II and IV, corresponding to the marks of the claws of digits II, III, and IV of theropod tracks. (Fig 4H). Some trackways exhibit a distinctive asymmetry of digits II and IV relative to digit III. The relative distance between the tracks, the asymmetry among them, the position of the tracks in an alternating pattern of right and left sides, and their occurrence in association with “true” walking theropod tracks support the idea that these traces are theropod tracks.

In these traces, the print of the rest of the autopodium is either absent or barely distinguishable. Some tracks exhibit a very shallow outline of the plantar or heel area, only visible in very low-angle light (Fig 4F and 4G). Based on the distance between the preserved claw traces, the tracks with this style of preservation are small (10–20 cm) to medium (20–30 cm) in length, made by theropods with hip heights ranging from 0.4 to 1.2 m.

At Carreras Pampa, the M1 style of preservation is prevalent and remarkably plentiful, with numerous entire trackways made up solely of puncture-like markings [17] (S3 Fig). Additionally, many solitary sets of these markings are observed. These claw-only tracks appear on the same surface and near deeper tracks with digits and a fully printed plantar area, similar to other preservation styles. The co-occurrence of the two track styles suggests that M1 tracks were made when the sediment was relatively firmer. The presence of indentations in the substrate and the posterior displacement rims in many claw prints indicates that the substrate was cohesive, and the distal ends of the toes with the claws were pressed into the sediment just before the animal withdrew its feet from the substrate.

Morphologically, they resemble some tridactyl tracks in the Guanghui site from the Lower Cretaceous Taoqihe Formation in Central Heilongjiang, China [92, Fig 10]. The authors interpreted this type of track as digit traces without the heel impressions and attributed them to ornithopod dinosaurs without providing the reasons for such a conclusion.

The morphology and pattern of occurrence of the claw-only prints at the Carreras Pampa ichnosite match what Thulborn and Wade [93] and Thulborn [38] interpreted as tracks formed during the kick-off (K) phase, as observed at the Lark Quarry Environmental Park in western Queensland, Australia. Those authors distinguish three phases during the trackmaker’s step-cycle: T, or the touch-down phase, when the foot makes contact with the substrate; W, the weight-bearing phase, when the foot sinks deeper, leaving an impression; and K, the kick-off phase, when the foot is released from the sediment. During the kick-off phase, the toes are lifted from the substrate, and in the process, the claws are incised into the sediment and pushed backward as the body moves forward. Thulborn [38, Fig 5.12] called these prints “incomplete footprints made by the tips of the toes.”

In Carreras Pampa, the movement of the autopodia left puncture-like markings in the substrate, sometimes accompanied by small rims of displaced sediment, most frequently at the posterior ends of the indentations. As the autopodia lifted, they were occasionally pushed backward to allow the animal to gain thrust forward, thereby forcing the soft sediment posteriorly and forming the expulsion rims. Due to the high degree of firmness and cohesion of the sediment, in most tracks, the foot made no impression on the substrate during the T or W phases, or the impression was very shallow. Only the claws left their imprint, as most of the animal’s weight was supported by the distal parts of the feet. Since digit III is the longest, its trace is often the deepest and most pronounced; in some tracks, it is the only one preserved (Fig 4H, track L3). Consequently, some of these trackways consist of three, two, or just one puncture-like mark alternating between the left and right sides.

#### Style of preservation M2 – Claw traces are absent or marked as narrow, linear grooves; poorly defined, very shallow depression, and indistinct edges.

Tracks of this style of preservation lack claw traces (Fig 4I–4L) or appear as narrow, linear grooves, most commonly along the middle of digit III (Fig 4M–4P) (S4 Fig). The prints feature indistinct edges, which consist of a very shallow, uniform depression (<0.5 cm) that makes the track recognizable, but mostly lacks morphological details. The metatarsophalangeal area is very shallow or absent. Overall, the outline of the track, including the three digits, is barely observable except under low-angle light conditions, although they become apparent in image analysis.

#### Style of preservation M3 – Claw marks shallow or absent, shallow or very shallow depression, track well marked.

Prints of this preservation style are shallow or very shallow, including the claw marks (Figs 5A–5D and S4 Fig). The outline of the track is distinct, making it recognizable, but mostly lacking morphological details. The posterior end of the heel may be absent or only very shallow. Although uncommon, the M3 style may include the hallux impression. All three digits are well-marked, although the trace of digit III and its claw may be more conspicuous than the other prints. Exceptionally, some tracks of this style may display shallow pad impressions (Fig 5D).

#### Style of preservation M4 – Well-defined tracks, sharp claw marks.

Tracks of this style of preservation vary from shallow to deep (0.5–3 cm) but are well-defined. All the digits are clearly marked, sometimes featuring a narrow interdigital expulsion rim (Figs 5E–5K and S5 Fig). The claw marks can be sharp and deep, though they may also be shallow, recognizable, or absent. The heel impression is distinctly marked and, although it can be shallow, is rarely missing. The impression of the hallux is quite common (Fig 5E, 5I and 12H). In this style of preservation, a linear groove or raised ridge may appear behind the heel in alignment with digit III (Figs 12H, 20 and S5 Fig). Exceptionally, some tracks in this style may exhibit shallow padding impressions (Fig 5K).

#### Style of preservation M5 – Very deep, elongated tracks, lack of morphological details.

Tracks are usually very deep (>3 cm, most commonly 5–15 cm) and lack most morphological details. They are elongated, with the impression of the metapodium. The prints feature smooth edges and vertical walls (Figs 6 and S6 Fig). They often lack distinct digit traces or show poor marking. Usually, they are preserved as round extensions of the shaft, featuring one or two short lateral impressions corresponding to digits II and IV but lacking claw traces (Figs 6 and S6 Fig). Many of these tracks exhibit tetradactyl characteristics due to the impression of the hallux on the lateroposterior side of digit II, resembling a claw mark (Fig 6E). These tracks are frequently associated with posterior ridges or tail-drag traces (Figs 6, 23, 24, 25C, 25D, 26A, 26B, 39, S6 Fig).

#### Style of preservation M6 – Very shallow, elongated depressions, lack of digits and claw marks.

The tracks in this style of preservation are preserved as round or semi-round. They are most frequently elongated, narrow, or kidney-shaped, forming very shallow to shallow (<0.5–1 cm) depressions with gentle slopes and lacking morphological detail (Figs 7 and S7). Some show a hint of digits II and III in smooth lobes. These structures may correspond to the infilling of tracks preserved in an underlying layer, visible in cross-sections at several locations where the edge of the tracking surface outcrops (Figs 9 and S7 Fig). A detailed description and discussion are found in another study.

#### Style of preservation M7 –Tracks preserved as undertracks or partial sediment filling.

This style of preservation is relatively scarce in Carreras Pampa. It is observable only near the edges of some sites (Fig 1C, south of the white line), where the upper surface of the tracked layer has recently eroded, revealing undertracks preserved as convex epirelief or concave epirelief. These tracks are tridactyl and display a faint outline of the digits and the metatarsophalangeal area, but lack significant anatomical details (Fig 8), except for the impression of the claw of digit III (white arrowheads in Fig 8A).

The undertracks in Fig 8A (white arrowheads) are in concave epirelief with layered sediment filling the concavities. They are overall rounded, but the projections of the digits are prominent. Some are very well defined, and the claw of digit III is distinctly marked. Undertracks preserved as concave epirelief, without layered sediment filling (Fig 8B–8D), exhibit smooth edges and poorly defined track morphology, with few or no anatomical details. They may show three, two, one, or no digits (Fig 8D) and lack marks of the claws and expulsion rims.

#### Style of preservation M8 – Tracks in convex epirelief or natural casts.

Only a few tracks are preserved in Carreras Pampa as convex epirelief or natural casts. They are found in fallen blocks of rock at the southern edge of CP7, where the tracked layer splits into two sublayers, leaving behind blocks of rock with well-preserved natural casts (Fig 9). Several of these tracks were removed from the site and are currently stored at the headquarters of the TTNP. These natural casts correspond to sediment filling the tracks in the lower trackbearing sublayer (ts2 of Fig 10A–10C). One of the best-preserved tracks is CPCS-27 (Fig 9B), which illustrates the padding of the feet. Track T22-2–354 (Fig 10D–10E) is significant because it displays the track’s cast in cross-section (black arrow) and in plain view, revealing an enigmatic sinuous extension of the heel that may correspond to the metapodium.

### Tracks in cross-section

Many tracks are visible in cross-sections along the edges of the study sites and in fallen blocks that can still be stratigraphically relocated (Figs 10 and S8 Fig). The tracks in the cross-section at site CP6 occur in blocks of rock detached from the trackbearing layer but remain in situ. One additional track in cross-section is preserved in a block of rock used to construct a sidewalk around the gazebo (Fig 10D).

Along the northern edge of site CP2, the trackbearing layer is exposed vertically, revealing the full thickness of the bed and its internal structure in cross-section, where several tracks are present. In this area, the layer measures 60 cm thick, comprised of planar and cross-bedded oolitic and ostracod-rich sandstone. The exposure is interrupted by a 0.5 cm thick lamina of mudstone (Fig 10C, yellow arrowhead). The thickness of the sandstone above and below the mudstone parting varies laterally along the outcrop, just as the continuity of the mudstone changes, having been removed by erosion and the infill of shallow channels in many areas. When the mudstone occurs as a continuous lamina above a track in cross-section, it clearly indicates at least two levels of tracks in the CP bed.

Tracks formed on older surfaces may also propagate as later sediment fills in the depressions, corresponding to the shallow, elongated structures interpreted as the style of preservation M6 on the tracked surfaces of the study sites (Fig 7). A fortuitous outcrop reveals tracks in cross-section across two levels (Fig 10A–10C). The track in the foreground cross-section of Fig 26A and the track in Fig 10C were printed on the younger surface (ts1), whereas the track in the cross-section in Fig 10B was printed on the surface of the lower level (ts2), marked with a yellow dotted line.

When an animal walks on a plastic substrate or loose sediment, the tracking surface and the underlying layers or laminae undergo deformation. Experiments demonstrate that the weight of the trackmaker is transferred radially outward into the substrate below and around the foot. The tracks in cross-section reveal a typical preservation pattern consisting of the deformation of the laminations of the trackbearing layer caused by the pressure of the autopodium on the substrate. In some cases, the deformation exhibits a downward compression to a lower vertex where the laminations seem to converge (Fig 10D, 10E, 10G and 10H). Other tracks in cross-section display convolute deformations and/or breakage of the underlying laminations (Fig 10A foreground, 10C and 10F). The sharpness of the deflection at the edge of the track in cross-section depends on the track’s depth.

A particularly complex print is displayed in Fig 10D, where the laminated sediment receiving the track is truncated laterally from the surrounding sediment. The laminations converge downward on each side, with the crests of the folds oriented downward and backward (df), indicating the direction of the foot insertion. The sediment in between is structureless (sl). These folds are the “sharp, nested Vs” of the track simulation experiments of Falkingham et al. [94], characteristic of penetrative tracks [56]. The narrow 1-cm-wide tongue (in yellow) at the lower part of the cross-section is the impression of the claw as it cuts through the bottom during the track production process. The deepest impression of the claw is expected in tracks made by trackmakers with sharp claws on their digits [44]. The shaft of the digit impression and the trace of the claw are straight and vertical, which is contrary to observations from other tracks in cross-section and in three dimensions, where the distal end of the digit III print shows an inward rotation caused by the claw being forced inwards [95]. The folds in the sediment in the upper half of the trace are oriented upward and outward (uf) toward the inner area produced by the upward movement of the toe during the initial kick-off phase of foot retraction and uplift. The claw penetrated approximately 17 cm into the soft substrate in this track. Given this depth, we hypothesize that this cross-section corresponds with the impression of digit III of a theropod pes. The study of theropod tracks in cross-section in the Upper Triassic of East Greenland by Milàn et al. [44] indicated that the deepest depression corresponds to the impression of digit III. This is consistent with our observations of very deep tracks in Carreras Pampa, where the depth range is 10–17 cm, and the impression of digit III is usually deeper than those of digits II and IV.

### Track morphology definition

Track morphology is expected to be uniform within the same trackway for a constant trackmaker moving on a substrate with homogeneous physical conditions. However, the opposite is most often the case, as no two footprints are identical, and any two footprints in the same trackway will differ in size and morphology [38,96–98]. Thus, the morphology of the footprints can vary significantly within the same trackway, which is determined by both biological and sedimentary factors [86–91].

At the Carreras Pampa tracksite, we observed notable variability both between and within trackways, showing differences in depth and track shapes. Some of these variations suggest changing conditions in the texture and consistency of the substrate, while others result from the dynamic interaction between the autopodium and the substrate. We call these morphological variations morphotypes.

### Track morphotypes

At Carreras Pampa, we distinguished eleven different morphotypes, labeled T1 through T11, based on the overall shape, the shape of the pedal area, the shape of the heel impression, the occurrence and shape of the medial notch, the relative size of digits, the relative size of digit III to the metatarsal area, the morphology of digit III (lanceolate, bulbous, slender/thick, curved/straight, sinuous), the asymmetry of digits II and IV in relation to digit III, and the morphology and orientation of digits II and IV (Fig 3).

#### Morphotype T1 – Slender tracks with strongly acuminate heel impression.

These tracks have the overall shape of an isosceles triangle. The metatarsophalangeal area is elongated, typically featuring an acuminate or narrowly rounded heel impression, along with a slight notch on one side (black arrowhead on Fig 11A and 11C). Digit III is well-printed, slim, lanceolate, and generally curved, with the tip directed toward the midline of the trackway. It is longer than digits II and IV, exhibiting strong mesaxony, and is as long as or longer than the metatarsal area. These tracks are longer than they are wide (FL/FW ratio is notably high, 1.8–2.3). The ratio of digit III to the metatarsal area ranges from 1:1.2 to 1:1.8. Digits II and IV may display significant asymmetry, with digit II protruding further forward than digit IV, due to the position of the hypex of digits III–IV being located further back than that of digits II–III (Fig 11A and 11B). Interdigital angles measure 23°–26° between digits II–III and 27°–30° between digits III–IV. While digit III is well printed, the prints of digits II and IV may show signs of limited sediment collapse and closing of the impression toward the claw; thus, these digits are often only partially printed, typically lacking, or occasionally exhibiting deep and very short claw marks. Digits II and IV are oriented anteriorly, though the claw traces may be oriented laterally or curved backward. Hallux impressions may also be present (Fig 11A). This morphotype is rare across all the studied sites.

#### Morphotype T2 – Very slender tracks with an extended, short, acuminate heel impression.

Tracks of this morphotype are narrow, with a prominent metatarsophalangeal print and a short, acuminate metatarsal (heel) impression (Fig 11E and 11F). The tracks may exhibit one or two notches on the medial sides. They are strongly mesaxonic, with the ratio of digit III to the metatarsal area being 1:0.8 to 1:2.1. Digit III is slender, lanceolate, or slightly curved inward. Some tracks demonstrate a significant asymmetry between digits II and IV. The digits are oriented anteriorly (straight); exceptionally, digit IV may be oriented laterally. The FL/FW ratio is low (1.1–1.5) in most tracks. Hallux impressions are absent. This morphotype is quite rare at the studied sites.

#### Morphotype T3 – Tracks with a strongly curved digit III and one or two narrow hypex areas.

This morphotype resembles morphotype T1 in the acuminate heel impression and the overall shape of an isosceles triangle (Fig 12). Some tracks exhibit a distinct notch on the medial side of digit II (Fig 12B, black arrowhead). They display strong mesaxony due to the closure of the distal parts of the digit II and IV impressions from sediment collapse. Digit III is short relative to the metatarsophalangeal impression, with a ratio of digit III to the metatarsal being 1:0.8–1.0. This digit is slightly curved toward the trackway midline, with pronounced inward narrowing of the hypex areas (white arrowheads) or only at the hypex area between digits III and IV (Fig 12A–12C). Digits II and IV are shorter than digit III and are oriented laterally. In some instances, the claw trace of digit II is directed posteriorly. FL/FW measures 1.6–2.0. Interdigital angles are 44° between digits II and III and 40° between digits III and IV. This morphotype is relatively abundant in the tracksite.

#### Morphotype T4 – Tracks with narrow hypex areas and anteriorly-oriented digits II and IV.

The metatarsophalangeal area is broad and ends with a square heel impression, occasionally slightly acuminate, featuring a notch on the medial side of digit II or digit IV (Fig 12G). The digits are oriented anteriorly and demonstrate strong mesaxony. The digit III impression is straight and lanceolate or curves inward, and the claw trace of digit II may be oriented backward (Fig 12G and 12H). The asymmetry of digits II and IV is minimal or absent. FL/FW ranges from 1.5 to 1.8. The ratio of digit III to the metatarsal area is between 1 and 1.5. Interdigital angles measure 23° to 33° between digits II and III and 23° to 36° between digits III and IV. The impression of the hallux is common (Fig 12D, 12E, 12G and 12H). This type of track is very abundant at the tracksite.

#### Morphotype T5 – Tracks with robust digit III and strong asymmetry.

Tracks of this morphotype feature a V-shaped metatarsal impression that ends in an acuminate or semi-rounded heel impression (Fig 13A–13F), or, in rare cases, with a subrounded heel impression (Fig 13E). Digit III is robust and lanceolate, sometimes curving inwardly, very broad at the base, and lacking pronounced hypeces. Digits II and IV are similar in size, with dII greater than dIV; they are short and wide, exhibiting strong mesaxony and asymmetry, and are oriented anteriorly or slightly inward. In some tracks, the anterior end of digit II is directed backward (Fig 13C). The ratio of FL to FW is 1.3–1.7. The ratio of digit III to the metatarsal area is 1.2–1.7. The interdigital angles range from 25° to 33° between digits II and III and from 23° to 39° between digits III and IV. Exceptionally, the angles may be more open, with interdigital angles II–III = 47° and III–IV = 54°. There is no hallux impression. This morphotype is abundant at the tracksite.

#### Morphotype T6 – Short and wide tracks with robust digit III.

T6 tracks exhibit bilateral symmetry, a wide metatarsophalangeal imprint, and a U-shaped heel impression (Fig 13G–13L). The medial notch is either absent or very subtle. Some tracks have notably thick digit impressions resembling those of ornithopod tracks. Mesaxony is weak. Digit III is short, robust, straight, and broad at the base, occasionally curving slightly inward. Digits II and IV are equal in size and directed slightly sideways. FL/FW ranges from 1 to 1.3. The interdigital angle between digits II and III is 24°–34°, and between digits III and IV is 31°–34°. No hallux impressions are present. This morphotype is not abundant at the tracksite.

#### Morphotype T7 – Wide tracks with round digits and strong asymmetry.

These are large tracks with a pronounced metatarsophalangeal impression in a generally wide, triangular shape, ending in an acuminate heel impression (Fig 14A–14C). Mesaxony is weak. A slight notch is present on the medial side of digit IV. There are thick, round digit impressions: in some tracks, the impression of digit III is notably wider and has a broader base than the impressions of digits II and IV. Digits II and IV are oriented sideways. The hypex area between digits III and IV may be quite acute. This morphotype appears only in three trackways at the studied sites (T22-246, T22-2–36, and T22-2–40). In trackway T22-2–40, digit III curves outward toward digit IV, and digit IV is longer than digit II, unlike most tracks in other morphotypes. The FL/FW ratio is 0.5–1.0. Digit III and the metatarsophalangeal impression have approximately equal lengths. The interdigital angle between digits II and III ranges from 30° to 54°, while between digits III and IV, it spans from 28° to 44°. The anterior ends of the digit impressions are not acuminate or pointed, as seen in other morphotypes, but rounded, particularly the impression of digit III.

#### Morphotype T8 – Tracks with strong asymmetry and an acuminate heel impression.

Tracks of this morphotype have short digits and a relatively large metatarsophalangeal area, ending in an acuminate heel impression (Fig 14D and 14E). They display strong mesaxony due to the incomplete impressions of digits II and IV. The printed side of digit II is straight, while a notch is present on the medial side of digit IV or at the heel impression. The hypeces are very prominent, significantly narrowing digit III at its base (white arrow, Fig 14D and 14E). Digits II and IV are oriented sideways. Digit III is small and short compared to the metatarsal print, exhibiting a lanceolate shape that is slightly curved inward. Strong asymmetry in digits II and IV is typical. The FL/FW ratio is 1.3–1.5. The interdigital angle between digits II–III is 21°–28°, while between digits III–IV, it is 26°–34°. This morphotype is very scarce at the tracksite.

#### Morphotype T9 – Tracks with round digits and metatarsophalangeal impression.

The T9 morphotype comprises large prints with round digits and metatarsophalangeal impressions (Fig 14F). The medial notch is absent. The digits are symmetric and oriented anteriorly. Digit III is prominent and round, narrowing at the base, with a surface area nearly as large as the metatarsal print and longer than it. The traces of the claws are absent. The ratio FL/FW is 1.3. The interdigital angle between digits II and III is 39°, and between digits III and IV is 34°. This morphotype is very scarce in the studied sites.

#### Morphotype T10 – Didactyl tracks with an acuminate metatarsal impression.

Didactyl tracks are missing a print of digit IV. Overall, their shape is conical, with the impression of digit III being approximately the same length as digit II (Fig 14G and 14H). The interdigital angle between digits II and III ranges from 19° to 24°. These tracks are very scarce at the tracksite and occur in trackways alongside tridactyl tracks. Thus, the didactyl shape is a preservation feature that does not reflect the anatomy of the autopodium. This morphotype is extremely rare at the tracksite.

#### Morphotype T11 –Tracks with a shallow, wide, elongate posterior impression.

Many tracks in Carreras Pampa exhibit an elongate posterior impression behind the heel print. If it were not for the presence of this posterior extension, these tracks could be classified alongside some of the other morphotypes. Nevertheless, this feature grants the distinction of a specific morphotype (Fig 15). These impressions are relatively common in the fossil record [40,99–105] and have been referred to by various names, including “elongate metatarsal,” “elongate metapodial impressions,” “metapodium,” “plantigrade,” “semi-plantigrade,” and “sitting” prints.

This type of track was first brought to attention by Charles N. Strevell in 1932 when he mentioned: “prints [in Utah that] show a long and well-developed heel mark” [106]. Their mechanism of formation has been interpreted as resulting from plantigrade gait associated with resting (sitting) behavior, sliding or slipping, squatting (crouching), or sinking deep into the soft substrate [e.g., 50,107,108]. Glenn Kuban [100] reported the occurrence of elongated dinosaur tracks in tracksites at the Paluxy River, Texas, which he interpreted as a facultative plantigrade or quasi-plantigrade mode of the walk, an idea also applied by Thulborn and Wade [98] for elongated tracks from Lark Quarry, Australia. *Grallator* tracks with elongated metapodium traces from the Newark Supergroup in Massachusetts and Polish Liassic deposits were interpreted as sitting impressions by Gierliński [103]. In the Upper Jurassic of Mattinata, Italy, Conti et al. [99] reported footprints with posterior elongation and described four consecutive theropod tracks with a very pronounced metapodium impression in an irregular sequence interpreted as made “during a very slow progression” movement “of an undecided or sliding trackmaker” (pp. 541–542). Kvale et al. [109] interpreted theropod footprints with elongated markings in the Middle Jurassic Bighorn Basin, Wyoming, indicating that the animal slipped on a wet substrate. Milàn and Loope [84] reported theropod tracks with “a posterior elongation of the track, resulting from a partial impression of the metatarsus during the stride” and interpreted them as evidence of a semi-plantigrade stance by the trackmaker. The example they show in their Fig 4B is very similar to those observed at the Carreras Pampa tracksite. Xing et al. [110] attributed several theropod trackways with elongate metatarsal impressions in the Lower Cretaceous Jiaguan Formation of Sichuan Province, China, to a plantigrade or quasi-plantigrade gait on extra-soft sediment. Elongate metatarsal impressions have been found in many footprints in the Cretaceous basins of NE Brazil, which Carvalho [111] interpreted as resulting from a low posture in mud flats or shallow water. Citton et al. [112] explained that elongate theropod tracks from the Cretaceous of Latium (central Italy) resulted from crouching and resting. Metatarsal marks have been reported in two ornithischian *Shenmuichnus* tracks of the Anomoepodidae ichnofamily in the Lower Jurassic of Yunnan Province, China, and interpreted as reflecting a sitting behavior [113]. More recently, Lallensack et al. [107] interpreted elongate dinosaur tracks “as deep penetrations of the foot in soft sediment.”

The elongated marks in the tracks at Carreras Pampa are not erosional and appear in otherwise common tridactyl tracks at the tracksite. They manifest in three styles of track preservation: very deep tracks of style M5 (Figs 6, S6 Fig and S9 Fig), shallow tracks of mode M3, and well-marked tracks of style M4 (Fig 5E–5K). The very deep tracks of style M5 are the focus of another detailed study and publication by these authors. Here, we describe and discuss the elongate tracks of styles M4 and M5. In these modes of preservation, the posterior impression extends from the heel impression toward the back without any change in depth of the print.

The morphology of the posterior wide extension is variable. In some, the length of the posterior extension is like the length of the metatarsal print (Fig 15A and 15B). These are similar to the semiplatigrade footprints reported by Pérez-Lorente [40] at the El Villar-Poyales Icnitas 3 tracksite and Las Losas tracksite, La Rioja, Spain. Others show a much shorter posterior impression with a round or an acuminate termination (Fig 15C–15E).

Their length is 5–12 cm and can be straight, oriented outwardly (Fig 15A, 15B, 15F and 15G), or inwardly (Fig 15C–15E). The acropodium impression is not reduced compared to what was reported in other cases [e.g., 107]. As seen on the surface of the trackbearing layer, the metatarsal mark is not deeper than the digit impressions; on the contrary, the shallowness is uniform in all the metatarsal impressions observed, indicating that the animal’s autopodium did not sink deep into the substrate (compared with Xing et al. [102], where the metatarsal mark is shallow posteriorly but gradually slopes anteriorly).

A remarkable case is track T22-2–354, found at the edge of site CP8, where the trackbearing layer is exposed in plain view and cross-section (Fig 15F). The track shows the filling sediment, including an enigmatic long posterior extension oriented toward the side of digit IV. In contrast to all others found at the Carreras Pampa tracksite, this posterior extension is very narrow and sinuous.

We do not interpret all the Carreras Pampa elongate tracks as “penetrative tracks” or “collapsed prints,” following the terminology of Falkingham et al. [94], Romano and Whyte [114] and Lallensack et al. [107], which commonly correlated with low anatomical fidelity [107]. The Carreras Pampa tracksite has trackways with very deep (decimeter-scale) tracks with posterior extensions and low anatomical details. Those deep tracks can be interpreted as penetrative tracks that remained deep and preserved as such after lifting the foot, i.e., without the collapse of the walls into the shaft.

Typically, collapsed tracks exhibit narrow digits and metapodium impressions due to the sediment falling into the impression left by the autopodium on the substrate [115]. In Carreras Pampa, the morphology of the metatarsophalangeal and digit prints of the tracks with wide posterior extensions resembles the “normal” walking tracks in the tracksite without the posterior mark. Neither the prints nor the elongated impressions show evidence of sediment collapse either during the descent of the digits or during the kick-off phase of the movement. Their digit impressions are well-marked, including details of the claw traces (depth, shape, and orientation), and there is no inner mound of sediment that could have formed after the autopodium sank into the sediment and was lifted. We would not expect to observe claw traces in these tracks with elongated posterior extensions if they resulted from deep penetration and subsequent lifting. Moreover, some of these elongated tracks have noticeable hallux marks, a feature commonly lacking in elongated tracks elsewhere. They do not exhibit an expulsion rim around the posterior extension or in the digit area, which would have formed as a result of sliding or pushing the sediment upward, forward, or laterally. The manus impression is absent [cf. 103]. These tracks with metatarsal impressions occur interspersed with digitigrade ones in the same trackway, a feature also seen at the Las Losas tracksite in La Rioja, Spain [40]. They appear in trackways with a regular, uniform pace between tracks, which indicates that the animal did not slow down or crouch during the formation of the posterior extension.

The similitude of pace, stride, and overall morphology between the tracks with wide posterior extensions and those without them is striking. We propose that the deep elongate tracks are penetrative as defined Lallensack et al. [107], but the origin of the shallow elongate tracks remains enigmatic, deserving further study.

### Abundance, distribution, and density of tracks

In this study, we report 16600 tracks across all sites in the Carreras Pampa tracksite (Tables 1 and 2), making it the largest dinosaur tracksite in the world and, more specifically, the largest tracksite featuring theropod tracks. The continuous areas of sites CP1, CP2, and CP3 also include a record number of prints, totaling 12376. In this count, we do not include solitary tracks from sites CP1, CP5, and CP7, nor the abundant tracks of preservation style M1 present at all sites, which consist of monodactyl, didactyl, or tridactyl indentations on the tracking surface. We estimate that if we were to include these unaccounted tracks, the total number would rise to nearly twenty thousand.

Table 1 shows the calculated track density for each studied site, ranging from 0.6 to 3.76 tracks/m². The overall density across the entire tracksite is 2.22 tracks/m^2^. The track density varies across the tracksite. Site CP7 has a low density of tracks because much of its surface has been eroded or altered. Without the count for site CP7, the total density figure for sites CP1-CP6, and CP8 and CP9 is 2.61 tracks/m^2^.

Three reasons may explain the variation in track density: 1) the concentration of the trackmaker’s activity in certain areas more than others; 2) the partial or total erosion of the upper tracked surface in some sectors of the studied sites; and 3) modification of the tracked surface due to bioerosion from plant roots. Moreover, track density could have been higher had we counted the tracks of preservation style M1 in all sites and the solitary tracks in sites CP1, CP5, and CP7. Higher density values indicate the sites where solitary tracks were counted. Each site has small extents where the tracked surface has been eroded or heavily impacted by bioerosion from plant roots, resulting in the disappearance of many tracks. The approximate areas of these eroded sectors are 50 m² on the NW side of site CP1, 115 m² on the north side of site CP2, 17 m² in the middle of site CP3, 10 m² on the north side of site CP4, 23 m² on the north side of site CP5, 17 m² on the north side of site CP6, a significant area of about 200 m² on the south side of site CP7, and 20 m² on the north side of site CP9 (S1 Fig). There are some small areas where the traces left by bioerosion from plant roots are so numerous that it is impossible to distinguish any footprints, and the trackways across those areas are discontinuous. These sectors with bioerosion from plant roots are significant in size on the north side of site CP5 and the south side of site CP7, which might explain the relatively low density values for those two sites (1.78 tracks/m² and 0.60 tracks/m², respectively). A significant area with bioerosion from plant roots also occurs on site CP9; thus, that site’s density could be considerably higher than the estimated value of 3.52 tracks/m². The density of the three small areas we selected on site CP1 and site CP2 to calculate the track density was much higher, possibly corresponding to regions of greater trackmaker activity. Track density was 5.74 tracks/m² in the 2.7x2 m area and 5 tracks/m² in the 4x4 m area, both on site CP1, and 4.40 tracks/m² in the 10x10 m area on site CP2. In this calculation, we did not include the tracks for preservation style M1.

Most studies on vertebrate tracks include a count of the total number of footprints found at the tracksite, but they often skip calculating the density of tracks per square meter. An exception is the summary study of the most significant tracksites in La Rioja, Spain, published by Pérez-Lorente [40], which includes an estimate of the track density in fourteen sites. The calculated densities range 0.07–0.9 tracks/m^2^. The assessment by those authors is “low” density for values 0.1 − 0.5 tracks/m^2^, “intermediate” for 0.9 tracks/m^2^, and “relatively high” for 0.9 tracks/m^2^ and “almost 1” densities. An estimate of track density in the published reports suggests that most tracksites in other parts of the world have densities of <1–2 tracks/m^2^. Compared with those values, the track density at Carreras Pampa is exceptionally high. Densities above five footprints per square meter are extremely rare in the known track record. Gaston et al. [116] reported in sandstone slabs from the Chinle Group of Colorado, USA, one of the highest concentrations of vertebrate tracks known to date: one slab has an estimate of 75 tracks/m^2^, including small *Grallator* and *Rhynchosauroides* footprints; the density of tracks in other slabs ranges from 26 to 40 tracks/m^2^. Jiang et al. [117] reported a density of 120 tracks/m^2^ of miniature theropod tracks in a slab of rock of 0.35 m^2^. Thus, apart from tracksites with very small (e.g., *Rhynchosauroides, Grallator*) and miniature tracks, the Carreras Pampa tracksite may have one of the highest theropod track densities known worldwide.

### Dimensions of the tracks

It is common to find theropod tracks of different sizes and morphologies preserved at the same tracksite [118,119]. At Carreras Pampa, in addition to multiple morphotypes, there are various footprint sizes. Here, we follow the footprint size classification proposed by Marty [41], with the footprint length (FL) categories of diminutive (or miniature, < 10 cm), small (FL = 10 − 20 cm), medium (FL = 20 − 30 cm) and large (FL > 30 cm) (Fig 3E).

Fig 16 illustrates the percentages of track sizes (FL) across different study sites and the combined tracksite. Medium-sized tracks account for over 50% of all tracks at all sites, with a strong representation in sites CP7 (70%) and CP9 (73%). The five sites with the most track size data are CP1, CP2, CP3, CP4, CP6, and CP9. In site CP1, 85% of the tracks fall within the range of 15 − 28 cm; in site CP2, 70% fall within the range of 16 − 29 cm; in site CP4, 78% fall within the range of 18 − 31 cm; in site CP6, 88% fall within the range of 14 − 26 cm, and in site CP9, 74% fall within the range of 20 − 28 cm. Across all sites combined, 82% of the measured tracks (n = 1146) have a foot length ranging from 16 to 29 cm, representing small and medium sizes, with small tracks being close to the medium range. The mean is 22.66 cm. Overall, there is minimal representation of large (>30 cm) footprints, with exceptions for sites CP5 (21%) and CP7 (23%).

The large number of tracks (1146) in the histogram of all tracks in Fig 16 does not follow a normal distribution, indicating that five modes of track lengths may be present with distributions surrounding them. In order of decreasing abundance, modes are suggested at 19–20, 22–23, 26–27, 12–13, and 34–36 cm within the range of 2–41 cm.

### Differences in mesaxony and digit length

The metatarsophalangeal and heel areas are well-marked in preservation styles M4 and M5 but poorly marked in styles M1–M3. Digit III is typically curved inward toward the trackway midline, which is a prominent feature in morphotypes T1, T2, T4, T7, and T8. Digits II and IV are oriented anteriorly in morphotypes T1, T2, T3, and T6, semi-laterally in morphotypes T2, T4, T5, T7, T8, and T9, and laterally in morphotype T2. Digit III is more deeply impressed than the others, often leaving a ridge of raised sediment (expulsion rim) in the hypex area. The tracks of morphotypes T1–T5 are strongly mesaxonic; that is, the impression of digit III is more prolonged than that of digits II and IV. In contrast, the tracks of morphotypes T6 and T7 show weak mesaxony. In most tracks, digit II exhibits more elongation than digit IV (e.g., T22-2–8, T22-2–3, T22-2–19, T22-2–21). However, there are trackways where the right tracks have digit IV shorter than digit II, while the left tracks preserve digit II shorter than digit IV. In some trackways (e.g., T22-2–49), both right and left tracks have digit II longer than digit IV (S5 and S6 Figs).

In most tracks, there is an “inner asymmetry” in which the relative lengths of the digits are III > II > IV, which is also found in other theropod ichnospecies such as *Majungasaurus crenatissimus* [120] and *Farlowichnus rapidus* [121]. Tracks of morphotypes T2, T3, T4, T5, and T6 may show a digit scale of length III > II ≅ IV, and very rarely III > IV > II. The asymmetry evokes the classical theropod shapes, and it is attributed in part to the presence of a notch at the base of digit II or digit IV [67].

### Total divarication

The interdigital angle or total divarication (αII–IV) is measured between the axes of the outermost toes (digits II and IV) (Fig 3B). Fig 17 represents the total divarication angles for all the sites at Carreras Pampa and the value for the combined sites. The five sites with the most accounted total divarication angles are CP1, CP2, CP4, CP6, and CP9. In site CP1, 73% (n = 279) of the tracks are in the range of 55° − 85°; in site CP2, 92% (n = 148) of the tracks are in the range of 30° − 70°; in site CP4, 80% (n = 95) of the tracks are in the range of 40^°^ − 65^°^; in site CP6, 81% (n = 118) of the tracks are in the range of 45° − 70°, and in site CP9, 60% (n = 93) of the tracks are in the range of 55° − 70°. For all the sites combined, 60% (n = 900) of the tracks exhibit a total divarication of 45°–70°.

The typical range for total divarication in theropod tracks is between 50° and 100°, depending on the size, species, and gait of the trackmaker. Total divarication may vary considerably within the same tracksite. For example, the reported total divarication for small, well-preserved theropod tracks in three tracksites in the Upper Cretaceous Seoyu-ri tracksite, South Korea, is between 33° and 104° for site L1, 46.5° and 84° for site L2, and between 39^°^ and 93.7^°^ for site L5 [122], and total divarication for small to medium theropod tracks in the Xiyangmugou tracksite in China ranges from 12° to 86° [123]. A review of the published values of total divarication shows a typical divarication of 40°–70° for small tracks (FL < 20 cm) and 30°–60° for medium and large theropods (FL 20–40 cm) [39], Some exceptions are known; for example, the total divarication for eight small theropod tracks (FL = 12.3–18.5 cm) from the Houcheng Formation, Hebei Province, China, is 16.7°–42.5° [124], and for small theropod tracks in the Upper Cretaceous of a quarry in Seoyu-ri, Hwasun County, Jeollanam-do, South Korea, is about 33^o^ [122]. Thus, with some exceptions, small theropods often have relatively high divarication angles compared to larger ones, and medium theropods exhibit moderate divarication, with some overlap in range with both small and large theropods. Greater divarication may suggest agility or adaptations for rapid turns and movement. For example, *Grallator* tracks, often associated with small, agile theropods, frequently show a total divarication in the 50°–70° range [46]. Lower angles (closer to 30°) might indicate a more linear, weight-bearing gait suited for larger, more stable animals. Medium-sized theropod tracks (FL 20–30 cm), such as those of *Hispanosauropus* (potentially allosaurids or megalosaurids) from the Upper Jurassic to the Lower Cretaceous Iberian Peninsula, exhibit divarication angles that range ~40°–50° [125]. Large theropod tracks like *Therangospodus* have narrow divarication angles (~30°–40°).

Several factors may influence the divarication of theropod footprints:

1) Species and foot morphology: Different theropod species had varying foot shapes, influencing divarication. Agile, gracile forms (e.g., small theropods) typically show higher divarication. Tracks of the same individual may show different divarication angles. For instance, the digit divarication shows some variation in the Upper Jurassic Langenberg large footprints, varying from 43° to 73° [126].
2) Substrate conditions: The consistency of the sediment is of primary importance. Field and experimental observations show that the most significant factor controlling track morphology is the moisture/density relationship within the substrate at the time of track formation [89]. Soft or wet substrate can spread the toes outward, artificially increasing divarication angles, and thus, digit divarication can vary with substrate conditions [127,128]. The divarication angle of modern emu tracks is very inconsistent, ranging from 61° to 102° in only 30 footprints of a single individual [129]. Digit divarication can also vary with the depth within the same trackway [129].
3) Speed and behavior: Running or rapid, random movement can increase toe splay, while steady walking or running tends to minimize divarication. Narrow (lower) divarication angles (25°–40°) are associated with longer strides and may indicate a stable and more energy-efficient linear gait, enabling greater speed and energy conservation over long distances. Moderate divarication (40°–60°) is associated with intermediate stride lengths typical of medium-sized theropods capable of versatile locomotion and suggests a balance between stability and agility. Wide (higher) divarication (60°–70° or more) is associated with shorter strides typical of smaller or more agile theropods. It may indicate adaptations for sharp turns or bursts of speed, often linked to more active predation or navigating uneven terrain.
4) Foot-substrate interaction: the total divarication also depends on how deeply the feet sink into the substrate. It has been demonstrated (experiments, computer simulations, and field evidence) that in many cases, a penetrative track shows a much larger divarication (often bird-like) than the foot that made it. In deep, penetrative dinosaur tracks, total divarication—the angle between the outer digits (e.g., toes II and IV)—can significantly increase compared to shallower prints. This occurs because the toes spread out as the foot sinks deeper into soft, unstable sediment. The resulting track often appears broader and less anatomically defined than a track left by the same animal in firm ground. As the dinosaur’s foot sinks deeply into the ground, the toes actively spread to create a wider base for stability [130,131]. This behavior exaggerates the natural angle of the foot.
5) Sediment collapse and flow: The fluid dynamics of the sediment can further exaggerate the track’s divarication. As the foot is withdrawn, sediment can collapse and flow inward, distorting the original print. In some cases, the sediment may also seal up behind the foot, obscuring anatomical detail but often creating a wide, distorted shape at the surface [131].

In our study, correlations among PANG, II-IV, PL, SL, FL, and WAP were explored. Neither the II-IV angle nor PANG explained more than 12% of the foot length, pace length, stride length, or track width. There is a poor negative correlation between total divarication and PANG and stride length, a negligible correlation between total divarication and pace length, and a poor positive correlation between total divarication and foot length (FL) for all sites.

### The hypeces between digits II–III and III–IV

In theropod tracks, the hypex is the point of convergence between the impressions of the digits on a footprint. Specifically, it is the V-shaped notch where the bases of digits II and III, and III and IV meet. Fundamentally, the morphology and size of the hypeces depend on the anatomy of the autopodium: the hypex corresponds to where the metatarsals articulate with the phalanges. For descriptive purposes, the areas where digits II and II, and III and IV converge are called hypex areas.

At the Carreras Pampa tracksite, the hypex areas in digits II–III and III-IV vary considerably, even along the same trackway; the reasons for this may be due to the differences among trackmakers, the distinct conditions of the substrate, and/or the gait of the trackmaker. This phenomenon has been observed in theropod trackways in other tracksites [e.g., 132]. Morphotypes T1, T2, T4, and T10 have a narrow hypex area with interdigital angles 23°–33°, rarely below 23° or above 33°. Commonly, the hypex area between digits II and III is narrower than between digits III and IV. The hypex areas are broad in morphotypes T3, T4, and T9, where the interdigital angles range from 30° to 54°.

In ichnites, the depth and shape of the hypex area can vary based on 1) the size and type of theropod trackmaker; 2) the substrate consistency (soft vs. hard ground); and 3) the preservation quality of the footprint. The morphology of the hypeces has taxonomic significance and thus variations in hypex area morphology (e.g., angle and depth) are sometimes used to distinguish between different ichnogenera such as *Grallator* (sharp, narrow and acute hypex), *Eubrontes* and *Anomoepus* (broader and often deeper hypex), and *Anchisauripus* (intermediate in terms of angle and depth between the two) [133–136], although this use has been questioned [137]. The hypex areas can be symmetrical, where the angles formed by the digits relative to the hypex are nearly equal, possibly reflecting a well-balanced gait, or they can be asymmetrical, where one toe (usually digit IV) diverges more than the others, indicating either a turning motion or an anatomical preference for weight distribution. The morphology of the hypex areas may have functional implications. A deeper hypex area may indicate a strong, flexed foot capable of gripping softer substrates. A shallower hypex area may reflect walking on firmer ground with less toe flexion.

### Foot rotation

Foot rotation in theropod tracks describes how the deviation of the footprint’s orientation, particularly the middle digit III, deviates from the direction of the dinosaur’s travel (Fig 3D). Foot rotation can be inward, outward, or neutral. Some theropod tracks exhibit slight inward rotation, where the toes point toward the midline of the trackway. Inward rotation indicates a narrower stance and is often associated with efficient, bird-like locomotion. Outward rotation is less common; however, some theropods display outward foot rotation, possibly due to wide-gait walking or maneuvering on unstable ground. In neutral rotation, tracks show little to no deviation from the direction of travel, indicating straight-line walking with minimal rotation.

Typically, in well-preserved trackways, theropod footprints often show minimal inward or outward rotation of 0°–5° [sensu 38, 138], with the middle digit (digit III) aligned roughly parallel to the direction of travel. A straight alignment suggests efficient, bipedal locomotion with little lateral movement. Tracks occasionally exhibit moderate rotation (15°–30°), which could be caused by turning or maneuvering, substrate slippage during locomotion, and the biomechanics of the hips and legs during specific gaits (e.g., running, injury).

Foot rotation may be highly variable within the same tracksite and even within the same trackway. For example, Lallensack et al. [139] reported from the Lower Cretaceous of Münchehagen, Lower Saxony, Germany, a very weak foot rotation, close to 0°, in a theropod trackway with medium-sized tracks, and a pronounced inward rotation averaging 11.2° in another trackway. Romero-Molina et al. [140] found that 65.4% of the theropod trackways in the Aptian Las Losas ichnosites, Enciso, La Rioja, Spain, have an inward or 0° orientation, and 34.6% have an outward orientation, with small theropod footprints showing more outward rotation and large theropod footprints showing inward orientation. Day et al. [50] reported a strong inward rotation of approximately 10° in the theropod trackways of the Middle Jurassic Ardley tracksite, Oxfordshire, United Kingdom, noticing that the rotation is only slightly inward in a section of the trackway associated with a change in gait.

At Carreras Pampa, foot rotation exhibits a bimodal distribution (Fig 18), with most tracks showing inward rotation of the distal end of digit III print, prominently marked in tracks of morphotypes T1–T5 and T8. This effect resulted from the claw being forced inwards during the outward rotation of the foot as the autopodium withdrew from the substrate [95]. Fig 36 presents the histograms of foot rotation for sites CP1, CP2, CP4, and CP9, along with the aggregated data for these four locations. At site CP1, foot rotation is predominantly inward (mean = 0.82°, n = 770). Although there are significant differences in the types of rotation observed at these four sites, the number of tracks showing inward and outward rotation is nearly equal (outward, mean = 0.11°, n = 1939).

### Preservation of the hallux

Many tracks in the Carreras Pampa tracksite show the impression of the hallux, which is the impression of digit I. Illustrations of the preservation of the hallux are shown in Figs 5E, 5I, 11A, 12D, 12E, 12G, 19 and S6B Fig.

The first pedal digit (digit I) in theropod dinosaurs is the opposed hallux or reversed hallux. Reconstructions of the foot anatomy show the hallux elevated off the ground and positioned higher on the metatarsus. This configuration is referred to as a functionally digitigrade stance, where the hallux typically does not make contact with the ground during normal walking or running. Therefore, its preservation in theropod footprints is relatively rare. Nevertheless, in some theropods, the hallux was larger or positioned closer to the ground, leaving a trace on the posterior-medial side of the print of digit II, thus creating a tetradactyl footprint. Fig 30 shows the variation in the morphology of the halluces found in the studied site. We distinguish three morphotypes of the hallux.

#### Hallux morphotype H1.

These traces are puncture-like markings measuring 1–3 cm in diameter, occasionally up to 4 cm, and 0.3–1 cm in depth (Figs 5I, 11A, 12D, 12G and 19A–19C). These indentations may be conical or irregular with several corners. The occurrence of an expulsion rim is rare. The distance from the track measures 2–4 cm, though they can be adjacent to the edge of the track (e.g., Fig 19A).

#### Hallux morphotype H2.

These traces are narrow, elongated, curved, or almond-shaped (Fig 19D–19F). They measure 4–5 cm in length, are not as deep as the hallux traces of morphotype H1, and are 0.5–1.5 cm thick, with the proximal end thicker than the distal end. When they possess an expulsion rim, it is thicker at the proximal end than at the distal end. Usually, they are found 2–3 cm from the edge of the track. This is the most abundant hallux morphotype in the Carreras Pampa study sites.

#### Hallux morphotype H3.

The halluces of this type are curved or sinuous and are attached to the edge of the track or form an extension of the latero-posterior side of the print (Figs 5E, 5I and 19G–19I). They are the longest and thickest of the three types of hallux impressions, with an inner length ranging from 4 to 11 cm and an inner width of approximately 2 cm. Some of them feature a thicker expulsion rim on the posterior side. This morphotype is rare at the studied sites.

#### Significance of the hallux preservation.

Theropod tracks with the impression of the hallux have been found in many tracksites worldwide [57,104,105,107,126,141–155]. The reason some tracks retain the hallux impression while others do not is unclear. The common assumption is that their presence is linked to very deep tracks. Xing et al. [156] asserted that only a small proportion of known theropod prints have associated hallux traces preserved, and that these traces typically did not register on the substrate unless the tracks were very deep. Hallux impressions occurring only in the deepest impressions have been reported in Jurassic theropod tracks in southern Italy [99] and in the Upper Jurassic Lastres Formation in Asturias, Spain [152]. However, the exclusive presence of hallux impressions in very deep tracks at those two locations could be due to the small size of the exposures.

Lockley et al. [157, p. 323] assert that “The depth of the tracks, on the order of 10–15 cm, presumably accounts for the preservation of hallux traces” in *Eutynichnium* theropod tracks from the Upper Jurassic of Portugal, and Lockley et al. [155] suggested that “the hallux may register more completely and often when tracks are deep and not when they are shallow.” However, halluces have been reported in relatively shallow tracks in other tracksites [e.g., 158].

In the Carreras Pampa tracksite, many trackways have preserved hallux impressions in very deep, deep, and shallow tracks. For example, one trackway of 82 tracks contains 79 tracks with halluces preserved. They are most common in shallow to deep tracks of the preservation styles M4 and M5. Trackway CP6–9 features three hallux impressions: one in a very deep track and the other two in deep tracks. Many very deep tracks in the M5 preservation style do not show a trace of the hallux; others display a side impression similar to the impression of digit II (Fig 7E, and the black arrow in Fig 6E). In the Carreras Pampa trackways, the hallux is located relatively low (i.e., distally) on the metatarsophalangeal impression, indicating that the trackmaker’s hallux was large. These tracks closely resemble the deep tracks with metatarsal impressions at the Paluxy River tracksite (Lower Cretaceous Glen Rose Formation), Texas [100, 107, Fig 1F].

Gatesy et al. [159] speculated that a reversed hallux impression is simply the trace left by a hallux that was slightly abducted and flexed but not reversed. The consistent posteromedial orientation and the similar location of the hallux impression in all the trackways at Carreras Pampa, including both shallow, deep, and very deep tracks, support the idea that digit I was anatomically low in the limb of the trackmakers, allowing for easy contact with the substrate upon flexion of the metatarsal joints [see 95, Fig 8].

Fig 19 shows that the impression of the hallux is positioned posteromedially relative to the impression of the metatarsophalangeal area, with some variability in the distance from the heel and the track’s rim. In most cases, the broader impression of the hallux is oriented toward the rim of the track, while the slimmer part faces away from it. In a few instances, the hallux impression runs parallel to the rim of the track. None of these impressions indicate that the hallux had a fully reversed anatomical orientation, as seen in many extant birds. Additionally, the relative variability of the hallux impression’s position in relation to the other digits suggests that digit I did not contribute significantly to locomotion or stability [160]. The hallux was likely slightly abducted and flexed but not reversed, making transient contact with the substrate.

### Tracks with straight posterior or anterior ridges or grooves

A few tracks in Carreras Pampa display a posterior straight ridge or a groove, while others exhibit an anterior groove. Some of these tracks are illustrated in Figs 12H, 20 and S6 Fig.

Several tracks have an anterior groove extending from the tip of digit III in tracks of the styles of preservation M3 and M4. Most of these anterior grooves are approximately 10 cm long and straight (Figs 11A and 20G); in one instance, it is around 15 cm long. Compared to tail-drag marks found in other trackways, these grooves are not curved inwardly or strongly sinuous, but are primarily straight or slightly curved inward, very short, and extend directly from the tip of the third toe.

We interpret the anterior grooves as the result of dragging the claw of the third toe forward after the kick-off phase of the autopodium’s movement. In contrast, the posterior grooves mark the same toe as it approaches the ground again.

Several cases with similar potential have been reported in the literature. Gow and Latimer reported one short drag mark in one undescribed tridactyl trackway in the Qwa Qwa tracksite, South Africa (Triassic Molteno Formation), which they claimed was “likely made by the middle toe of a partially raised foot” [161]. However, they did not illustrate this feature, and thus, we cannot compare it with our findings. Lockley et al. [162] reported three parallel toe drag traces 2–3 m apart associated with tracks of ornithopod affinity in the Cretaceous Escalante Rim tracksite, western Colorado. The authors provided only a sketch drawing of the marks and no dimensions or other physical characteristics. Meyer and Thüring [142] reported “a medial posterior groove” in some trackways found in the Middle Jurassic El Mers Formation, Middle Atlas Mountains, Morocco.

The linear grooves and ridges at Carreras Pampa resemble markings observed in modern birds walking on soft substrates like wet sand, soft mud, or snow (our observations). Typically, these markings manifest in several patterns: 1) a long groove (1x–2x the length of the track) from the third toe with either a straight or slightly curved shape, 2) a short or long groove (up to 3x the length of the track) that runs straight on the posterior side to the trace of digit I (hallux). In some trackways, the groove connects two successive tracks; however, the grooves in Carreras Pampa’s theropod trackways do not connect two consecutive tracks.

The posterior grooves occur in shallow tracks of the style of preservation M3, deep tracks of the style M4, or very deep tracks of the style M5. The width of the ridge is approximately 1 cm, and the length ranges from 13 cm to 50 cm. It starts gradually and ends abruptly at the heel impression, indicating that the printing of the autopodium’s contact with the ground cut the ridge. Almost all the tracks of the trackway T22-72 exhibit a posterior ridge. The ridge typically has a lee (crest) and a stoss (trough) side, corresponding to the low-angle and high-angle slopes. Fig 20B illustrates a particularly long (55 cm) ridge in track R8 of the trackway T22-121. The anterior part (a) near the heel impression reveals the ridge structure, which consists of two parallel ridges separated by a 1.5–2 cm wide U-shaped groove that narrows proximally. The posterior part (b) tapers off to become flat.

The posterior grooves are puzzling. They might result from the closure or collapse of the metatarsal impression in penetrative tracks, as defined by Lallensack et al. [107]. However, this interpretation may conflict with several pieces of evidence. First, the grooves are consistently straight and uniform in size, except for the very posterior end, which tapers to a point, while the metatarsal impressions in deep penetrative tracks show morphological variability. Second, the length of the grooves does not match the length of the preserved tracks; some grooves are shorter than the tracks, others are the same length, and some are twice as long (Fig 20). Third, there is a clear boundary between the well-printed tracks that show high anatomical accuracy and the straight ridges and grooves, which would not be expected if the linear structures resulted from uplifted toes in penetrative tracks. Therefore, although we recognize that the origin of these eleongate tracks is enigmatic, we prefer the interpretation that these ridges and grooves are marks left by dragging the third toe during normal gait when the trackmaker withdrew its toes from the substrate, trailing the claws forward through the rim of the footprint [38]. In some ridges, the trough is deeper than the surrounding area, indicating that the toe created a groove whose walls then collapsed into the narrow trough, pushing sediment out to one side.

A detailed description and model for the formation of the posterior ridges is in preparation by several of the authors.

## Descriptions of trackways

The trackways comprise successive tridactyl tracks made by theropod trackmakers. Aside from the trackways in the northernmost area of site CP2 and small patches of weathered, tracked surfaces across various sites, most trackways in the Carreras Pampa tracksite are well-preserved, allowing for the measurement of pace length, stride length, and other dimensions. These measurements enable estimates of hip height calculations and locomotion speed. Identifying tracks formed by the left and right foot (siding) was possible in all the exposed and studied trackways. The siding was clear and unambiguous throughout all the trackways. With few exceptions, they are narrow-gauge trackways (see below). The trackways are illustrated in Figs 6, 7, 21–29 and S9 Fig.

Some trackways contain compound prints, which are tracks characterized by different morphologies resulting from different behaviors of a single producer [163–167]. Some trackways contain tracks of various preservation styles and more than one morphotype.

### Abundance and length of the trackways

Within the exposed CP sites, 1321 trackways of two or more successive tracks were found (Table 1). Some of the trackways oriented roughly E-W could be traced across sites CP1, CP2, CP5, and CP8, while some trackways oriented roughly N-S could be traced across sites CP1, CP2, CP3, and CP8. If linked between sites, this would reduce the total number of trackways and increase the average trackway length. The contiguous areas of CP1, CP2, and CP3 (Fig 1) contain 864 exposed trackways. Sites CP1 and CP2 have longer trackways because their shape is roughly oriented N-S, matching the orientation of most of the trackways (see below).

Of the 1321 total trackways identified, 46 were marked, but no measurements were taken. For the remaining 1275 (Table 2), the longest trackways are in sites CP1 and CP2 and correspond to trackways T22-021 (95 m, 99 tracks), T22-2–025 (94.59 m, 103 tracks), T22-2–248 (90 m, 34 tracks), T22-2–007 (88.45 m, 98 tracks) and T22-2–446 (86.31 m, 88 tracks). The total accumulated length of all the study site trackways is 14311 m.

**Table 2:** Number of trackways in each CP site categorized by length. Of the 1321 total trackways identified, 46 were marked, but no measurements were taken.

Site	Length (m)									
	<9.99	10-19.99	20-29.99	30-39.99	40-49.99	50-59.99	60-69.99	70-79.99	80-89.99	≥90
CP1	187	43	18	18	23	3	1	1	0	1
CP2	289	56	24	13	14	7	0	2	2	2
CP3	68	24	2	1	0	0	0	0	0	0
CP4	120	13	0	0	0	0	0	0	0	0
CP5	51	16	4	0	0	0	0	0	0	0
CP6	63	11	13	2	0	0	0	0	0	0
CP7	64	18	0	0	0	0	0	0	0	0
CP8	13	0	0	0	0	0	0	0	0	0
CP9	65	19	2	2	0	0	0	0	0	0

### Orientations of the trackways

The orientation of theropod trackways can provide insights into their behavior, movement patterns, and environmental context. The CP sites exhibit groups of trackway orientations, predominantly located within a bimodal distribution of NNW-NNE (300°–30°) and SSE (120°–185°), with a few trackways oriented E (75°–105°) and W (240°–285°) (Fig 30A). A similar pattern is observed when the orientation is plotted for each site (Fig 30C–30J). Site CP6 displays a more dispersed distribution of trackway orientations, yet retains a predominant NW/SE bimodal orientation. Dimensions and orientations of trackways and tracks are provided in the Supporting Information (S1 Table).

#### Other bimodal orientation descriptions.

A similar bimodal orientation was recorded for 101 theropod trackways in the El Molino Formation at the Km99 tracksite, which is located 4.3 km NE of Carreras Pampa [24]. The Cal Orcko tracksite in Sucre, Bolivia, also shows a similar bimodal orientation for 428 trackways. However, the bimodal orientation at the Cal Orcko tracksite is not as pronounced as at the Carreras Pampa tracksite [14]. Another similar orientation pattern was reported for 52 theropod trackways in the Campanian-Maastrichtian of Yeosu, South Korea, where the crestlines of wave ripples are WSW-ENE [168]. Day et al. [50] reported a similar trend in the Middle Jurassic Ardley tracksite in Oxfordshire, United Kingdom, for over 40 more-or-less continuous theropod and sauropod trackways, although the theropod trackways are all oriented in the NE direction. A bimodal orientation was observed in 236 Lower Jurassic *Eubrontes giganteus* track*s* by Getty et al. [169] at the Dinosaur Footprint Reservation in Holyoke, Massachusetts. Twenty-eight small trackways of medium-sized tridactyl tracks exhibit a SE-NW orientation pattern in the Upper Jurassic Courtedoux-Bois de Sylleux tracksite in Switzerland [170]. A strong NW-SE bimodal pattern was observed in 200 tracks and 35 trackways of *Anomoepus* in the Lower Jurassic Lisbon Valley Dinosaur Tracksite in Utah [171]. A strong NE-SW bimodal trend of trackway orientation was observed in 60 tracks comprising 17 trackways at the Desert Tortoise Tracksite 1 in the Lower Jurassic Kayenta Formation, Utah [172]. A remarkable NNE-SSW bimodal orientation pattern was reported for 88 theropod tracks at Namibia’s Lower Jurassic Waterberg tracksite [173]. Other examples of the bimodal orientation of theropod trackways are also known (e.g., [174–176]).

#### Significance of orientation.

Compared to other known tracksites worldwide, the bimodal pattern of trackway orientation at Carreras Pampa is noteworthy for several reasons. First, it is observed over a larger exposure than at other tracksites, indicating that the trackmakers traveled across an area at least 300 m wide (Fig 30B), with the movement directions predominantly NNW or SE throughout that width. Second, the number of trackways with bimodal orientation reaches into the hundreds, far exceeding other sites where such a pattern is only inferred for dozens or fewer trackways. The presence of multiple trackways heading in the same direction also shows that many trackways run parallel (Figs 21–25). Parallel trackways are important for interpreting behavior, as will be discussed below.

### Differences and similarities among track sites

Because the sites are closely spaced and measured tracks are all on the same surface, it seemed plausible that each site sampled a single population for all sites. To test their similarity, we compared the means of stride length (SL), pace length (PL), trackway width (WAP), foot length (FL), distal depth (DD), and proximal depth (PD) for each site to see if they differed. This approach was supported by the relatively large area and quantity of trackways and tracks at each site (Tables 1 and 2). An ANOVA was first performed to evaluate whether means differed among nine different locations, followed by a Tukey Honest Significant Difference (HSD) post-hoc test to assess pairwise differences between groups. To account for multiple comparisons and reduce the likelihood of false positives, the resulting p-values from the Tukey HSD test were adjusted using a False Discovery Rate (FDR) correction (*p* = 0.05).

After applying the FDR adjustment, none of the pairwise comparisons showed statistically significant differences in mean pace length, stride length, or trackway width. However, important differences among means from each site did occur for foot length, proximal depth, and distal depth. For example, mean foot lengths from the same nine sites revealed that 13 out of the 36 possible site pairs showed statistically significant differences in their site means after FDR (p = 0.05) adjustment. The first three measurements relate to marks along a trackway, and their measurements are less influenced by the shape of an individual track. Conversely, the last three measurements focus on details of individual tracks and are much more affected by slight differences in formation, preservation, or weathering. These statistical tests indicate that measurements from trackways represent the entire population and support conclusions based on the overall distribution of results. Results from individual tracks differ among sites, indicating that this measurement class was affected by very local conditions. Examples of pairwise comparison statistics are presented in Supporting information S10 Fig.

### Trackway measurements

#### Pace length (PL).

The mean pace length is 84 cm with one standard deviation about the mean falling between 64 and 114 cm (Fig 31). Pace length distributions are similar across all track sites (n = 806). Pace length is not quite normally distributed, even for the large number of samples. The distribution may be multimodal with dominant modes at 85–90, 75–80 and 65–70 cm and minor modes at 25–30, 125–135, and 150–155 cm. Complete data sets for PL and SL are in the S1 Table and illustrated in Figs 26 and 27.

The pace length in a theropod trackway refers to the distance between two consecutive footprints (e.g., from the right to the left footprint) along the line of progression of the trackway (Fig 3D). The stride length is the distance between successive footprints of the same foot (e.g., from the right to the following right footprint) (Fig 3D). These measurements infer the animal’s size, gait, and speed. A longer pace typically indicates a larger theropod or one that is moving more quickly. Comparisons between pace length and the size of the footprints can help determine the relative gait (e.g., walking or running). The size of the theropod is the main factor influencing the pace and stride length; larger theropods generally have longer paces, as their legs are proportionally longer. Other factors include: 1) speed – walking paces are shorter and more consistent while running trackways exhibit longer paces and larger spacing; 2) type of locomotion – theropods, being bipedal, produce a more linear trackway with less lateral displacement compared to quadrupeds; and 3) substrate and environment – soft or uneven ground might shorten pace due to instability, and wet or slippery conditions can also alter track spacing.

##### Stride length (SL).

The mean stride length across all sites (n = 831) is 165 cm, with one standard deviation around the mean ranging from 132 to 198 cm (Fig 32). The distribution slightly deviates from normal, likely due to the presence of multiple modes within the overall population. The total stride length (SL) range spans 80–260 cm. Trackways with short (<65 cm) or very long (>100 cm) stride lengths are few but noteworthy, as they reflect the extremes of small trackmaker sizes or slow speeds, and large trackmaker stride lengths or running stride lengths, respectively. Sites CP1, CP2, CP7, and CP9 feature significant trackways exceeding 2 m in stride length, including a few up to 2.6 meters, indicating groups of large-sized trackmakers or fast-moving animals.

Stride length generally increases with theropod size and locomotion speed. The walking stride is approximately 4–6 times the foot length, while the running stride can exceed 6 times the foot length, depending on the speed. For example, the small and medium *Hispanosauropus* tracks from the Upper Jurassic to the Lower Cretaceous Iberian Peninsula have a walking stride length of approximately 150 cm and a running stride length of roughly 250 cm [125]. For walking, stride length/hip height is ~ 2.0 or less; for running, stride length/hip height is > 2.0. At Carreras Pampa, trackways show a positive correlation between foot length (FL) and stride length (SL) (Fig 33).

One pair of measurements was compared to determine a correlation between individual tracks (foot length) and their distribution along a trackway (pace length). These measurements were analyzed for each of the nine sites, as there were significant differences among the site means for foot length. All comparisons demonstrated a positive correlation (R) ranging from 0.15 in sites with few measurements for both values to 0.8.

##### Trackway width (WAP) and pes pace angulation (PANG).

The width of the angulation pattern or trackway width (WAP) is measured perpendicular to the stride length, and the trackway pace angulation (PANG) is the angle between the right and left footprints in a bipedal or quadrupedal trackway, measured where the footprints form a sequence of three successive tracks (Fig 3D).

Trackway width (WAP) and pace angulation (PANG) were measured in several trackways from each site. Our visual observations indicated that the width was not constant along the entire length of the trackway but varied by a few centimeters. This is also reflected in the PANG values in Figs 24 and 26–29. Measurements from all sites show a skewed range for WAP with a mode of 6–7 cm and a range from 0 to 50 cm. The histogram for all sites shows a significant predominance of narrow-gauge trackways and smaller modes at 7, 21, and 29 cm (Fig 34). A negligible correlation (R = 0.04) exists between WAP and SL for all sites (Fig 35).

Trackway pace angulation (PANG) was measured for a limited number of trackways at sites CP2, CP5, CP6, and CP9 (Figs 23, 24 and 26–29). The combined data (Fig 35) are not normally distributed and display a dominant mode between 165° and 170° (n = 108), along with a suggestion of a second mode between 150° and 155°. These two modes were most distinctly separated at site CP6. Collectively, these data indicate that slightly over half of the trackways exhibited a narrow gauge (165°–185°) gait, while marginally less than half displayed a wide gauge and more variable (130°–165°) gait. In CP2, we observed two trackways (2.6% of the total measured) with a PANG >180°, suggesting that the trackmaker walked with their feet crossing the trackway midline. Narrow-gauge trackways are illustrated in Figs 23 and 24, while wide-gauge trackways are depicted in Figs 24, 26 and 28. Some studied trackways display a transition from wide gauge to narrow gauge or vice versa (Figs 26–28), a characteristic commonly observed in the studied trackways. We also observed that long trackways show several shifts from one type of gauge to another.

Narrow-gauge pace angulation between 160° and 170° is most observed for theropods and indicates upright and efficient locomotion. A pace angulation below 160° indicates a broader stance [38,177–179]. Trackway width and pace angulation depend on several factors: 1) the anatomical structure of the trackmaker, with hip width and limb positioning influencing trackway angulation; 2) the speed of locomotion, as larger pace angulation may suggest faster movement, since trackmakers bring their legs closer to the body axis to maintain balance; and 3) local substrate conditions, where WAP and PANG may vary as the trackmaker adjusts its speed to softer or firmer substrates. Thus, whether low or high in terms of angle or width is a geometric adjective, and therefore, the criterion for distinguishing between narrow and wide-gauge trackways is not defined in most published studies. Commonly, trackways are categorized as either type without quantification [e.g., 61,180]. One exception is Day et al. [181], who describe three theropod trackways in the Ardley Quarry in Oxfordshire, UK, as wide-gauge having a PANG of 117°–132°, compared to the “more usual narrow-gauge form of theropod trackway in which pace-angulation values range between 160° and 170°” [181, p. 494]. We looked for a correlation between pace angulation and pace length but found only a very weak correlation (Fig 12C).

##### Change in PANG along the trackway.

Several trackways show a sudden change in the PANG, often linked to a shift in the direction of movement. Some trackways exhibit a section of deep tracks where the trackmaker attempted to adapt to the soft substrate and are occasionally associated with tail traces. An example is trackway CP6–32 (Fig 27), where the first two tracks are deep (style of preservation M5) with a broad PANG of 133° between R1 and R2, and 136° between tracks L1 and L2. Then, the PANG gradually widens to 174° in the subsequent shallow tracks of style M4. The PL in the narrow-gauge section averages 63 cm, while the PL in the wide-gauge section between L1 and R2 is 93 cm, indicating an increase of approximately 47%.

In trackway CP7-24-1 at site CP7 (Fig 28A–28C), the PANG widens from 117° to 151° to 169° in the trajectory between R1 and R3 (narrow-gauge to wide-gauge), with the very deep tracks made in soft substrate being L2, L3, and R3, and then narrows to 104° between R3 and R5 (wide-gauge) where the substrate was firmer. Interestingly, the trackway widens (wide-gauge) in the sector where the substrate was firm. Crossing the trajectory of this trackway is CP7-24-2, which has the opposite pattern of PANG, where a narrow-gauge (wide PANG) between L1 and R2 turns to wide-gauge (narrow PANG) between R2 and R3.

Trackway CP6–9 in site CP6 is wide-gauge but shows a change in PANG and PL in the area of soft substrate (Fig 29A–29D). From the first (R1) to the last exposed track (L5), the PL decreases by approximately 15%, and the pace angulation narrows from 134° (R1–R2) to 149° (R2–L3) and then gradually widens to 118°. Between R2 and L4, except for track R4, the tracks are very deep and exhibit a prominent expulsion rim on the outside of the track, a feature observed in very few trackways in Carreras Pampa. In this area, the animal entered a zone where the substrate was soft, causing its feet to sink deeply, leaving a displacement rim on the outer side. To maintain balance, the animal shortened its pace and reduced the pace angulation, resulting in shorter and broader steps. Tracks L3 and L4 have tail traces, which may have contributed to stability in the soft substrate. Three tracks (R1, R3, and R4) show the impression of the hallux, but only R3 is a very deep track (see below).

##### Relative stride length and speed.

Estimates of the speed of dinosaurs from the dimensions of the tracks and trackways are of interest in paleoichnology. Two authors have proposed formulas to determine the absolute velocity of theropods based on the ichnological evidence: Alexander [51] and Demathieu [182]. The most widely used formula to estimate the speed of theropods is Alexander’s, which uses the footprint length (to obtain the height at the hip) and stride length: *v(m/s) = 0.25 g*^*0.5*^ *SL*^*1.67*^*h*^−*1.17*^, where *g* is the acceleration due to gravity, *SL* is the stride length and *h*, the hip height (generally *h* = 4FL, where FL = footprint length). More recently, Ruiz and Torices [54] proposed an updated version of this formula: *v = 0.226 g 0.5 SL*^*1.67*^*h*^−*1.17*^. Several studies have used the formulas of both Alexander [51] and Ruiz and Torices [54] to estimate the speed of theropod dinosaurs [53,183–185]. The movement of dinosaurs has also been described in terms of gait. The gait of a trackmaker can be calculated by dividing the stride length by the estimated height at the hip (*SL*/*h*). This is also referred to as the relative stride length. Thulborn [38] proposed that the gaits of dinosaurs fell into three categories: walking (SL*/h* ≤ 2.0), trotting (2.0 < SL*/h* < 2.9), and running (SL*/h* ≥ 2.9). More recent studies have excluded the trotting category, and termed SL/*h* values of 2.0 to 2.9 as a “slow run” [186], or have termed all SL/*h* values over 2.0 as a running gait [187]. Bishop [188] argued that it is likely that theropod dinosaurs did not have discrete walking and running gaits, similar to modern birds. Thus, we use relative stride length values to group trackways without classifying them into distinct walking or running gaits. Elsewhere, we use the terms walking and running to refer to relatively slow-moving or quickly-moving trackmakers, respectively, without assigning specific relative stride length values to those terms. The Carreras Pampa tracksite, with its abundant trackways, offers an excellent opportunity to evaluate the gaits and speeds of theropod dinosaurs.

Data from multiple tracksites worldwide show that moving at low speeds with low relative stride lengths (SL*/h* ≤ 2.0) was the most common locomotion behavior in theropod dinosaurs. Overall, bipedal dinosaurs commonly used a relatively slow to moderate speed of 3.3 km/h to 7.0 km/h (0.91 m/s to 1.94 m/s) and only resorted to high speeds on rare occasions [24,46,48,53,63,171,189–208]. A few cases of fast-moving theropods are known from a few localities in China, England, Morocco, Massachusetts, and Utah (USA) [50,59,158,181,191,209–214].

In the Rio do Peixe Basin, Brazil, Leonardi [59] reported the speed of dinosaurs in 75 trackways, of which 58 trackways (78.67% of the sample) yielded a speed between 3 km/h and 7 km/h, with seven trackways (9.33%) showing a slower speed of ≤2 km/h (four of these sauropod trackways). Nine trackways (12%) indicate a speed between 8 km/h and 23 km/h. Five trackways (6.67%) have calculated speeds of 8–10 km/h; another four trackways (5.33%) are distributed over a range of 12.8–23 km/h. The conclusion is that the dinosaurs in the tracksites of the Rio do Peixe Basin generally moved with relative stride length values below 2.0 and only very rarely produced values over 2.9 [59, p. 172].

##### CP relative stride length and speed.

The gaits and speeds of trackways at the Carreras Pampa tracksite were calculated for trackways with two or more measured stride lengths (532 trackways) (Fig 37). Most trackmakers were moving slowly: 367 (69%) of the trackways had SL/*h* ratios below 2.0, with higher SL/*h* values being less common; 154 (29%) of the trackways had SL/*h* ratios of 2.0 < SL/*h* < 2.9, and 11 (2%) of the trackways had SL/*h* ratios over 2.9 (Fig 37A). The estimated speeds of the trackways ranged from 2.13 km/h to 21.30 km/h [51] or from 1.92 km/h to 19.26 km/h [54]. Most trackways had estimated speeds within 6–8 km/h [51] or 5.5–7.5 km/h [54] (Fig 37B and 37C).

##### Analysis of relative stride lengths and speed.

Studies have found that while humans have a distinct transition from walking to running, transitioning between the gaits of ground-dwelling birds is not as straightforward [188,215]. Bishop et al. [188] found evidence that theropod dinosaurs had a continuous locomotor repertoire similar to modern birds and argued that the gaits of theropod dinosaurs should not be grouped into discrete walking and running gaits. The distribution of relative stride length values at Carreras Pampa was unimodal with no modal divisions along the spectrum of values. Relative stride lengths of SL/*h* ~ 1.8 were the most common, with lower and higher values becoming less common with increasing distance from SL/*h* ~ 1.8. The distribution of estimated speeds of the trackmakers was also unimodal. These data support the conclusion of Bishop et al. [188] that theropods had continuous locomotor repertoires rather than discrete, discontinuous gaits.

Our data followed the general pattern for theropod trackways in that relative stride lengths below SL/*h* = 2.0 were the most common. However, a much higher percentage of the trackways at Carreras Pampa had values above SL/*h* = 2.0 than is commonly seen at other tracksites. The estimated speeds for the trackways were also higher than those typically found at other tracksites. When using Alexander’s formula [51], 312 (58.6%) of the trackways had an estimated speed of over 7 km/h, and 209 (39%) were over 7 km/h if using the formula of Ruiz and Torices [54] (Fig 37B and 37C). This represents a significantly higher percentage of trackways than Leonardi found [59], suggesting that the trackmakers at Carreras Pampa moved faster on average than at other tracksites. There is no apparent reason why the trackmakers at Carreras Pampa were moving faster than typically seen at other tracksites.

##### Sinuous patterns of walking.

Most trackways in Carreras Pampa are straight or slightly curved, as shown in Figs 21–28. However, numerous sinuous trackways deviate from a straight-line trajectory, indicating changes in the direction of locomotion (Fig 25). Trackways with multiple small turns conforming to a sinuous walking pattern are common at the tracksite. An example is trackway T22-2–21, which displays four turns in the preserved section: 10° to the west at R9, 10° to the north at L15 (with the walking direction straight north), 6° to the west at R17, another 6° further to the west at R21, back 12° to the north at L23, 7° to the north at L25, and 10° to the north at L35. On these curved paths, the stride length of footprints on the inner side of the turn decreases while it increases on the outer side. The angle of the footprints relative to the trackway axis changes along the curve, with the medial digit III slightly turned toward the new direction of movement. Footprints may show varying depths as weight shifts during turning or balancing. However, we did not observe any significant change in the depth of footprints at the turning points in Carreras Pampa.

Sinuous patterns have been observed in trackways of theropods [216], ornithopods [74,217], and are also known in sauropod trackways. [66,201,216–220]. Animals often take sinuous paths to a goal or destination, especially considering a long distance. Thus, it is likely that dinosaurs would have left meandering trackways, which are now not always observed because of the small surface area of the tracksites. In this context, the extensive exposure of the Carreras Pampa tracksite is a unique place to study the sinuous trackways of theropods.

Possible causes of sinuous trackways include 1) a locomotion behavior in which the dinosaur was avoiding obstacles, foraging, or navigating uneven terrain, 2) changes in substrate conditions, such as soft ground, and 3) social interactions in which a theropod might have been following a leader, avoiding predators, evading other individuals in the same group, or interacting with others, resulting in non-linear movement. The sedimentological characteristics of the tracked layer indicate that the tracksite was primarily a flat, even surface on which the trackmakers walked freely. The large number of trackways at the Carreras Pampa tracksite suggests many trackmakers were present simultaneously, leading to interactions that caused deviations in locomotion orientation. We observed that the trackways with deep tracks interspersed in the succession do not significantly modify the linearity of the trackways. Therefore, the rheological conditions of the substrate did not significantly affect the direction of locomotion.

##### Turns in trackways.

At Carreras Pampa, trackways with changes in movement direction are rare, and most deviations are minor. For example, trackway T22-2–497 is 3.22 m long, featuring four tracks of preservation style M2 and contains a turn of 22° in the direction of movement at R1 (S4D Fig).

McLarty and Esperante [21] described multiple trackways from Carreras Pampa that contained evidence of behaviors not commonly preserved in dinosaur trackways, including significant changes in the direction of movement; three trackways turned more than 45°, and a fourth turned 44^°^ (Fig 38). These trackways were found in sites CP1–CP4, with tracks ranging from miniature to medium.

Lockley et al. [221] found that although changes in trackway orientation of less than 45° are common, changes in orientation of over 45° are rare. Lockley et al. [221] described only 13 trackways with changes in orientation over 45°; theropods made six, sauropods made six, and an ornithopod made one. Weems [222] described and illustrated multiple theropod trackways from the Culpeper Quarry in Virginia, USA, but the orientation changes were not quantified. The tracksite map provided by Weems [222] shows multiple trackways with one or more turns, which appear to represent changes in orientation of more than 45°.

##### Stop or pause in the trackway.

Four CP trackways indicated that the trackmaker was walking, stopped or paused, placed both feet next to or near each other, and then began walking again (sites CP1, CP2, and CP4) (Fig 38C and 38D). One of these trackways also preserves evidence that the trackmaker was limping due to an injury on the right side. Stops and pauses were associated with changes in direction and within straight sections of trackways, and track size ranged from miniature to large. One trackway was found, following a straight path until it made an abrupt turn to the right. The trackway then gradually returned to almost the same orientation leading up to the abrupt change in direction. This path was interpreted as avoidance of some obstacle, possibly another dinosaur. One series of tracks and tail traces was interpreted as a possible crouching trace made by a theropod dinosaur. The multiple trackways at the Carreras Pampa tracksite, which indicate uncommon behavior preserved in the track record, are likely due to the large exposure area of the tracksite [223].

##### Tracks with tail traces.

**Fig 44:**
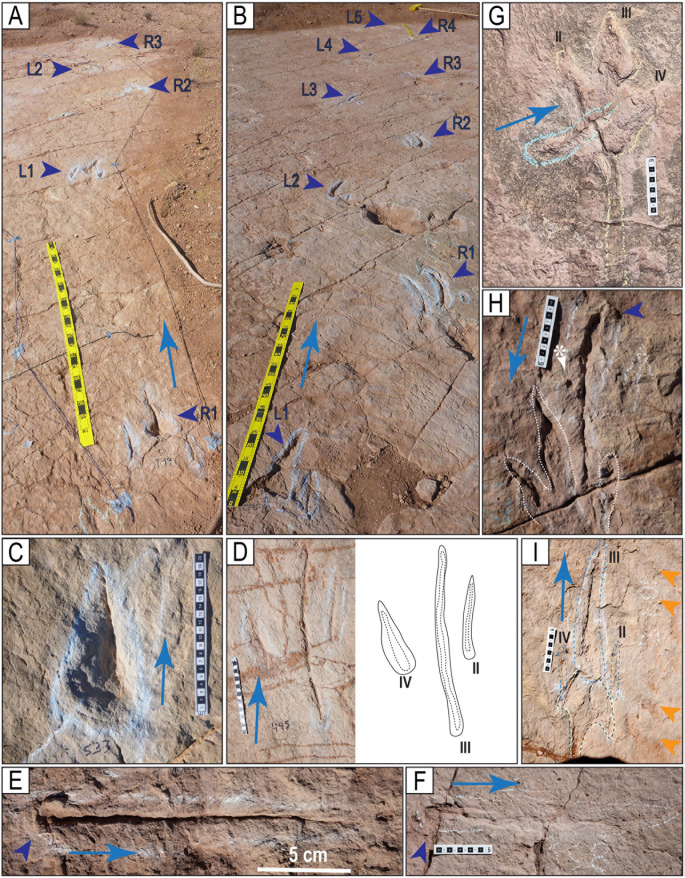
Theropod swim traces. A) Swim trackway CP6-S1 consists of five exposed sets of traces of morphotype S1. B) Swim trackway CP6-S2 comprises nine exposed sets of traces of morphotype S1. Notice the longer step and stride in A compared to B. C) Single swim trace CP7-S33 of morphotype S2. Notice the prominent expulsion rim on the posterior side. D) Swim track T22-2-495 features long, slender scratches of the three digits of morphotype S3. E) Detail of a straight swim trace of TS102 of morphotype S3. F) Swim trace TS8 of morphotype S3, measuring 45 cm in length. G) Swim trace intersecting a theropod track that was previously printed. Notice the posterior ridge of the track and the strong asymmetry of digits II and IV. H) Swim trace TS107 crossing a theropod track. Notice the occurrence of two rosette-like burrows (white asterisk). I) Swim set 8 from the swim trackway TS104 intersecting the posterior side of a tridactyl track. The three traces of the swim set are indicated with dotted blue lines. The four orange arrowheads denote the crests of four wave ripples. Notice the abundant superficial burrows within and outside the track. Blue arrows indicate the direction of swimming. The scales are in cm.

**Fig 45:**
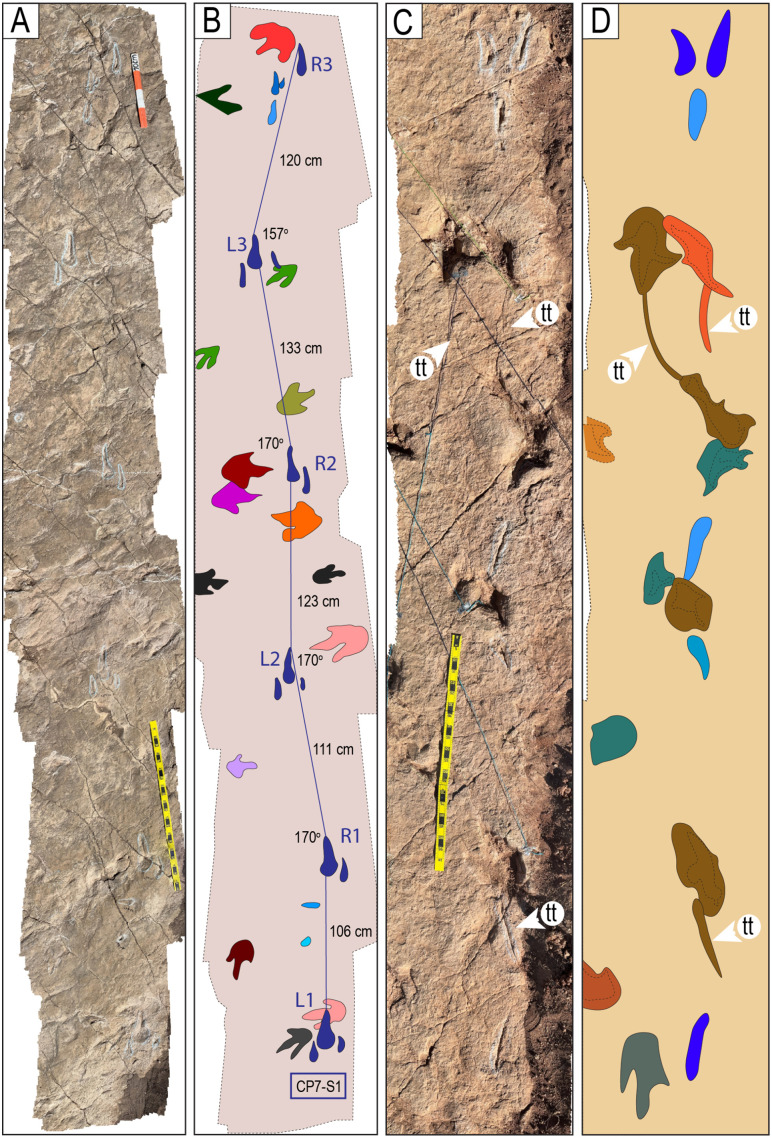
Swim trackways and tail traces in site CP7. A–B) Swim trackway CP7-S1 consists of six consecutive tracks. The PANG values, ranging from bottom to top of the figure, vary from 170° to 157°, which predominantly correspond to a narrow gait trackway of walking tracks. Other swim trackways are shown in several shades of blue. C–D) Swim tracks depicted in various shades of blue. The brown and orange trackways feature tail traces (tt).

**Fig 46:**
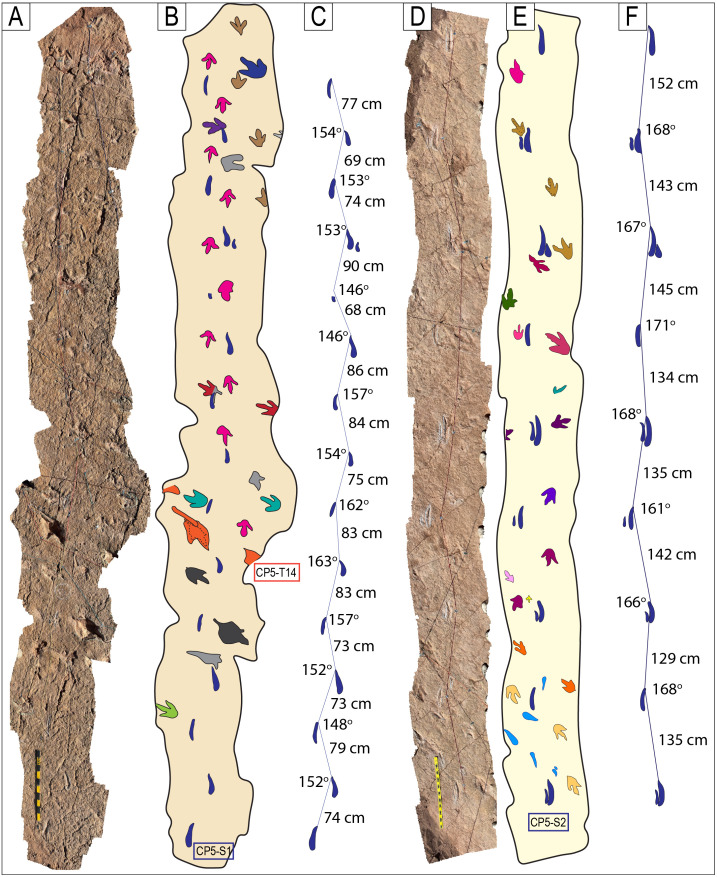
Swim trackways in site CP5. Swim tracks in blue color. A–C) Swim trackway CP5-S1, consisting of fifteen consecutive swim tracks. The values of the PANG correspond to a wide-gauge walking trackway. Notice the variability of the pace length between 68 cm and 90 cm. Notice a track with a tail trace in the trackway CP5-T14. D-F) Swim trackway CP5-S2. The values of the PANG correspond to a narrow-gauge walking trackway. Notice the high values of the pace length for each step compared to the swim trackway CP5-S1 (A–C).

More than thirty trackways with tail traces were found at the Carreras Pampa tracksite, a remarkable occurrence because tail traces are uncommon in theropod trackways. Tracks with tail traces are illustrated in Figs 6B–6E, 21–23, 24C, 24D, 25A, 25B, 38, 44C, 44D, 45A, 46D and S9 Fig. This is the first record of dinosaur tail traces in the South American continent, the first record of theropod tail traces in the southern hemisphere, and the second record of theropod tail traces in the Upper Cretaceous after Xing et al. [118] reported a purported tail drag mark in the Xiaohutian tracksite of the the Xiaoyan Formation, Huangshan City, Anhui Province, China. A detailed description of their characteristics and a discussion of their formation will be published in a forthcoming publication.

The tail traces occur in trackways with shallow tracks of style M3, deep tracks of style M4 (Figs 21–25C, 25D, 26A, 26B and 39), and, most frequently, very deep tracks of style M5 (Figs 6, 21–24, 38 and S9 Fig). These traces are linear or semi-straight grooves, often curved toward the trackway midline and preserved behind the posterior edge of the track, or they emerge from the anterior edge of the track, commonly between the impressions of digits II and III. Exceptionally, they may be sigmoid. In several cases, the tail traces are located laterally adjacent to the tracks. In some instances, the tail trace is marked across part or all of the track. The traces are narrow and of uniform width, except for the proximal and distal ends, which are acuminate. The distal end often terminates in a sediment mound, which may also be present laterally along the trace. In a few cases, the trace extends continuously from one track to another (Fig 39D and 39E).

McLarty et al. [224] analyzed 10 trackways with associated tail traces from the Carreras Pampa tracksite. They found that the presence of a metatarsal mark, anterior depth, and pace length were significant predictors of the association between tail traces and tracks. When anterior depth was removed from their models (to account for the possibility that any penetrative tracks may not have preserved the actual sinking depth of the foot), the presence of a metatarsal mark and pace length remained significant predictors of a tail trace being associated with a track. These results suggest that sinking into the substrate played a crucial role in the formation of most tail traces at the tracksite and likely resulted from some type of locomotive behavior exhibited by the dinosaurs when walking, which involved sinking into the substrate. Some tail traces are associated with shallow tracks, which suggests that tail traces formed through more than one mechanism. However, further study is needed to understand the mechanisms for tail traces associated with shallow tracks.

Dinosaur (ornithopod and theropod) trackways with tail drag traces are rare and have been recorded in only a few locations. Kim and Lockley [225] mentioned thirty-eight records in their review of dinosaur tail traces, with fourteen occurrences of theropod trail traces from the Jurassic and Lower Cretaceous. Since then, other tail traces from tracksites have been reported. Kim et al. [226] reported a theropod trackway composed of six consecutive tracks with a nearly continuous tail trace from the Saniri Formation of Gaesanri tracksite, North Chungcheong Province, South Korea. Lockley et al. [155] reported a large theropod trackway with a tail trace at the Lower Cretaceous Chabu tracksite in Inner Mongolia, China.

##### Miniature tracks.

Several miniature tridactyl tracks, measuring less than 10 cm long, are found in the Carreras Pampa tracksite (Fig 40 and Table 3). The presence of diminutive tracks at this Carreras Pampa tracksite is not surprising, given the abundance of preserved tracks. However, very small or miniature tracks are rare in the fossil record; thus, their occurrence in the Carreras Pampa tracksite is highly significant. We do not know if the trackmakers were juveniles of the larger trackmakers or a different species of smaller body size.

**Table 3:** Dimensions of the miniature tracks. FL and FW are in cm.

Track	FL	FW	αII–III	αIII–IV	αII–IV	ATL/ATW
CP22–320	5.7	4.4	35	29	63	0.36
I22-54	4.8	3.4	24	29	51	0.38
I22-76	4.6	4.0	27	38	65	0.37
S23-9	3.1		40			
CP9-S3	2.7	2.3	28	40	69	0.33
T133 (mean values)	~7.1	1.6	25.6	26.0	51.7	0.69

The preservation of miniature tracks suggests that the rheological conditions of the substrate were suitable for the formation and preservation of miniature theropod tracks or very small tracks of other taxa in at least some areas of the tracksite. Most trackways consisting of small to large tracks could be traced from one edge of the tracksite to the other and likely continued beneath the overlaying sediment beyond the tracksite exposure. However, many of the trackways, consisting of miniature tracks, appeared and disappeared on the track-bearing surface, away from any of the edges. Despite thorough searches under optimal lighting conditions, no additional tracks belonging to these trackways were found. This pattern resulted from varying substrate consistency during track formation. The dinosaurs that left these tiny tracks created impressions in softer sediment; however, these trackmakers were too light to leave marks in firmer ground. This caused trackways to appear and disappear suddenly as the trackmaker transitioned from a firm substrate to a soft substrate and then back to a firm substrate again [21].

At Carreras Pampa, most diminutive tracks are solitary, a phenomenon common in other tracksites worldwide [e.g., 227]. Only a few tracks form a short trackway segment. Trackway T133 is an exception at 39.05 m long, containing 99 miniature tracks with an average length of 7.1 cm. These tracks are mesaxonic with slender, acuminate digits, some exhibiting well-defined claw traces (Fig 40A–40K). Miniature track CP9-S3 in Fig 40H displays moderate mesaxony, round digits, and blunt claw marks. The heel impression is round, revealing a hollow consistent with a *Skolithos*-like burrow. Nevertheless, except for the T133 trackway mentioned above, the remaining miniature tracks are solitary prints.

Henderson [52] found that calculating hip height using 4.5x foot length was the most accurate method for small theropods. Using this formula, the smallest track in Carreras Pampa would have been made by a trackmaker with a hip height of 12.2 cm, and the largest of the miniature tracks would have been left by trackmakers with hip heights of 32.0–34.7 cm.

#### The tracks were made by theropod dinosaurs.

The morphological differences (morphotypes) among footprints and trackways indicate that the tracks did not result from one individual repeatedly visiting the site, but rather from multiple distinct individuals walking in the area. The walking tracks at Carreras Pampa are theropod footprints. Most tracks are tridactyl, while some tracks of the preservation style M1 are didactyl or monodactyl, and some are tetradactyl with hallux impressions. The lack of manus impressions indicates that obligate bipeds are present and eliminates quadrupedal trackmakers. We identify the Carreras Pampa walking tracks as theropods based on the following criteria that distinguish tracks of tridactyl theropods from ornithischians: 1) the trackways are narrow; 2) relatively long steps; 3) asymmetry of the footprint due to a medial indentation (notch) between digit II and the metatarsophalangeal print (heel impression); 4) narrow digit impressions; 5) mesaxonic digit impressions (the impression of digit III is longer than those of digits II and IV); 6) slightly sinuous impressions of digit III; 7) the orientation of the tip of digit III toward the midline of the trackway; 8) the prints of the digits end in claw marks; 9) the claw marks show a convergent margin or constriction at the junction with the digital pads; 10) pes rotation is commonly positive (inwardly oriented toward the midline); and 11) tracks are longer than wide [228–232].

##### Possible sizes of trackmakers.

Paleontologists often rely on correlations between foot length and body size derived from studies of known theropod skeletal fossils. Additionally, the size of a theropod trackmaker can be estimated using the dimensions of the footprints, as determined by the formula *h = *4FL [51,52]. Tracks under 20 cm often belong to juveniles or small species (hip height ~0.8 m, length ~2–4 m), such as *Coelophysis*. Tracks 20–40 cm in length suggest mid-sized theropods (hip height ~1–1.6 m, length ~5–10 m), such as *Dilophosaurus* or *Allosaurus*. Tracks over 40 cm are attributed to large theropods (hip height ~2 + m, length ~10 + m), such as *Tyrannosaurus rex* or *Giganotosaurus*.

Fig 41 shows the calculated hip height, with 79% of the trackmakers having a height between 65 cm and 110 cm. Fig 42 shows the calculated size of the trackmakers at Carreras Pampa. For the aggregated sites, 80% of the trackmakers had a height at the hip between 65 cm and 115 cm (mean 90.64, n = 1146), with 53% (n = 611) in the 75–105 cm range. Significantly, few trackmakers were taller than 125 cm; however, a few taller trackmakers walked on sites CP4, CP5, and CP7, and very few in the other sites. Overall, the trackmakers that walked on site CP7 were slightly taller than those at the other sites, which may reflect some group behavior.

The size of trackmakers can also be described in terms of body mass. Weems [191] proposed that the body mass (BM) of theropods can be calculated using the formula BM = (4.73H)^3^, where H represents the hip height in meters. This equation relies on an accurate estimation of the hip height to accurately estimate the body mass of the trackmaker. In this study, we use the formula *h* = 4FL to calculate hip height (Fig 41), which tends to give relatively conservative estimates for the speed of trackmakers [53]. It has also been argued that *h* = 4FL is more accurate than other methods [52]. Lockley and Xing [233] reviewed the estimated range of body masses for theropod dinosaur trackmakers from various tracksites, spanning from the Triassic to the Cretaceous. They used two formulas to calculate hip height, first proposed by Thulborn [38]: *h* = 4.5FL for tracks less than 25 cm and *h* = 4.9FL for tracks over 25 cm. Thulborn [234] called these morphometric ratios. In the following discussion, we estimate body mass using the hip height equations of [38] to compare them with the findings of Lockley and Xing [233]. We estimated the body mass of trackmakers for trackways that contained two or more tracks and had data for track lengths. Body mass was estimated for 962 trackways, and hip height was calculated using the two methods described above. Of the 962 trackways, 21 had an average track length of 25 cm; in this case, we used *h* = 4.9FL to calculate hip height using morphometric ratios. Fig 43 illustrates the distribution of body mass estimates obtained from both hip height calculations.

A problem arises when using the morphometric ratios for hip height. Trackways for which we estimated body mass have a continuum of track lengths ranging from 5.5 to 43.3 cm. When using the morphometric ratios to calculate hip height, as described by Thulborn [234] and Lockley and Xing [233], there is a notable increase in the hip height estimate when tracks transition from less than 25 cm to 25 cm or greater. For our trackways, this transition occurs between 24.9 to 25 cm. This increases the estimated hip height of 45 cm with only a 0.1 cm increase in track length, creating a 46 kg difference in the body mass estimates (the gap seen in the lower histogram in Fig 43B).

Calculating hip height from *h* = 4FL produces a continuum of body mass estimates that follow track lengths. However, the highest estimated body mass using this method is almost half that of the highest estimated using the morphometric ratios (550 kg vs 1011 kg). We do not seek to determine which method is better for estimating body mass; we only draw attention to the significant difference between the two calculations. In the following discussion, we use the results from the morphometric ratios since that is the method Lockley and Xing used [233].

Schroeder et al. [235] proposed three size classes for theropod dinosaurs based on body mass: small (less than 100 kg), intermediate (between 100 and 1000 kg), and megatheropod (more than 1000 kg). They found that most adult theropod dinosaurs were either below 100 kg or above 1000 kg and proposed that juveniles of megatheropods filled the ecological niches of predators in the 100–1000 kg body mass range. At Carreras Pampa, the trackways we estimated body mass for suggested that most trackmakers fell within the small (440 trackways, 46%) and medium (521 trackways, 54%) size classes.

Only one trackway had an estimated body mass of over 1000 kg. This suggests that megatheropods were potentially rare in this region or in the local area during the time of track formation. The presence of large megatheropods in the TTNP is supported by a natural cast of a very large theropod track (71 cm long) found in a track-rich layer at a tracksite on the outskirts of Torotoro (1.9 km from site CP2), now housed in the headquarters of the TTNP. This track provides an estimated body mass of 4456 kg for the trackmaker, probably a member of the carcharodontosaurid family. We have not studied this tracksite in detail; therefore, we do not know if this track interval indicates a greater presence of megatheropods than Carreras Pampa.

If Schroeder et al. [235] are correct that juvenile megatheropods filled the ecological niches of intermediate-sized predators, then the high number of trackways with body mass estimates within the intermediate range suggests a large number of juvenile theropods were moving through the local area during track formation. An alternative possibility is that one or more intermediate-sized theropod species were present locally during the formation of the tracks. Esperante et al. [24] described a tracksite near Carreras Pampa with two track-bearing levels. The upper level contained 63 trackways with two or more tracks. The body mass estimates from this site were similar to those at Carreras Pampa, with 35% falling within the small range and 65% within the intermediate range. None of the trackways had an estimated body mass over 1000 kg. This finding suggests that intermediate-sized trackmakers represented a large portion of the theropod dinosaur community within the local area, not just for a single track-bearing interval. A comparison between Carreras Pampa and the Cal Orcko site in Sucre, Bolivia, would be of interest to evaluate if this pattern is also present. These comparisons will help clarify if the pattern is localized within the Torotoro area or present over a larger region of Bolivia.

Other Upper Cretaceous tracksites also suggest a high number of small and intermediate-sized trackmakers. Lockley and Xing [233,236] analyzed track assemblages from the Upper Cretaceous Laramie Formation (Maastrichtian) in Colorado, USA. Trackmakers fell within the small and intermediate size ranges (most within the small range) with one exception: one track had a length of 70 cm, representing an estimated body mass of 4,270 kg. Xing et al. [118] presented the maximum length measurements for the “best-preserved theropod tracks” from two Upper Cretaceous tracksites in China. There were 34 tracks for which definite measurements were given (some tracks had maximum lengths given as greater than some value, i.e., “>11.3”). Of these 34 tracks, 62% had small body mass estimates, 38% had medium body mass estimates, and none fell within the megatheropod range. Huh et al. [122] presented data from three tracksites from the Upper Cretaceous of South Korea. Track length data were given for 57 trackways across the three sites. The body mass estimates for these tracksites were 51%, 40%, and 9% for the small, intermediate, and megatheropod ranges, respectively. The lack of megatheropods at these sites may result from the absence of theropods in these size ranges or be a product of the relatively short time intervals during which tracks were made.

##### Possible ichnotaxonomic identity of trackmakers.

The attribution to an anatomical group of theropod dinosaurs and the discussion of the ichnotaxonomic attribution are not the aims of this study. However, the different morphotypes can be compared to footprints figured in other studies. Many of the tracks found in the previously studied Km99 tracksite, a few km from the Carreras Pampa tracksite [24], and this study are comparable to the *Grallator-Anchisauripus-Eubrontes* plexus on its general morphology and configuration of digital impressions. Morphotypes T1–T4 (Figs 12 and 13) bear morphological resemblance to tracks found in the Lower Jurassic dinosaur tracksites from Massachusetts [237], the Lower Jurassic tracks from the Etjo Formation in Namibia [173], and the Lower Cretaceous tracksites of the Huérteles Formation in Soria, Spain [238]. Morphotypes T5 and T6 (Fig 13) strongly resemble pes prints attributed to carnosaur theropods. Many of the tracks in the Carreras Pampa tracksite are also comparable in their general morphology and relative dimensions to large tridactyl footprints produced by tyrannosaurids from the Upper Cretaceous of the USA, including those of *Tyrannosauripus*, which also bears the impression of digit I.

The *Grallator-Anchisauripus-Eubrontes* plexus was named by Olsen [239] based on theropod tracks from the Jurassic of New England, USA. Olsen [240] initially argued that the plexus might represent an allometric growth series, where *Grallator* corresponds to the small and narrow tracks of juveniles and *Eubrontes* to the larger adult tracks, potentially of a single species. Olsen et al. [241] later suggested that the plexus might represent discrete theropod species with similar foot anatomies. What the plexus represents is an ongoing debate for Lower Jurassic track assemblages [177,242,243].

##### Track taphonomy.

Taphonomy of theropod tracks pertains to the processes that influence the creation, preservation, and eventual fossilization of theropod footprints. These processes influence the quality, morphology, and context of the tracks as they appear on the tracksite. In assessing the quality of preservation of the tracks, we find it difficult or impossible to use the numerical scale of morphological quality suggested by Marchetti et al. [244, Table 1]. The mixed morphological traits of the Carreras Pampa tracks, along with the variety of styles of preservation, hinder the use of their numerical scale. Furthermore, we find that the absence of certain traits, which are assigned a low numerical value of 1 for preservation, might accurately reflect the quality of preservation. In Table 1 of Marchetti et al. [244], tracks of level 1 are described as “Digit impressions and palm/sole recognizable but incomplete (if taxonomically relevant) (I). Ungual marks and digital pads may be missing. Footprints may be faint, blurred, or distorted; imprint walls may not be well defined (II). A notable example is the tracks of the style of preservation M1, which only show the impressions of the claws and lack those of the digits, sole, and ungual and pad impressions.

Nevertheless, they cannot be considered “poor, intermediate, suboptimal” as the scale claims. Those tracks of the style of preservation M1, which only show the indentations of the claws, are an excellent example of a type of track formation in which only the claws leave a mark on the substrate. The point is that the presence or absence of specific morphological features in a footprint does not necessarily indicate good or poor preservation. Therefore, it cannot be evaluated against a scale of morphological preservation because those traits were never recorded. Yet, the M1 tracks are representatives of a specific interaction between the feet and a substrate that possibly was highly viscous, cohesive, or semi-dry. In conclusion, the groups of morphological characters in the scale of preservation quality by Marchetti et al. [244] could not be used in the study of the Carreras Pampa tracksite.

The scale proposed by Belvedere and Farlow [81, Table 6.1] consists of four steps in increasing the quality of preservation from poor preservation grade 0 (“No visible morphological details”) to excellent preservation grade 4 (“All digit impressions completely sharp and clear; digit walls well defined, all ungual marks clearly preserved, distinct digital pads present”). Steps 2 and 4 include the quality of preservation of manus prints in quadrupedal vertebrates, a feature not applicable in this study. This scale can only be partially applied to the tracks at the Carreras Pampa tracksite because the digital pads are not preserved, except in very few tracks (e.g., Fig 5K). Nevertheless, tracks of the styles of preservation M1 and M2 are graded with a quality of preservation of 0 or 1, and the tracks of the style of preservation M4 are of a quality of grade 4. The tracks of the styles of preservation M3 and M5–M8 cannot be graded using Belvedere and Farlow’s scale.

Exceptional morphological details, such as skin textures, have not been observed in any footprint, and digital pads appear in very few tracks, which are suboptimal (Fig 5K). Both features are also quite rare at other tracksites around the world. One reason for this absence at Carreras Pampa is the presence of surface and vertical burrows in many of the tracks, which would have obliterated the fine details if they had been preserved initially. The morphology and final preservation of tracks and traces in the rocks depend on various factors, including the morphology of the autopodia, the pattern of movement of the animal, the behavior of the producer, the rheological conditions of the sediment at the time of track formation and post-formation processes, as well as post-formation burrowing of the substrate [38,93,98,244–248].

The Carreras Pampa tracksite features numerous tridactyl tracks exhibiting various morphotypes and styles of preservation. Often, some trackways display several styles of preservation and footprint morphology. For instance, most tracks of trackway T22-126 are very deep, belonging to the preservation style M5, while others correspond to mode M4 and consist of very shallow tracks of mode M3. It is common for theropod tracks of different sizes and morphologies to be found at the same tracksite, and even within the same trackway [118,119,249]. In most tracks, the middle digit III is the most deeply impressed into the tracked surface, most likely because it is where the animal placed most of its weight when in contact with the ground.

The intra-trackway variation observed at the Carreras Pampa tracksite indicates that dinosaurs of the same species or even the same individual may produce different types of footprints based on their behavior or the conditions of the substrate. Conversely, dinosaurs of various species can leave similar structures when they exhibit the same behavior. Morphological variation along single trackways is also observed at other track sites, with differences in length, width, depth, and other characteristics, which strongly suggests that track morphology is significantly influenced by the properties of the substrate [144]. At the Carreras Pampa tracksite, the substrate conditions varied, as reflected in the different styles of preservation and morphotypes.

The characteristics of the sediment (grain size and shape, layering) and the water content determine the properties that regulate the ground’s response to treading and, thus, strongly control the track’s morphology [42,57,86,89,250–252]. These properties are viscosity (plasticity), consistency (cohesion), and adherence [40].

Laporte and Behrensmeyer [253] assert that there is a narrow range of sediment textures and moisture content for preserving vertebrate tracks. Sediments that favor the preservation of vertebrate tracks include carbonate mud and silt, fine sand, and volcanic ash. Fine-grained sediments, such as clay, have similar rheological properties, but tracks are rarely preserved in such sediments. Loose, unconsolidated sands tend to collapse into the surface’s deformations while exposed to wind or are reworked by water currents [253]. Nevertheless, tracks have been recorded in fine-grained sandstone sediment [254]. The ichnological literature indicates that many dinosaur tracksites are found in layers of carbonate sediments. The rheological characteristics of fine-grained carbonate sediments (viscosity, consistency) render them a common substrate for footprint recording.

Viscosity measures a substrate’s resistance to deformation, ranging from fluidity (a liquid or gas) to rigidity (a solid rock). Tracks would not form under the rheological conditions at these extremes. There are both shallow and deep tracks within the same trackways, indicating that the viscosity, or ability to penetrate the substrate, was highly variable [40]. In the Carreras Pampa tracksite, the viscosity of the substrate varied over time and space, as evidenced by the existence of several modes of preservation (from very shallow to very deep tracks). Some trackways exhibit changes in depth along their path, with shallow tracks followed by significantly deeper tracks of preservation style M5, often accompanied by tail traces. This variation is common in most natural subaerial substrates that go through wet and dry cycles. Dinosaurs moved through the Carreras Pampa site at different times, causing changes to the substrate’s physical properties. Additionally, the substrate exhibited different behaviors from one point to another, as indicated by tracks of varying depths within the same trackway.

The length of the digit impressions is consistent in all the tracks of the same trackways. Although this is common in trackways elsewhere, a notable exception is the tracks of the LS1 trackway at La Senoba, La Rioja, Spain, where some tracks have longer digit prints than others due to slipping [40].

The consistency or cohesion of the sediment is evident in the deformation that occurs after the foot is withdrawn from the substrate. Consistency is high if the walls of the shaft remain vertical, whereas it is low if the walls of the shaft collapse due to deformation. The consistency of the sediment must have been high during the time of track formation because the shafts of the tracks are intact, including the walls of the digit imprints, which do not show evidence of collapse like those observed in the Santisol, La Senoba, La Era del Peladillo, La Virgen del Prado, and many other tracksites in La Rioja, Spain [40,115], except the limited collapse observed in the distal portions of digits II and IV of tracks of morphotype T3, and some tracks of style of preservation M5. At the La Rioja tracksites, the collapse primarily affects the anterior part of digit III, resulting in a broad and short impression of digit III. At La Virgen del Prado, the substrate collapse impacted the entire interdigital space, fusing the walls of all three digits [40]. Those features are absent at the Carreras Pampa tracksite, where the impressions of the digits are separate and well-defined, except in the very deep tracks of style M5 and the tracks of style M1, which consist solely of claw impressions. The absence of collapse structures in the tracks indicates that the sediment’s consistency was high at the time of track formation.

Adherence refers to the extent to which a substrate adheres to the foot of a trackmaker. This factor significantly influences the formation, morphology, and preservation of theropod tracks. High adherence results in a sticky substrate, which occurs in substrates with high moisture content, such as sticky mud or clay. The substrate sticks to the bottom of the autopodium during the kick-off phase of locomotion, resulting in substrate deformation at the center of the footprint or near the toes. When lifted, the foot pulls material upward, creating distinct features such as toe drag marks, rim structures around the track due to displaced sediment, and sediment mounds in the center of the track. High adherence may produce well-defined and deep tracks, often with detailed impressions of toes, claws, and skin textures. Low adherence results in a non-sticky substrate, which is characteristic of firmer, compacted sediments or dry, sandy substrates where the foot leaves minimal drag or displacement, leading to shallow or less-defined tracks. Low adherence may produce tracks with only the general outline or partial impression of the toes.

We observed minimal surface substrate deformation at Carreras Pampa, except in a few tracks exhibiting sideward expulsion rims. The tracks and the associated sediment do not display structures (e.g., ejecta) that would indicate the sediment’s adherence to the bottom of the autopodia.

According to the behavior of the substrate, the tracks can be classified as follows:

1) There are very deep tracks (depth > 3 cm) with vertical walls. These tracks indicate that the substrate was of low viscosity but high cohesiveness.
2) Shallow and very shallow tracks, located next to or in the same area as deeper footprints, indicate that the substrate had high viscosity and cohesiveness. For the description of morphotypes, we established a category of deep tracks (1–2 cm) that characterizes tracks with the best-preserved morphological details.

The preservation potential is minimal under conditions of prolonged exposure without any sedimentation. The morphology and preservation of footprints depend heavily on the events that occur after the tracks are formed, because exposed prints are highly susceptible to destruction through erosional processes (e.g., water currents, wind, invertebrate bioturbation). Preservation is favored by rapid lithification and burial [255]. Therefore, the cementation of the studied trackbearing layer of Carreras Pampa had to occur relatively rapidly before erosion could take place during exposure or before the deposition of the overlying layer. The deposition of the overlying thin green layer (Fig 2) filled the depressions between ripple marks, theropod prints, and delicate avian tracks, and this process must have occurred without disrupting those previously formed structures. Footprint recording happens under conditions of rapid cementation.

## Swim tracks

**Fig 47:**
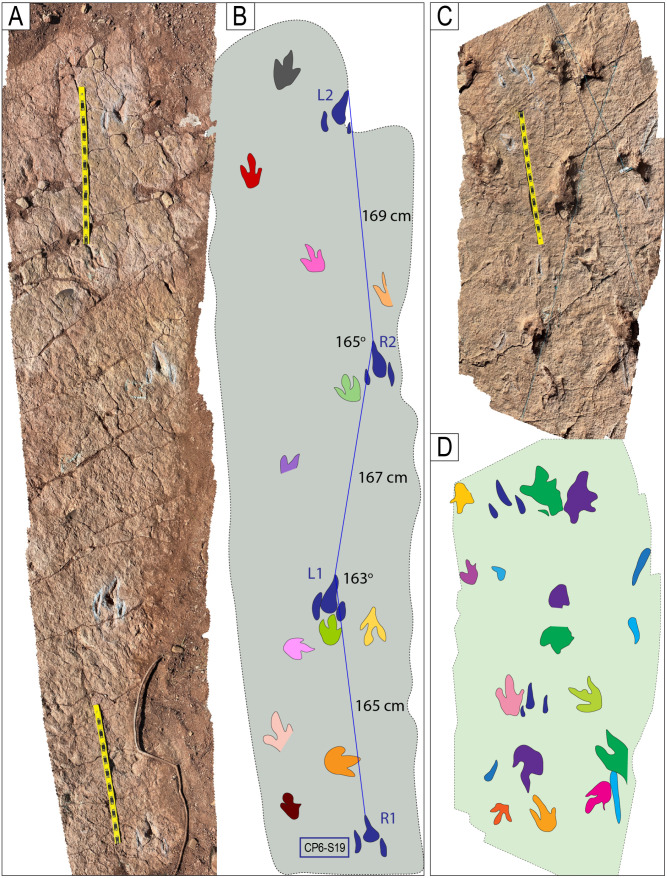
Swim trackway CP6-S19 and solitary swim tracks at site CP7. A–B) Swim trackway CP6-S19 and associated walking tracks. The swim trackway consists of four continuous tracks. The values of the PANG correspond to a narrow-gauge walking trackway. C–D) Solitary swim tracks in several shades of blue, along with associated walking tracks at site CP7. The walking tracks exhibit styles of preservation M4 and M5. The scales are 1 m. Notice the root of an ‘espinillo’ (Acacia caven) shrub in the lower part of the photo in A.

**Fig 48:**
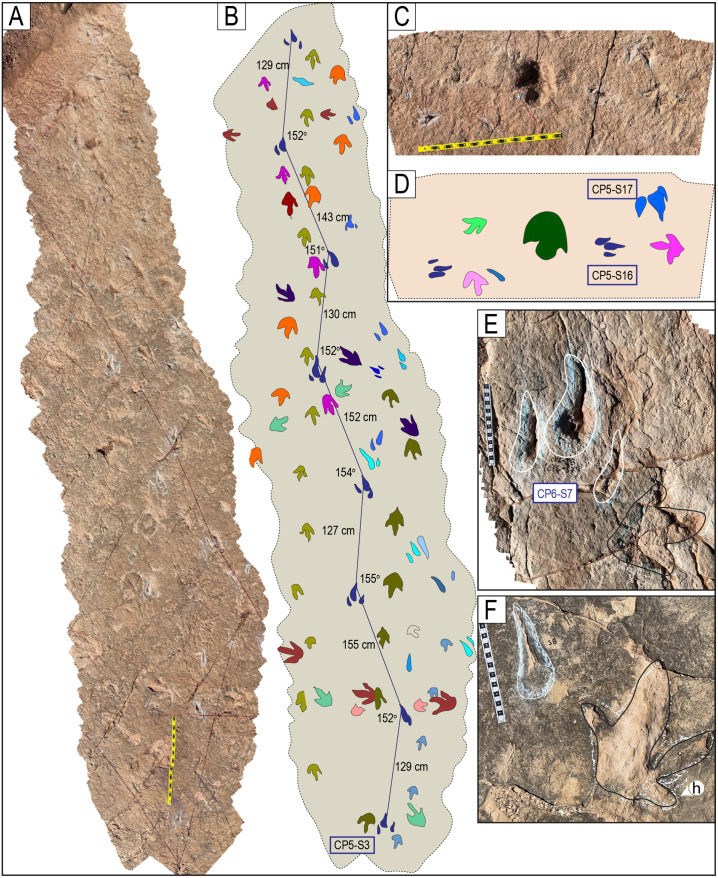
Swim tracks and trackways in sites CP5, CP6, and CP7. A–B) Swim trackway CP5-S3 and the associated walking tracks. Other swim tracks are represented in shades of blue. The PANG values (151°–155°) correspond to a relatively wide-gauge walking trackway. C–D) Four sets of swim traces and the associated walking tracks in site CP5. E) Swim track CP6-S7 consists of one medial long trace and a smaller lateral trace on each side, corresponding to the scratch made by the claw of digit III and the claws of digits II and IV, respectively, with an associated walking track. F) Swim track CP7-S8 consists of a single scratch made by the claw of digit III. An associated walking track of preservation style M3 is preserved on the side, with a well-marked impression of the hallux (h). The scales in A–D are 1 m, and in E–F are 20 cm.

Theropod swim tracks are abundant at all Carreras Pampa sites, with most forming continuous swim trackways (Figs 44–48 and S11 Fig). A detailed description and discussion are being prepared by several of the authors. Here, we present preliminary observations and comparisons with swim traces in other tracksites. The terms “swim trace(s)” and “swim track(s)” have been widely used interchangeably in the ichnological literature to refer to structures resulting from the swimming activity of reptiles and dinosaurs, including theropods [77, 256–262, among others]. In this study, the terms “swim track” and “swim set” are equivalent, referring to the elongated scratches or grooves resulting from the swimming action of one of the trackmaker’s feet. Each of the individual scratches or grooves within a swim set is referred to as a “swim trace.” The term “swim trackway” refers to the linear succession of swim sets produced by the same animal during a single swimming movement, indicating the sets or groupings of elongated traces in an alternating right-left disposition at regular spacing. A complete swim set consists of three traces with an extended medial groove corresponding to the scratch made by digit III, and one smaller trace on each side produced by digits II and IV, respectively. Incomplete sets of one or two traces are common in the tracksite and consist of the medial trace (digit III) alone or the medial trace and the inner trace (digit II).

### Criteria to differentiate swim tracks

There is abundant literature on vertebrate footmarks made underwater, including discussions about their formation, producers, and criteria for determining the paleoenvironment (dry ground vs. underwater).

Peabody [263] interpreted some tracks in the Lower Triassic Moenkopi layers of the western United States as footprints of tetrapods that were likely swimming. The traces show partial impressions made by the tips of the digits; usually, only digits III and IV are printed. For Peabody, the diagnostic characteristics of swim tracks were the lack of any clearly defined print of a foot, the lack of trail continuity, and the general appearance of the trace as being made by the tips of digits.

Brand [264] compared trails of small living amphibians and reptiles made on dry, damp, wet, and subaqueous sand to the fossil trails in the Permian Coconino with trails obtained in laboratory experiments using living animals and observed that the laboratory trails form continuous traces across all the substrates. The presence of toe marks, a short sole impression (compared to its width), and a uniform appearance throughout the trails most closely resembles the trails produced by living tetrapods on a subaqueous substrate.

Lockley [265] referred to isolated toe impressions and elongated drag traces found in the Chinle Formation of eastern Utah and tentatively attributed them to swimming phytosaurs. Traces from the Triassic Red Beds of Wyoming, commonly considered prod marks of driftwood, were reinterpreted by Boyd and Loope [266] as marks produced by swimming tetrapods. The criteria used to identify these traces as swim tracks included striations on the surface of the mark, similar dimensions, expulsion rims, regular spacing and orientation, and reflectures (a s- or z-shaped continuation of the trace originating from the posterior of the track created by retraction of the foot).

Coombs [267] described swim marks of a possible theropod in the Jurassic of Connecticut. These marks occur in two sizes, with their middle digit, III, being the longest, and have both a claw and phalangeal pad impression. The lateral digits II and IV are shorter. Some scratch marks show sediment mounds at the posterior ends corresponding to expulsion rims formed by the plowing action of the digits.

Thomson and Lovelace [268] indicated that the diagnostic criteria for swim tracks in the Moenkopi and Red Peak formations are the presence of longitudinal, highly variable trace lengths, kick-off scours, digit reflectures, incomplete or unexpected traceway configurations, and posterior overhangs.

According to Xing et al. [262], swim tracks of buoyant tetrapods are characterized by 1) the presence of elongated parallel or slightly divergent striations, scratches or imprints of digits, and claws; 2) the absence of distinct anatomical details such as phalangeal pad impressions or skin texture; 3) the presence of a posterior displacement rim or sediment mound behind digits.

Here, we follow and modify the criteria established by Esperante et al. [24] to define theropod swim tracks and trackways: 1) the presence of a successive linear occurrence of scratches with similar morphology (interpreted here as scratches of digits II, III, and IV); 2) the disposition of three or more sets of scratches in a pattern alternating between the right and left sides of a medial line; 3) the occurrence of three or more sets of scratches shaped curved to the right, alternating with those curved to the left; 4) the presence of smaller side scratches (interpreted here as scratches of digits II and IV) on both sides of a longer medial scratch (interpreted as the scratch of the claw of digit III), in alternating sets of traces; these smaller lateral traces are not found between the sets; 5) the regular spacing of the sets of scratches; 6) the same geographic orientation of all the scratches within any given succession; and 7) the absence of metatarsophalangeal impressions. Several authors assert that elongated and parallel digital impressions and sediment mounds at the posterior ends of digit scratches are common features of tetrapod swim tracks [77,256,269–271]. The absence of metatarsophalangeal region impressions is common in swim tracks, interpreted as scratch marks made on the substrate by the distal ends (claws or toe tips) of the trackmaker’s autopodia [77,256,262,267,271].

### General description of the swim tracks

At Carreras Pampa, we discovered 280 swim trackways and 1378 swim tracks. One hundred fifty-eight trackways consist of solitary swim tracks that do not form sets of successive tracks, while twenty-nine trackways comprise ten or more swim tracks. Recognizing scratches in the sediment as swim traces based on their linear succession (criterion 1) allows us to identify, analyze, and count individual, isolated scratches as swim tracks due to their similar morphology. The longest swim trackway we studied was TS-76, measuring 130.2 m in length. To date, it remains the longest exposed swim trackway in the world. Many other swim trackways in the Carreras Pampa tracksite have lengths of several meters.

Milner et al. [77] reported “approximately 3200 individual claw marks” at the St. George Dinosaur Discovery Site at Johnson Farm (SGDS) in southwestern Utah (Lower Jurassic), being the “largest and best-preserved true swim track assemblages ever recorded” (p. 315). The claw marks occur as “sets of three and rarely singular or paired” scratches and were interpreted as swim traces of theropod dinosaurs. The swim track assemblage in the Carreras Pampa tracksite is exceptional due to the abundance of tracks and excellent preservation of the traces. The Carreras Pampa tracksite has the largest number of swim tracks outside the North American continent and the most significant number of swim tracks worldwide that form continuous trackways.

All the swim tracks in the Carreras Pampa tracksite are preserved alongside true theropod and bird tracks as concave epireliefs, with elongated, straight, or comma-shaped grooves. These are often accompanied by one or two smaller side traces of similar morphology, which show no metatarsophalangeal pad impressions (foot outlines), as would be expected from imprints that result from the weight-bearing phase of walking on dry land [cf. 98]. The long medial trace corresponds to the mark left by the scratching of the third toe (digit III) during the swim action, while the two smaller lateral grooves correspond to the traces left by digits II and IV. They typically exhibit an inward curvature toward the midline. These traces are regularly spaced, showing an unambiguous alternation between right and left, corresponding to the right and left pedes, and they are oriented in the same direction within the same linear trackway.

The scratches made by digit III are longer and deeper, while those produced by digits II and IV are shorter and shallower. Xing et al. reported that in two theropod swim trackways at the Lower Cretaceous Guanghui site in China, the digit II marks are consistently shorter and deeper than the digit IV marks. However, this is not the case at Carreras Pampa, where the trace of digit II can be longer, shorter, or equal in length to the trace of digit IV, and often the trace of digit IV is absent (S11 Fig).

The tracks may be shallow or deep and often show an expulsion rim, which is more pronounced in the central groove of the deep traces. This expulsion rim corresponds to the posterior side of the trace and was formed when the autopodium’s third toe pushed the soft sediment of the substrate backward during the swimming action.

Shallow swim tracks can be quite long (35–55 cm), uniform in depth and width, and tend to be straight, slightly curved, or sinuous. They lack an expulsion rim and are very shallow, occurring in isolation from other swim traces. Typically, they do not show marks from the claws of digits II and IV. Deep swim tracks are shorter (15–30 cm, though exceptions exist) and tend to be markedly curved or slightly sinuous. They also exhibit a prominent expulsion rim on the side opposite the direction of swimming (the posterior end). These tracks start shallow and narrow at the anterior end, then progress deeper and wider toward the posterior end, with the expulsion rim changing from narrow and shallow to wide and thicker. The curved deep swim tracks may also feature a very curved expulsion rim, giving the entire structure a comma shape.

### Swim track morphotypes

We distinguish three swim track morphotypes, S1, S2, and S3, which correspond to the swim morphotypes S1, S2, and S3, respectively, reported by Esperante et al. in the nearby tracksite Km99 (this tracksite was briefly exposed and then removed during the construction of a road to the town of Torotoro).

#### Swim track morphotype S1 – Comma-shaped scratches.

These scratches can vary in length, being either short or long, and are slightly curved or comma-shaped (Figs 44A, 44B, 44G–44I and S11A–11I Fig). The tracks feature a shallow, sharp anterior tip that transitions into a slender groove, which widens and deepens posteriorly, culminating in a conical to subconical and rounded depression or ‘reflecture’ that ranges from deep to very deep (1–3 cm) [sensu 271], with an asymmetrical displacement rim of sediment. These features occur only in true footprints, not underprints [93]. Commonly, the rim initiates halfway through the trace and appears on both sides along the posterior half of the rectilinear section of the scratch, continuing to the curved end. The posterior end often has a bulb shape, with an asymmetric displacement rim that is wider and thicker toward the inner side, aligning with the midline of the swim trackway. Frequently, these traces are accompanied by one or two smaller side scratches located on the right and left sides near the posterior bulbous end of the medial scratch. The side scratches are either similar to the medial one or consist of straight, slender, and shallow scratches or prod marks, but with a terminal rounded displacement rim resembling that of the medial long scratch. This type of swim track is the most abundant at the Carreras Pampa tracksite.

#### Swim track morphotype S2 – Teardrop-shaped scratches.

These swim tracks have a teardrop shape and consist of single, short prod marks (Fig 44C). Some are wide (2–3 cm), while others are narrow (0.5–1 cm); they are typically short in length (2–5 cm). Scratches in this category are often deep (1–2 cm) and may lack displacement rims or can be very shallow and thin. The tracks are shallow at the anterior end and deepen toward the posterior end. Swim tracks with this morphotype commonly occur in isolation, without forming recognizable swim trackways. This type of swim track is abundant at the Carreras Pampa tracksite.

#### Swim track morphotype S3 – Very long (30– > 120 cm) scratches.

These swim tracks consist of very long, shallow, narrow scratches corresponding to digit III of the foot, sometimes accompanied by a tiny side groove, typically from the claw of digit IV, and rarely by a short groove corresponding to the claw of digit II (Figs 44D–44F and S11J Fig). These scratches can be straight but are most commonly slightly curved, though not sinuous. Often, they show evidence of sediment collapse and obliteration of the scratch, resulting in traces of uniform or nearly uniform width without displacement rims. This morphotype of swim track is very rare in the Carreras Pampa tracksite. The two side markings are smaller than the medial trace in the three swim morphotypes.

Commonly, and most notably in morphotype S1, the distal ends of the traces left by digits II and IV are subparallel or parallel, situated closer to the print midline than in walking tracks, as the toes were not spread out and bearing the weight of the animal [272]. However, in large-sized swim sets of morphotype S1, the traces of digits II and IV may be arranged diagonally to the trace of digit III, with the trace of digit IV demonstrating a wide aperture; in rare cases, the posterior end even partially crosses the midline. Most of them occur on the sides of the posterior expulsion rim; however, in some rare instances, they occur more medially, toward the anterior end of the central trace. Exceptionally, the small side mark of digit II is parallel to the trace of digit III, and the trace of digit IV is marked posteriorly and diagonally to the trace of digit III. The swim tracks preserve marks made only by the claws inserted into the soft substrate, and there is no evidence of the impression of the distal phalangeal pads.

Coombs [267] argued that swim tracks were inconsistent with their formation as undertracks or imperfect prints in a very firm substrate, as the well-preserved bourrelets indicate.

### Orientation of swim tracks

Fig 30K illustrates the orientation of solitary swim tracks and trackways for all sites combined. The rose diagram indicates a strong trend toward the SSE (120°–180°), with a minor component toward the NNW (330°–350°).

The visual observation of the swim trackways reveals that they are very straight or exhibit only a slight change in direction, without any sudden turns. This slight change in direction may result from avoiding another swimmer or from a water current that pushed the trackmaker sideways.

### Pace length and angulation of swim tracks

We have data on the PL and PANG of five swim trackways (Table 4, Figs 45–48 and S11F Fig). The PL and PANG vary greatly within trackways, although the swim trackways can be categorized into the same two types: narrow-gauge and wide-gauge, similar to the walking trackways. The mean PANG in the five swim trackways is near or above the lower range for narrow-gauge walking trackways (≥165°). The mean PL of the CP5-S1 trackway is significantly smaller than that of the other four swim trackways measured.

**Table 4:** Values of PL and PANG for measured swim trackways in sites CP5, CP6, CP7, and CP9. Decimal values are rounded up to the nearest whole number.

Swim trackway (Fig)	Number of tracks (n)	Mean PL (cm)	Range of PL (cm)	Mean PANG	Range of PANG
CP7-S1 (44)	6	119	106–133	167^o^	157^o^–170^o^
CP5-S1 (45)	14	78	68–90	153^o^	146^o^–163^o^
CP5-S2 (45)	9	139	129–152	167^o^	161^o^–171^o^
CP5-S3 (47)	8	188	127–155	152^o^	151^o^–155^o^
CP6-S1 (S10F Fig)	5	177	174–182	163^o^	161^o^–165^o^
CP6-S19 (46)	4	167	165–169	164^o^	163^o^–165^o^
CP9-S3	23	149	135–163		

### Formation of swim tracks

Several lines of evidence suggest that theropod dinosaurs likely produced the swim tracks in Carreras Pampa: 1) all the true tridactyl tracks preserved on the rock surface of Carreras Pampa are theropod and avian; 2) the bird tracks lack webbing, unlike those of swimming birds. Given that swimming birds have webbed feet, it is unlikely they would have scratched the substrate and left long, deep tridactyl furrows with prominent displacement rims; 3) the length and width of the bird tracks are under a decimeter, indicating a small bird trackmaker, whereas most of the swim tracks have a decimetric length, which corresponds to a larger trackmaker; 4) it is improbable that a small trackmaker would exert the pressure needed to create deep and wide scratches and produce displacement rims of the size observed in the swim tracks (Figs 44A–44C, 44H, 48E, 48F and S11 Fig); and 5) the preservation of the true tracks, posterior ridges, tail traces, and bird tracks indicates a short interval between their impression on the substrate and the impression of the swim tracks, which occurred afterward. Therefore, it is likely that the same type of theropod that created the true tracks and tail traces also produced the swim tracks. The occurrence of a large number of theropod tracks and the absence of other tetrapod tracks (except avian tracks) further suggest that these dinosaurs were the producers of the swim traces.

The swim tracks do not register two categories of morphologies that differentiate between manus and pes prints, potentially assigned to swimming crocodiles, as reported in other tracksites [e.g., 273]. Crocodilian swim tracks can be tridactyl, tetradactyl, or pentadactyl. Typically, tridactyl crocodilian swim tracks display three elongated, parallel scratches that are slightly sinuous and of similar length, or with the medial trace longer than the other two. At the Carreras Pampa tracksite, the swim tracks are never tetradactyl or pentadactyl.

At Carreras Pampa, most swim trackways consist of continuous successions of multiple sets of traces, with all successive sets preserved and clearly marked on the exposed surface of the tracksite (Figs 44–48 and S11 Fig). The sequence presents an alternation of right-left traces with a concave curvature toward the midline of the trackway. This alternation indicates that the theropod producers swam by alternately moving their right and left legs. The swim tracks were likely formed when the claws or toes of the swimming theropods scraped the soft substrate. The swim tracks comprise sets of three parallel or subparallel scratches. However, tracks with sets of two or a single scratch are common, which is consistent with the expected scratches left by the claws of tridactyl theropods, rather than by the four-digit hindfeet or five-digit forefeet of crocodiles.

Some swim trackways have one or more missing sets (S11 Fig). Some swim trackways consist of only a few (2–5) sets of traces that show no further continuity on the rock surface, and many solitary swim tracks exist. Although the missing swim tracks could be attributed to preservation factors, the excellent preservation of most swim tracks suggests that taphonomic factors are not responsible for the lack of traces in a succession of swim trackways. Most likely, the absence of some swim tracks in succession or the lack of further continuity in a trackway can be explained by variations in the buoyancy of the producer during the swim action, resulting in intermittent contact while swimming or wading.

The morphologies of the ichnites, their ichnological relationship to true tracks, and their sedimentological context indicate that the traces are not undertracks but were formed underwater during a swimming gait in buoyant conditions. Essential differences between terrestrial and aquatic locomotion can explain the ichnological characteristics of vertebrate traces. Variation in the morphology of subaqueous traces, including differences in both inter-trackway and intra-trackway depth and length, pace, and stride, can be attributed to variations in water depth, dimensions of the feet, differing hydrodynamic conditions of the water body (e.g., currents, waves), substrate properties, speed of paddling, and the propulsive force applied by the autopodium into the substrate.

We did not observe any swim tracks cross-cutting other swim tracks. Still, some swim tracks cut across previously formed walking tracks and ripple marks (Fig 44G–44I), which indicates that the theropods walked on the substrate first, the water level rose, and then the dinosaurs swam, leaving scratches on the still soft bottom.

The ripple crests on sites CP2 and CP3 are oriented within the range of 275°–297°. However, these ripple crests are deformed by both walking and swimming tracks, and therefore do not indicate the direction of wave motion at the time the swim tracks formed. Nevertheless, the paleogeography is unlikely to have changed significantly between the initial wave ripple formation and the secondary flooding event during which the swim tracks formed. If this is the case, the swimming direction would have been perpendicular to the wave motion. Some asymmetrical swim track marks, however, suggest they were countering a current or wind that was oblique to the wave ripple crests. The uniform appearance of swim traces along individual trackways and among different trackways suggests a consistent water depth. We do not know what size classes the trackmakers that were swimmers belonged to.

A truly swimming animal is fully buoyant and does not need to contact the substrate to support its weight or propel its body forward. However, contact with the substrate may occur under certain conditions (e.g., limb length, water depth, behavior). McAllister [274] asserts that in a floating organism, the digits can extend farther posteriorly in the propulsive phase without the animal losing balance. This allows the animal to exert the propulsive force on a more horizontal plane, scratching the bottom instead of pressing downward into the sediment. Depending on the extent of foot-sediment contact, a paddling animal can create various traces with this horizontal movement. For instance, it is expected that if the digits barely touch the substrate while paddling, the foot will not encounter much resistance from the substrate and will continue posteriorly along the arc described by the limb. The resulting trace will be a long scratch mark in the substrate, deepest in the middle or rear end. If the phalanges extend further into the substrate, more resistance will be exerted on the foot, and the limb will not complete the described arc. Under conditions of lower water levels or low buoyancy, the paddling animal’s feet would touch the ground, inserting the claws into the soft sediment, generating a propulsive force that pushes the animal forward and leaves a deep groove on the bottom. The claw’s intrusion into the soft sediment and subsequent limb movement would push the displaced sediment backward, forming a displacement rim along the scratch and, more noticeably, at the rear end. As a result of the propulsion force, the scratch trace may display a sediment overhang or expulsion rim at the posterior end. Morphotyp S1 is interpreted this way. Therefore, the expulsion rim is located opposite the direction of movement.

The Carreras Pampa swim tracks indicate that, during the swimming action, digit III left long and deep marks, typically with a conspicuous, well-defined rim of sediment displaced from the anterior to the posterior end. In contrast, digits II and IV left shorter and shallower marks, often lacking rims or featuring smaller posterior mounds. The expulsion rims may be very shallow or nonexistent, but are usually noticeable. The largest expulsion rims in all the studied sets were discovered in swim trackway CP6-S3, with swim set R1 displaying a rim 20 cm long (anterior-posteriorly), 30 cm wide, and 3 cm thick above the surrounding flat substrate. Other swim tracks in CP6-S3 also exhibit very prominent expulsion rims (Figs 44A–44C, 44H, 48E, 48F and S11 Fig). Additionally, the traces from that swim trackway are the deepest among all the studied sites, with depths ranging from 3.1 to 8.1 cm.

Swim morphotypes S1 and S2, characterized by their deep grooves and pronounced posterior displacement rims, suggest that the claws of the autopodium were embedded into the soft substrate rather than merely resting on the surface. The rim resulted from the compression of the digit into the relatively soft and plastic sediment during the swim step cycle [84]. The bulb-shaped depression or ‘reflecture’ at the posterior end is interpreted as displaced sediment during the final push and release of the claw from the substrate. It is possible that the deepest parts of the swim grooves became partially closed by sediment coming in from the walls. Still, the bulk of the groove remained intact because the displaced sediment did not collapse into the groove of the swim track after the claw was retracted from the substrate.

Swim morphotype S2 is very similar to the depression of pes digit III in tracks interpreted as theropod swim impressions found at Rocky Hill, Connecticut [267, Figs 1A and 2]. Expulsion rims or sediment mounds at the posterior end of the traces are a common feature in tetrapod swim tracks [256,257,269–271].

Under conditions of high buoyancy, if the digits barely touched the bottom while paddling, we would expect the limbs to rotate swiftly from the anterior to the posterior side of the body. This would result in elongated striations on the substrate, with minimal depth in the scratch, leading to a very shallow displacement rim or its absence at the posterior end of the trace. The resulting traces would be the long, slender, straight, or slightly curved furrows of morphotype S3 presented here. This morphotype appears to match McAllister’s description of traces produced when the digits “barely touch the substrate” [269]. This situation was probably caused by a higher water level or buoyancy than that when swim traces of morphotypes S1 and S2 were made.

Thulborn and Wade [93] illustrated the morphological variations in various footprints made by coelurosaurian theropod and ornithopod dinosaurs at the Lark Quarry tracksite in western Queensland, Australia, under conditions of decreasing water content in a muddy substrate. The variability of the morphology of the prints is interpreted as differential results of the three phases of interaction between the autopodium and the substrate: touch-down or T-phase, weight-bearing or W-phase, and kick-off or K-phase. In Fig 1, these types of traces represent long, parallel impressions formed during the Bb and Bc stage of the kick-off (K) phase on a firm substrate where the feet of the coelurosaur theropod did not sink. In the model of Thulborn and Wade [93], posterior mounds may result from the push of sediment backward by the uplifting claws; thus, there is a possibility that the three scratches might correspond to walking locomotion rather than swimming.

Several features of the Carreras Pampa swim tracks and walking tracks do not align with Thulborn and Wade’s model for the scratches in the Bb and Bc stages of the kick-off phase: 1) none of the walking tracks exhibit a posterior rim of sediment as the swim traces do. At Carreras Pampa, there is a clear distinction between the “normal” walking tracks and the other traces, which we interpret as a different mode of locomotion—swimming; 2) the three scratches in each set are inconsistent in size and morphology within the same trackway, and often, only one or two scratches are present in each set; and 3) the curvature of the scratches, especially of digit III, and their orientation do not match the putative impression left by the autopodium pressing on a soft or firm substrate. Therefore, the sequences of sets of furrows in Carreras Pampa cannot be interpreted as resulting from “normal” walking on a firm substrate when the foot fails to sink into the substrate. We surmise that these sets of furrows are swim tracks made by swimming theropods.

Swim tracks at Carreras Pampa resemble those figured by Xing et al. [92] at the Lower Cretaceous Guaghui tracksite (Taoqihe Formation) in China, consisting of long, slender, parallel, tapering scratches that lack any impressions made by the metatarsophalangeal regions. Xing et al. [262, pp. 448–449], describing elongated and parallel digit traces interpreted as swim tracks, assert, “In all cases, the anterior portions of the impressions are deepest, and the traces become shallower posteriorly. These features indicate that the distal tips of the digits initially contacted the sediment with the greatest impact force, and that the foot was then lifted as it moved posteriorly, propelling the animal forward and pushing the sediment backward. Contrary to this description, the anterior portions of the Carreras Pampa swim tracks are shallower than the posterior portions. We interpret this feature as the trackmaker gradually inserting the claws more deeply into the substrate and pushing the sediment backward as it withdrew the claws.

Following Lockley et al. [275], it is worth noting that swim tracks are morphologically highly variable, a feature that we also observed in the study of the Carreras Pampa tracksite. Variability includes features such as the number of traces (scratches) in each track, the dimensions of the traces, the amount of foot surface printed (only the claws printed or part or all of the foot), and the presence of interdigital webs. We do not find swim tracks at Carreras Pampa that record the entire foot or show evidence on interdigital webs.

Milner et al. [77, p. 315] asserted that at the SGDS tracksite in southwestern Utah, the swim traces are “often incomplete, occurring in irregular and confusing configurations” and consist of ‘elongate, parallel to subparallel “scrape marks” […] that occur in high densities, almost invariably *without* associated walking traces.’ In common with this description, the Carreras Pampa swim traces are not associated with corresponding walking traces, although walking traces are abundant on the same rock surface. This suggests that the swim traces had a different mode of formation than the “regular” tracks on the same surface.

Milner et al. [77] distinguished three morphological categories of swim tracks, which they interpreted as having formed in different water current conditions: 1) Inferred down-current traces, with variable claw impression depths, the deepest and longest trace made by the mesaxonic digit (digit III); 2) traces oriented up-current that consist of ubiquitous parallel scrape marks, vary considerably in overall dimensions, and are usually in sets of three, occasionally with two or one claw marks, the mark of digit III being the longer and deeper of the three scratches, and 3) cross-current swim tracks that have a similar morphology as those of category 2. Similar to the swim traces figured by Milner et al. [77], the swim traces at Carreras Pampa occur in sets of three, two, or single scratches, often in parallel. Still, they differ in that the overall morphology is more comma- or drop-shaped, and the size of the scratches of the claws of digits II and IV is much smaller than the size of the scratch of the claw of digit III. Except for a swim trackway in site CP2, we do not observe morphological features in the Carreras Pampa swim trackways that would allow us to determine the direction of the water current and whether the animal was swimming up-current or down-current. This feature is part of another investigation by a team member.

Alexander’s [51] formula for calculating hip height based on the size of the track requires the presence of the metatarsophalangeal pad impression, which is lacking in the tracks. We hypothesize that the size of the trackmakers for the swim tracks was the same as those that left the true digitigrade tracks on the same substrate, based on the short time the tracking surface remained soft.

### Ichnotaxonomic attribution of the swim tracks

The three theropod swim morphotypes reported from the Carreras Pampa tracksite were also found at the Km99 tracksite in the TTNP, Bolivia [24]. One of us (RE) has observed swim tracks of morphotype S1 at the Jarun Llust’aqani tracksite, located five kilometers southeast of the Carreras Pampa tracksite, on the eastern slope of the Torotoro Syncline, and in the Upper Jurassic to Lower Cretaceous Castellón Formation in the Canyon of the Santa Ana River, Tarija, Bolivia.

Similar theropod swim tracks have been reported in the Middle Jurassic Saltwick Formation of England [276], the Lower Jurassic Zagaje Formation of Poland [277], the Upper Jurassic of the Lusitanian Basin [278], the Lower Cretaceous Feitianshan Formation of Sichuan, China, [271] the Lower Jurassic at Rocky Hill, Connecticut, USA, attributed to the *Eubrontes* ichnotaxon [267, see Fig 2A and 2B], and in the Lower Cretaceous of the Cameros Basin, Spain [40,256,279–281]. Grooves GCI-T1 and GCI-T2 at the Ganchong tracksite I, China, interpreted as possible tail traces by Xing et al. [262], are morphologically comparable to swim traces at the Carreras Pampa tracksite. Possible theropod swim traces have been found in the Middle Jurassic Gypsum Spring Formation in Wyoming [109, Fig 15]. Two traces from the Hettangian of Sołtyków, Poland, consisting of sets of three parallel grooves [277, Fig 10], with possible affinity to the ichnotaxon *Characichnos tridactylus*, were tentatively assigned to swimming theropods and are similar to style S3 of Carreras Pampa.

Three ichnogenera have been assigned to traces of swimming archosaurus: 1) *Characichnos*, 2) *Hatcherichnus*, and 3) *Wintonopus*. *Characichnos* is interpreted as a theropod swim trace [77,271,275,276]. Other prints interpreted as *Characichnos* swim traces have been reported in the Upper Jurassic Morrison Formation of Colorado [282], in the Lower Cretaceous of La Rioja, Spain [281] and in the Maastrichtian El Molino Formation of Bolivia [24].

*Hatcherichnus* is inferred to represent a crocodilian swim trace [258,278,283,284], and is characterized by a tetradactyl pes impression consisting of a digit-only trace, with outside digits I and IV nearly equal in length, as are middle digits II and III. The outside digits are approximately 25% shorter than the middle digits [283,285]. Contrary to this description, the Carreras Pampa swim traces are never tetradactyl but mo nodactyl, didactyl, or tridactyl, and the outside digits II and IV are different in length compared with the median digit III. Moreover, the overall morphology of *Hatcherichnus* differs from that of the swim traces described in the Carreras Pampa tracksite.

The *Wintonopus* tracks of Lark Quarry [98] consist of tridactyl, mesaxonic pes traces that are wider than long; digit traces are short, with digit III being the longest and digit IV being equivalent to or longer than digit II; both digit II and IV traces extend farther proximally than the digit III trace. Except for digit III being the longest, none of the other traits of *Wintonopus* match the characteristics of the swim traces observed at Carreras Pampa. The ichnogenera *Wintonopus* was first described as formed during a stampede of a mixed herd of small ornithopod and theropod dinosaurs in the mid-Cretaceous Winton Formation, Queensland, Australia, as tridactyl tracks of running dinosaurs registering only the distal parts of their digits. Later, Romilio et al. [286] reinterpreted the tracks as evidence of swimming dinosaurs, an interpretation challenged by Thulborn [287], who reaffirms his original interpretation of these tracks as made by running ornithopods during a stampede. The discussion highlights the challenges of interpreting these tracks, which only capture the distal portion of the digits. The morphological characteristics of *Hatcherichnus* and *Wintonopus* do not correspond with the traces we report from the Carreras Pampa tracksite.

The swim tracks reported here from the Carreras Pampa tracksite are tentatively assigned to the ichnotaxon *Characichnos*, first identified in the Middle Jurassic Saltwick Formation of England [276]. With Whyte and Romano’s ichnotaxon diagnosis, the Carreras Pampa swim tracks share the following traits: 1) several elongate, parallel epichnial grooves, which may be straight, gently curved or sinuous; 2) the termination of the grooves may be straight or curved; 3) the trackway consists of two rows of traces, and 4) the long axes of the traces are parallel to each other in a straight trackway.

Sadlok and Pawełczyk [261] distinguished between compact and spread morphologies in vertebrate swim tracks. The compact appearance consists of sets of swim scratches contacting each other, whereas the spread appearance shows separated scratches–the typical morphology of the type material of *Characichnos* ichnites [cf. 278]. Sadlok and Pawełczyk [261] attribute the two different appearances to behavioral and anatomical causes, with spread morphologies likely attributed to the presence of interdigital webs in the feet of the trackmakers, which may or may not have been impressed on the substrate. The swim tracks of the Carreras Pampa described here are of the spread type but do not record the impression of any interdigital web.

### Taphonomy of the swim tracks

Two significant features have been reported in convex epirelief swim traces of tetrapods: 1) thin longitudinal striations 1 mm in width on each digit mark, presumably produced by scales or claws on the foot of the tracemaker, and 2) curved digits with v-shaped, s-shaped, or z-shaped reflectures produced when the tracemaker’s foot changed direction during trace formation.

These features have been reported in the Lower-Middle Triassic Moenkopi and Red Canyon Formations of the Western United States [268], in the Lower Triassic Baranów Formation of the Holy Cross Mountains, central Poland [261], in the Lower Jurassic Moenave Formation of Saint George, Utah [77], and in the middle Cenomanian Dunvegan Formation, northeast British Columbia, Canada [273]. These features are lacking in the concave epirelief swim traces at Carreras Pampa. This absence may be related to the preservational potential of exposed trackbearing surfaces. The striations observed in at least some of the track sites have been recently eroded from cliffs or exposed on the surface, whereas the Carreras Pampa track site has been exposed for a long time, and the minor preservational features may have been eroded as a result. Moreover, the images of the convex hyporelief swim traces with striations do not show the rock surface disturbed by invertebrate bioturbation. Even minor surface invertebrate bioturbation would have obliterated fine details such as striations.

## Avian tracks

**Fig 49:**
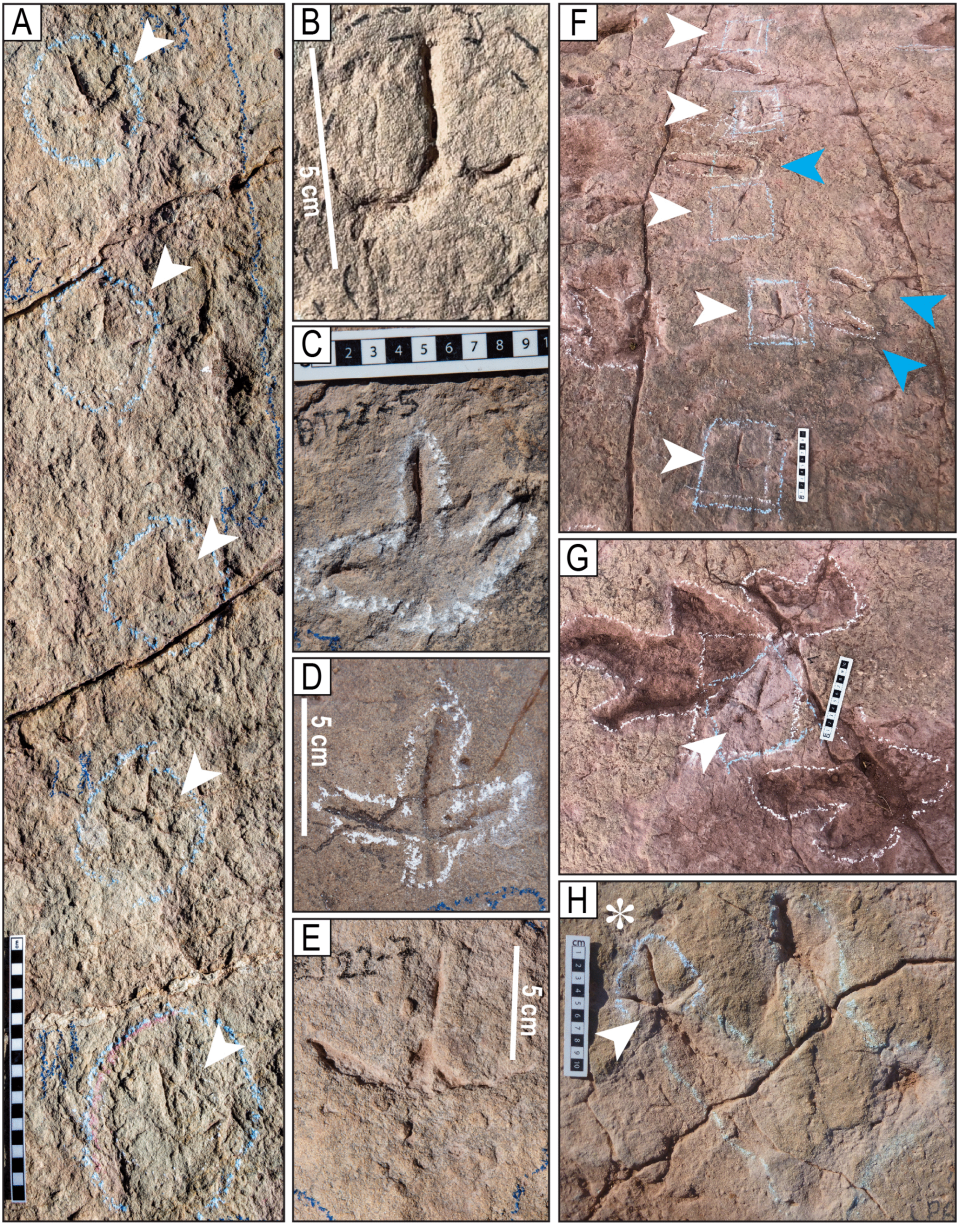
Avian tracks associated with theropod and swim tracks. A) Track BT22−1 features five consecutive impressions. B) Track BT22−9. C) Track BT22−5. D) Trackway BT22−6. E) Track BT22−7. F) Trackway showcasing five consecutive impressions. White arrowheads denote the bird footprints, while blue arrowheads represent theropod swim tracks. G) Track associated with three theropod tracks at site CP1. H) Bird track (black arrowhead) at the distal end of digit II of a theropod track. Tracks in A–E originate from site CP1, the track in F comes from site CP2, and tracks in G–H are from site CP6. The scales are measured in cm.

At the Carreras Pampa tracksite, avian tracks have been found in sites CP1, CP2, CP3, CP6, CP7, and CP9 (Figs 1, 49 and S12 Fig) and are likely to be found in the other sites [25]. Within the TTNP, avian tracks have been reported at the dinosaur tracksite Camino El Vergel, 1.7 km northeast of the Carreras Pampa tracksite [26], and a detailed study has been published in a thesis [288].

These tracks differ from typical theropod walking tracks. They are not penetrative theropod tracks. The dimensions, overall divarication angles, and the presence of a backward-facing fourth toe suggest that these tracks are avian rather than theropod. Furthermore, many of these tracks form recognizable trackways with a pronounced inward rotation of the foot, a feature common in some avian trackways.

We found several hundred solitary avian tracks and many avian trackways within the study sites. Avian trackways were particularly abundant in site CP9 and the northeastern side of site CP2. A detailed study of the avian tracks at these sites is ongoing and will be published.

The association of avian and dinosaur tracks, especially those of theropods, is relatively common in many Lower and Upper Cretaceous tracksites. In China, it has been reported from numerous tracksites [e.g., 289–294]. Avian and dinosaur tracks have been reported in the Campanian Anacleto and Las Curtiembres formations [295,296], as well as in the Maastrichtian Yacoraite Formation of Argentina [297,298]. The latter is a sedimentary succession considered correlative to the El Molino Formation in Bolivia [28,299,300]. In North America, they have been reported from the Albian-Cenomanian Dakota Formation in Utah [301], the Aptian Gething Formation in British Columbia, Canada [302], the Cantwell Formation of Denali National Park, Alaska, USA [303], and the uppermost Las Encinas Formation, Coahuila, Mexico [304].

## Associated fauna

**Fig 50:**
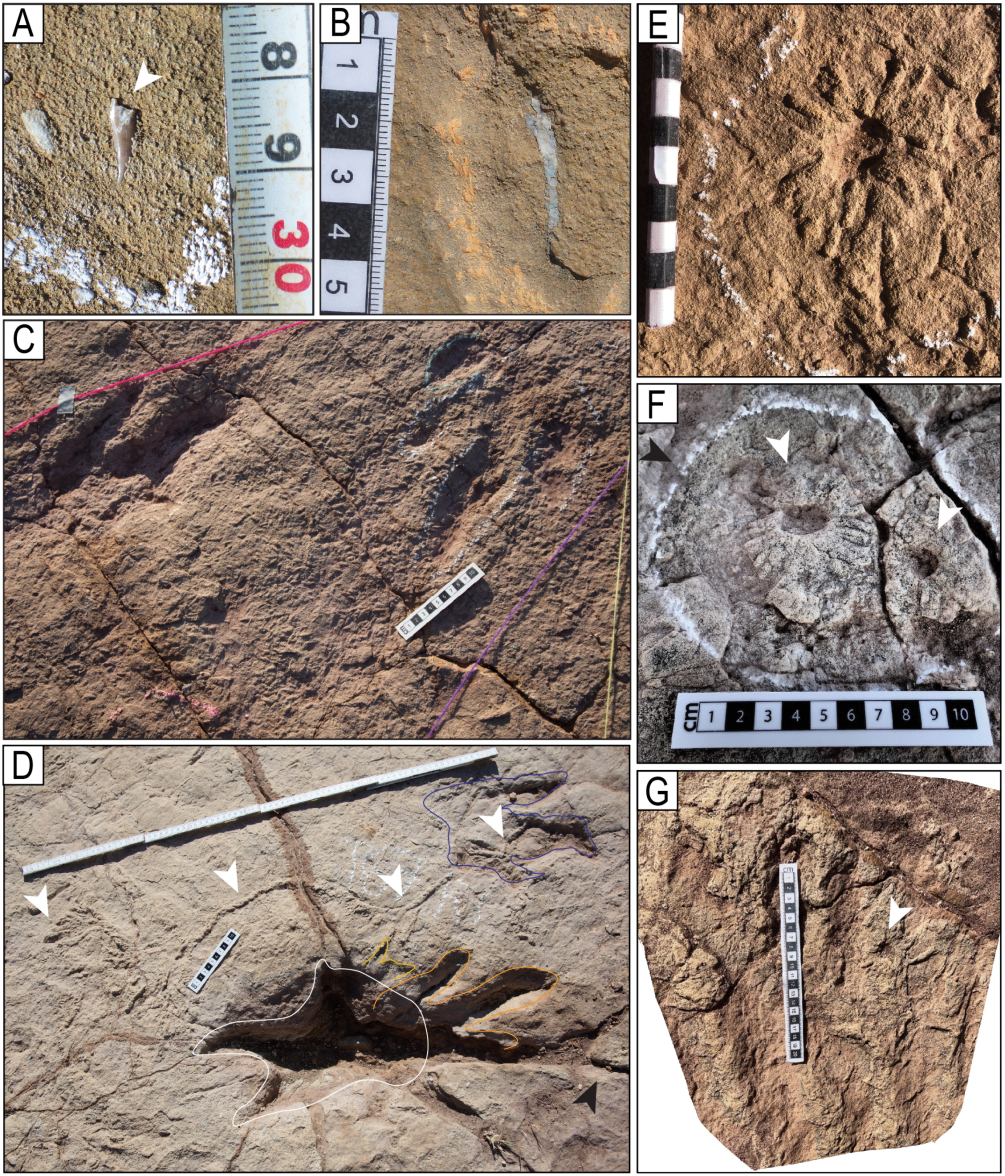
Associated fossils. A) *Pucapristis branisi* fish teeth. B) An osteoglossomorph fish mandible. C) A monospecific assemblage of very small (1 cm) spiral gastropods covering much of the Carreras Pampa tracksite. D) *Hillichnus* burrow (white arrowheads) in site CP3 associated with two very deep tracks of the style of preservation M5, two theropod tracks of the style of preservation M4, and one tail trace (black arrowhead). E–G) Rosette burrows (indicated by white arrowheads) on the surface of the tracked layer. G) A rosette burrow on the crest of a ripple and small, curved surface burrows. The scales are in cm.

Little is known about the body fossil content of the Torotoro and El Molino formations in the TTNP. The stratigraphic sections examined by this team in the field have yielded few fossils, primarily isolated bone fragments, fish teeth, shell fragments, and a well-cemented bivalve shell bed in the middle member of the El Molino Formation. In 2023, we discovered a dinosaur vertebra in a sandstone layer alongside theropod tracks, as well as dinosaur limb bones and a rib in a gray siltstone layer, both of which were found in the middle member of the El Molino Formation along the Sucusuma River. A cross-bedded carbonate-cemented sandstone, located 3.2 m stratigraphically above the tracked layer, has yielded some *Pucapristis branisi* fish teeth (Fig 50A). Thus far, these teeth have been identified only in the Maastrichtian deposits of the El Molino Formation in Bolivia and the Yacoraite Formation in northwestern Argentina [305–307].

The tracked layer at Carreras Pampa has yielded only fragments of body fossils, including shells and fish teeth. In site CP7, a fragment of an osteoglossomorph fish mandible was found (Fig 50B). This likely corresponds to *Phaerodusichthys taverni*, as reported by Gayet et al. [305] and Gayet [308] in deposits of the El Molino Formation in several locations in central Bolivia.

A large area of the surface of site CP2, along with parts of the other sites, is covered with an assemblage of monospecific, very small (1 cm) spiral gastropods. The shells are not oriented but scattered randomly. In some areas, the shells have eroded, leaving small lenticular concavities on the surface of the substrate. We interpret these gastropod shells as an episodic accumulation after the tracks were made (Fig 50C).

A low-diversity invertebrate trace fossil assemblage is present on the upper surface of the tracked layer (work in progress), consisting of three types of burrows: *Hillichnus* burrows, radial/rosette-like burrows, and a variety of holes (including vertical holes). *Hillichnus* burrows are rare, and only six more or less complete specimens were found on the exposed surface of the tracksite [309,310].

Radial/rosette burrows (Figs 50E–50G and S3 Fig) represent the most abundant biogenic structures in the entire tracksite. These trace fossils are characterized by a central depression or vertical shaft surrounded by radiating burrow structures that resemble the petals of a flower or a rosette. Their density is 60–65/m^2^, which is particularly noticeable in sites CP1 and CP2, including areas with ripple marks, where they appear in both the crests and troughs, with very few within the tracks. The rosette-like burrows within the prints indicate that the latter are true tracks, not undertracks. Invertebrate bioturbation altered the surface layer and modified the preservation of the printed area, resulting in tracks that lack fine anatomical details of the surface of the autopodium.

## Paleoecology

It is well known that Bolivia has one of the highest concentrations of dinosaur footprints in the world, but a scarcity of skeletal remains. Only scattered bones have been found, and no dinosaur bones have been excavated using professional paleontological methods in Bolivia. Our team discovered two dinosaur vertebrae in the Sucusuma tracksite, located about 5.2 km from the Carreras Pampa tracksite. The Sucusuma tracksite is also part of the El Molino Formation. In 2023, our collaborator Germán Rocha Rodríguez, an engineering geologist based in Cochabamba, Bolivia, informed us of a set of dinosaur limb bones situated about 200 m downstream and stratigraphically above the Sucusuma tracksite in a mudstone layer of the El Molino Formation. Although we documented the findings with photographs, the remains were destroyed before we could retrieve them due to a flash flood on March 9, 2024. In March 2025, we received pictures of three large rock blocks with exposed dinosaur vertebrae from the outcrop following a recent storm. These bones appeared in layers devoid of dinosaur tracks.

Our team found a partial, articulated postcranial skeleton of a terrestrial vertebrate in the sediment above the Carreras Pampa tracksite, about 3 meters from the western edge of site CP1 and 1.5 meters vertically above the tracked surface. A detailed analysis of the skeleton by team member Caleb LePore revealed that it belonged to a Pleistocene ground sloth, deposited in fine red mudstone filling a depression in the red Cretaceous shale that stratigraphically overlies the tracked layer (Fig 2).

The scarcity or total absence of dinosaur skeletal parts associated with or within the same stratigraphic range of footprints has been documented worldwide throughout the Mesozoic. For example, in northern China, vertebrate skeletons from the Tuchengzi Formation (Upper Jurassic to Lower Cretaceous) are scarce; however, numerous tracksites have been discovered, featuring thousands of tracks and trackmakers [294]. The Upper Cretaceous Korean dinosaur record also presents a similar situation regarding the abundance of tracksites and trackways, as well as the scarcity of skeletal remains [168]. However, the Korean ichnological record differs from the Bolivian in the composition of the ichnites: in the former, ornithopod tracks are the most abundant, with sauropod tracks subordinate and theropod tracks very limited [311,312], whereas in Bolivia, theropod tracks are the most abundant, with sauropod tracks subordinate and ornithopod tracks scarce.

The scarcity of dinosaur bones and eggs in sediments containing abundant footprints, and the scarcity of footprints in layers with abundant bones or eggs, is a well-known paradox in paleontology [38]. The reasons for this are still debated. Several factors related to the formation and preservation of fossil tracks versus body fossils are presented here.

1. *Different conditions for preservation.* Footprint formation may require sediment conditions that do not favor the preservation of bones and vice versa. Dinosaur footprints form when the animal walks on a soft, impressionable surface, such as mud, sand, or silt, in relatively few environments, including beaches and shorelines, estuaries, tidal lagoons, floodplains, deltas, lakeshores, waterholes, swamp environments, and desert sandflats and dunes. In these environments, footprints may be expected and preserved through the rapid hardening of the substrate and subsequent burial by fine sediment. Footprints are not destroyed by scavenging, decay or dissolution. Skeletons, however, may not survive in these environments due to rapid disarticulation and erosion by waves in the littoral zones or the abundance of scavengers that consume the carcass before it can be buried and fossilized. One of us (RE, December 10–12, 2003) has observed an abundance of partial skeletons and disarticulated bones of marine mammals in wide shoreline-parallel transects of the Colorado River delta mudflats. The bones persist in the wet sediment of the mudflat for several years in various degrees of preservation [313]. Still, the footprints left by invertebrates (e.g., crabs), birds and humans only last a few hours to a few days before the next tide or the wind erases them. Bones require unique fossilization (diagenetic) conditions, including rapid burial, mineralization, and the absence of scavenging or decay. These conditions may not coincide with the environments where footprints are made. Even within suitable environmental conditions, footprints may only be preserved in particularly favorable areas, which may be limited to a narrow transect with wet sediment [38,253].
2. *High activity areas.* The interpreted paleoenvironments for the tracksites suggest that dinosaurs were actively searching for food or moving to another area, resulting in numerous tracks. These locations are less likely to preserve entire skeletons because such areas are prone to erosion, flooding, and other disturbances that can scatter or destroy bones. The tracksites in the TTNP may have served as extended or short-distance migration pathways; thus, few animals would have died in the area. However, while this may apply to carnivorous dinosaurs (theropods), it is difficult to conceive that, given the high number of individuals represented by the thousands of theropod footprints and sauropod tracks at various tracksites, very few bones would have been buried and fossilized.
3. *Temporal separation.* The formation of a trackway and the deposition of a carcass may not occur simultaneously. A dinosaur might leave tracks in one location and die elsewhere, meaning no bones would be found in the same layer. However, this is also difficult to accept in a scenario of high transit with a potentially large population on the move, as depicted by the tracks at the Carreras Pampa tracksite.
4. *Scavenging.* If a dinosaur died near its tracks, its body might be scavenged or decay before it was buried. Bones could be scattered or destroyed, leaving no trace in the same sedimentary layer. The destruction of organic and bony remains by scavenging is highly likely in the high-transit area of carnivore dinosaurs recorded at the Carreras Pampa tracksite.

The absence of dinosaur bones in the stratigraphic range of the Carreras Pampa tracksite, where abundant tracks are preserved, is both puzzling and expected for the reasons explained above. It aligns with the typical pattern observed worldwide. This also inhibits the identification of the trackmakers with anatomical precision.

## Inferred behaviors

The exceptionally high density of tracks in the Carreras Pampa tracksite may indicate trackmaker behavior, as in the case of shore birds that feed in large numbers and in shore environments [314]. Alternatively, a high footprint density among theropods may indicate high activity levels [315].

Because dinosaurs cannot be observed in action, their behavior is always inferred from the fossil record. This has been a matter of discussion since the discovery of the monodominant skeletal sites of *Iguanodon* and *Plateosaurus* in Europe [316,317]. Dinosaur behavior is inferred mainly from three lines of evidence: 1) trackways; 2) nesting sites [318,319]; and 3) monodominant mass death assemblages (bonebeds). In skeletal taphonomic studies, monodominant bonebeds are evidence of social behavior [320–334]. In South America, gregarious behavior has been proposed based on Lower Cretaceous bone assemblages in the Bajo Barreal Formation [322], the Candeleros Formation [335], and the Huincul Formation in Argentina [336].

However, as stressed by García-Ortiz and Pérez-Lorente [337], it is essential to distinguish between group behavior or gregariousness [338,339] and social behavior [340]. According to the Oxford Dictionary of Ecology [341], ‘gregariousness’ is defined as “The tendency of animals to form groups which possess a social organization,” ‘social behaviour’ is defined as “The interactive behaviour of two or more individuals, all of which belong to the same “species,” and the related term ‘herding’ is defined as “The formation by mammals of groups of individuals that have a social organization.” The concepts of ‘gregariousness’ and ‘herding’ are practically identical, and the two terms imply the idea of forming a group that does something together (e.g., hunting, feeding, migrating, or defending). The fact that animals may be together in a social group does not imply that they exhibit group behavior or gregariousness. In a gregarious behavior, animals gather into structured social groups for a common purpose (defense and protection, hunting, etc.).

Additionally, the term ‘social behavior’ is reserved for the gregarious behavior of individuals of the same species. Thus, forming a group does not necessarily entail a social organization (e.g., ranking, age, or sex distribution); the animals can be together as a social group, either temporarily or permanently, and engage in the same activity. Two examples will illustrate the difference between gregariousness or group behavior and social behavior. A group of humans walking out of a sports stadium exhibits social behavior (they are of the same species), but this does not strictly qualify as group behavior because there is no common purpose (except for the fact that they are exiting a building). Conversely, a group of humans walking behind a funeral vehicle or queuing at an event represents group behavior because there is a common purpose. Tadpoles in a pond live together as a social group, but they do not necessarily display group behavior unless, for example, they swim away from a threat.

### Walking behaviors

A high number of trackways observed in a tracksite suggests high traffic of individuals and gregarious behavior [342,343]. Therefore, based on these definitions, applying these three terms (gregariousness or group behavior, herding, and social behavior) in the study of dinosaur footprints is challenging. Nevertheless, the terms ‘gregarious behavior’ and ‘gregariousness’ have been widely used in the study of dinosaur trackways.

Ichnological features suggesting gregarious behavior, primarily when occurring together, are: 1) the occurrence of multiple trackways of the same type on the same surface; 2) the occurrence of parallel or subparallel trackways; 3) lack of superposition or little overlap of the trackways; 4) similarity between tracks belonging to different trackways; 5) regular trackway spacing, namely the separation of trackways made by two or more individuals walking together; 6) similar speed values; 7) similar pace values, and 8) uniform depth [175,98,344–354].

A literature review indicates that the primary evidence for gregarious behavior is the occurrence of parallel or subparallel trackways in the same overall direction of movement [249,327,337,342,345,348,355–359]. Lucas [338, p. 210] asserted that the “strongest evidence” for gregarious behavior is the occurrence of “multiple trackways of dinosaurs that walked in the same direction.” Ostrom [339] suggested that parallel trackways could result from a biological factor (e.g., gregarious behavior) or a ‘physically controlled’ pathway, such as a geographic barrier. However, the two are not mutually exclusive because a group of dinosaurs might have walked in the same direction (e.g., migrating, hunting), following a pathway restricted by a geographic barrier.

Gregarious behavior in dinosaurs has been proposed based on ichnological evidence from several track sites worldwide and stratigraphic settings, primarily those of ornithopods and sauropods. Parallel ornithopod trackways indicating gregarious behavior are relatively numerous in the Cretaceous of Canada [343,360], China [292], Japan [345], South Korea [168,350,353,361–364], Spain [40,337,365–367], the United Kingdom [368], the Lower Jurassic and Cretaceous of the United States [272,339,352,354,369–374], the Upper Jurassic of Portugal [375], and the Upper Jurassic of Spain [45].

Gregarious behavior has been suggested for tracksites in South America with parallel trackways in the same direction, with similar pace lengths and track morphologies, including the Lower Cretaceous Chacarilla tracksite in Chile with two trackways of large theropods [249], the Lower Cretaceous of the Agrio Formation with four trackways [376], and the Cenomanian Isla de Cerritos tracksite of the Candeleros Formation in Argentina with three trackways [201,377].

Parallel trackways have also been found in sites with sauropod trackways. For example, all twenty-three sauropod trackways identified at the Davenport Ranch footprint locality (Lower Cretaceous Glen Rose Formation) in Texas are subparallel and lead in the same direction [339,378]. For Bird, this was evidence that the animals “passed in a single herd” [378, p. 64]. Other sites with parallel trackways of sauropods have been reported in the Lower Jurassic to earliest Cretaceous Castellón Formation in Bolivia [3], the Middle Jurassic of England [50], the Upper Jurassic of Spain [45], the Upper Jurassic of Portugal [349], the Lower Cretaceous of Portugal [379], the Lower Cretaceous of Spain [346], and the Lower Cretaceous of China [110]. García-Ortiz and Pérez-Lorente [337] summarized the record of twenty-eight pieces of evidence of gregariousness in twenty-five tracksites in the Lower Cretaceous Cameros Basin, La Rioja, Spain, with sauropod, ornithopod, and theropod footprints, and one-way and two-way trackways.

The mention of gregarious behavior inferred from parallel ornithopod and sauropod trackways is relevant here because the parallel theropod trackways of the Carreras Pampa tracksite may reflect the locomotion of one or more herds of carnivorous dinosaurs following or pursuing a herd of ornithopods or sauropods. However, the evidence for such pursuing behavior is lacking at the tracksite, as no ornithopod or sauropod tracks were recorded.

**Fig 51:**
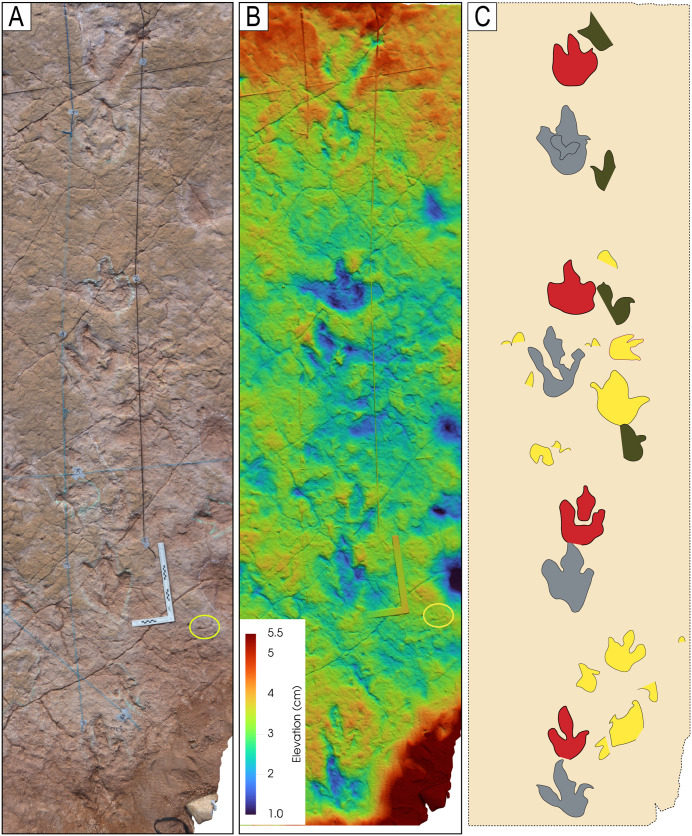
Trackways and solitary footprints at site CP6. Note the three parallel trackways (red, gray, and green) oriented north, which belong to the M3 and M4 preservation styles. Other theropod prints are oriented in different directions. The yellow ellipse highlights a bird footprint. The scale is 35 cm.

At Carreras Pampa, the presence of several hundred trackways in bimodal orientation suggests that some individuals moved parallel to each other in the same direction (Fig 30). Examples of parallel trackways are shown in Figs 21–24 and 51. However, the high number of trackways makes distinguishing parallel trackways of the same morphotype challenging. We remind readers that many trackways registered tracks with more than one morphotype. There is a high level of trackways crossing over (Figs 21–24 and 26–28), which suggests that the trackmakers were moving in the same direction but not necessarily parallel. Furthermore, the occurrence of numerous trackways of the same species at one tracksite is not always proof of gregarious behavior, as it could reflect the passage of different solitary individuals at different moments [337,339].

Some parallel trackways in the Hartford Basin, Massachusetts, USA, have been interpreted as made by a solitary individual passing through the same place and direction at different times due to an environmental setting (geographic obstacle or barrier) rather than group behavior [169,380]. At Carreras Pampa, the numerous trackways and diverse morphotypes suggest that the trackways were not produced by a single individual passing at different times, but rather by multiple individuals of different species walking in roughly the same direction over a short period. The bimodal orientation of the trackways, toward the N-NW or S-SE, and the different morphotypes indicate the passage of a large group of individuals of various species in both directions.

A bimodal orientation of trackways has also been noticed in theropod *Eubrontes* trackways in the Dinosaur Footprint Reservation, Holyoke, Massachusetts, where the trackways show parallelism [381]. A bimodal pattern of parallel trackways is considered strong evidence for shoreline-paralleling behavior. However, although the parallel trackways suggest shoreline-paralleling behavior, it does not prove that such social behavior occurred because, as Lockley [315] pointed out, a shoreline would constrain groups and individuals to walk in the same direction. Laporte and Behrensmeyer [253] observed vertebrate tracks along the present-day damp strand line of Lake Turkana. They noticed that “[t]racks are most evident in this relatively narrow zone of moist, vegetation-free sediment,” a zone which, according to those authors, is of the order of only “up to several tens of meters wide.” Lockley [265] affirms that “such an observation suggests that tracks are very useful indicators of specific environmental zones,” including shoreline zones.

The criterion of regular spacing between the trackways cannot be applied here due to the high number of trackways, many of which intersect at a low angle at some points (Figs 21–28). Barco et al. [197] correctly point out that two other factors influence the inter-trackway distance: the size of individuals and the pattern of group movement (were they traveling as a large or small group moving in several waves?). Deviating trackways indicate that the trackmakers were not strictly confined to a passage by any physical barrier, except for the shoreline [339]. Therefore, most trackmakers walked in parallel or semi-parallel directions to the coastline, with few trackmakers deviating from that pattern.

The criterion of uniform depth of the tracks may not be applied at the Carreras Pampa tracksite, where some trackways show various styles of preservation, including shallow, deep, and very deep tracks. In the past, the Carreras Pampa area was likely a vast site of dinosaur transit; only a fraction is now exposed, yet it displays sectors with different rheological conditions (soft/firm).

Deviating trackways indicate that the trackmakers were not strictly confined to a passage by any physical barrier. Most trackmakers walked in parallel or semi-parallel directions, with few straying from that pattern. Paleocurrent indicators suggest that the general direction of sand transport was from south to north, indicating a general slope to the north. This slope may have influenced the direction of animal movement.

The criterion of uniform depth of the tracks may not be applied at the Carreras Pampa tracksite, where some trackways show various styles of preservation, including very shallow, shallow, deep, and very deep tracks. In the past, the Carreras Pampa area was likely a vast site of dinosaur transit; only a fraction is now exposed, yet it displays sectors with different rheological conditions (soft/firm).

The time interval for the theropod group or groups was limited. However, the occurrence of parallel trackways in different styles of preservation and varying depths of the prints indicates that not all the tracks were made during the same event. Furthermore, the overlap of tracks in different directions suggests that individuals walked on the substrate in more than one episode, although these episodes were close in time. The overlapping tracks indicate that the substrate’s rheological conditions were suitable for preserving the tracks during trackmaking.

In conclusion, the high number of trackways running parallel or semi-parallel, along with similar geographic orientations at the Carreras Pampa tracksite, strongly suggests that one or more groups of dinosaurs moved together or were separated by a short time. Eleven trackways indicate that the trackmakers were running. The group(s) may have shared a common purpose, showing gregarious behavior.

## Swimming behaviors

The abundant swim tracks were made by the theropods, which nearly floated and swam while kicking the soft bottom with the tips of their toes. The presence of swim tracks impressed upon walking tracks indicates that the latter formed first, and the swim track producers swam later in the same area. The common orientation of the solitary swim tracks and trackways (Fig 29K) may relate to group or social behavior. The alignment of the swim tracks to the shoreline inferred from the wave ripples suggests that the trackmakers were swimming slightly parallel to the shore. The reason for this behavior is unknown.

In wading depth, theropod traces would appear morphologically similar to tracks made on subaerial substrates because the trackmaker experienced minimal buoyancy [273]. As the water depth increased, leading to greater buoyancy, the tracks would gradually be replaced by swim traces, consisting of scratches made when only the tips of the autopodia touched the bottom. Some ‘intermediate’ or ‘hybrid’ traces would indicate this transition. In the tracksite, no ‘hybrid’ traces exhibit features of the change from walking to swimming. Therefore, the presence of swim traces suggests that the water depth in the Carreras Pampa area at that time must have been roughly equivalent to the hip height of the theropod trackmaker [271]. The thick, massive marls, gastropod shells, and desiccation cracks support a low-energy, nearshore depositional scenario.

## Tracked Substrate

Symmetrical wave ripple bedforms are present in many areas of the tracksite (Figs 2B and 6A), but not within footprints or swim tracks, suggesting that the measured walking and swim tracks formed after oscillatory reworking of the surface occurred. The formation of ripples, burrows, and tracks indicates that the substrate was deformable, not thixotropic, and cohesive enough to maintain track shapes as described. However, the presence of tracks in various preservation styles, including very deep (penetrative) and very shallow tracks, indicates a polyphasic substrate with changing water saturation and substrate strengths. The occurrence of various preservation styles, with deep and very shallow tracks within the same trackway, indicates that the firmness of the substrate varied across the surface even while a single trackway formed [43]. More specifically, the various preservation styles indicate that the substrate’s three key properties—viscosity (plasticity), consistency (cohesion), and adherence—were not uniform at any given time.

Some trackways have missing tracks, indicating that the substrate was too rigid and the trackmaker was too light to leave tracks, a characteristic observed especially in trackways consisting of small tracks. Moreover, some trackways are characterized by various modes of preservation, including shallow tracks of mode M3 and deeper tracks of modes M4 and M5, all within a single trackway.

In addition to the walking tracks with various preservation modes, swim tracks also mark the same surface and include some swim traces that cut across tridactyl tracks. This stratigraphic relation indicates that the substrate preserved walking track impressions even while water levels rose enough for dinosaurs to swim. Sediments remained deformable enough for swim tracks to form on the same surface as the true tracks. Swimming trackways and isolated, shallow swim tracks were created by individuals who paddled and scratched the substrate.

A fossil footprint is a record of a brief interaction between the trackmaker and the substrate, and its preservation represents a very narrow window of time recorded in the rock [88]. The preservation of small and miniature theropod tracks, bird tracks, rosette burrows, swim tracks, and displacement rims in bird tracks and swim traces strongly indicates that erosion or disturbance of the ichnites by waves, water currents, or wind was minimal or absent, and time averaging was also minimal. Vertebrate tracks are generally not preserved along present-day shores or foreshore environments of lakes, lagoons, the sea, or on riverbanks because waves, longshore currents, and winds continually move the sediment particles at the sediment-air or sediment-water interface [253].

### Timing of events associated with track formation

Based on the ichnological association of tracks and traces, we can reconstruct the timing of events that left the complex assemblage of ichnites in the tracked layer:

1) Analysis of sedimentary structures in the oolitic, ostracod-rich arenite CP bed shows initial transport and emplacement through migrating troughs, small channels, and bars. At a time when approximately the first third of this sandstone accumulated, an older set of tracks was made and preserved in some parts of the Carreras Pampa tracksite. Where preserved, the first generation of tracks is often associated with a parting surface or laminae of clay-rich sediment (Fig 10). These tracked areas were not affected by subsequent deposition, which continued the channel and bar deposition until the entire thickness of the CP bed accumulated. The upper 5–10 cm of the CP bed was subsequently reworked by oscillatory flow, indicating that the tracked surface was submerged before the observed tracks were produced. The water level then dropped, exposing nearly all of the Carreras Pampa surface on which dinosaurs had walked.
2) Theropod dinosaurs and birds walked on the surface of the upper track layer, leaving prints of various preservation styles and morphotypes, as well as tail traces. The rheological properties of the substrate were not uniform over space and time, resulting in both shallow and deep tracks within the same trackways. This track variability suggests lateral variations in substrate water saturation and changing water saturation conditions over time.
3) The substrate was burrowed by invertebrates, creating *Hillichnus* and rosette-like burrows along with surface galleries that slightly altered the quality of preservation of some tracks without modifying their morphology, size, digits, or hallux impression. The occurrence of theropod tracks shows impressions of the claws, the hallux, and the sharp contours of the digits. At the same time, the well-preserved avian prints indicate that both erosion and extensive modification by invertebrates were minimal. Minimal padding and no skin impressions were observed, likely because they were not recorded due to the coarser grain size of the sandstone substrate. Invertebrate bioturbation within the tracks suggests that the substrate remained workable for invertebrates after the tracks were made.
4) The water level rose without eroding the ichnites and rosettes surrounding the burrows in the tracked layer. The preservation of ichnites is explained by incipient calcite cementation that stabilized grain contacts but remained weak enough to be deformed by contact with swimming dinosaurs. Patchy occurrences of calcite mud within the substrate may have also contributed to the stabilization of the sediment. The water level rose shortly after the theropod and avian tracks were produced. This timing was essential to preserve structures from modification by wind, rain, waves, or currents and before extensive invertebrate burrows could alter them.
5) Before the swim tracks were made, a single layer of a monospecific assemblage of small gastropods was deposited on the substrate, covering most of the submerged area, including tracks.
6) The presence of abundant swim traces is relevant in reconstructing the paleoenvironment of the Carreras Pampa at the time of ichnite formation and preservation. Some swim traces cut across walking tracks, indicating that the latter formed first and that the sediment was not consolidated when the swim traces formed. During periods of high water level, while the bottom sediment was deformable, many theropod dinosaurs paddled under conditions of high buoyancy, with their digits barely touching the bottom and leaving elongated scratches on the substrate, which formed swim trackways. The occurrence of solitary swim tracks likely resulted from changes in buoyancy or the relief of the substrate.
7) Mudstones abruptly overlie the tracked surface, suggesting deposition in profundal water conditions. Mudstone accumulation may have also inhibited further bioturbation, aiding in track preservation. These conditions persisted at Carreras Pampa until 3 m (compacted thickness) accumulated (Figs 2 and 10).
8) The excellent preservation of the swim tracks with expulsion rims, the previously formed theropod and avian tracks, and the exquisite preservation of the tail traces suggest that incipient cementation occurred rapidly after the tracks were made. This is further supported by the absence of bioturbation altering the swim traces.

### Depositional environment of the CP bed

No single paleoenvironmental interpretation explains the entire El Molino Formation. It comprises a wide variety of clastic and carbonate lithologies, and internal correlations have not been established to allow for lateral comparisons. These complexities and limitations make it difficult to compare conclusions from studies in different areas and stratigraphic levels. Some authors interpret the sedimentary succession of the El Molino Formation as primarily non-marine lacustrine, with periodic outflow to marine waters [31,33]. In contrast, others interpret it as deposits from a lacustrine system with periodic marine inflow [14,28,32,305,382,383]. The data used to support either model are based on the values of δ^18^O and δ^13^C, as well as the presence of terrestrial, freshwater, and marine fauna [28,31,306–308,382,384–386]. Based on paleontological and sedimentological data, Camoin et al. [34] suggested a lacustrine environment based on many freshwater taxa, although later Gayet et al. [387] argued the data and interpretation were “in part incorrect and one-sided”. Palma [388] proposed a lacustrine environment for the Yacoraite Formation in northwestern Argentina, equivalent to the El Molino Formation in central Bolivia, a proposal later disputed by Gayet et al. [387]. The paleontological data do not support an environment of continuous continental deposition, but rather repeated incursions of marine species and freshwater fish taxa transported by rivers into the basin [28,305,385].

So far, the evidence is inconclusive, but as Fink and others have suggested, the presence of marine fauna indicates that the El Molino Formation layers were deposited near or at sea level, rather than in an open-sea environment [29,306,308,387]. The Pajcha Pata fossil locality, approximately 30 km northeast of Carreras Pampa, has yielded both freshwater and a diverse array of marine taxa, strengthening the hypothesis of a lacustrine environment with marine influence [28,387]. The more abundant marine taxa than freshwater taxa indicates a strong influence of the marine incursions in this proximal setting, something to be confirmed in the more distal parts of the El Molino Basin. Based on the mixed continental taxa (anurans, reptiles, and mammals), freshwater taxa (Caudata, anurans, and some fish), and numerous and diverse marine taxa (fish), Gayet et al. [308] concluded that the depositional environment was probably lagoonal or estuarine. Later, Gayet et al. [305, p. 63] asserted that “the same marine fauna [of fossil fish] lived in the whole [El Molino] basin.”

The occurrence of the mandible of the osteoglossoform fish is not determinant of the paleoenvironment. Today, except for *Hiodon*, which is found only in North America, all the other extant osteoglossiform fish dwell exclusively in tropical and subtropical freshwater environments in the southern hemisphere, including Southeast Asia, Africa, South America, Australia, and the tropical Pacific Ocean islands. Some species of recent freshwater osteoglossiformes are thought to tolerate brackish waters and sometimes enter marine (estuarine) environments [389]. Their fossil representatives were widely distributed on both the northern and southern continents in freshwater deposits from the Barremian to the Eocene. In contrast, marine fossil osteoglossiforms have been identified in Palaeocene and Eocene rocks in Europe, North America, Central Asia, and Africa [390–393]. In Bolivia, Gayet et al. [305] and Gayet [308] report the osteoglossiform fish *Phaerodusichthys taverni* and other freshwater and marine fish in the upper Maastrichtian, El Molino Formation site of Pajcha Pata north of Torotoro, which has yielded a variety of invertebrates, plants, and vertebrates, including dinosaurs, reptiles, mammals, amphibians, and terrestrial and marine ichthyofauna.

The mudstones separating the sandstones were deposited in a freshwater lacustrine setting. This interpretation is supported by their fine-grained size, freshwater algal palynomorphs, and charophytes. Transitions between sandstones and mudstones are sharp, suggesting abrupt base-level changes or autogenic processes. The tracked sandstone consists of transported carbonate and clastic grains. Depositional processes implied by lithology and sedimentary structures include tractional flow in small channels and migrating bars that transition to sand flats with final reworking by waves. Lateral changes in the widths of troughs imply an anastomosing distributary complex, with paleocurrent indicators showing transport generally to the north. Wave ripple bedforms indicate that the upper portion of the CP bed was not cemented at that time and was submerged; therefore, wave ripples likely formed as the lake level fell. Local variation in wave ripple crest orientation implies the influence of local shoals or interference caused by nearby shorelines. Shallowing water and subaerial exposure of Carreras Pampa are also consistent with burrow structures at the surface, vertebrate tracks, and increased alkalinity. Since no significant amounts of clay minerals were present in the sandstone and no indications of microbialite mats were observed, the sediments were likely stabilized by local accumulations of calcite mud and early incipient cement. Stabilization of the substrate is indicated by the preservation of invertebrate and vertebrate ichnofossils throughout the unit, as well as well-preserved vertebrate tracks on the upper surface of the bed. Moderately packed textures facilitate the early stabilization of grain packing before significant grain rearrangement occurs. Pervasive calcite cements and the absence of sulfate minerals indicate that pore waters were saturated with respect to calcite, suggesting a lack of magnesium ions, which is consistent with lacustrine settings. The rheological conditions and early, variable cementation can explain the variety of track types and depths observed in theropod tracks.

## Conclusions

The Carreras Pampa tracksite in Torotoro National Park, Bolivia, is an extraordinary assemblage of dinosaur and avian ichnites, theropod swim tracks, tail traces, and various invertebrate burrows. Several lines of evidence suggest that theropods created both walking and swim tracks. The tracksite boasts several worldwide records, including the greatest number of exposed dinosaur tracks (16600), theropod tracks, tail traces, and swim tracks preserved in trackways. It also features many avian tracks associated with the dinosaur tracks. The swim tracks, tail traces, and avian tracks are remarkably well preserved, and most tracks are found in continuous trackways. 1321 trackways, consisting of two or more successive tracks, along with 289 solitary tracks, were discovered across all the studied sites. The abundance and exceptional preservation of these tracks and traces make the Carreras Pampa tracksite an ichnologic concentration and conservation Lagerstätte.

Multiple lines of evidence indicate that the tracks are genuine and not undertracks, and therefore were made on the original surface. We distinguished eight different styles of preservation, ranging from very shallow, claw-only tracks to very deep prints, many of which feature hallux impressions, posterior extensions, or raised rims. Tail traces mainly occur with very deep tracks, although a few are associated with shallow footprints. Most tracks are small to medium in size, displaying strong mesaxony and a total divarication of 45° − 70°. The claw prints are often continuous with the digits. Very few tracks exhibit a bourrelet or expulsion rim, which typically appears on the exterior side of the tracks.

The tracksite has the highest number of dinosaur tail traces worldwide, most of which are associated with deep and very deep tracks. The tail traces appear as curved or sinuous grooves on the posterior edge of the tracks, extending from the anterior edge of the tracks, or connecting two tracks. These traces are well preserved. Many tracks have a straight raised posterior ridge, and a few have an anterior groove out of the tip of digit III. Tetradactyl tracks with hallux impressions are common in both shallow and very deep prints. We distinguished three different morphotypes of hallux impressions.

Due to the large number of tracks and trackways, the location offers an exceptional opportunity to infer that the measured and derived attributes are characteristic of the population present. Mean values should represent the average for the population, while extreme values likely indicate actual extremes. The trackways display a distinct trend in orientation, with most trackways falling into a bimodal grouping oriented NNW-NNE (300°–30°) or SSE (120°–180°), and a few trackways oriented approximately SW (181º–255º). The Carreras Pampa tracksite was once a hub of high dinosaur activity. Aside from tracksites with very small vertebrate footprints and miniature dinosaur tracks, the Carreras Pampa tracksite may boast one of the highest densities of theropod tracks known worldwide. The abundant parallel and subparallel trackways, along with the bimodal orientation, strongly suggest the presence of some form of group behavior. The overlapping and crossing of trackways indicate that groups of theropods walked at different times in both preferred orientations.

Our data follows the general pattern for theropod trackways in that relative stride lengths below SL*/h* = 2.0 were the most common. However, a significantly higher percentage of the trackways at Carreras Pampa exhibit values above SL*/h* = 2.0 than is commonly observed at other tracksites. The estimated speeds from the trackways are also greater than those typically found at other tracksites, suggesting that the trackmakers at Carreras Pampa moved faster on average than at these other sites. Relative stride length values exhibit a unimodal distribution, with no peaks within the previously proposed gait values. This supports previous suggestions that theropod dinosaurs did not have discrete gaits. Most trackways are straight, but sinuous trackways are also common. Some trackways show stops and turns exceeding 45°, reflecting a population where most dinosaurs continued straight, but a few, even on the same surface, turned and stopped.

We identified eleven track morphotypes based on morphological traits and the orientation of the digits and heel impressions. The hallux impression is relatively common in both shallow and deep tracks, showing three distinct morphotypes. Most (82%) of the measured tracks (n = 1146) have a foot length ranging from 16 to 29 cm, classified as small to medium, with small tracks closely resembling the medium range. Most (80%) of the trackmakers had a hip height between 65 cm and 115 cm, with a greater percentage in the 75–105 cm range. Notably, very few trackmakers exceeded a height of 125 cm.

Theropod footprints with tail-drag traces are abundant and well-preserved in the tracksite, appearing in trackways featuring shallow, deep, and very deep tracks. The tail traces suggest that dinosaurs exhibited some form of locomotive behavior in response to sinking into soft substrate, which resulted in their tails coming into contact with the surface. However, the presence of tail traces associated with shallow tracks indicates that some mechanism, aside from sinking into the substrate, also contributed to the formation of tail traces in certain instances.

Swim tracks are abundant at the tracksite, with many forming trackways consisting of sets of 1–3 scratches that alternate between right and left with regular spacing. The traces are in excellent preservation condition, and we identified three different morphotypes. Some swim tracks cross-cut walking tracks, and most swim trackways are oriented toward the SSE. The swim tracks are tentatively assigned to the ichnotaxon *Characichnos* based on their morphological traits.

Avian tracks are found in several areas of the tracksite, with a notable abundance at site CP9, where over two hundred tracks are associated with theropod walking and swimming. The bird footprints are well-preserved and include several trackways.

A large area of the surface at site CP2 and parts of other sites are covered with an assemblage of monospecific, very small (1 cm) spiral gastropods. The shells are scattered randomly rather than oriented. In some areas, the shells are eroded, leaving a slight lenticular concavity on the surface of the substrate. A low-diversity invertebrate trace fossil assemblage is found on the upper surface of the trackbearing layer, including three types of burrows: Hillichnus burrows, radial/rosette burrows, and various holes (vertical holes?). The rosette burrows are very abundant (60 − 65/m^2^).

## Supporting information

S1 Table Dimensions of the studied tracks and trackways in the Carreras Pampa tracksite. where the CP trackbed is exposed in cross-section. (XLSX)

S1 Fig Aerial views of the Carreras Pampa sites. **A)** View of the Carreras Pampa tracksite from the north. The white lines indicate the areas where the CP bed is exposed in cross-section. B) View of site CP1. Note several conspicuous trackways with deep tracks, characteristic of the preservation styles M4 and M5. The approximate surface area is 1890 m^2^. The black arrows indicate the trackways with deep tracks and tail traces. The green boxes indicate three areas where the tracked surface is weathered. C) View of site CP2. Note several conspicuous trackways with deep tracks, characteristic of the preservation styles M4 and M5. The approximate surface area is 2550 m^2^. The black arrows indicate the trackways with deep tracks and tail traces. The green box indicates three areas where the tracked surface is weathered. D) View of site CP3. Note several conspicuous trackways with deep tracks, characteristic of the preservation styles M4 and M5. A) A curved trackway of tracks of the styles of preservation M4 and M5 (dashed line). Trackways a, b, and c are of tracks from styles of preservation M4 and M5, with associated tail traces, The green box indicates an area of weathered substrate. The black arrows indicate the trackways with deep tracks and tail traces. The approximate surface area is 590 m^2^. E) View of site CP4. Note several conspicuous trackways with deep tracks, styles of preservation M4 and M5. The approximate surface area is 285 m^2^. F) View of site CP5. Note several conspicuous trackways with deep tracks, characteristic of the preservation styles M4 and M5. The black arrows indicate the trackways with deep tracks and tail traces. The green box indicates an area where the tracked surface is weathered. The approximate surface area is 415 m^2^. G) View of site CP6. Sites CP5, CP2 and CP1 are in the background. The green boxes indicate an area where the tracked surface is weathered. The approximate surface area is 334 m^2^. H) View of site CP7. The black arrows indicate the trackways with deep tracks and tail traces. The green box indicates an area where the tracked surface is weathered. The approximate surface area is 1120 m^2^. I) View of site CP8. The green box indicates an area where the tracked surface is weathered. The approximate surface area is 41 m^2^. J) View of site CP9. The black arrows indicate the trackways with deep tracks and tail traces. Two people stand for scale. The green box indicates an area where the tracked surface is weathered. The approximate surface area is 312 m^2^.
(PDF)

S2 Fig Sedimentary characteristics of the tracked bed in cross-section. A) Cross-section of CP bed showing wavy and sub-horizontal lamination, trough cross-lamination, and wave ripple cross-lamination. B) Cross-section of CP bed showing trough cross-lamination at a scale of tens of centimeters and wave ripple cross-lamination at the top. C) Typical appearance of the red bed (RB) outcrop showing rectangular and resistant blocks. D) Cross-section of the red bed (RB) showing characteristic ripple trough and low amplitude trough cross-lamination. Laminae are enhanced by the weathering residues of quartz grains that follow cross-lamina surfaces. E) Wave ripple bedforms on the upper surface of the wave ripple (WR) bed. Many of the ripples have sharp crests with horizontal burrow traces that follow the ripple crest. F) Pseudomorphs of evaporite crystals exposed by weathering and characteristic of bed evaporite bed (EB). G) Typical appearance of silty claystone units. The resistant bed at the top of the outcrop is the EB unit.
(PDF)

S3 Fig Tracks and trackways of sty le of preservation M1. A) Trackway T22-128. Small track with the claws well marked and the metatarsophalangeal area absent. B) Trackway T22-443. One track of the style of preservation M1. C) Trackway T22-483. D) Trackway T22-258. This trackway consists of three tracks (L1, R1, L2) preserved as indentations of the claws at the northern side of site CP1. R1 shows two linear, deep, and very narrow traces of digits III and IV, with digit II consisting of only a tiny scar. L2 exhibits comma-shaped, deep, and narrow markings for digits II and IV, and a longer marking for digit III. No other morphological details are preserved. E) Trackway T22-2–393. F) Trackway T22-483. G) Trackway T22-449. H) Trackway T22-496. I) Trackway T22-2–130. J) Trackway T22-2–293. Trackway with two tracks of the style of preservation M1. There is a well-preserved rosette-like burrow (rb) next to the tip of digit III of the R1 track. K) Trackway T22-2–494. Three tracks of the style of preservation M1. L) Trackway T22-2–503. Three tracks of the style of preservation M1. M) Trackway T22-2–392. This trackway consists of tracks of the style of preservation M1, except the last three tracks, which are in the style M3 (not shown here). O) Trackway T22-2–392. This trackway consists of tracks of the style of preservation M1, except the last three tracks, which are in the style M3 (not shown here). P) Trackway T22-2–36. Trackway with eleven large tracks of the style of preservation M1, consisting of three deep, conical indentations. R2 shows the shallow contour of the track and a shallow indentation at the posterior end of the impression of the heel. The scale in A-C, E, F, G, H, K and M is 20 cm; in J is 50 cm; in D, L and O is 1 m; in I and P is 10 cm.
(PDF)

S4 Fig A-F) Tracks and trackways of styles of preservation M2 and M3. A-B) Trackway T22-423. C) Trackway T22-2–500. Digit IV is not impressed in this track. D) Trackway T22-2–497. This trackway is 3.22 m long with four tracks of the style of preservation M2. There is a turn of 22 degrees in the direction of movement at R1. The scale is in cm, 1 m in the large photograph and 20 cm in the small photographs. E) Trackway T22-2–500. One track of the style of preservation M2. Digit IV shows raised sediment. F) Trackway T22-2–415. Trackway 1.9 m long with six tracks of the style of preservation M2. G-I) **Tracks and trackways of the style of preservation M3**. G) Trackway T22-236. Three tracks of the style of preservation M3. H) Trackway T22-2–123. I) Trackway T22-2–258. J) **Tracks of the styles of preservation M2, M3 and M4.** Trackway T22-2–3. Very long trackway with nineteen tracks (R5 missing) of the preservation styles M2, M3 and M4. K) Trackway T22-2–6. Trackway with four tracks of the styles of preservation M2 and M3. The scale in D is 1 and 20 cm in the three small photos; in F is 1 m; in A-C, E and G is 20 cm and in H-K is 10 cm.
(PDF)

S5 Fig Tracks and trackways of the style of preservation M4 and M5. A) Trackway T22-79. Trackway with many deep tracks of the styles of preservation M4 and M5. Some have the mark of the hallux (white arrow). B) Trackway T22-72. Most tracks of this trackway are of the style of preservation M4, well-marked and with hallux impressions (white arrow). Several of them have a raised posterior ridge (indicated by the yellow arrow). C) Trackway T22-2–21. Tracks of the style of preservation M4. The trackway has ninety-nine small tracks, and one is missing (R20). The impression of the hallux is indicated with a white arrow. Notice padding preserved in track R30. D) Trackway T22-2–47. Very long trackway with small tracks of the styles of preservation M3 and M4. The heel impression is poorly marked, acuminate or narrowly round. Some claw marks are oriented backward (R1, R5, L6, R7, L9). The scale in A and B is 20 cm; in C and D is 10 cm.
(PDF)

S6 Fig Tracks and trackways of style of preservation M5. A-B) Trackway T22-102. Trackway with tracks of styles of preservation M4 and M5. Most of this trackway’s tracks are well-marked and have hallux impressions (white arrow). Several tracks have a raised posterior ridge (yellow arrow). The left (L) track in the middle part of photo B shows a posterior groove instead of a raised ridge. C) Trackway T22-2–25. Very long trackway with one hundred three very deep tracks of the style of preservation M5. Several tracks have associated tail traces. The scale in B is 20 cm and in C is 1 m.
(PDF)

S7 Fig Trackway T22-180. This trackway consists of shallow depressions of the style of preservation M6 that we interpret as the filling of actual tracks occurring in a sedimentary layer below the surface. Most of these depressions are elongated with both ends round; some show a narrowing in the middle, and others are slightly curved or round. The scale is 1 m.
(PDF)

S8 Fig Tracks in cross-section. A) Track CP3−1. Deformation in two different layers corresponding to two different tracks (white arrowheads). B) Track CP3−3. C) Track CP7CS2. D) Track CP7CS16. E) Unnamed track in cross-section. F) Track TPCS24−2. G) Track TPCS24−3. The scales are in cm.
(PDF)

S9 Fig Trackways CP9–79, 80, 81, and 82. Trackways CP9–81 (orange) and CP9–82 (green) have tail traces associated. Notice that the tail traces cut through the tracks. The arrows indicate the direction of movement. The scale is 1 m.
(PDF)

S10 Fig Pairwise comparisons of proximal depth (PD) (A), distal depth (DD) (B) and foot length (FL) (C) using the Tukey Honest Significant Difference (HSD) test with a False Discovery Rate (FDR) correction (p = 0.05). (DOCX)

S11 Fig Swim tracks and trackways. A-B) Swim trackway CP5-S2. Eight sets of swim traces, with L3 missing. The first five sets are placed along the red yarn line and indicated with black arrows. Two additional swim traces occur near the distal end of the ruler. The scale is 1 m. The image on the right shows the first five swim tracks of the succession. C) Swim trackway CP5-S3. Eight sets of swim traces of morphotype S1. Several other swim trackways and isolated swim sets are associated. The scale is 1 m. D) Swim trackway CP5-S4. Four sets of swim traces. Several additional swim traces occur. Sets R1 and L1 consist of single traces of digit III of morphotype S2, whereas sets R2 and L2 consist of three traces each corresponding to digits II, III and IV of style S1. The scale is 1 m. E) Swim trackway CP5-S8. Four sets of swim traces with several missing. Additional swim traces of CP5-S3, CP5-S6, CP5-S7 and CP5-S10 occur. All the swim traces are of morphotype S1. Notice a track of trackway CP5-T7 with a posterior ridge. The scale is 1 m. F) Swim trackway CP6-S1. Five sets of large swim traces of morphotype S1. The scale is 30 m. The measurements correspond to pace, stride length and WAP. G) Swim trackway CP6-S3. Twenty-three sets of large swim traces of morphotype S1. Total length of the exposed trackway is 31.50 m. The traces of this trackway have the largest expulsion rims in all the studied sites. Yellow-black rulers are 1 m long. H) Dimensions of the traces in the swim trackway CP6-S3. Twenty-three sets of exposed swim traces, beginning with R1 and ending with L12. I) Swim trackway CP7-S1. Six sets of small swim traces of morphotype S1. Other additional swim traces are associated with R1 and L1. Swim track L1 cuts across a previously formed theropod track. The scale is 1 m. J) Swim trackway on Site CP1. Long swim traces of morphotype S3. The scale is 2 m long.
(PDF)

S12 Fig Bird tracks. A) Two bird tracks associated with track L4 of trackway CP6–36 encircled in blue chalk. The two blue arched lines indicate two indentations of a left track of style of preservation M1. The scale is 30 cm. B) One bird track (white box) associated with track L15 of trackway CP6–36. Notice the small sinuous galleries resulting from the growth of plant roots. The scale is 30 cm.
(PDF)
