# Supplementary figures and images for: Morphotypes, preservation, and taphonomy of dinosaur footprints, tail traces, and swim tracks in the largest tracksite in the world: Carreras Pampa (Upper Cretaceous), Torotoro National Park, Bolivia
Source: PLoS One. 2025 Dec 3;20(12):e0335973. doi: 10.1371/journal.pone.0335973 (PMC12674571; doi:10.1371/journal.pone.0335973)

# Supporting Information S1 Fig

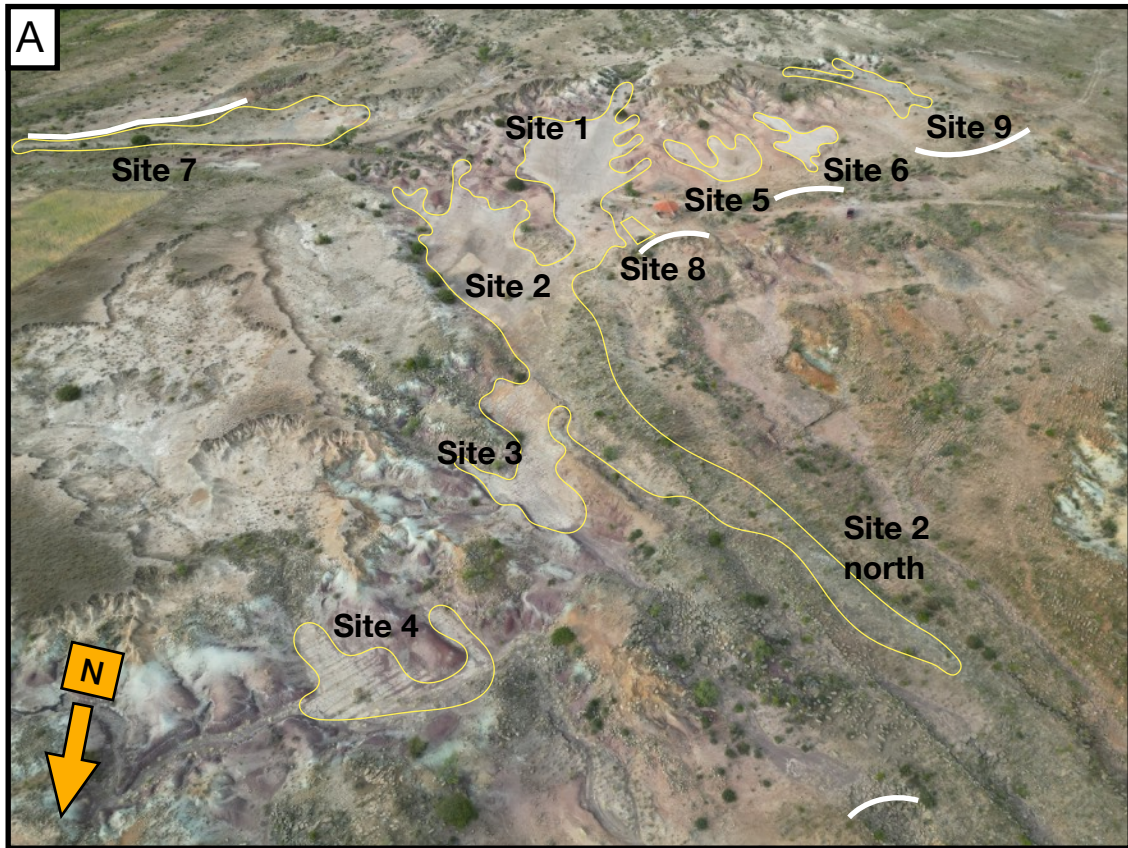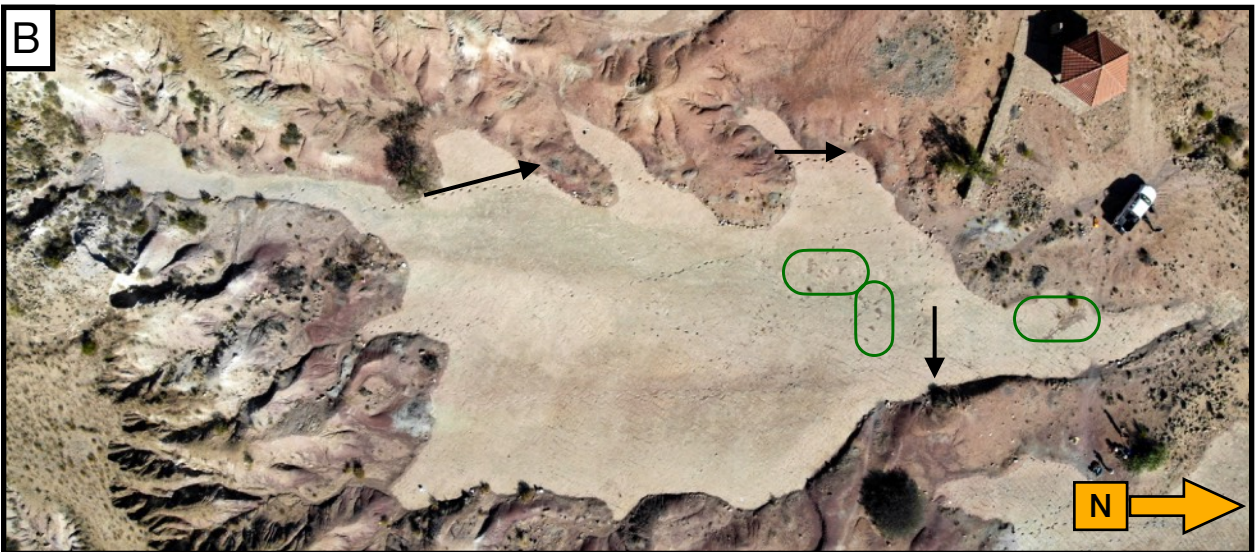

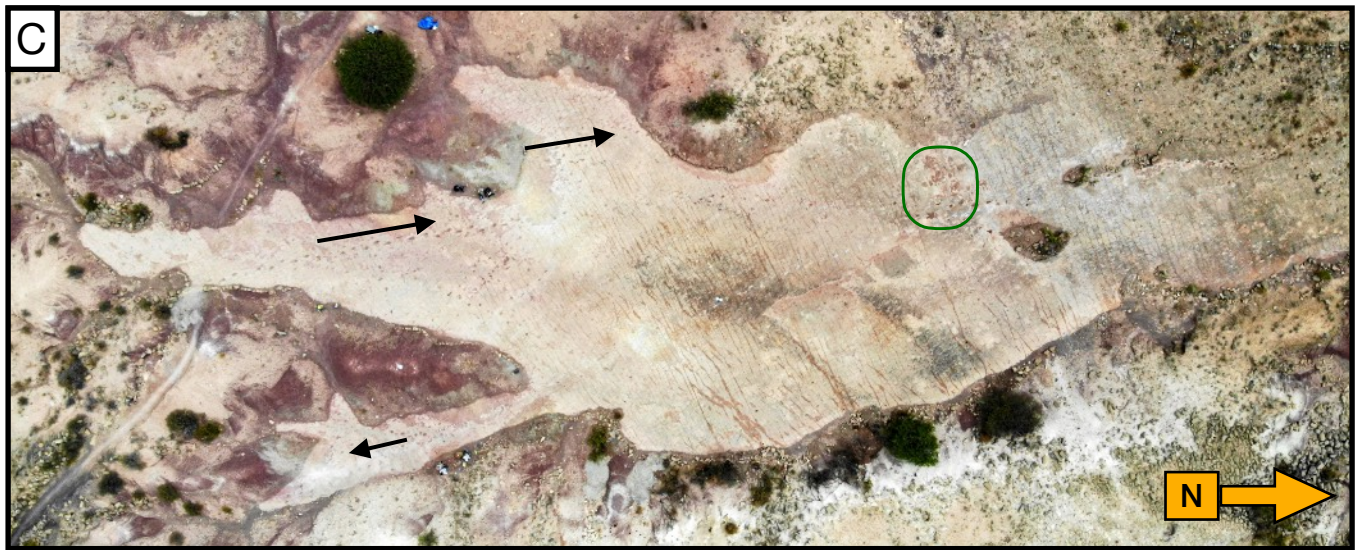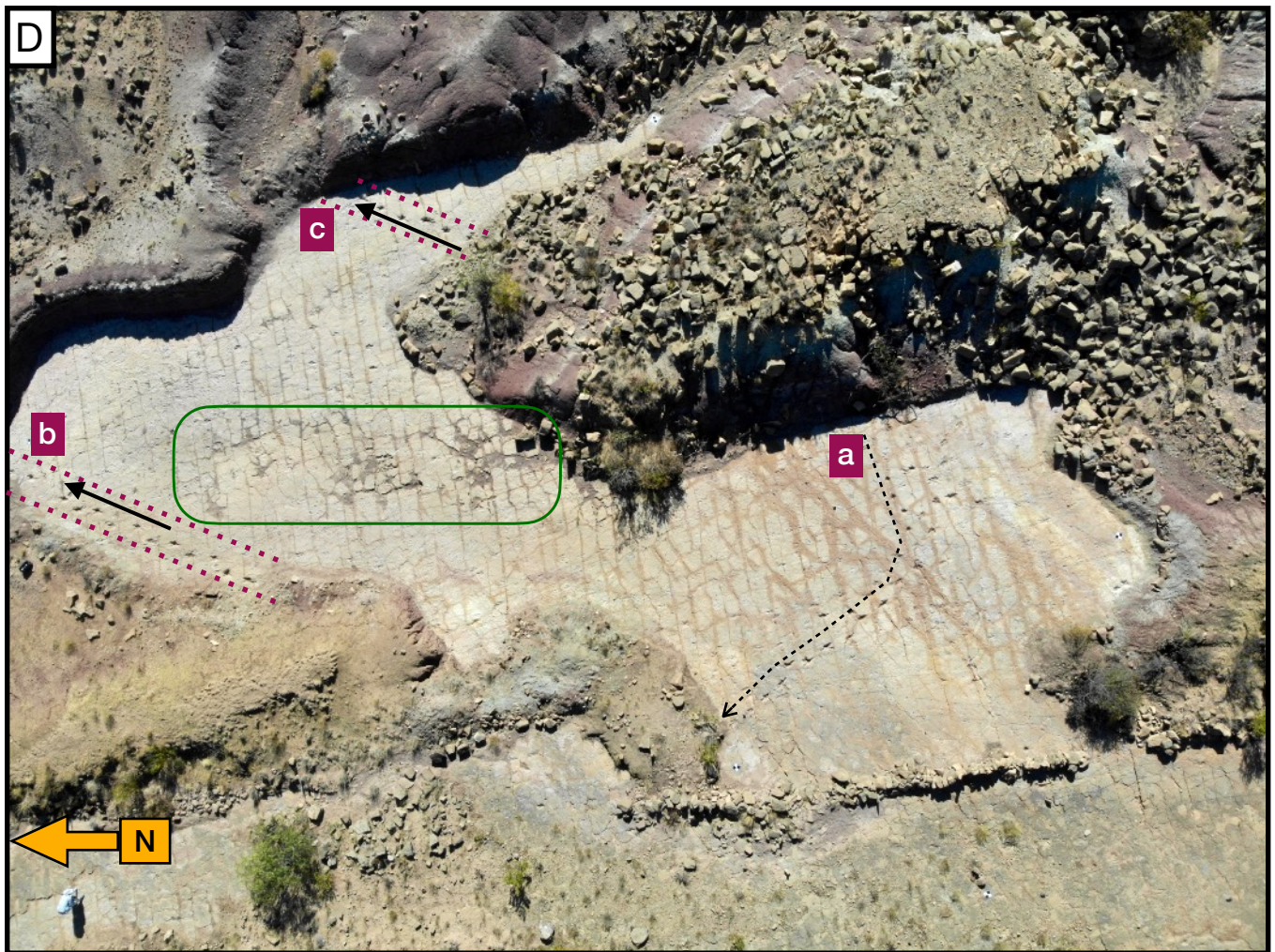

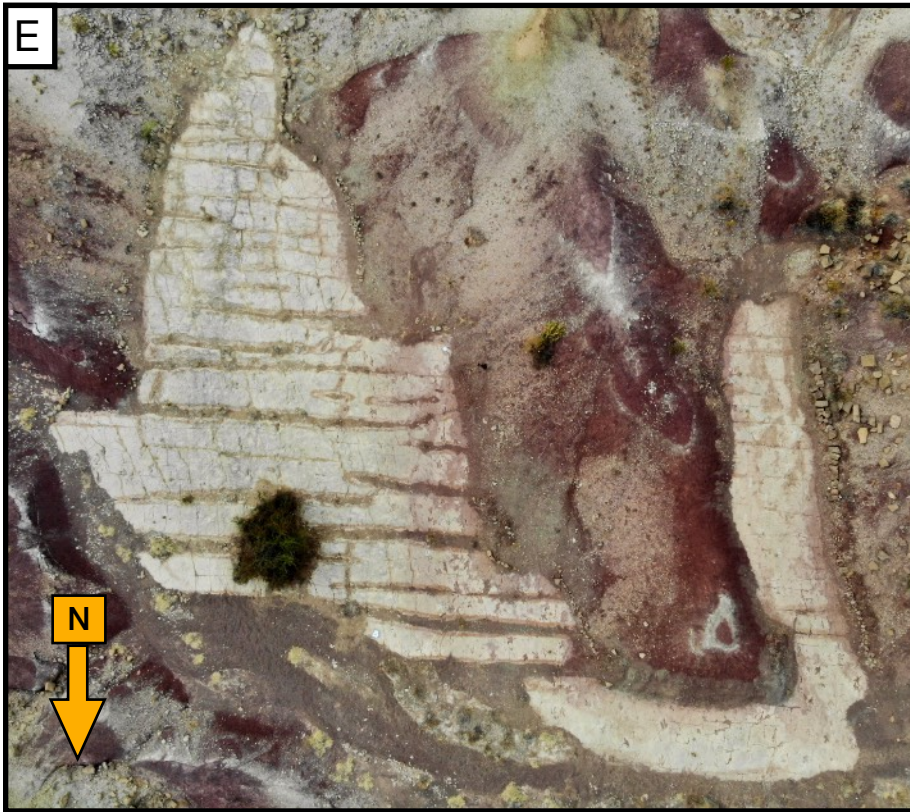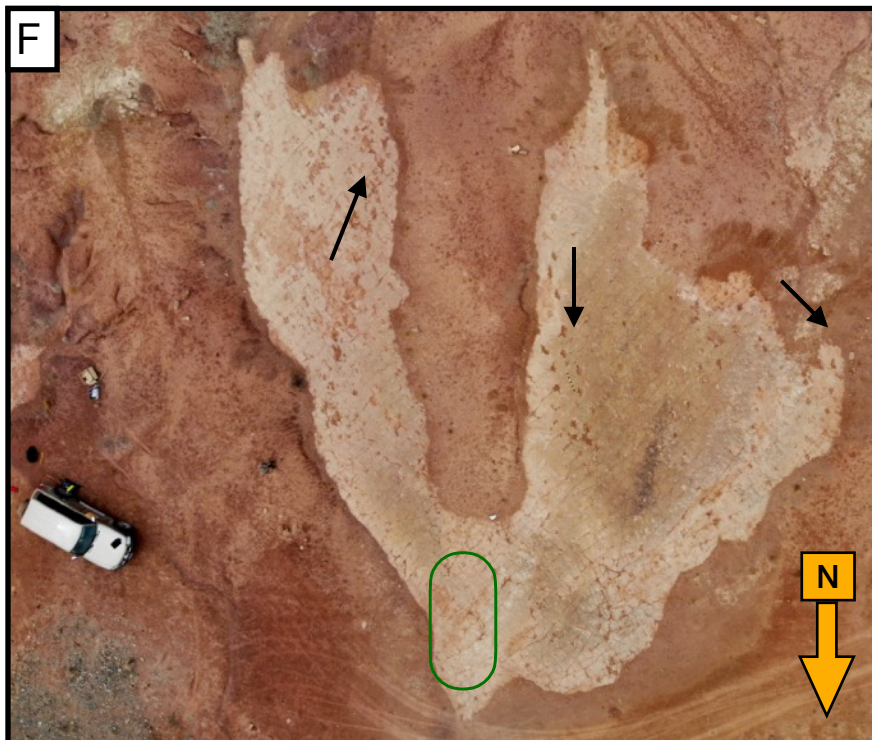

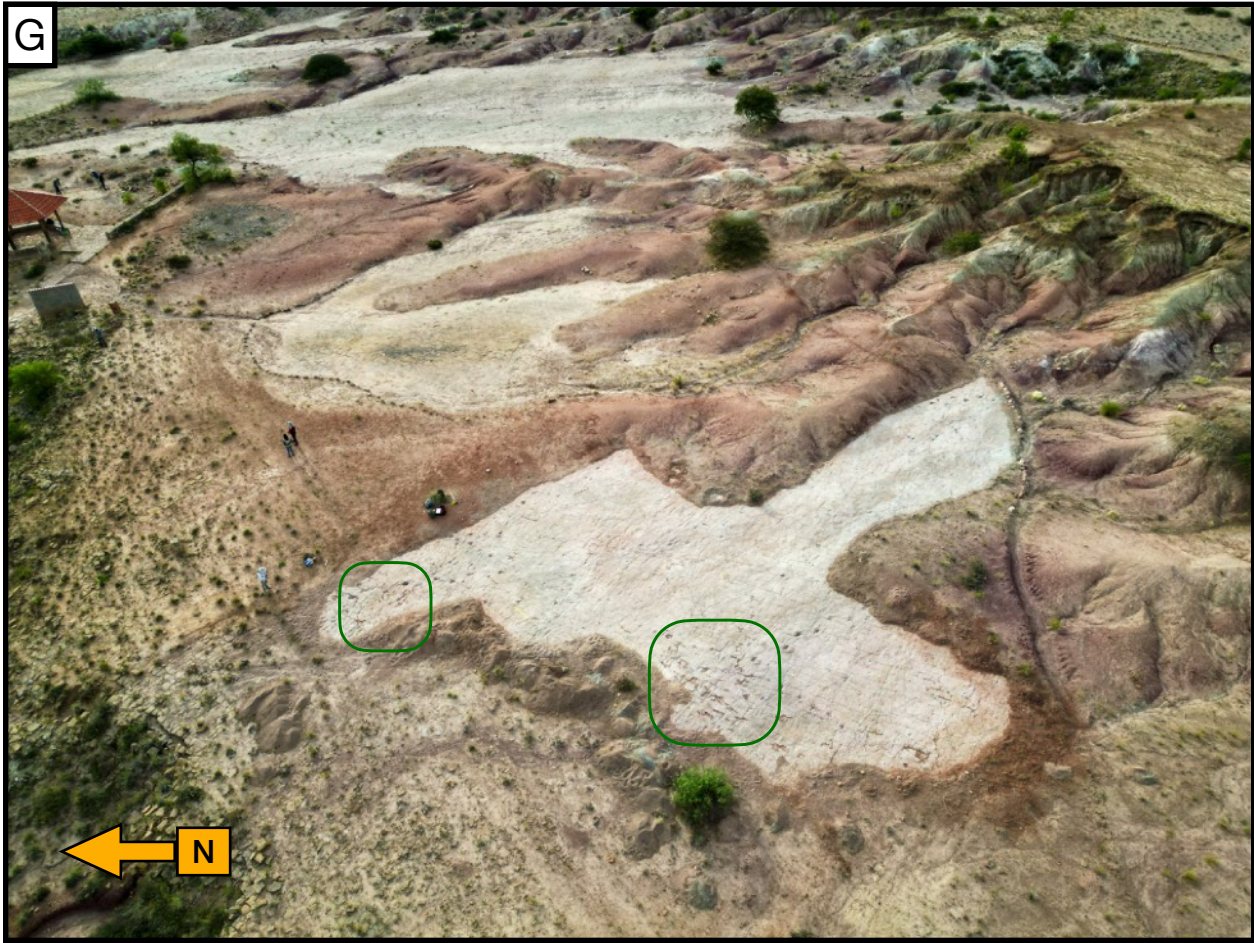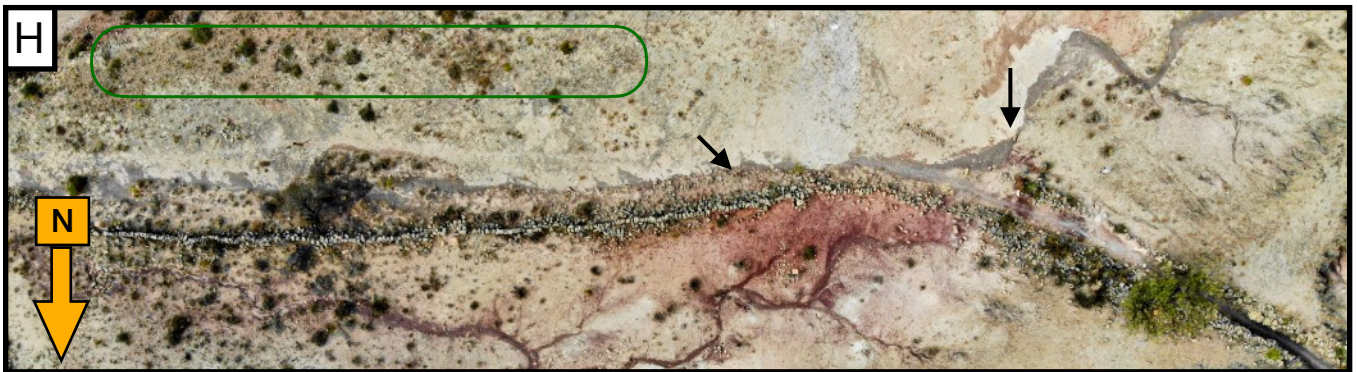

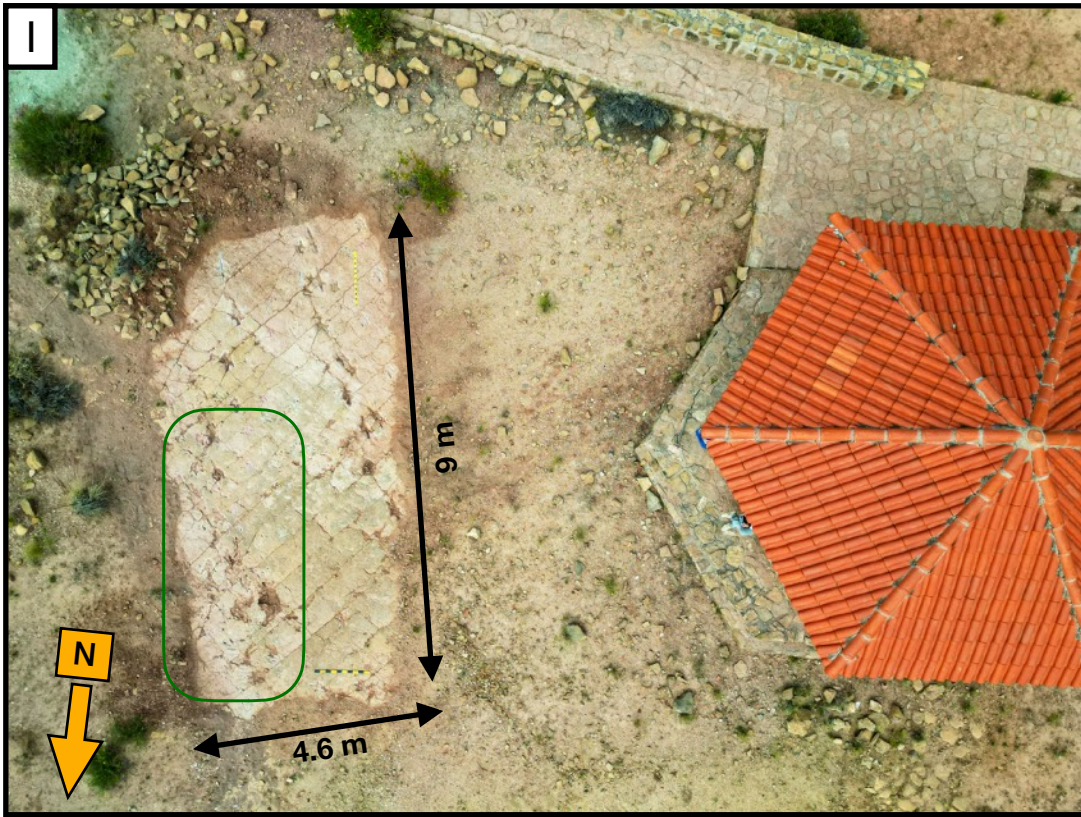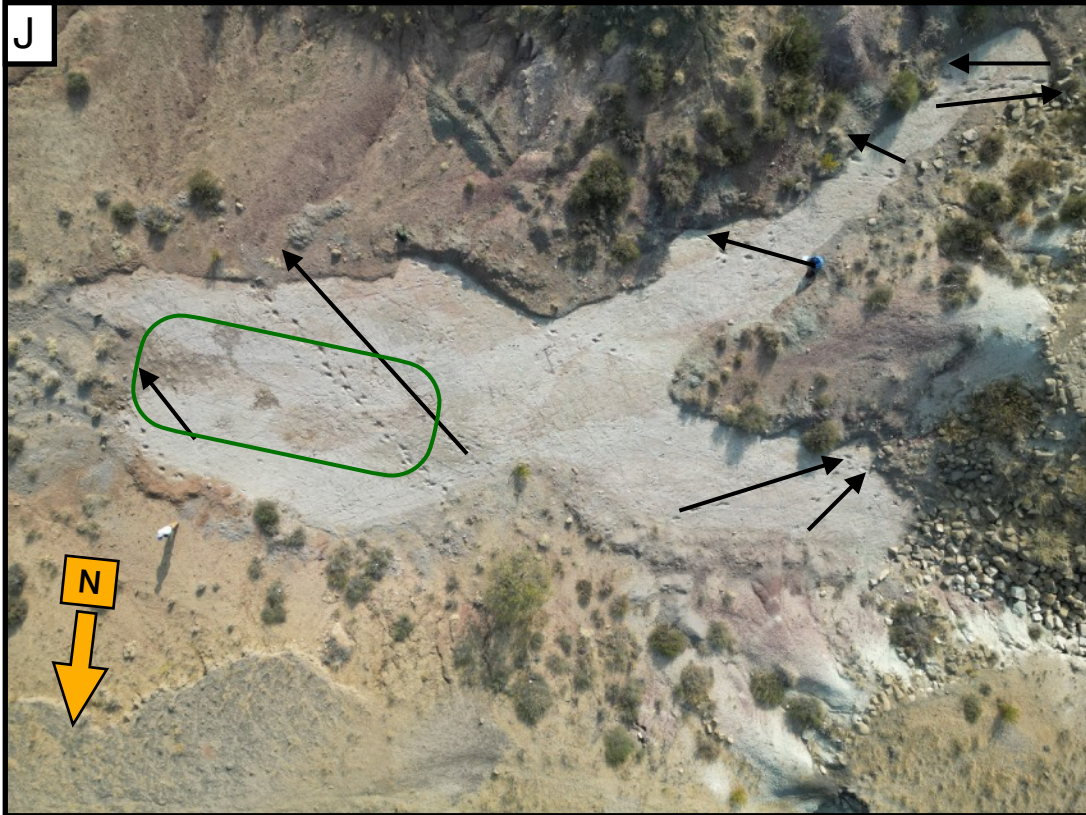

Supplement: S1 Fig — A) View of the Carreras Pampa tracksite from the north. The white lines indicate the areas where the CP bed is exposed in cross-section. B) View of site CP1. Note several conspicuous trackways with deep tracks, characteristic of the preservation styles M4 and M5. The approximate surface area is 1890 m2. The black arrows indicate the trackways with deep tracks and tail traces. The green boxes indicate three areas where the tracked surface is weathered. C) View of site CP2. Note several conspicuous trackways with deep tracks, characteristic of the preservation styles M4 and M5. The approximate surface area is 2550 m2. The black arrows indicate the trackways with deep tracks and tail traces. The green box indicates three areas where the tracked surface is weathered. D) View of site CP3. Note several conspicuous trackways with deep tracks, characteristic of the preservation styles M4 and M5. A) A curved trackway of tracks of the styles of preservation M4 and M5 (dashed line). Trackways a, b, and c are of tracks from styles of preservation M4 and M5, with associated tail traces, The green box indicates an area of weathered substrate. The black arrows indicate the trackways with deep tracks and tail traces. The approximate surface area is 590 m2. E) View of site CP4. Note several conspicuous trackways with deep tracks, styles of preservation M4 and M5. The approximate surface area is 285 m2. F) View of site CP5. Note several conspicuous trackways with deep tracks, characteristic of the preservation styles M4 and M5. The black arrows indicate the trackways with deep tracks and tail traces. The green box indicates an area where the tracked surface is weathered. The approximate surface area is 415 m2. G) View of site CP6. Sites CP5, CP2 and CP1 are in the background. The green boxes indicate an area where the tracked surface is weathered. The approximate surface area is 334 m2. H) View of site CP7. The black arrows indicate the trackways with deep tracks and tail traces. The [file pone.0335973.s002.pdf]

## Supporting Information S2 Fig

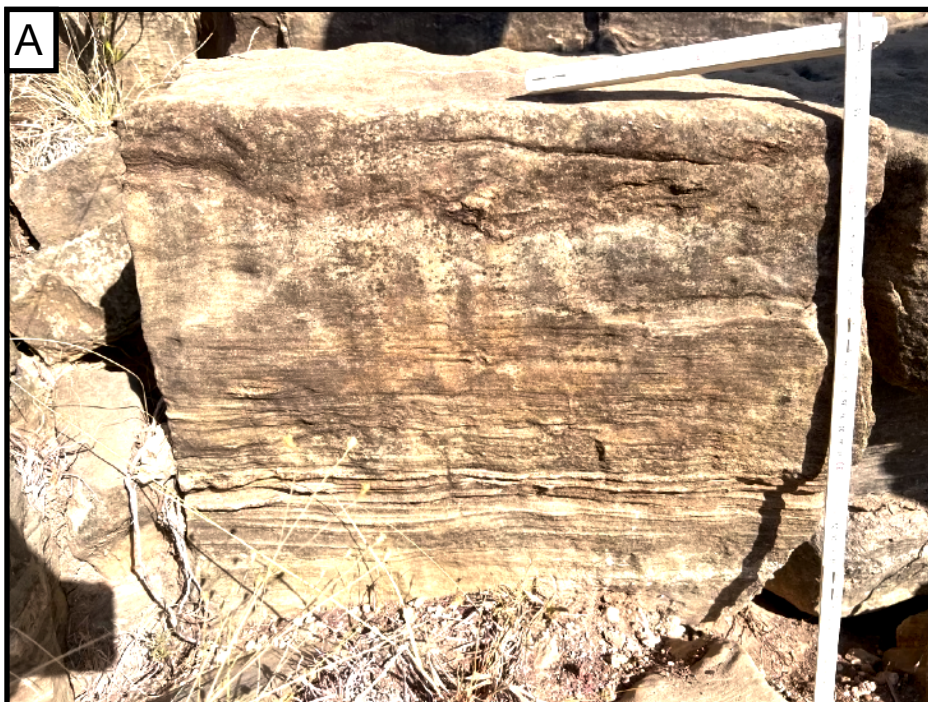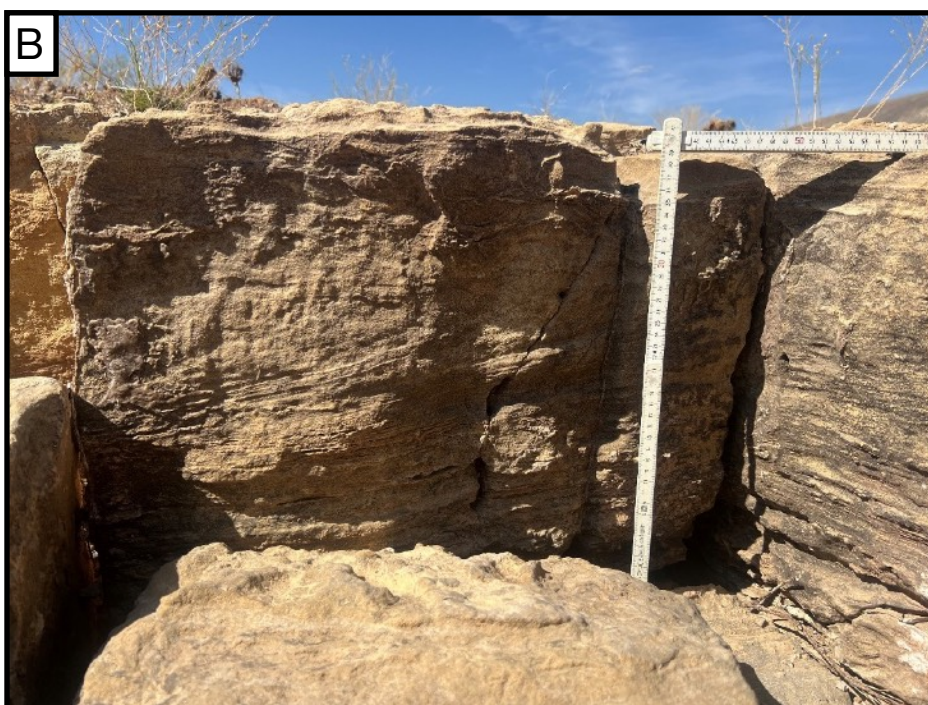

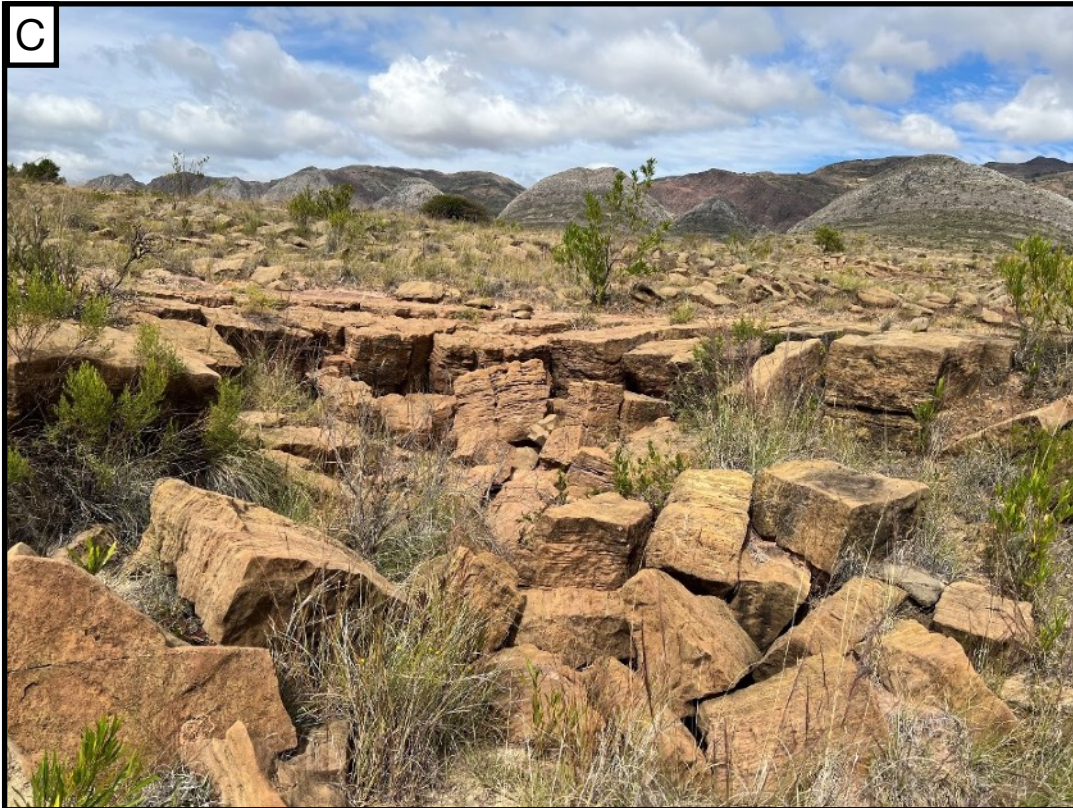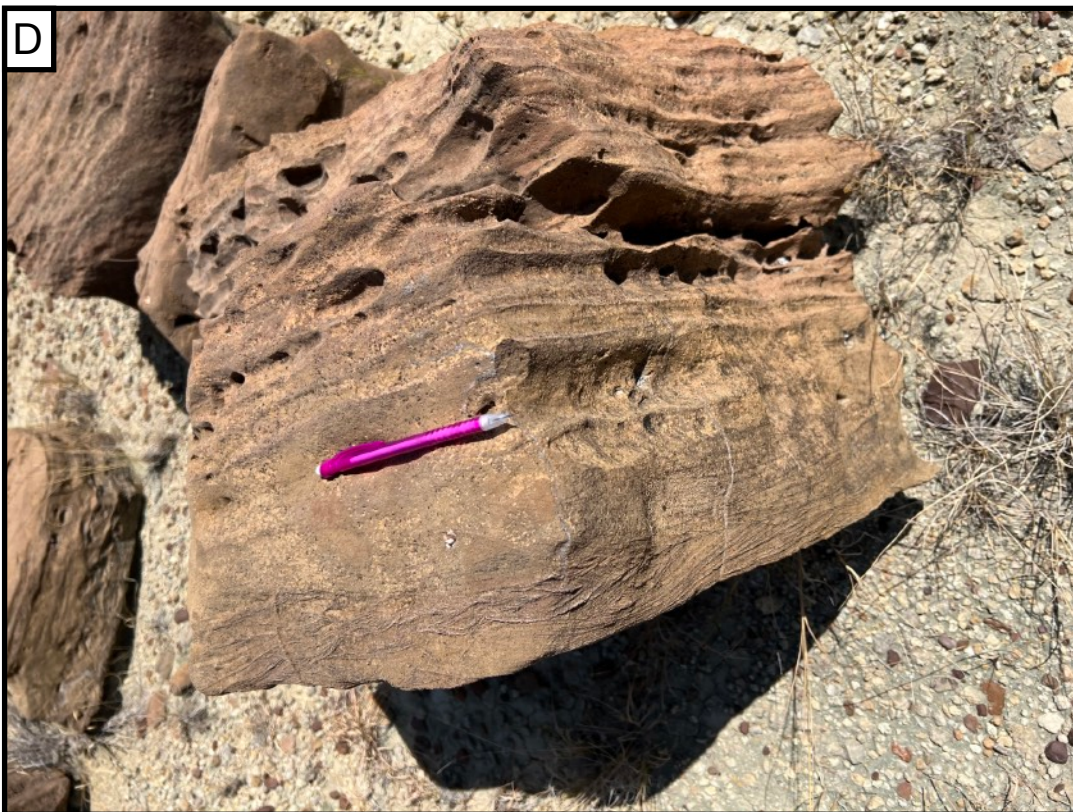

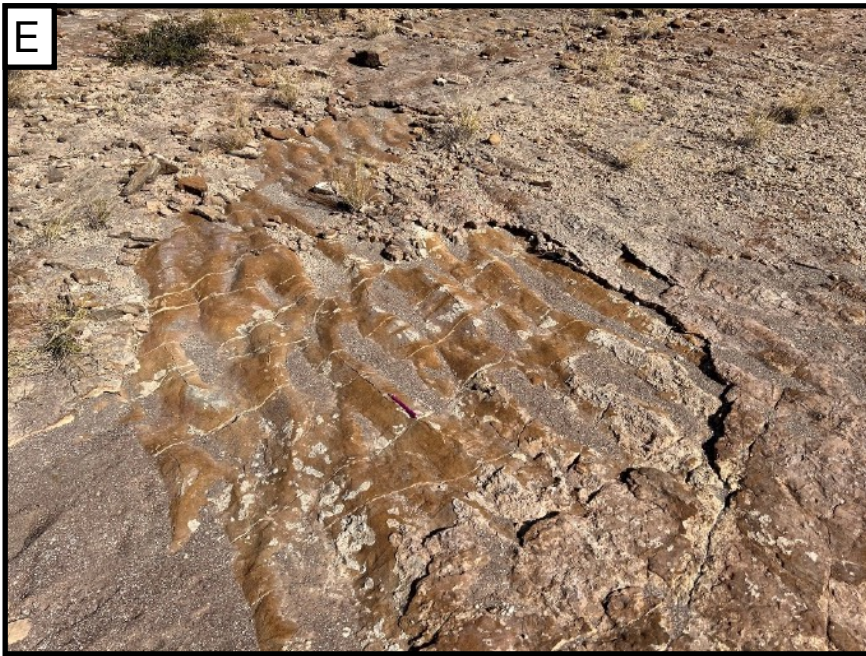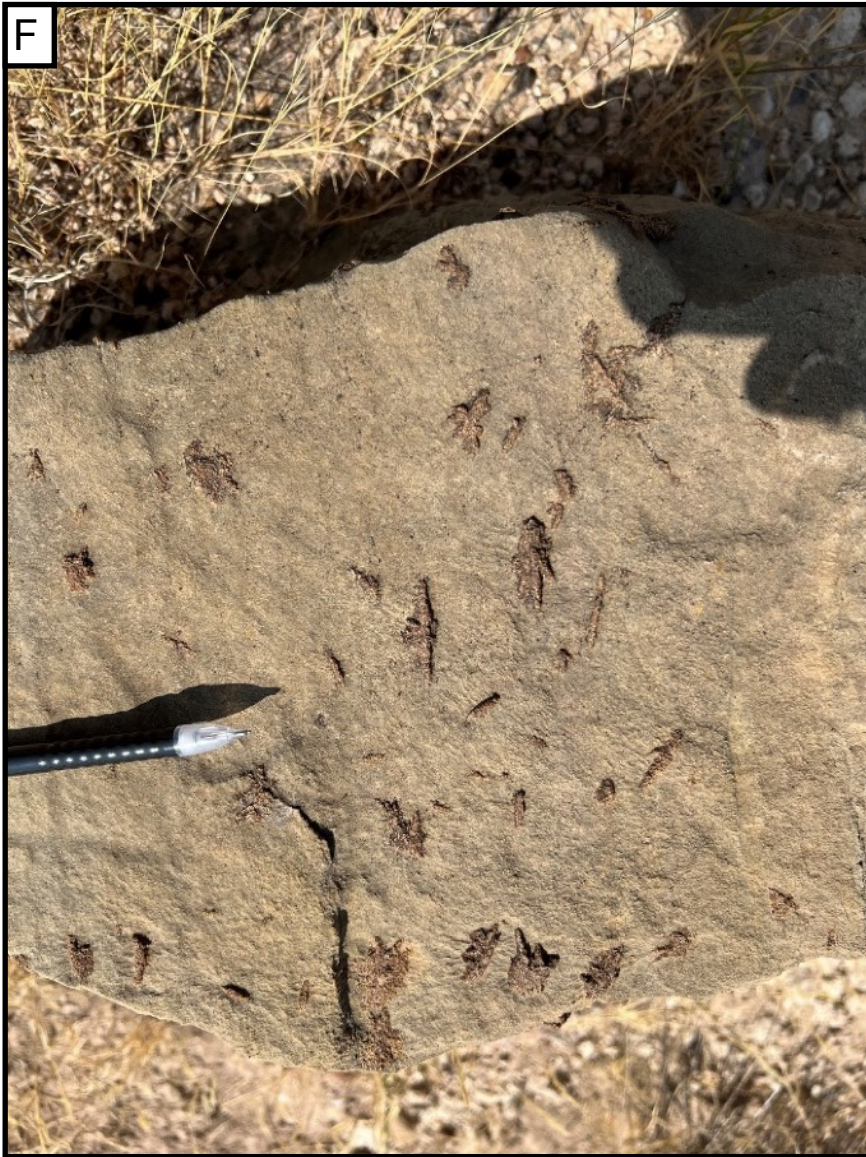

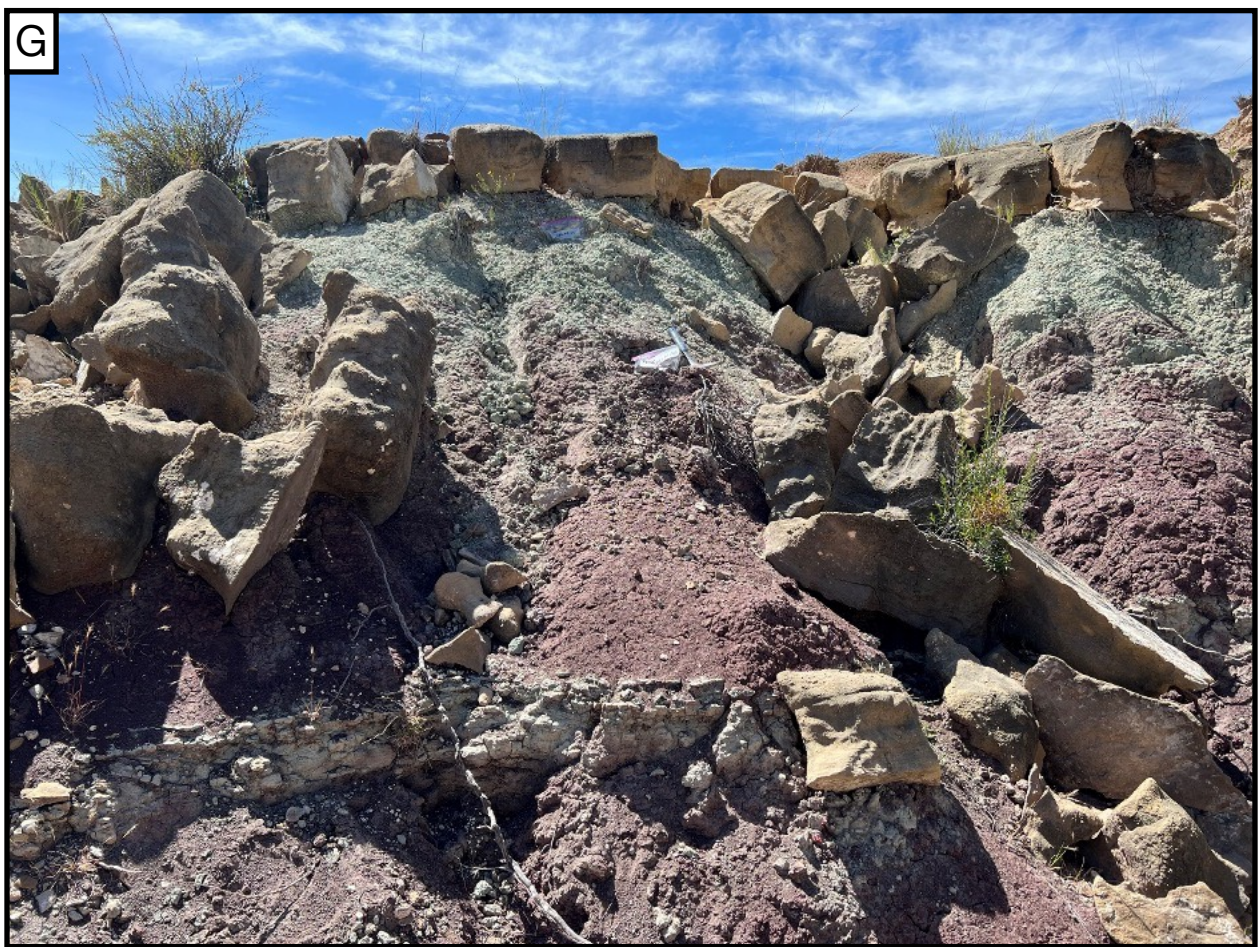

Supplement: S2 Fig — A) Cross-section of CP bed showing wavy and sub-horizontal lamination, trough cross-lamination, and wave ripple cross-lamination. B) Cross-section of CP bed showing trough cross-lamination at a scale of tens of centimeters and wave ripple cross-lamination at the top. C) Typical appearance of the red bed (RB) outcrop showing rectangular and resistant blocks. D) Cross-section of the red bed (RB) showing characteristic ripple trough and low amplitude trough cross-lamination. Laminae are enhanced by the weathering residues of quartz grains that follow cross-lamina surfaces. E) Wave ripple bedforms on the upper surface of the wave ripple (WR) bed. Many of the ripples have sharp crests with horizontal burrow traces that follow the ripple crest. F) Pseudomorphs of evaporite crystals exposed by weathering and characteristic of bed evaporite bed (EB). G) Typical appearance of silty claystone units. The resistant bed at the top of the outcrop is the EB unit. (PDF) [file pone.0335973.s003.pdf]

## Supporting Information S3 Fig

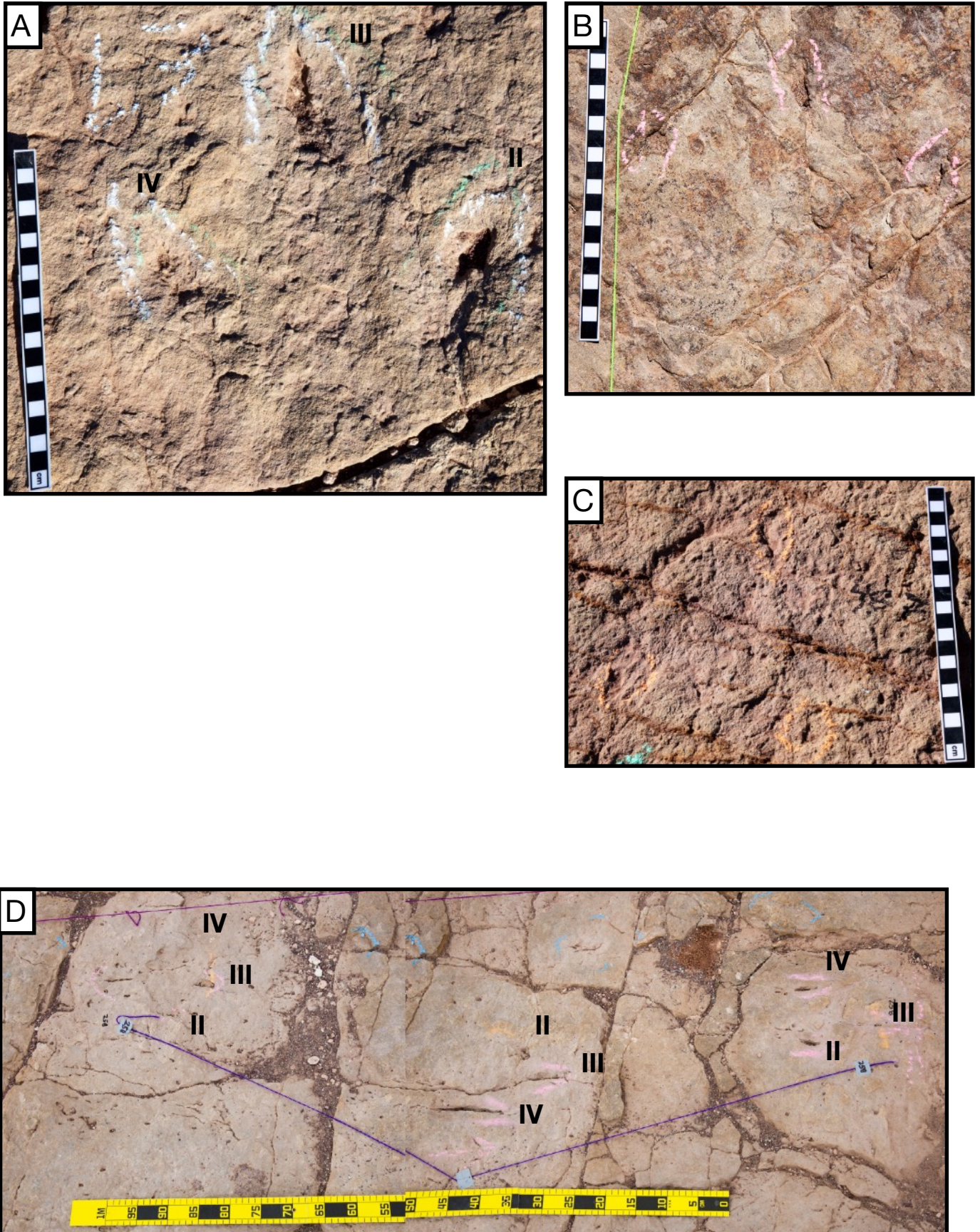

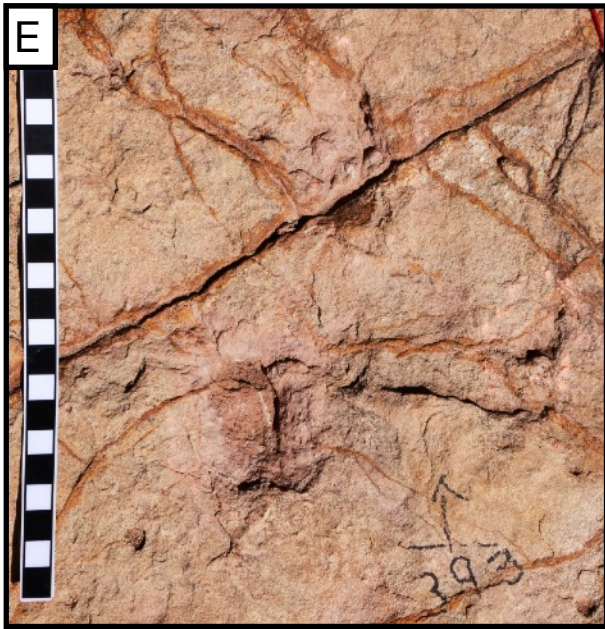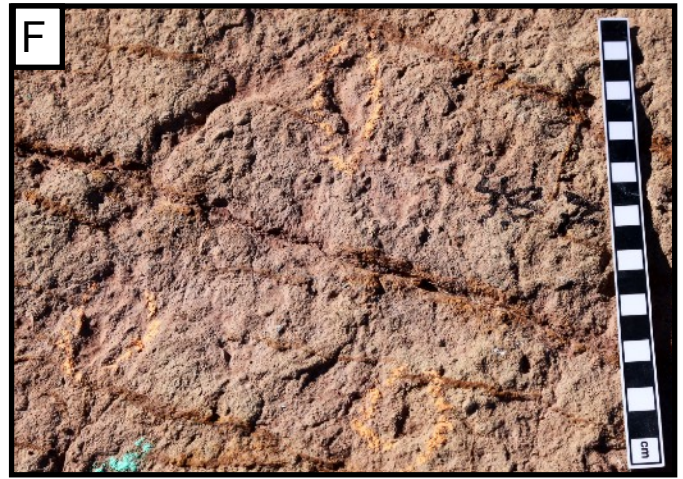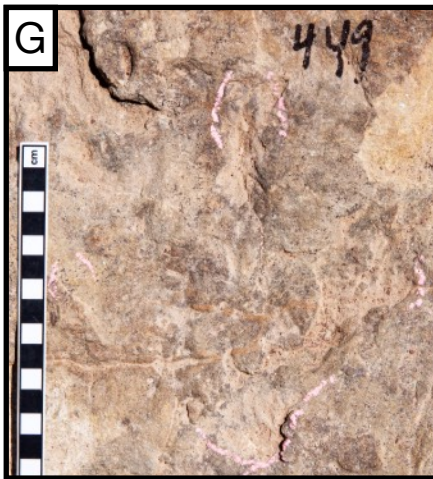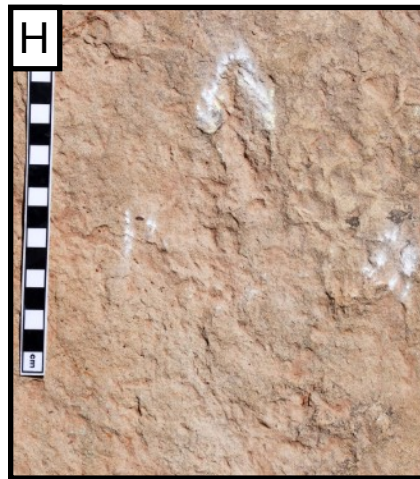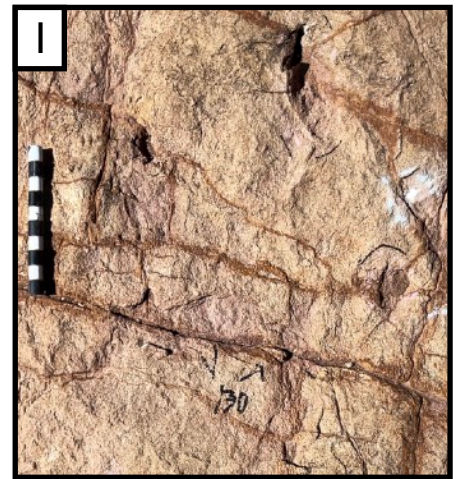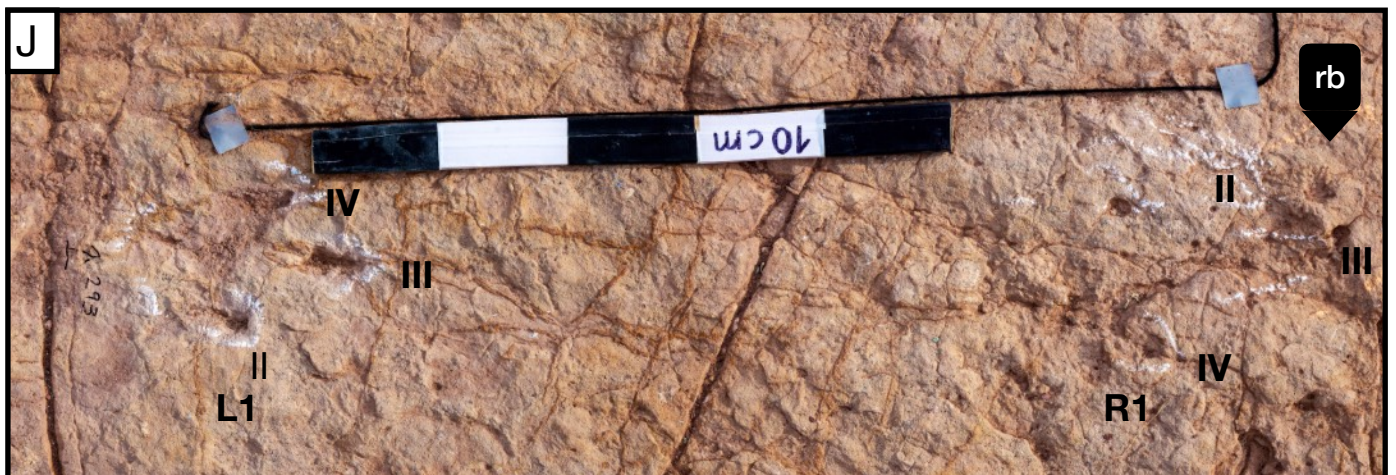

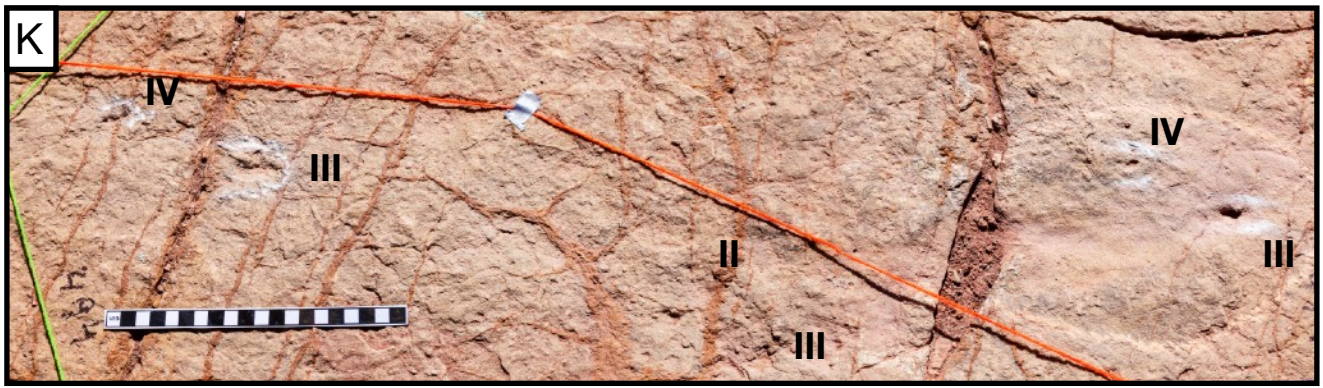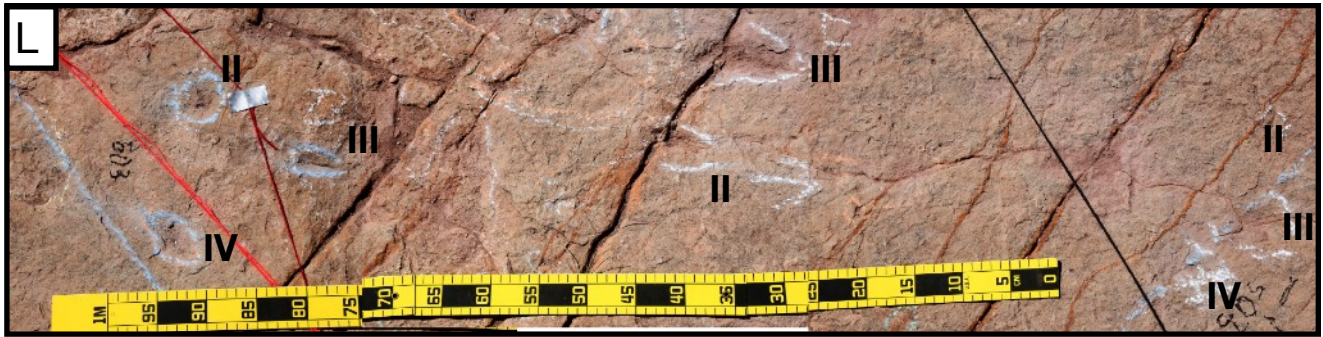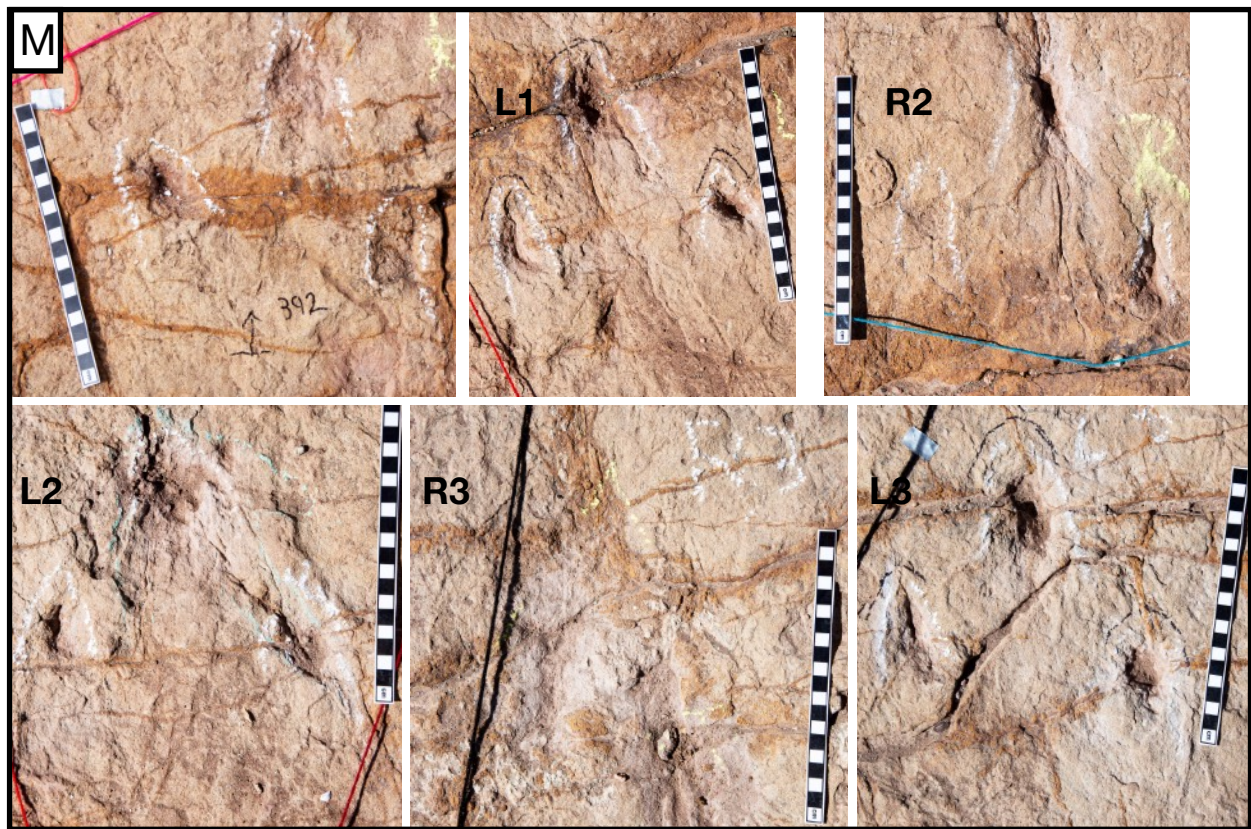

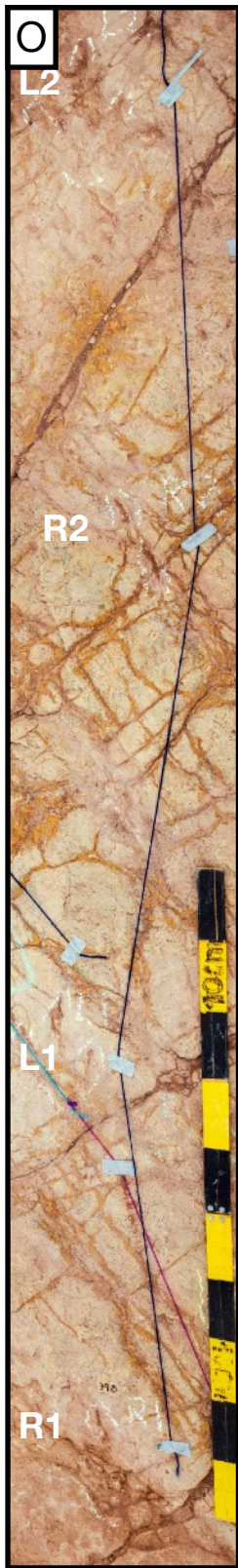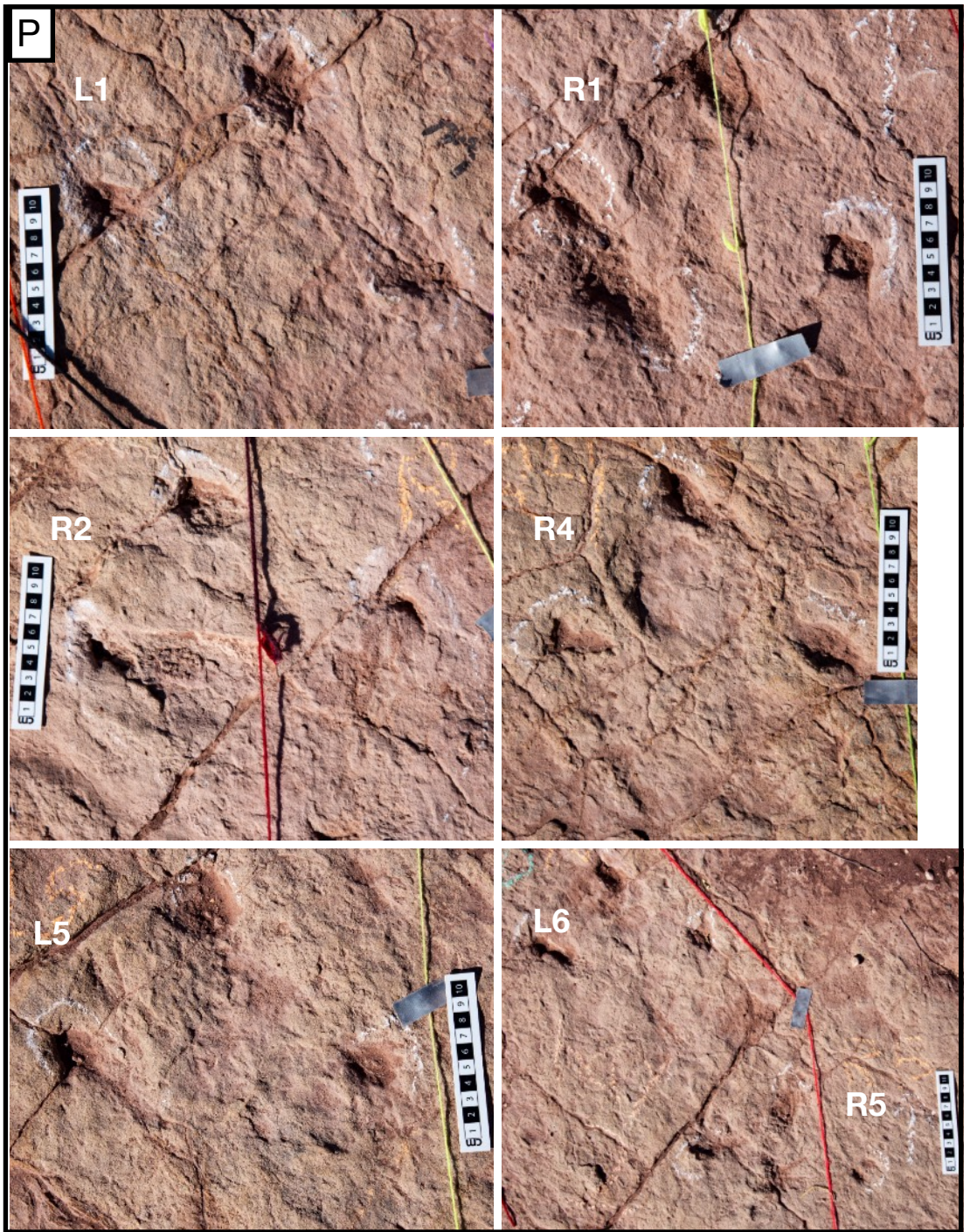

Supplement: S3 Fig — A) Trackway T22-128. Small track with the claws well marked and the metatarsophalangeal area absent. B) Trackway T22-443. One track of the style of preservation M1. C) Trackway T22-483. D) Trackway T22-258. This trackway consists of three tracks (L1, R1, L2) preserved as indentations of the claws at the northern side of site CP1. R1 shows two linear, deep, and very narrow traces of digits III and IV, with digit II consisting of only a tiny scar. L2 exhibits comma-shaped, deep, and narrow markings for digits II and IV, and a longer marking for digit III. No other morphological details are preserved. E) Trackway T22-2–393. F) Trackway T22-483. G) Trackway T22-449. H) Trackway T22-496. I) Trackway T22-2–130. J) Trackway T22-2–293. Trackway with two tracks of the style of preservation M1. There is a well-preserved rosette-like burrow (rb) next to the tip of digit III of the R1 track. K) Trackway T22-2–494. Three tracks of the style of preservation M1. L) Trackway T22-2–503. Three tracks of the style of preservation M1. M) Trackway T22-2–392. This trackway consists of tracks of the style of preservation M1, except the last three tracks, which are in the style M3 (not shown here). O) Trackway T22-2–392. This trackway consists of tracks of the style of preservation M1, except the last three tracks, which are in the style M3 (not shown here). P) Trackway T22-2–36. Trackway with eleven large tracks of the style of preservation M1, consisting of three deep, conical indentations. R2 shows the shallow contour of the track and a shallow indentation at the posterior end of the impression of the heel. The scale in A-C, E, F, G, H, K and M is 20 cm; in J is 50 cm; in D, L and O is 1 m; in I and P is 10 cm. (PDF) [file pone.0335973.s004.pdf]

## Supporting Information S4 Fig

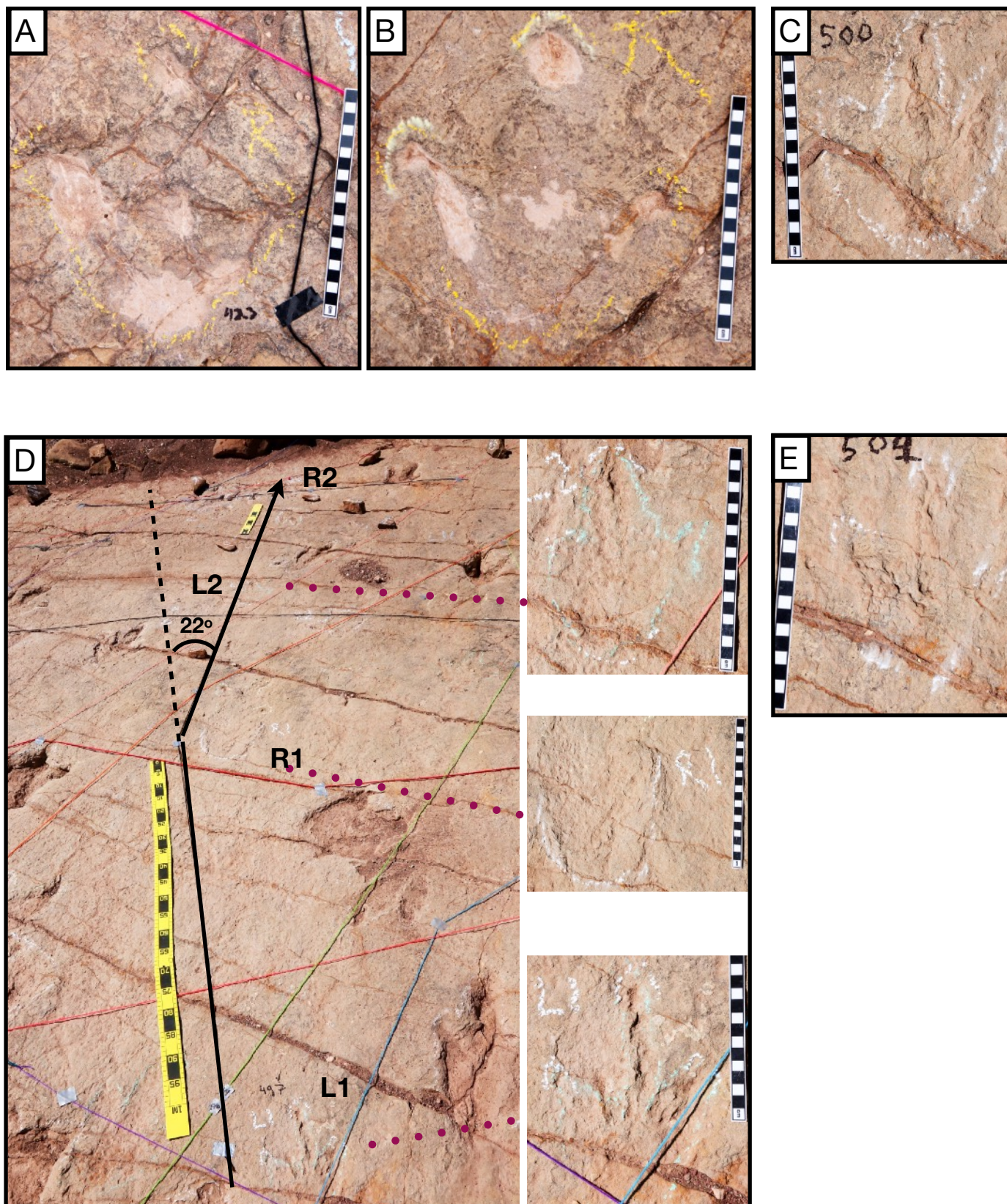

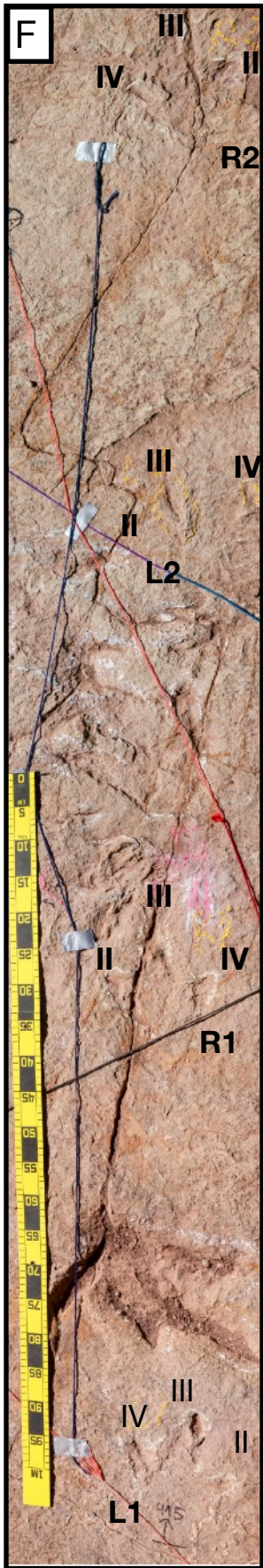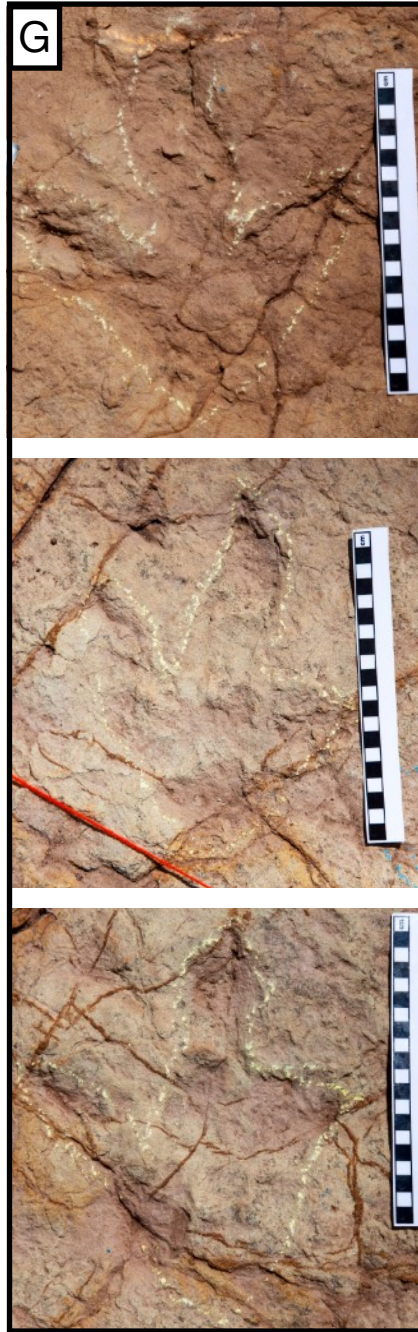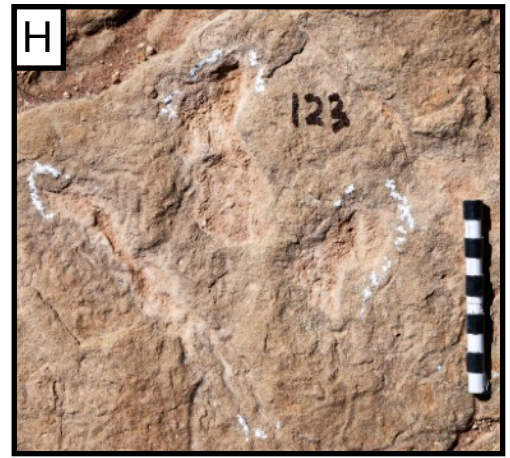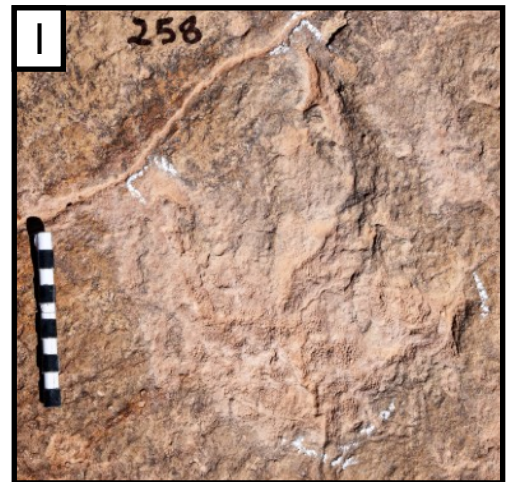

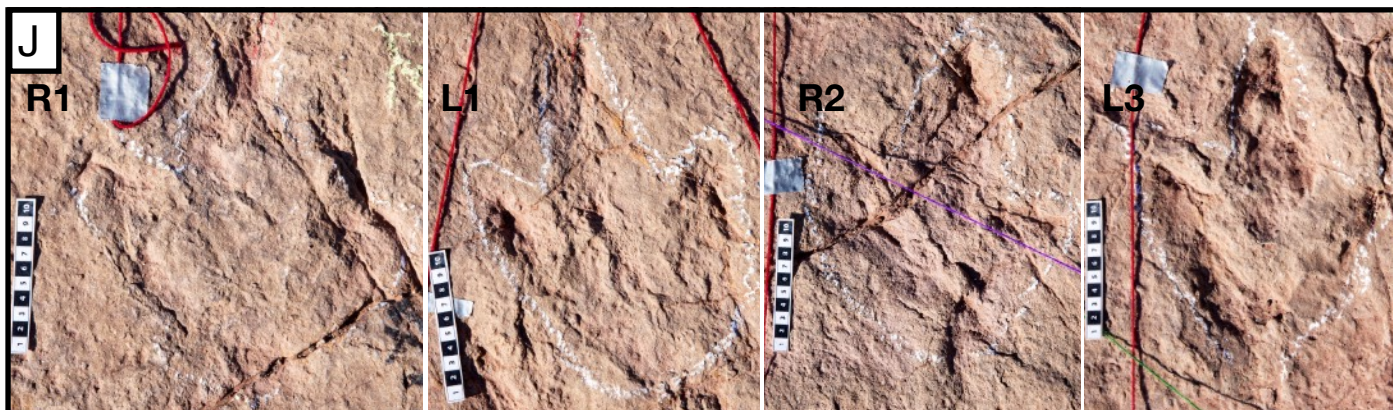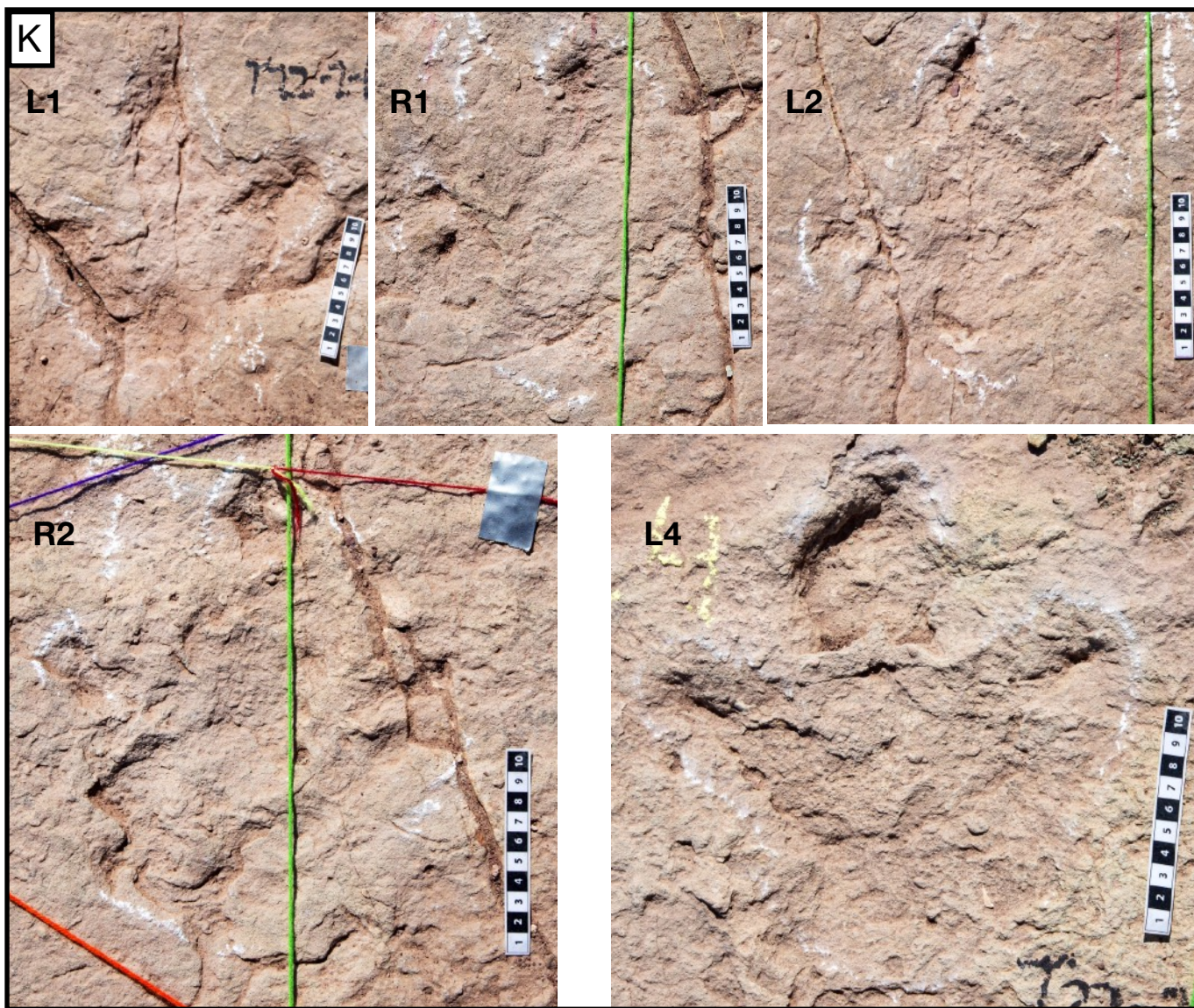

Supplement: S4 Fig — A-B) Trackway T22-423. C) Trackway T22-2–500. Digit IV is not impressed in this track. D) Trackway T22-2–497. This trackway is 3.22 m long with four tracks of the style of preservation M2. There is a turn of 22 degrees in the direction of movement at R1. The scale is in cm, 1 m in the large photograph and 20 cm in the small photographs. E) Trackway T22-2–500. One track of the style of preservation M2. Digit IV shows raised sediment. F) Trackway T22-2–415. Trackway 1.9 m long with six tracks of the style of preservation M2. G-I) Tracks and trackways of the style of preservation M3. G) Trackway T22-236. Three tracks of the style of preservation M3. H) Trackway T22-2–123. I) Trackway T22-2–258. J) Tracks of the styles of preservation M2, M3 and M4. Trackway T22-2–3. Very long trackway with nineteen tracks (R5 missing) of the preservation styles M2, M3 and M4. K) Trackway T22-2–6. Trackway with four tracks of the styles of preservation M2 and M3. The scale in D is 1 and 20 cm in the three small photos; in F is 1 m; in A-C, E and G is 20 cm and in H-K is 10 cm. (PDF) [file pone.0335973.s005.pdf]

## Supporting Information S5 Fig

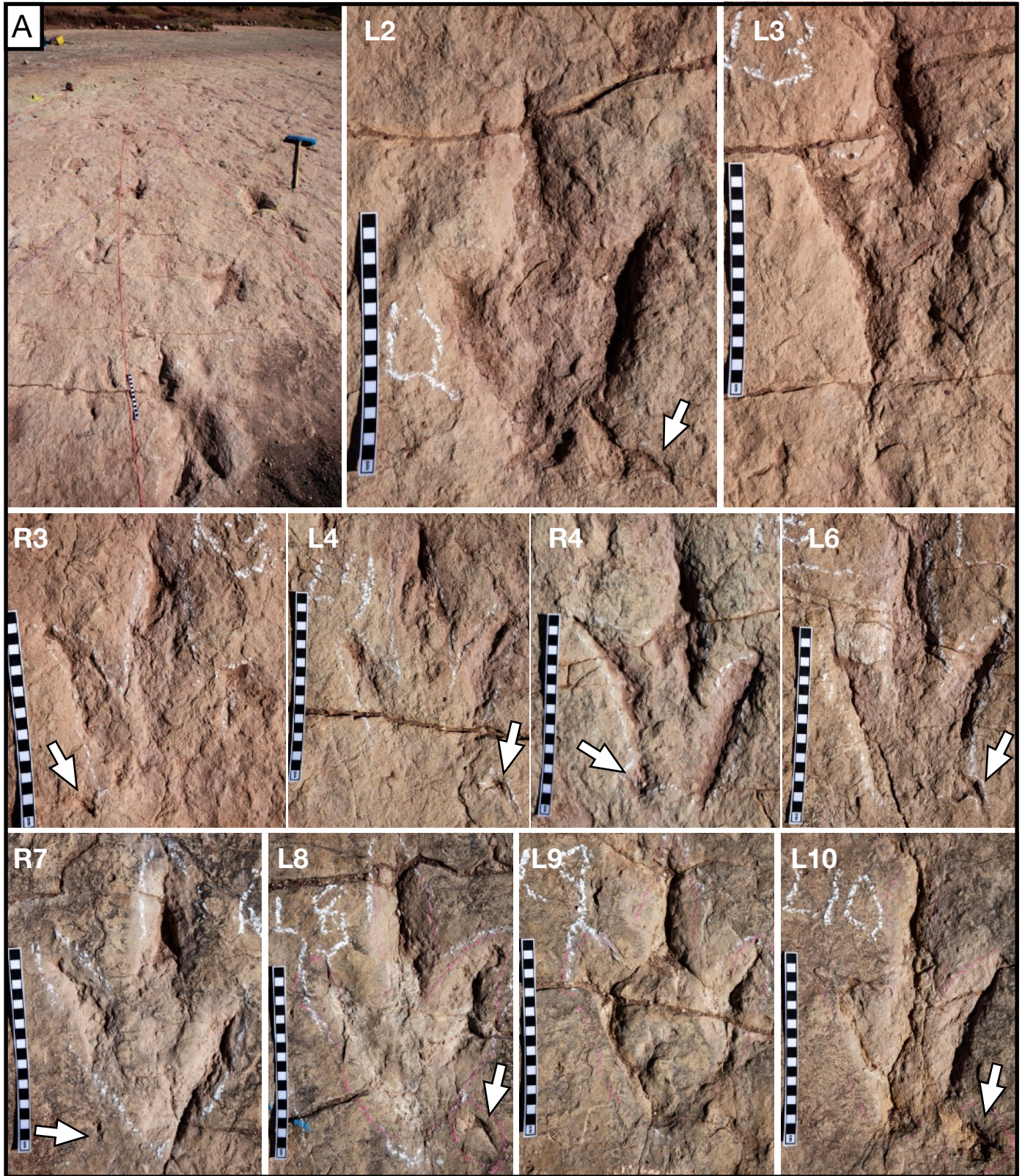

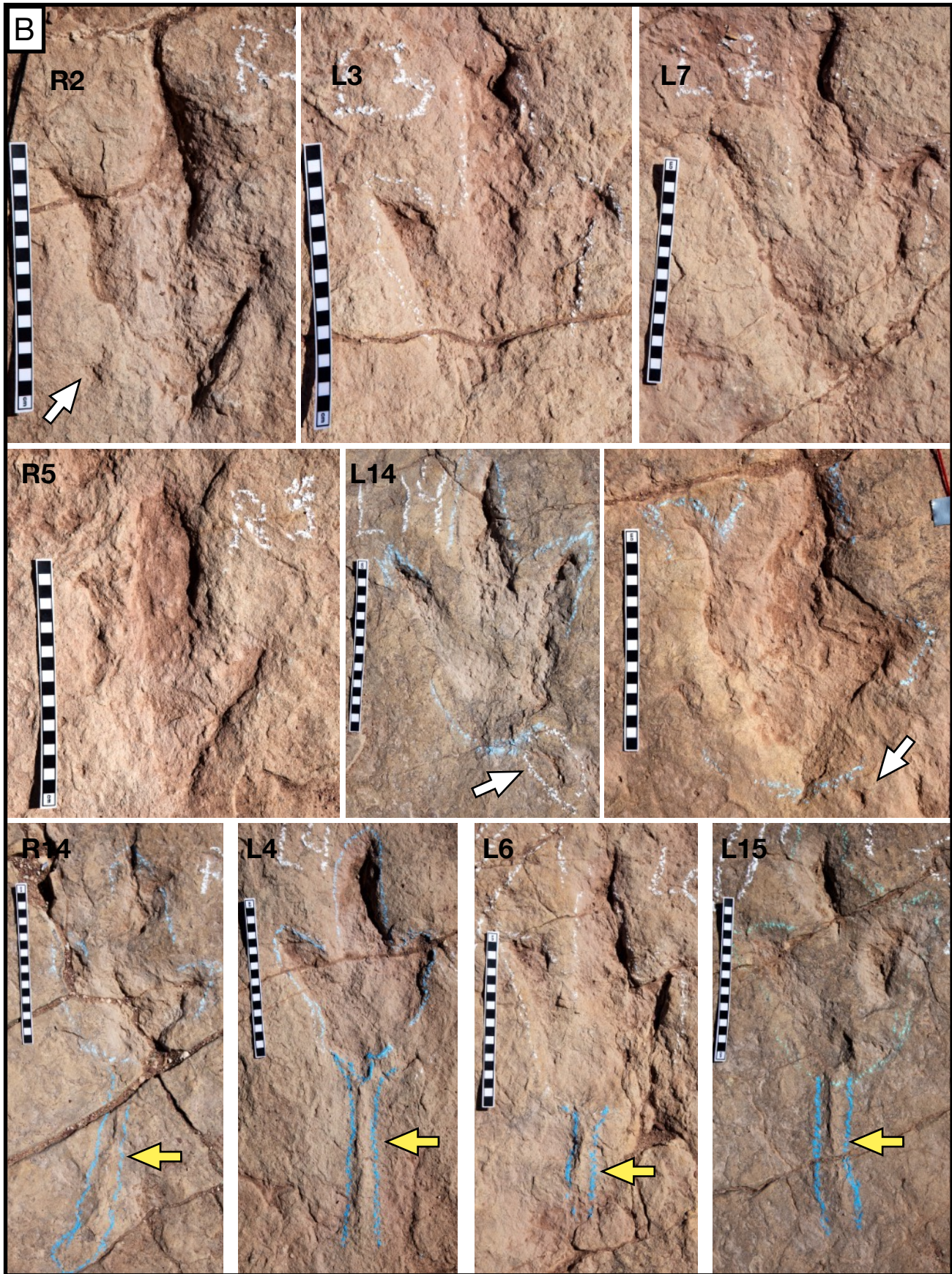

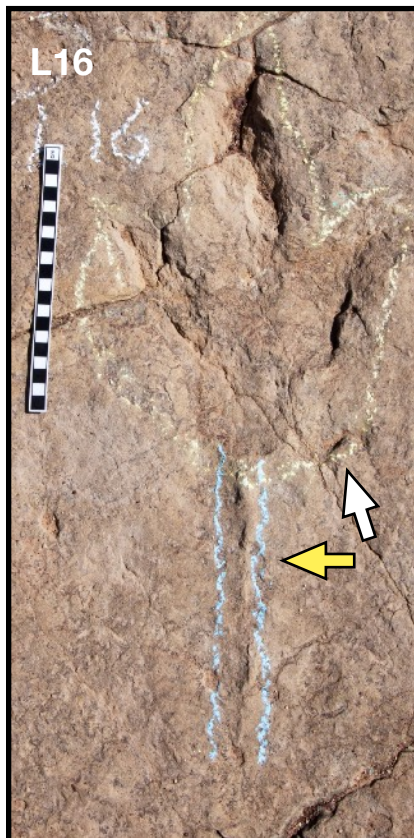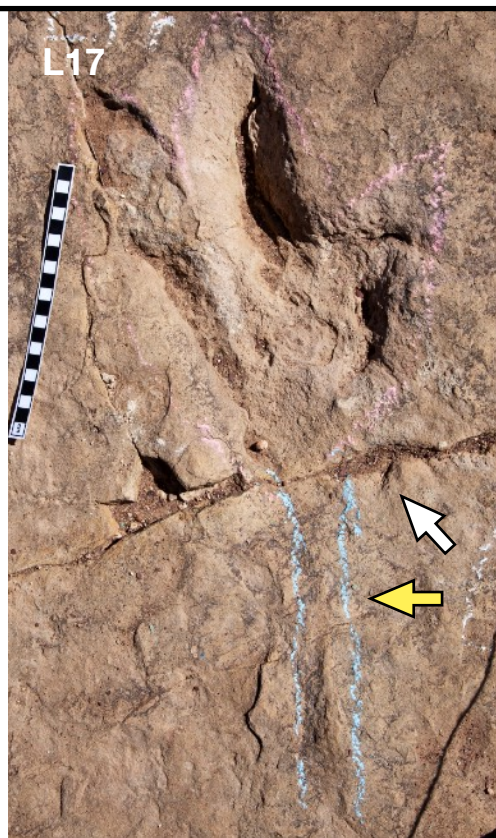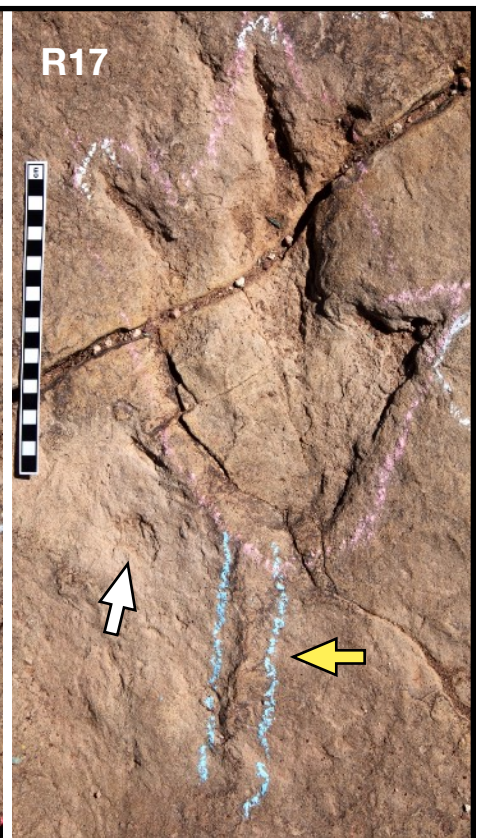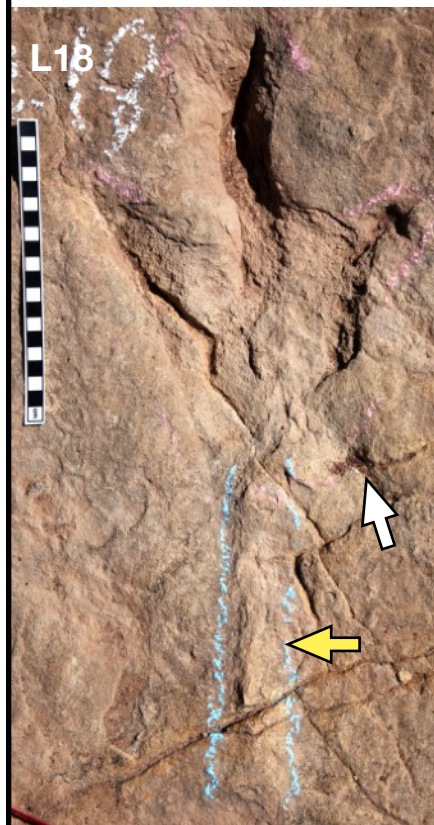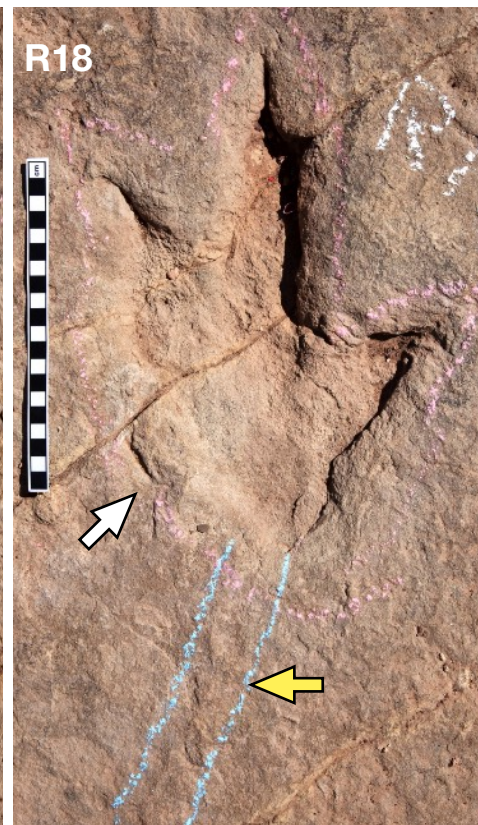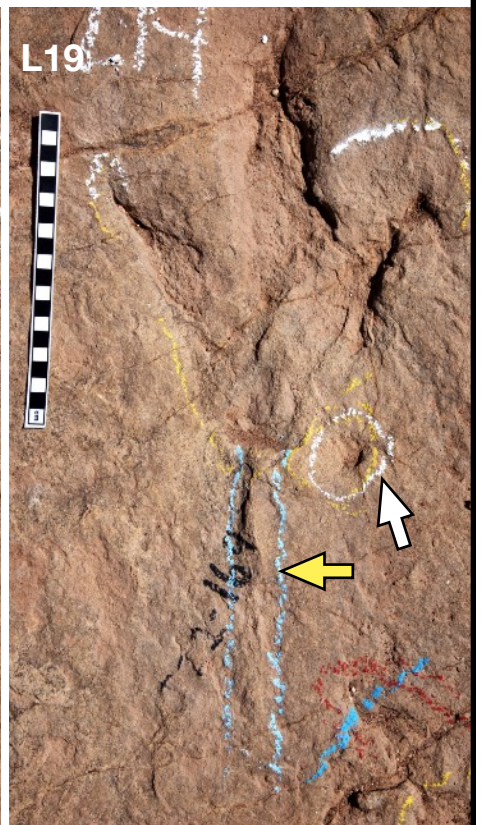

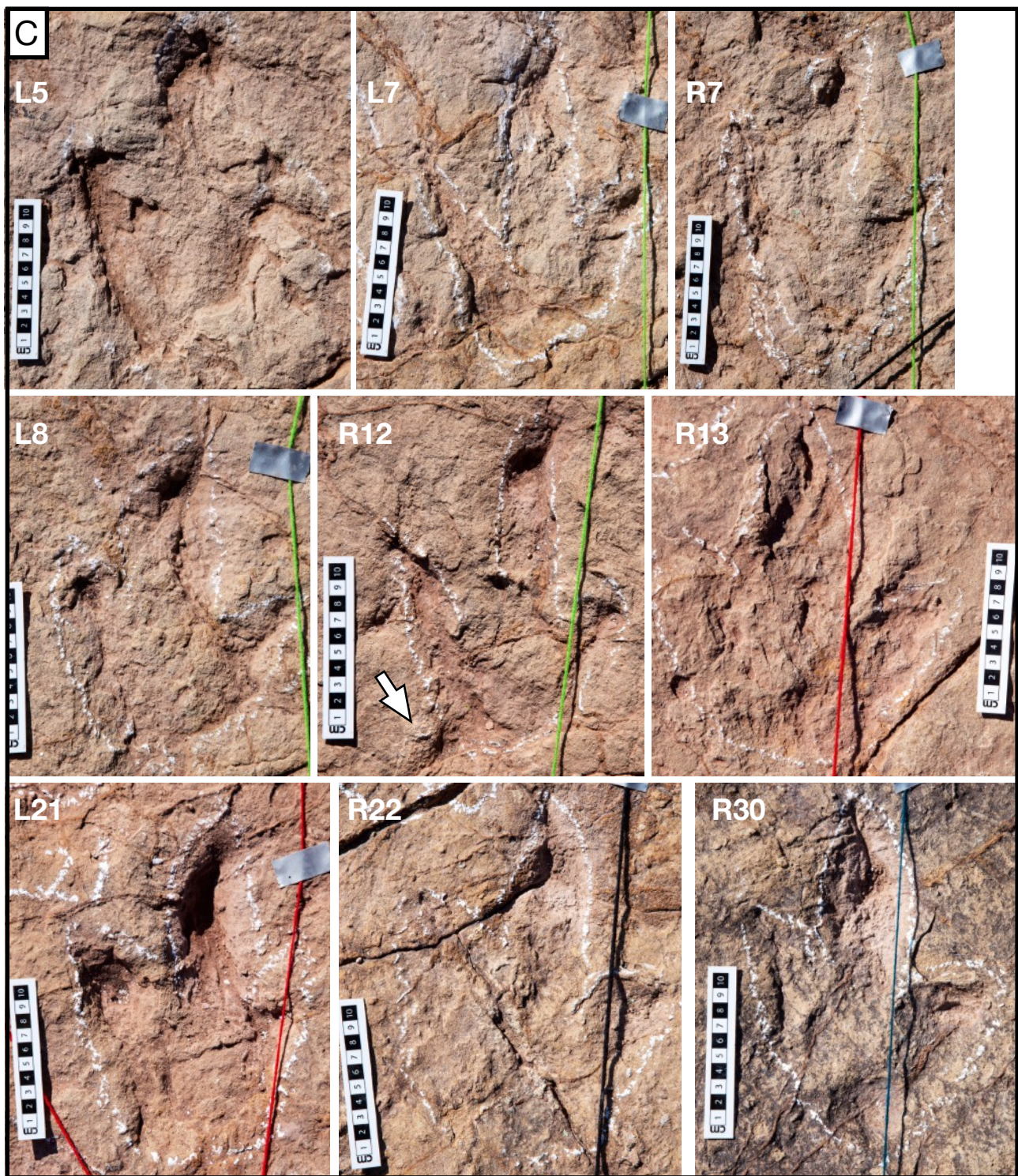

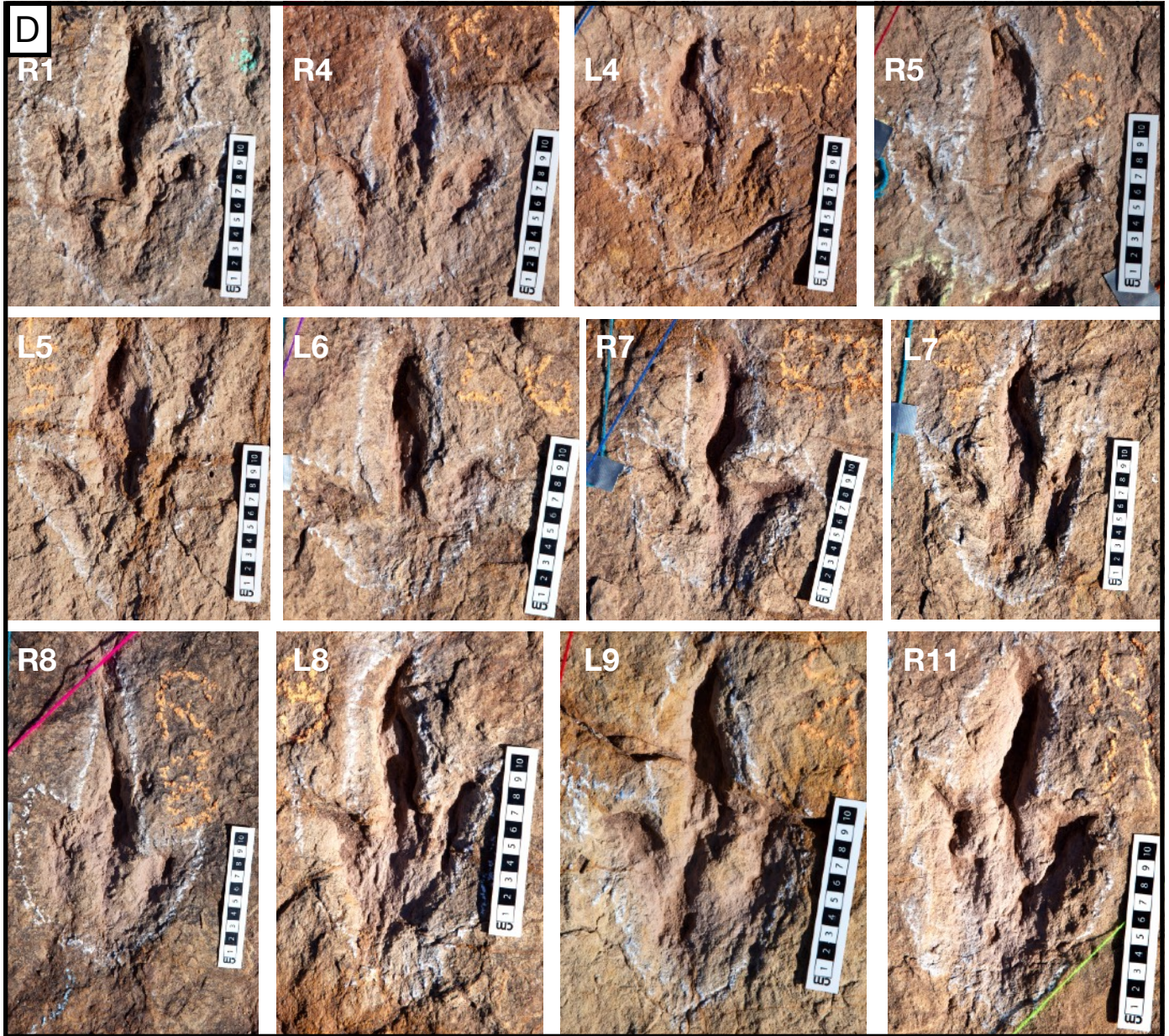

Supplement: S5 Fig — A) Trackway T22-79. Trackway with many deep tracks of the styles of preservation M4 and M5. Some have the mark of the hallux (white arrow). B) Trackway T22-72. Most tracks of this trackway are of the style of preservation M4, well-marked and with hallux impressions (white arrow). Several of them have a raised posterior ridge (indicated by the yellow arrow). C) Trackway T22-2–21. Tracks of the style of preservation M4. The trackway has ninety-nine small tracks, and one is missing (R20). The impression of the hallux is indicated with a white arrow. Notice padding preserved in track R30. D) Trackway T22-2–47. Very long trackway with small tracks of the styles of preservation M3 and M4. The heel impression is poorly marked, acuminate or narrowly round. Some claw marks are oriented backward (R1, R5, L6, R7, L9). The scale in A and B is 20 cm; in C and D is 10 cm. (PDF) [file pone.0335973.s006.pdf]

## Supporting Information S6 Fig

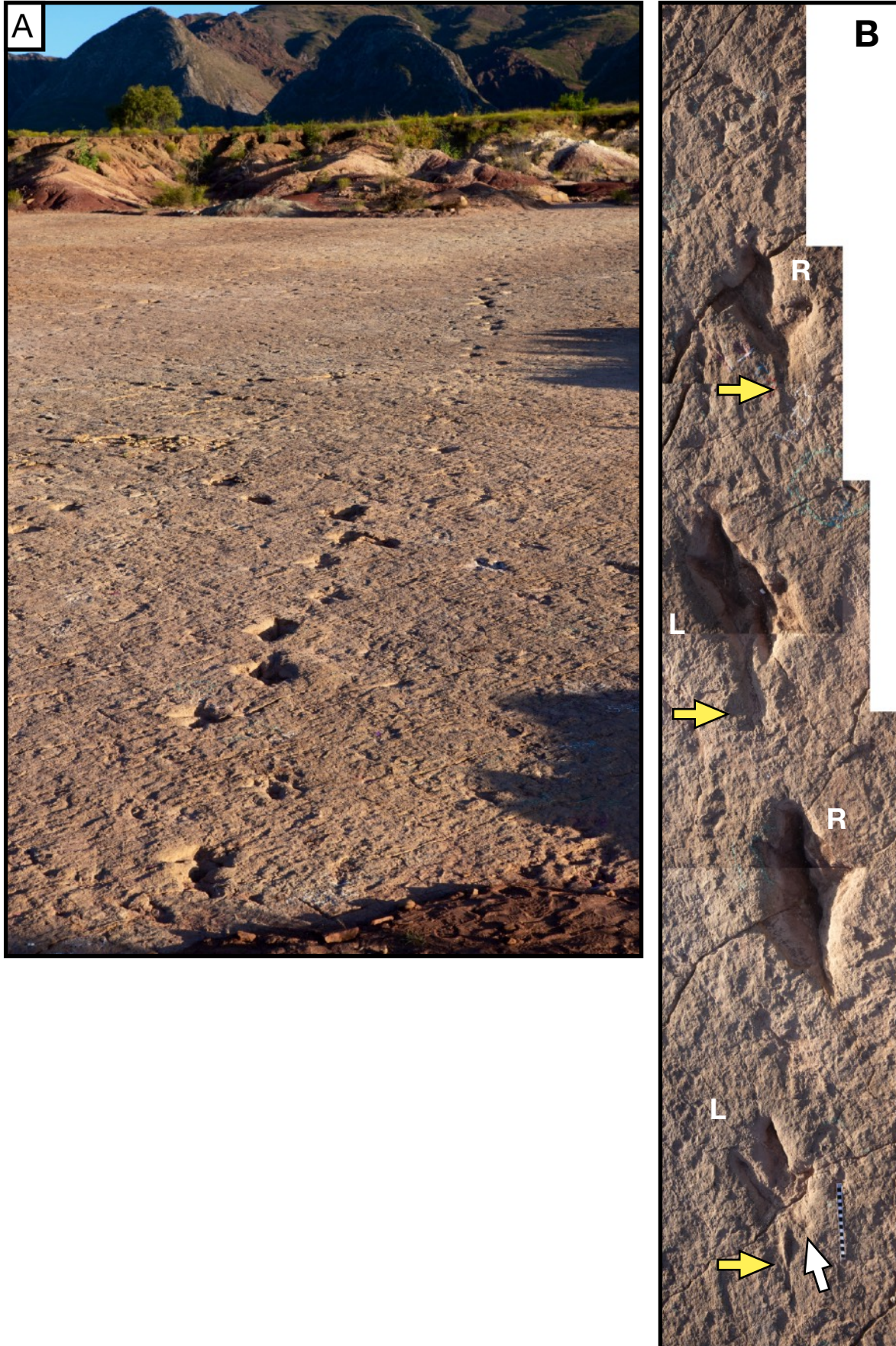

C

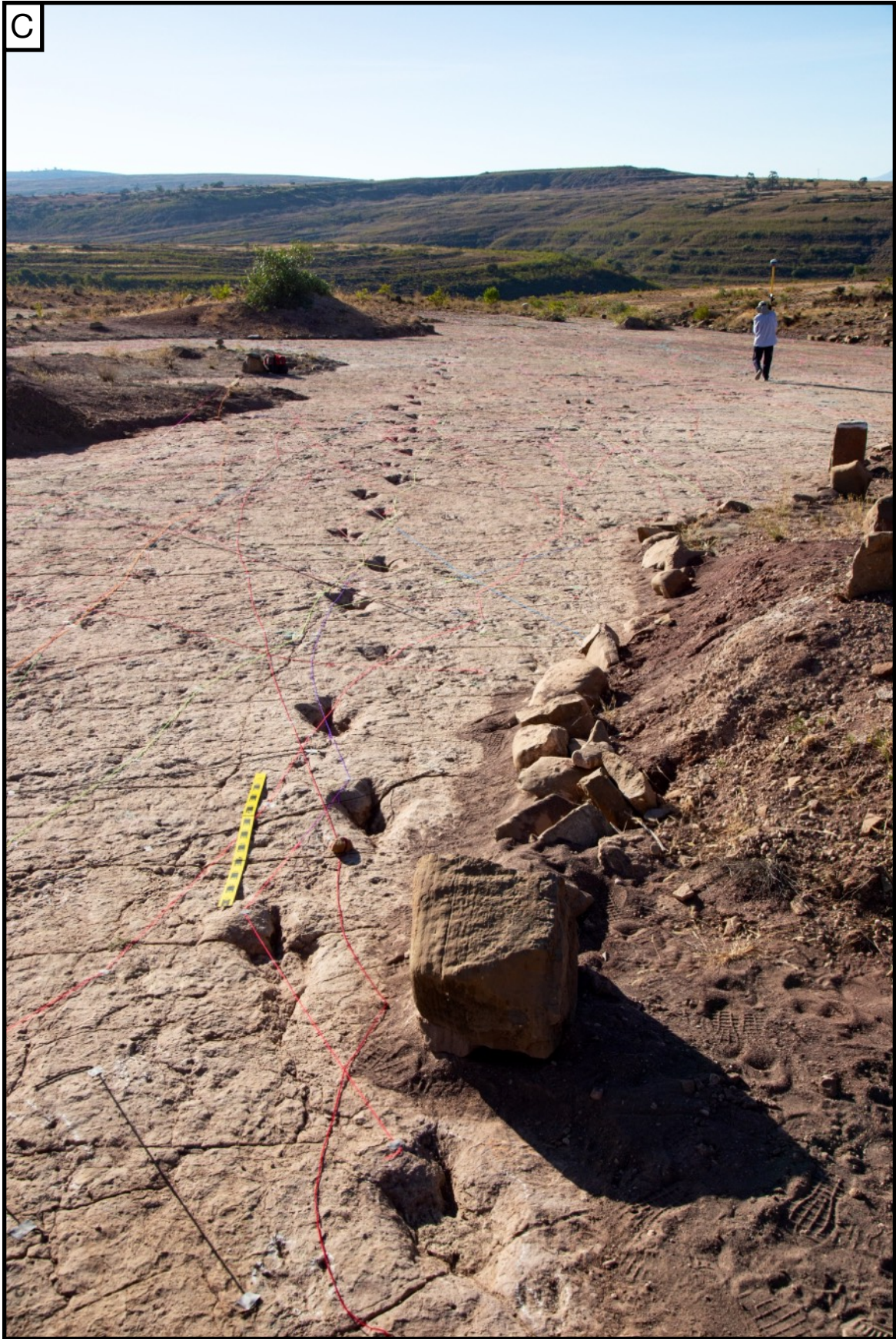

Supplement: S6 Fig — A-B) Trackway T22-102. Trackway with tracks of styles of preservation M4 and M5. Most of this trackway’s tracks are well-marked and have hallux impressions (white arrow). Several tracks have a raised posterior ridge (yellow arrow). The left (L) track in the middle part of photo B shows a posterior groove instead of a raised ridge. C) Trackway T22-2–25. Very long trackway with one hundred three very deep tracks of the style of preservation M5. Several tracks have associated tail traces. The scale in B is 20 cm and in C is 1 m. (PDF) [file pone.0335973.s007.pdf]

## Supporting Information S8 Fig

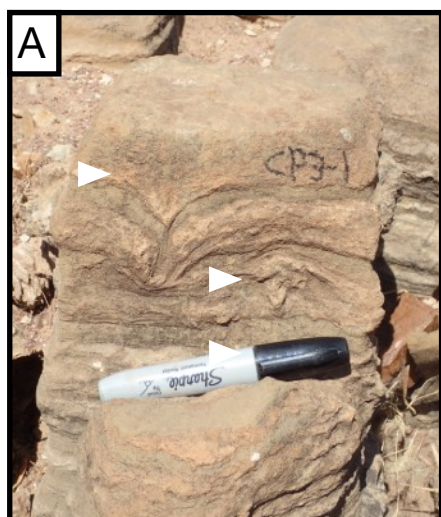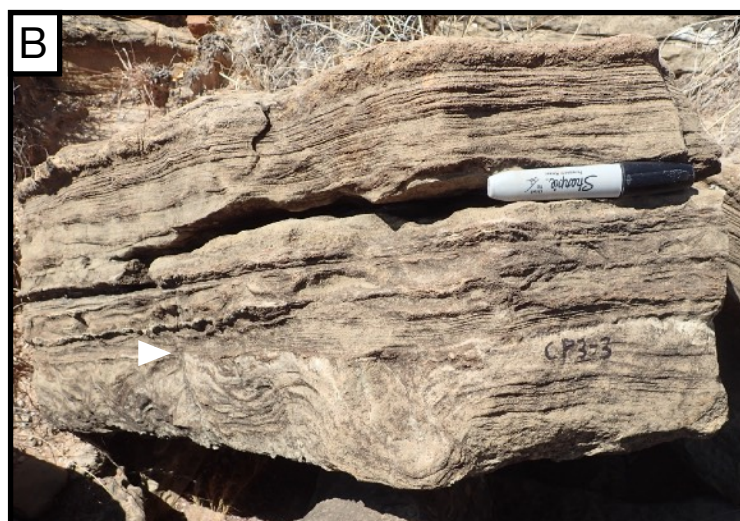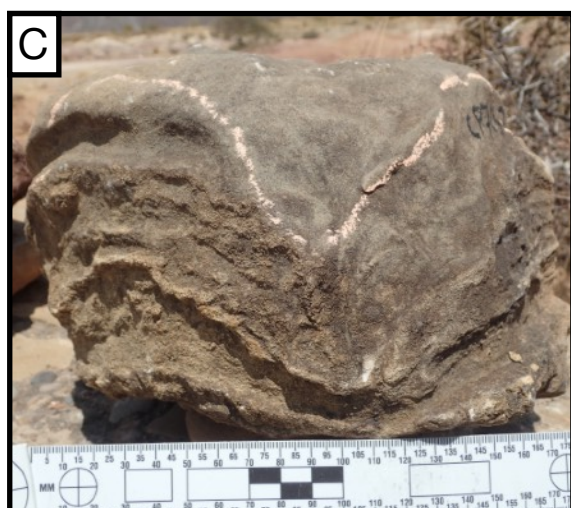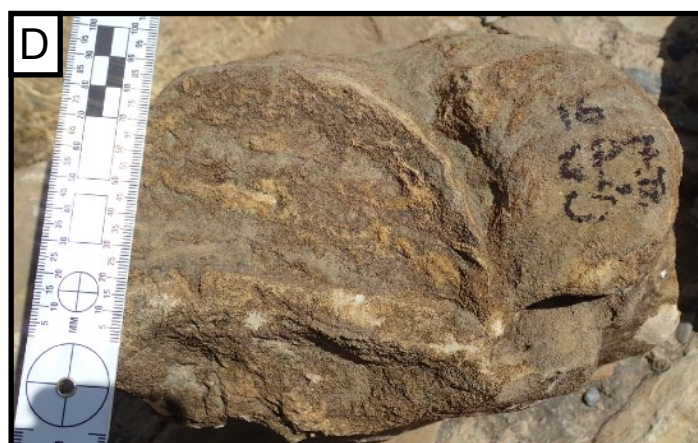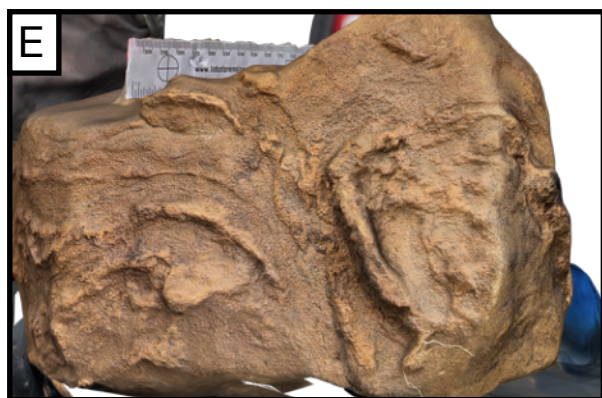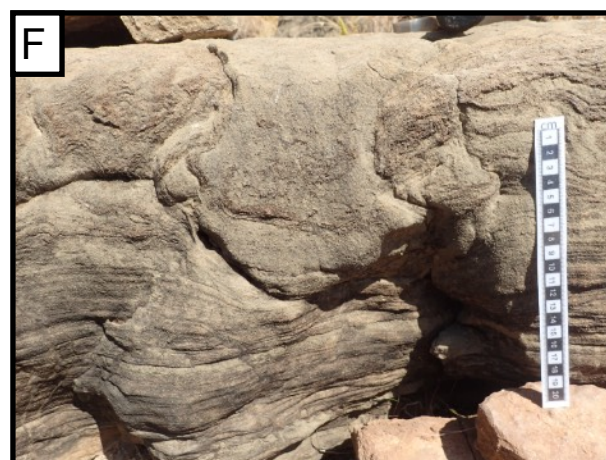

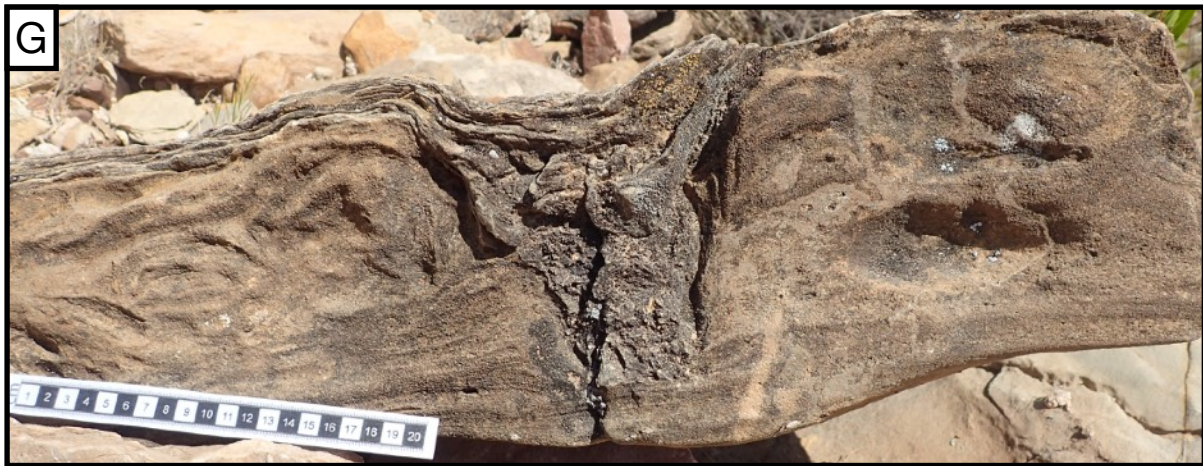

Supplement: S8 Fig — A) Track CP3−1. Deformation in two different layers corresponding to two different tracks (white arrowheads). B) Track CP3−3. C) Track CP7CS2. D) Track CP7CS16. E) Unnamed track in cross-section. F) Track TPCS24−2. G) Track TPCS24−3. The scales are in cm. (PDF) [file pone.0335973.s009.pdf]

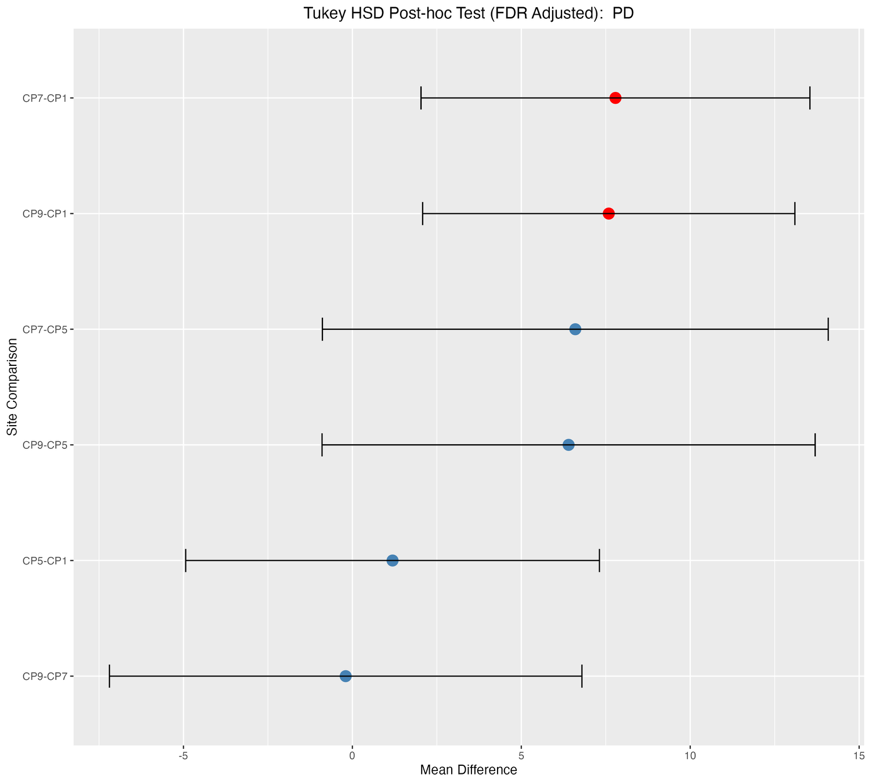


A


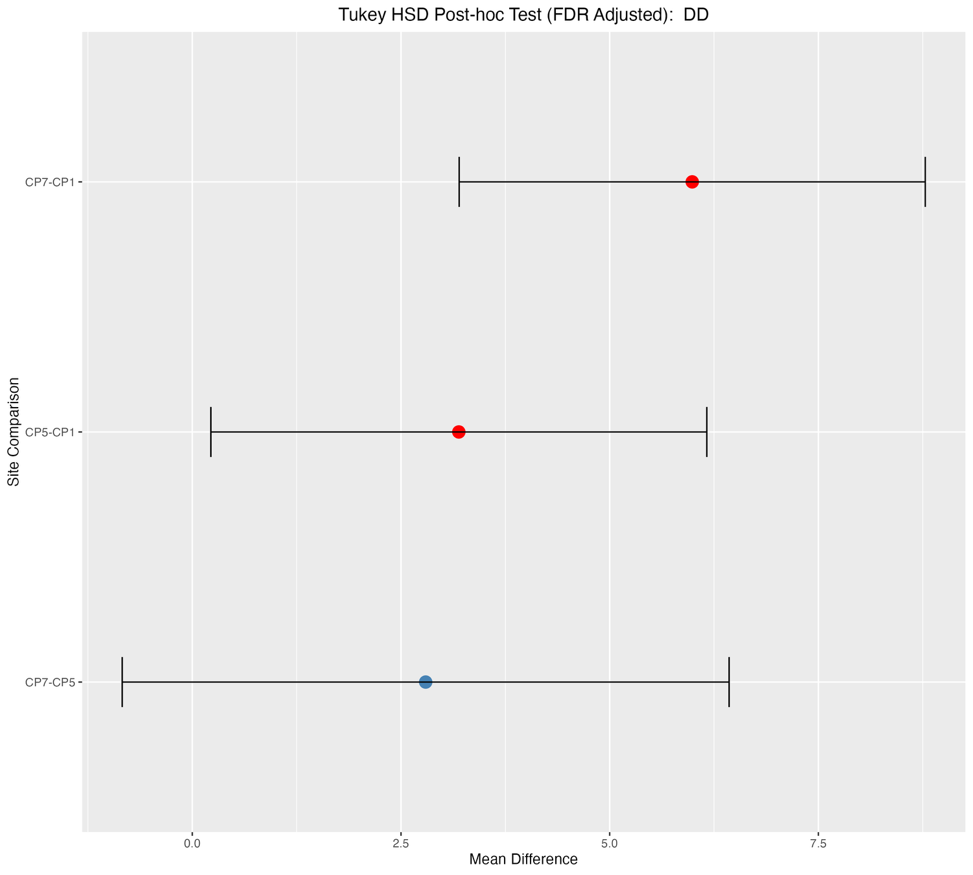


B


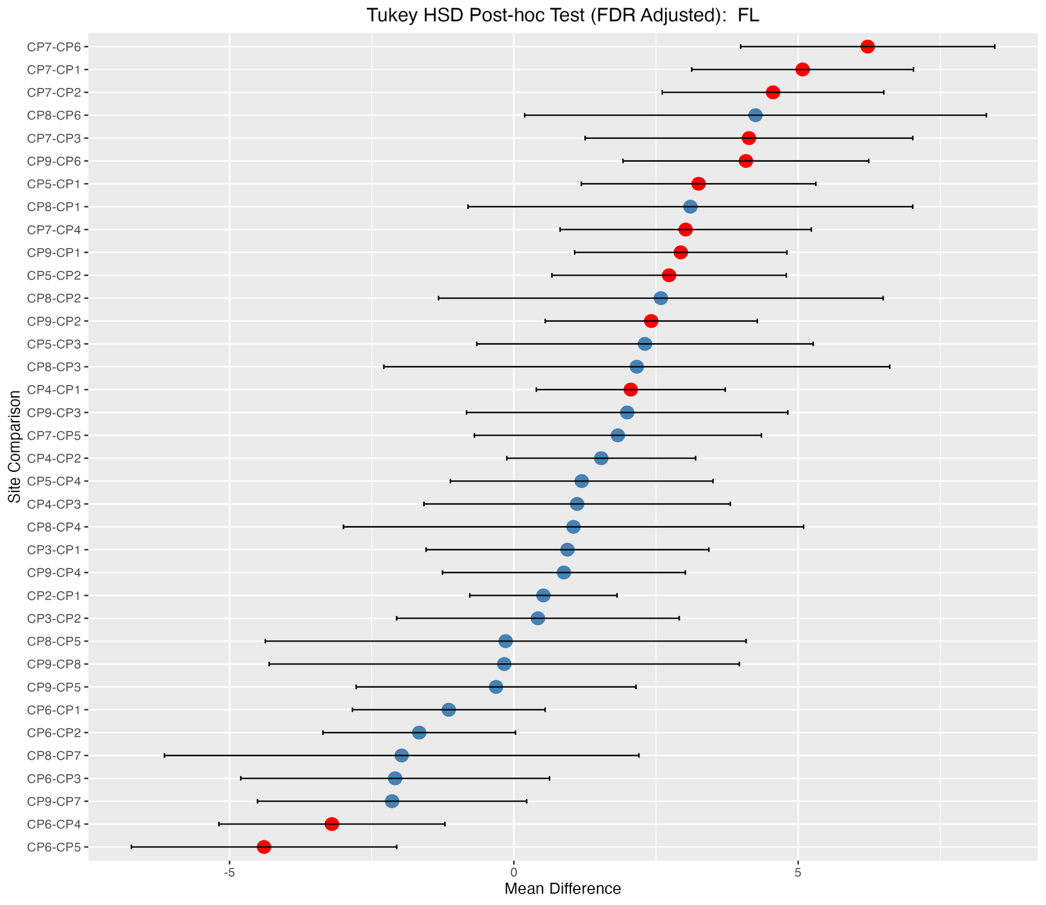


C

Supplement: S10 Fig — (DOCX) [file pone.0335973.s011.docx]

## Supporting Information S11 Fig

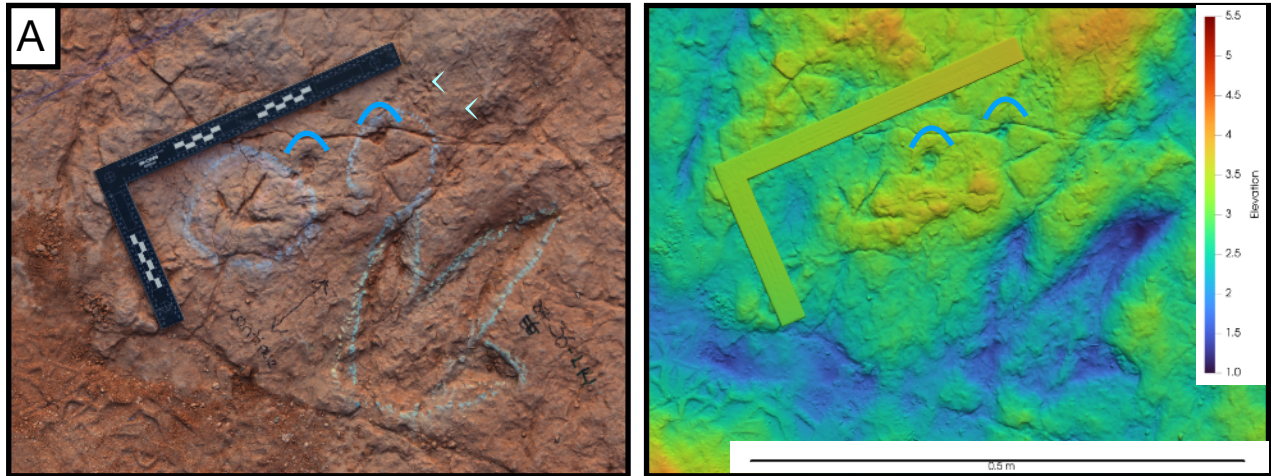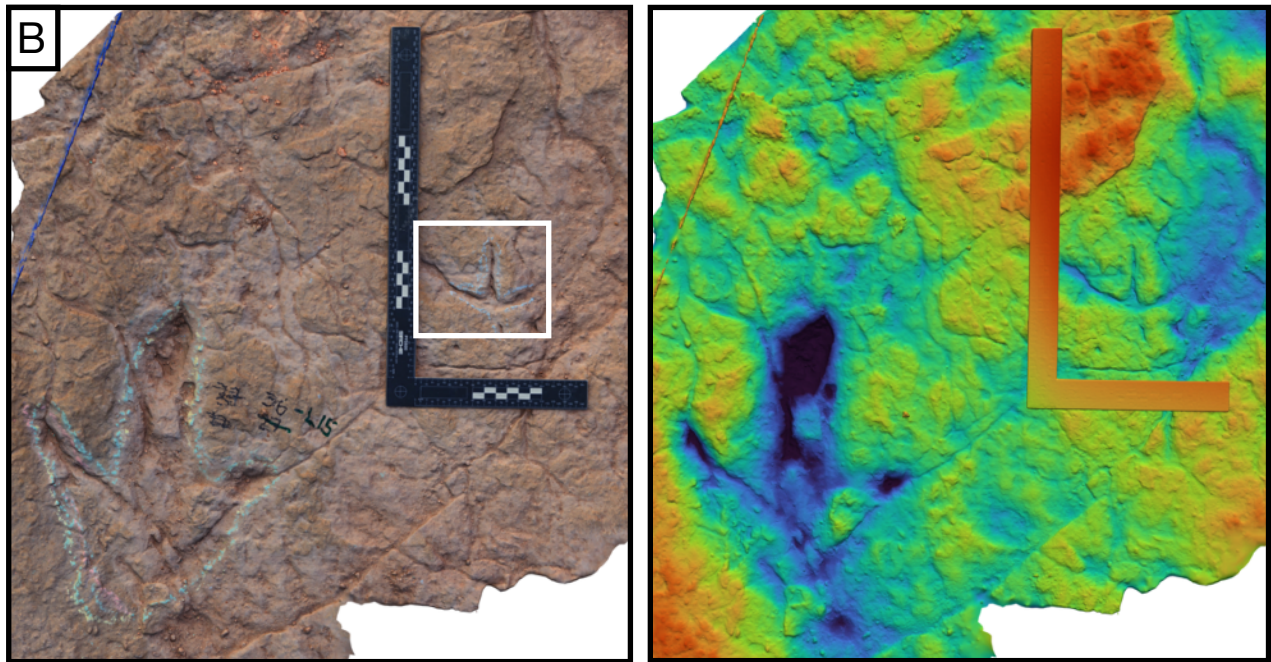

Supplement: S12 Fig — A) Two bird tracks associated with track L4 of trackway CP6–36 encircled in blue chalk. The two blue arched lines indicate two indentations of a left track of style of preservation M1. The scale is 30 cm. B) One bird track (white box) associated with track L15 of trackway CP6–36. Notice the small sinuous galleries resulting from the growth of plant roots. The scale is 30 cm. (PDF) [file pone.0335973.s013.pdf]
