# Supplementary material for: Morphotypes, preservation, and taphonomy of dinosaur footprints, tail traces, and swim tracks in the largest tracksite in the world: Carreras Pampa (Upper Cretaceous), Torotoro National Park, Bolivia
Source: PLoS One. 2025 Dec 3;20(12):e0335973. doi: 10.1371/journal.pone.0335973 (PMC12674571; doi:10.1371/journal.pone.0335973)
Supplement: S7 Fig — This trackway consists of shallow depressions of the style of preservation M6 that we interpret as the filling of actual tracks occurring in a sedimentary layer below the surface. Most of these depressions are elongated with both ends round; some show a narrowing in the middle, and others are slightly curved or round. The scale is 1 m. (PDF) [file pone.0335973.s008.pdf]

Supporting Information S7 Fig

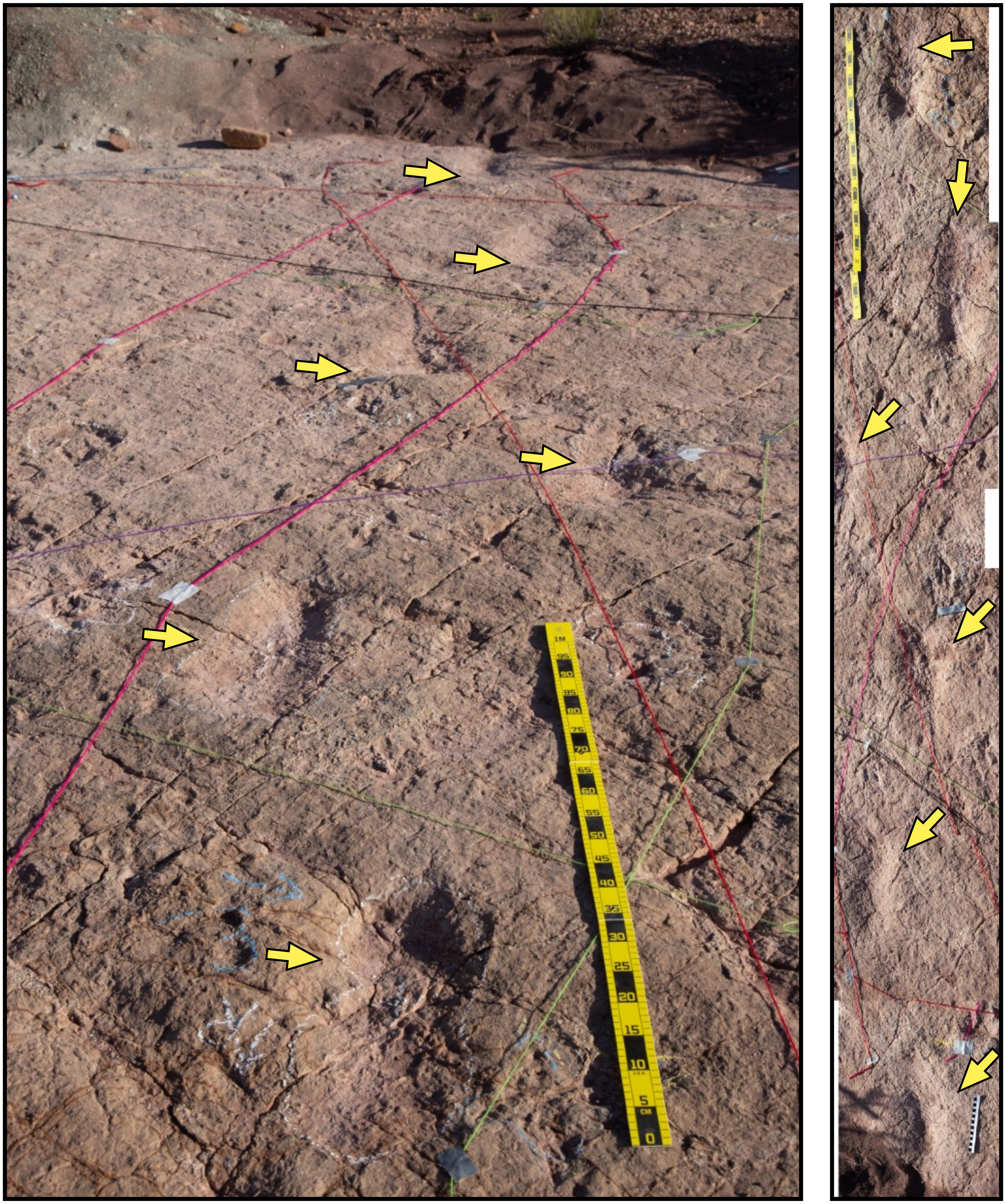

S8 Fig 1. Trackway T22-180: This trackway consists of shallow depressions of the style of preservation M6 that we interpret as the filling of actual tracks occurring in a sedimentary layer below the surface. Most of these depressions are elongated with both ends round; some show a narrowing in the middle, and others are slightly curved or round. The scale is 1 m.
