# Supplementary material for: Morphotypes, preservation, and taphonomy of dinosaur footprints, tail traces, and swim tracks in the largest tracksite in the world: Carreras Pampa (Upper Cretaceous), Torotoro National Park, Bolivia
Source: PLoS One. 2025 Dec 3;20(12):e0335973. doi: 10.1371/journal.pone.0335973 (PMC12674571; doi:10.1371/journal.pone.0335973)
Supplement: S9 Fig — Trackways CP9–81 (orange) and CP9–82 (green) have tail traces associated. Notice that the tail traces cut through the tracks. The arrows indicate the direction of movement. The scale is 1 m. (PDF) [file pone.0335973.s010.pdf]

# Supporting Information S9 Fig

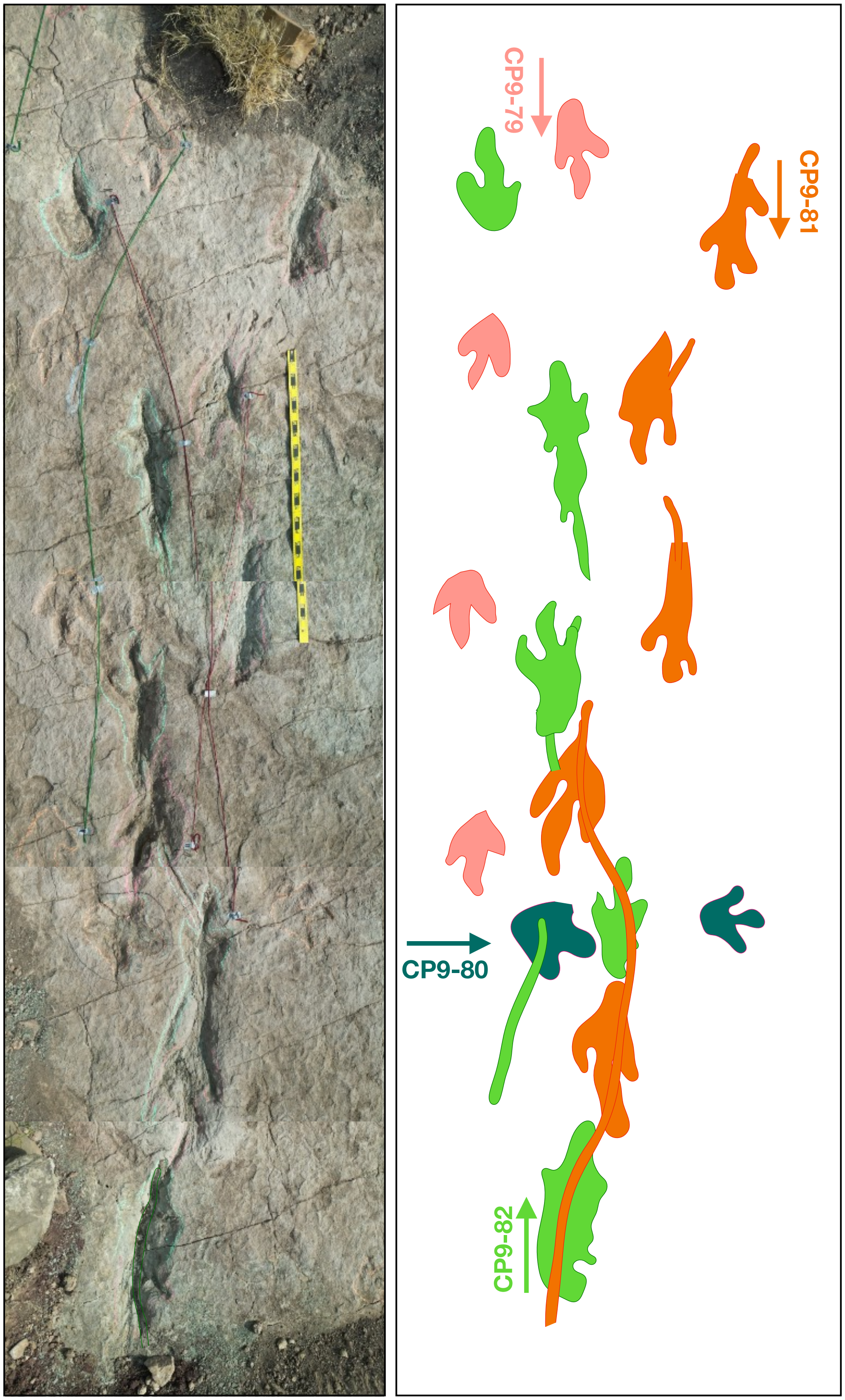


***S11 Fig 1. Trackways CP9-79, 80, 81, and 82.*** Trackways CP9-81 (orange) and CP9-82 (green) have tail traces associated. Notice that the tail traces cut through the tracks. The arrows indicate the direction of movement. The scale is 1 m.
