# Supplementary material for: Morphotypes, preservation, and taphonomy of dinosaur footprints, tail traces, and swim tracks in the largest tracksite in the world: Carreras Pampa (Upper Cretaceous), Torotoro National Park, Bolivia
Source: PLoS One. 2025 Dec 3;20(12):e0335973. doi: 10.1371/journal.pone.0335973 (PMC12674571; doi:10.1371/journal.pone.0335973)
Supplement: S11 Fig — A-B) Swim trackway CP5-S2. Eight sets of swim traces, with L3 missing. The first five sets are placed along the red yarn line and indicated with black arrows. Two additional swim traces occur near the distal end of the ruler. The scale is 1 m. The image on the right shows the first five swim tracks of the succession. C) Swim trackway CP5-S3. Eight sets of swim traces of morphotype S1. Several other swim trackways and isolated swim sets are associated. The scale is 1 m. D) Swim trackway CP5-S4. Four sets of swim traces. Several additional swim traces occur. Sets R1 and L1 consist of single traces of digit III of morphotype S2, whereas sets R2 and L2 consist of three traces each corresponding to digits II, III and IV of style S1. The scale is 1 m. E) Swim trackway CP5-S8. Four sets of swim traces with several missing. Additional swim traces of CP5-S3, CP5-S6, CP5-S7 and CP5-S10 occur. All the swim traces are of morphotype S1. Notice a track of trackway CP5-T7 with a posterior ridge. The scale is 1 m. F) Swim trackway CP6-S1. Five sets of large swim traces of morphotype S1. The scale is 30 m. The measurements correspond to pace, stride length and WAP. G) Swim trackway CP6-S3. Twenty-three sets of large swim traces of morphotype S1. Total length of the exposed trackway is 31.50 m. The traces of this trackway have the largest expulsion rims in all the studied sites. Yellow-black rulers are 1 m long. H) Dimensions of the traces in the swim trackway CP6-S3. Twenty-three sets of exposed swim traces, beginning with R1 and ending with L12. I) Swim trackway CP7-S1. Six sets of small swim traces of morphotype S1. Other additional swim traces are associated with R1 and L1. Swim track L1 cuts across a previously formed theropod track. The scale is 1 m. J) Swim trackway on Site CP1. Long swim traces of morphotype S3. The scale is 2 m long. (PDF) [file pone.0335973.s012.pdf]

Supporting Information S10 Fig

The blue arrows indicate the direction of the swim

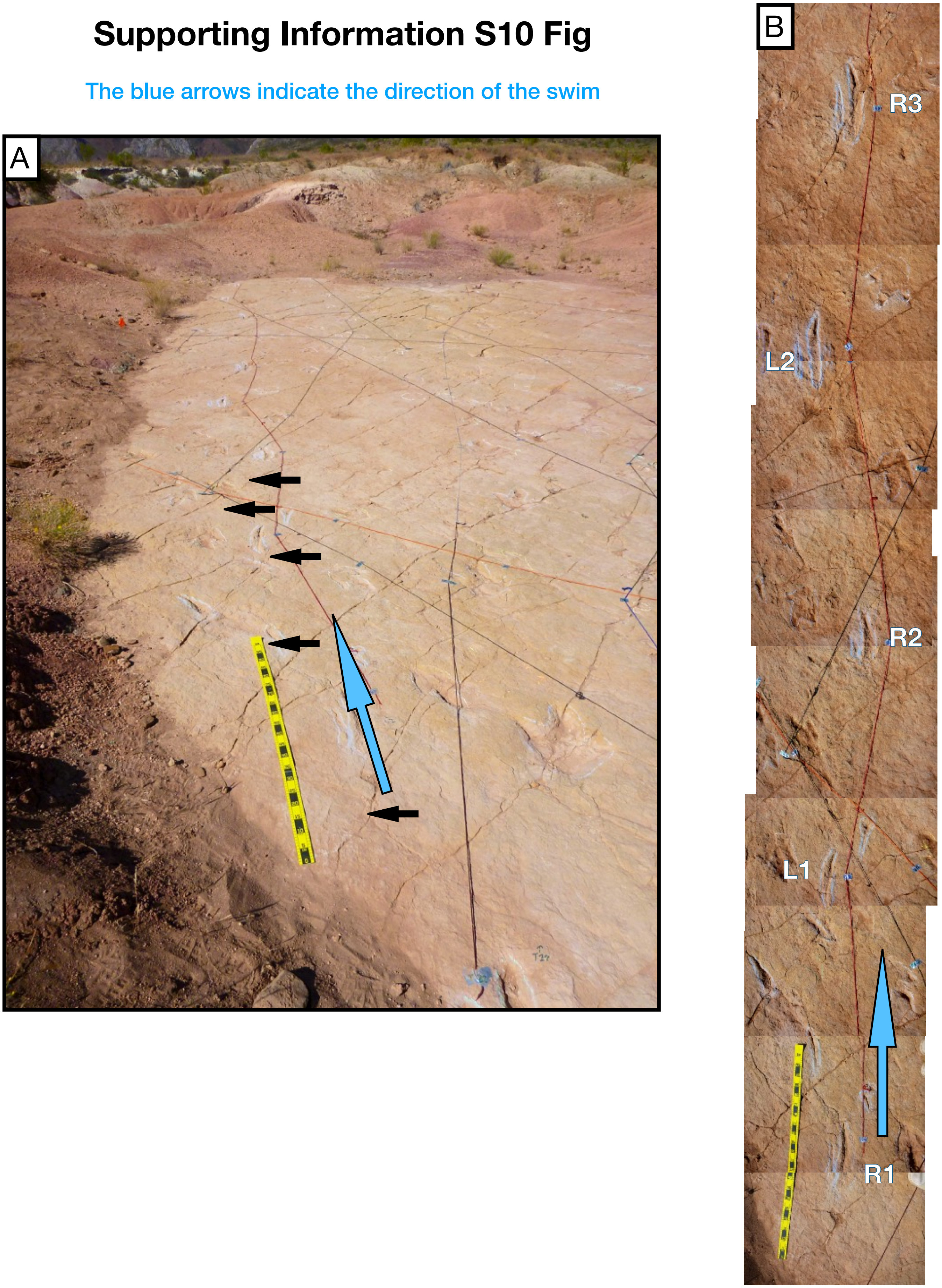

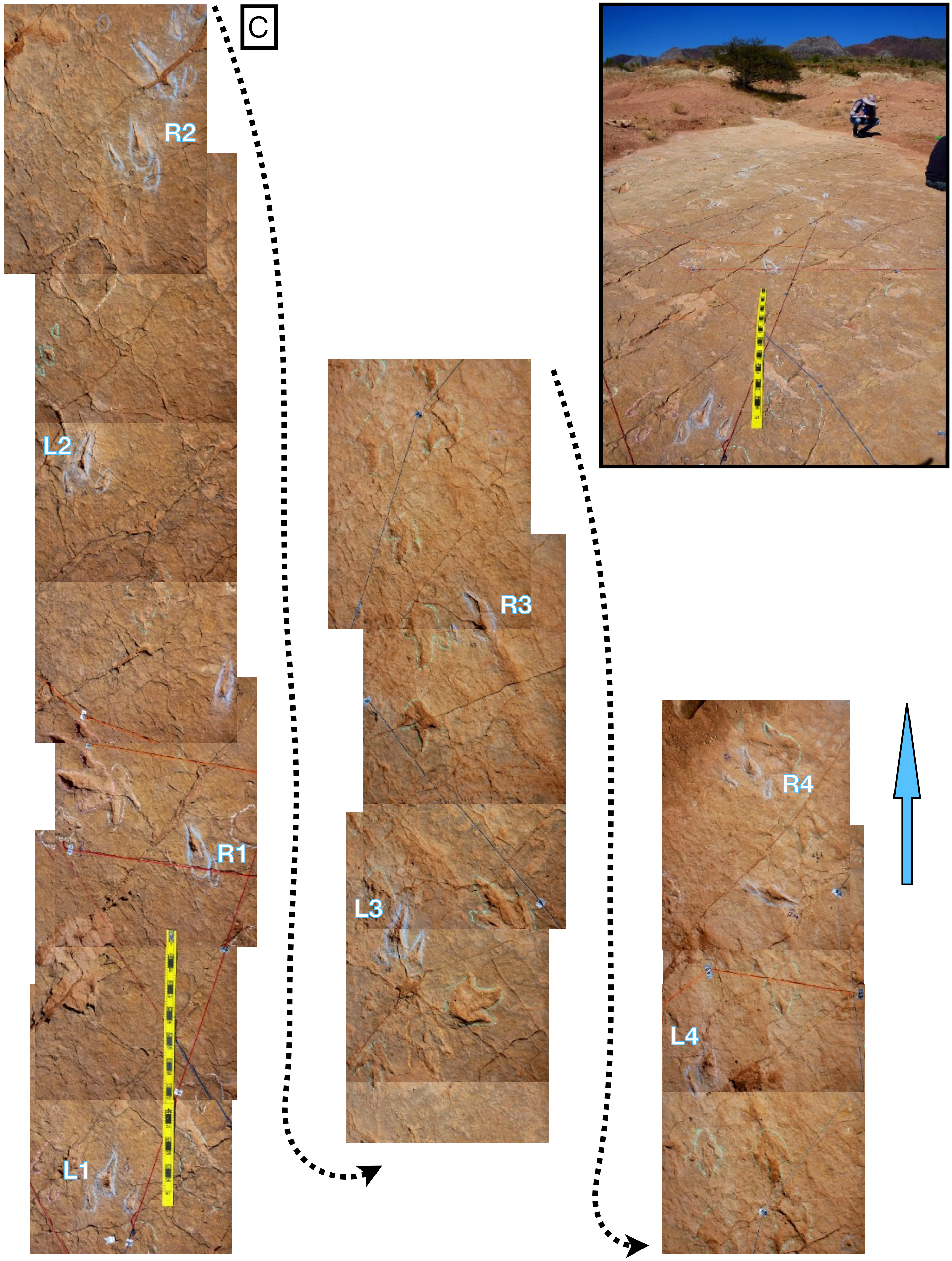
C
R2
L2
R1
L1
R3
L3
R4
L4

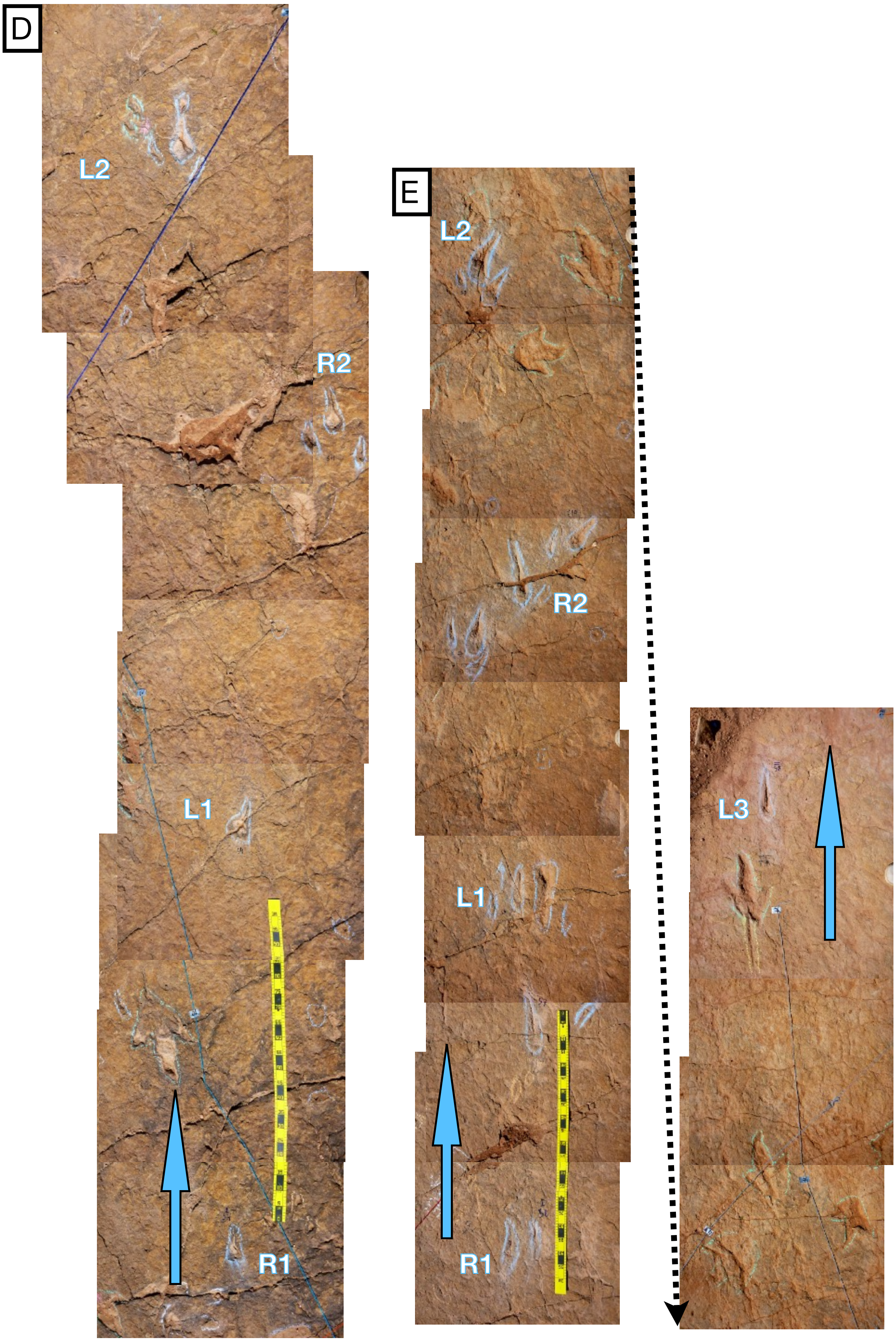
D
L2
R2
L1
R1
E
L2
R2
L1
R1
L3

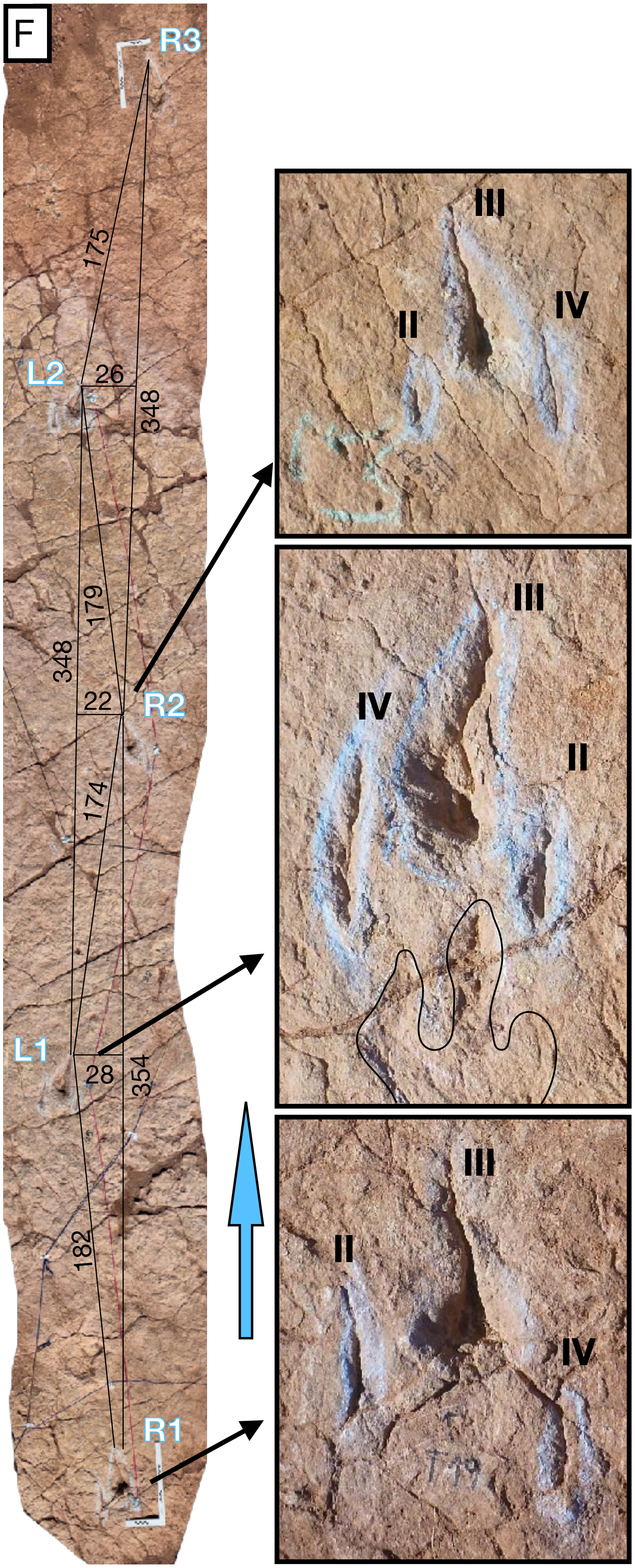

F
R3
175
L2
26
348
179
348
22
R2
174
L1
28
354
182
R1
III
II
IV
III
IV
II
III
II
IV

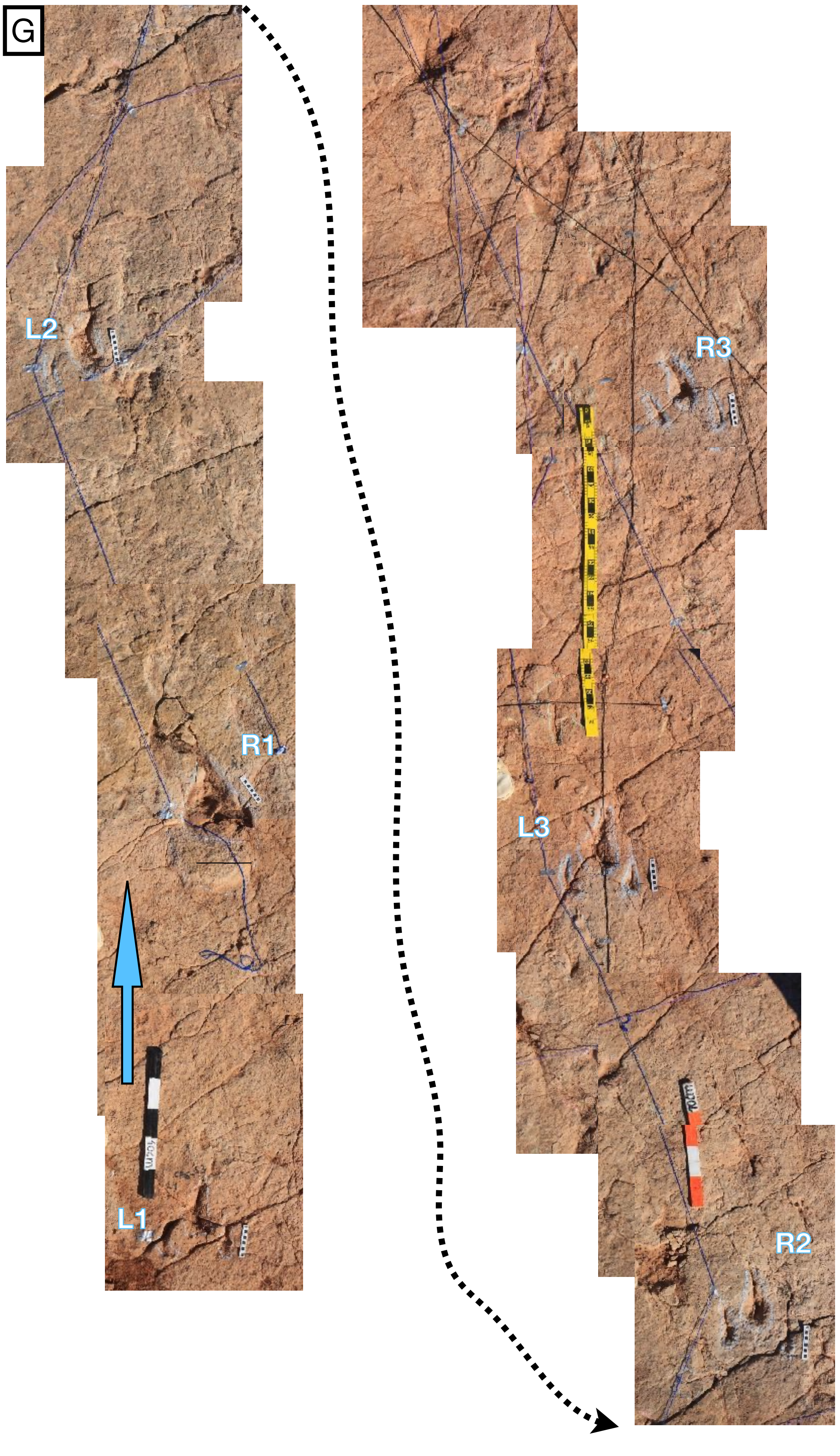
G
L2
R1
L1
R3
L3
R2

Swim trackway CP6-S3: Continued.

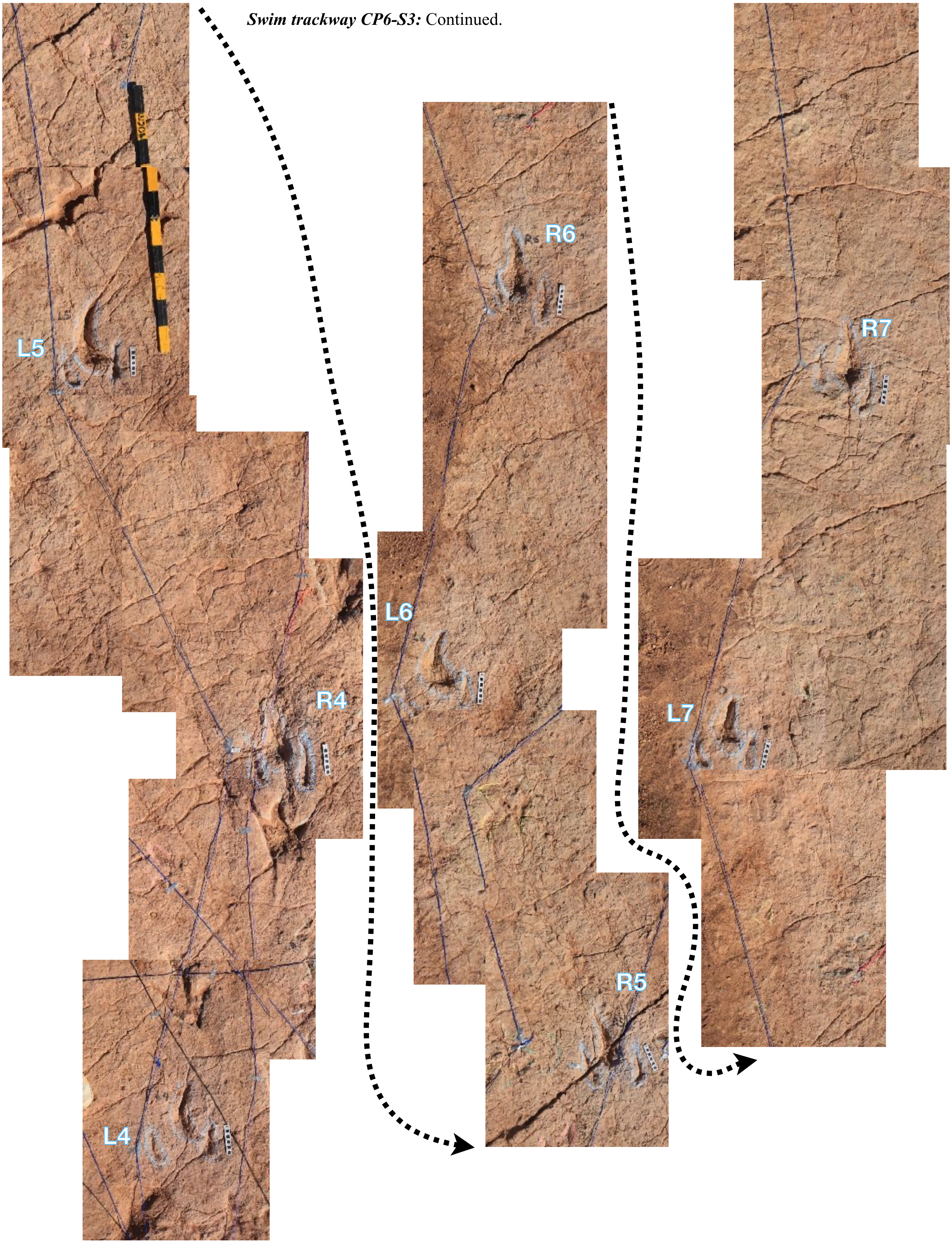

Swim trackway CP6-S3: Continued.

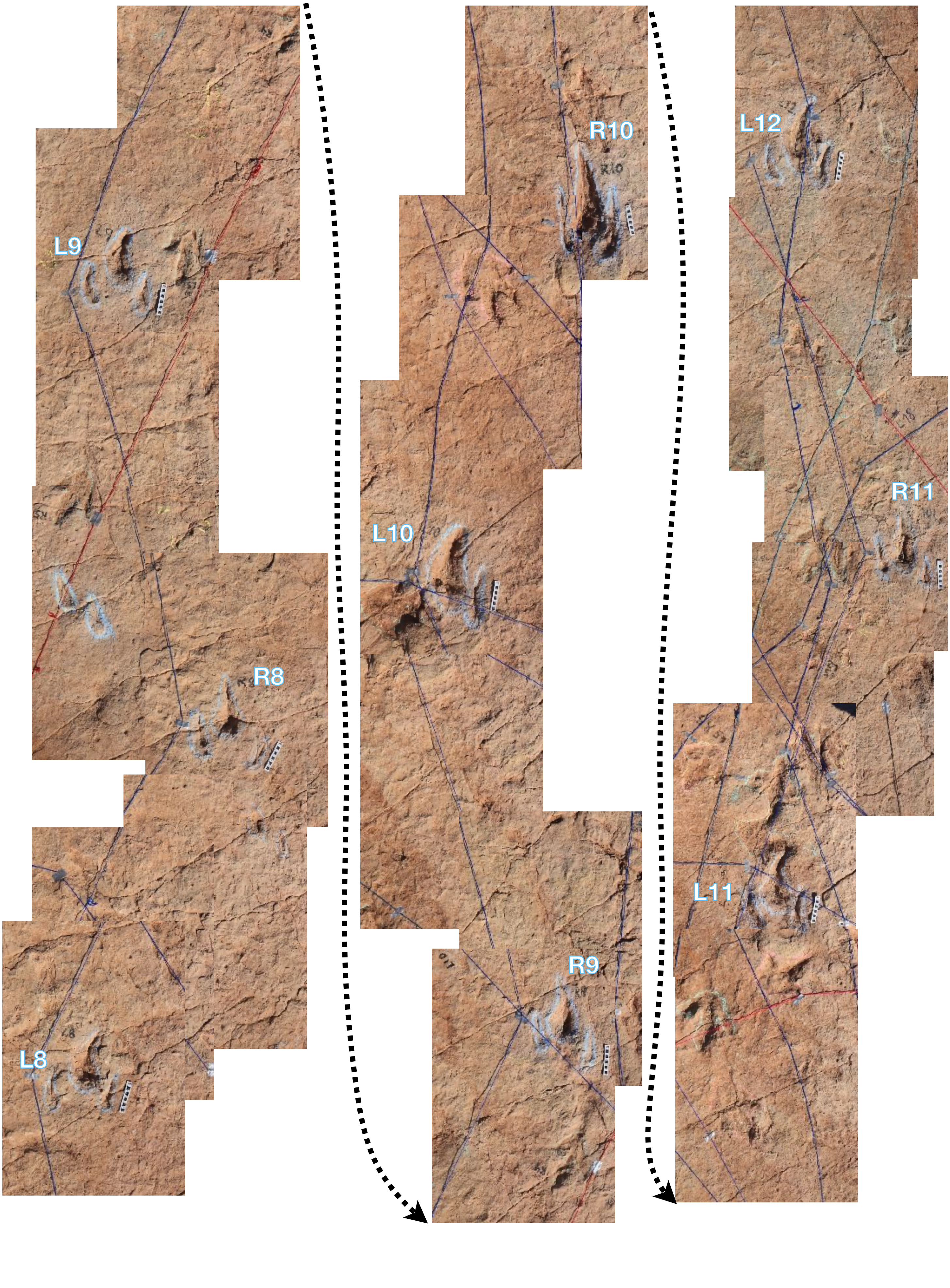

H

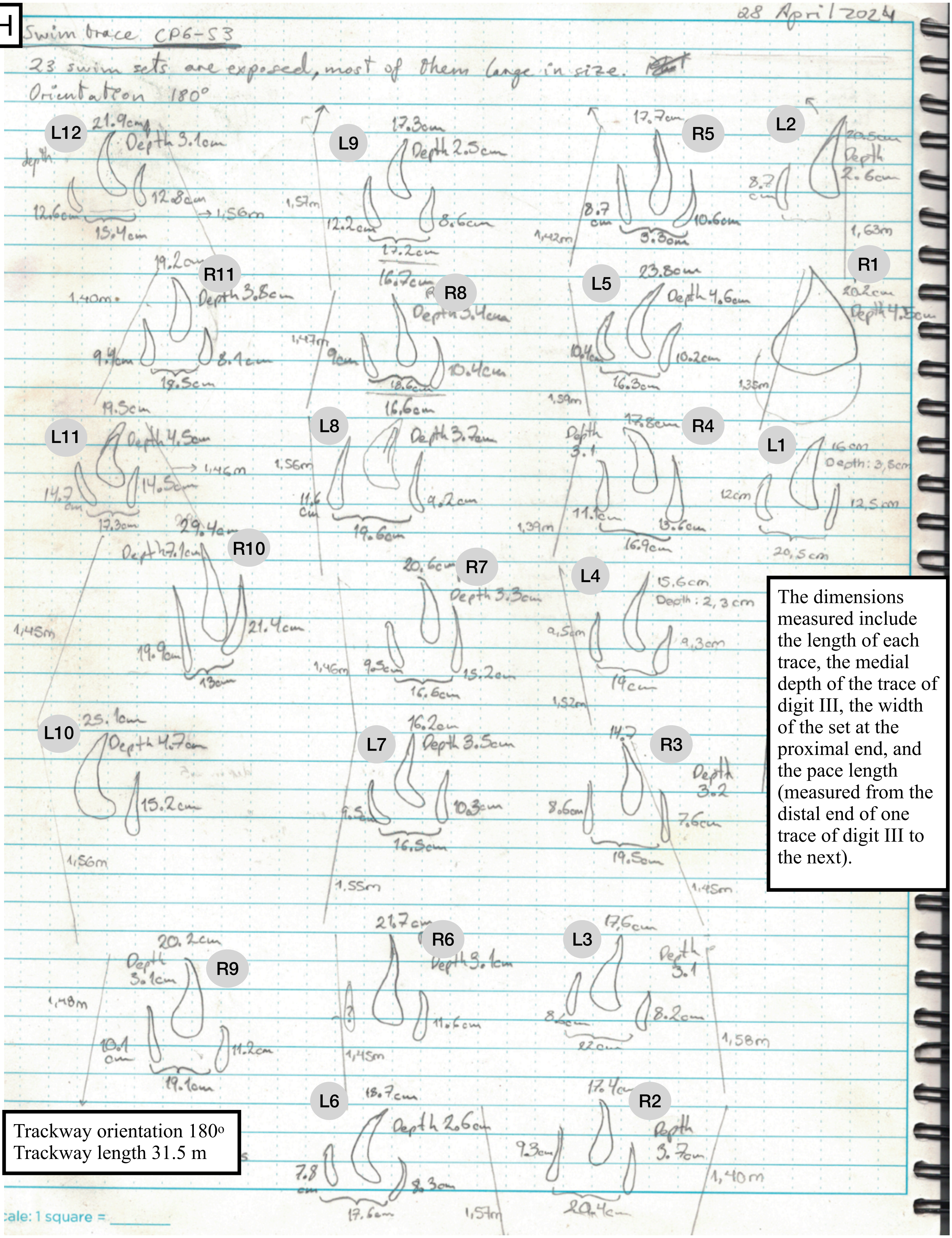
28 April 2024
Swim trace CP6-S3
23 swim sets are exposed, most of them large in size.
Orientation 180°
L12
21.9cm
Depth 3.1cm
12.8cm
12.6cm
15.4cm
1.56m
L9
17.3cm
Depth 2.5cm
12.2cm
8.6cm
17.2cm
1.57m
R5
17.7cm
8.7 cm
10.6cm
9.3cm
1.42m
L2
20.5cm
Depth 2.6cm
8.7 cm
1.63m
R11
19.2cm
Depth 3.8cm
1.40m
9.4cm
8.1cm
18.5cm
R8
16.7cm
Depth 3.4cm
9cm
10.4cm
18.6cm
16.6cm
1.47m
L5
23.8cm
Depth 4.6cm
10.4cm
10.2cm
16.3cm
1.59m
R1
20.2cm
Depth 4.5cm
1.35m
L11
19.5cm
Depth 4.5cm
1.46m
14.7 cm
14.5cm
17.3cm
L8
Depth 3.7cm
1.56m
11.6 cm
9.2cm
19.6cm
R4
17.8cm
Depth 3.1
11.1cm
13.6cm
16.9cm
1.39m
L1
16cm
Depth: 3,8cm
12cm
12.5cm
20.5cm
R10
29.4cm
Depth 7.1cm
21.4cm
19.9cm
13cm
1.45m
R7
20.6cm
Depth 3.3cm
9.5cm
15.2cm
16.6cm
1.46m
L4
15.6cm
Depth: 2,3cm
9.5cm
9.3cm
19cm
1.52m
L10
25.1cm
Depth 4.7cm
15.2cm
1.56m
L7
16.2cm
Depth 3.5cm
9.5cm
10.3cm
16.5cm
1.55m
R3
14.7
Depth 3.2
8.6cm
7.6cm
19.5cm
1.45m
R9
20.2cm
Depth 3.1cm
10.1 cm
11.2cm
19.1cm
1.48m
R6
21.7cm
Depth 3.1cm
11.6cm
1.45m
L3
17.6cm
Depth 3.1
8.6cm
8.2cm
22cm
1.58m
L6
18.7cm
Depth 2.6cm
7.8 cm
8.3cm
17.6cm
1.57m
R2
17.4cm
Depth 3.7cm
9.3cm
20.4cm
1.40m
Scale: 1 square =
The dimensions measured include the length of each trace, the medial depth of the trace of digit III, the width of the set at the proximal end, and the pace length (measured from the distal end of one trace of digit III to the next).
Trackway orientation 180°
Trackway length 31.5 m

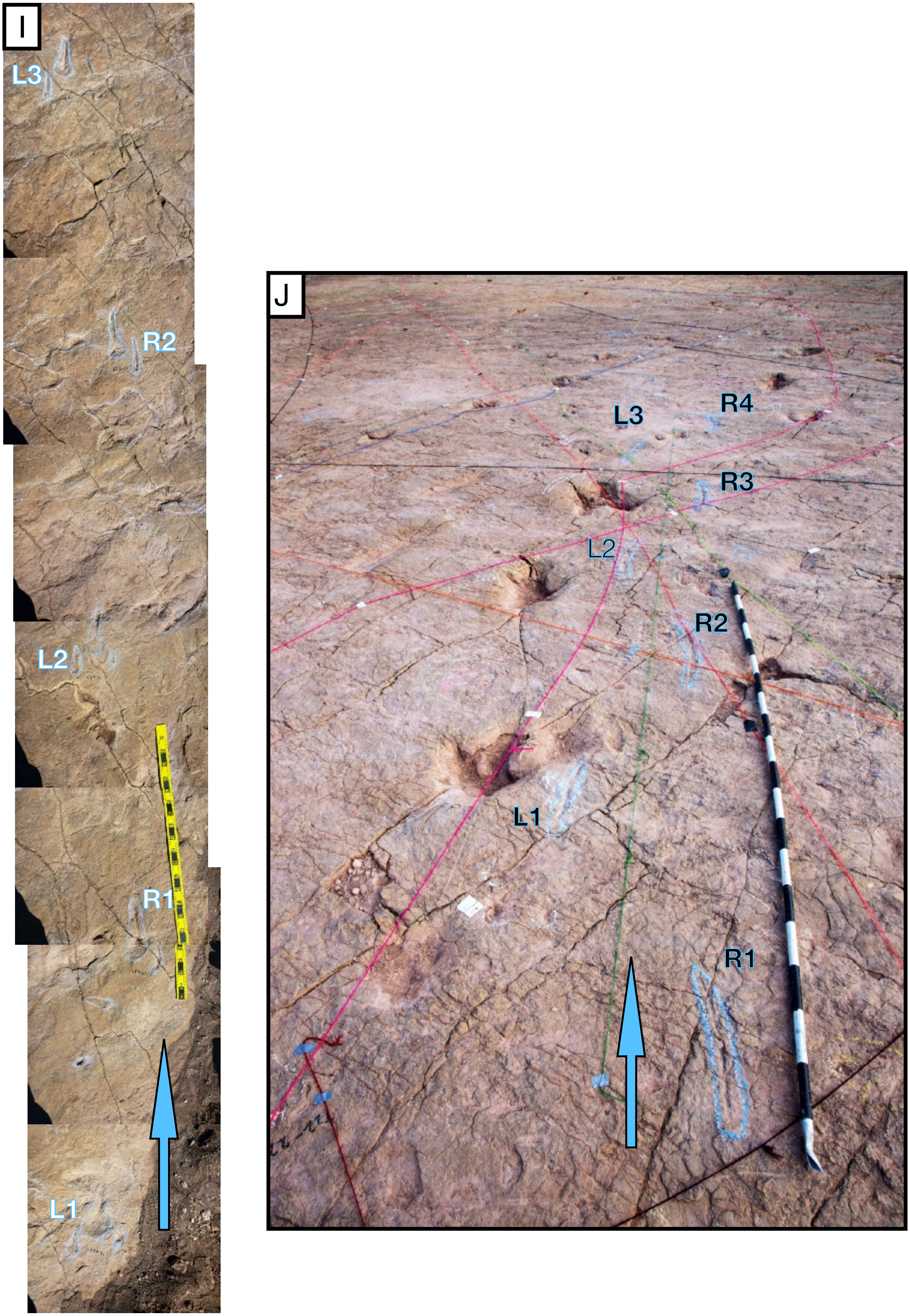
I
L3
R2
L2
R1
L1
J
L3
R4
R3
L2
R2
L1
R1
